# Supplementary material for: Distribution and diversity of classical deacylases in bacteria
Source: Nat Commun. 2024 Nov 3;15:9496. doi: 10.1038/s41467-024-53903-0 (PMC11532494; doi:10.1038/s41467-024-53903-0)
Supplement: Supplementary file 1 — Supplementary Information [file 41467_2024_53903_MOESM1_ESM.pdf]

SUPPLEMENTARY INFORMATION

Distribution and diversity of classical deacylases in bacteria

Leonie G. Graf[1], Carlos Moreno-Yruela[2,6], Chuan Qin[1], Sabrina Schulze[1], Gottfried J. Palm[1], Ole Schmöker[1], Nancy Wang[3], Dianna M. Hocking[3], Leila Jebeli[3], Britta Girbardt[1], Leona Berndt[1], Babett Dörre[1], Daniel M. Weis[1], Markus Janetzky[1], Dirk Albrecht[4], Daniela Zühlke[4], Susanne Sievers[4], Richard A. Strugnell[3], Christian A. Olsen[2], Kay Hofmann[5] and Michael Lammers[1*]

[1]University of Greifswald, Institute of Biochemistry, Department Synthetic and Structural Biochemistry, 17489 Greifswald, Germany
[2]Center for Biopharmaceuticals & Department of Drug Design and Pharmacology, Faculty of Health and Medical Sciences, University of Copenhagen, Copenhagen 2100, Denmark
[3]The University of Melbourne, Department of Microbiology and Immunology, Peter Doherty Institute for Infection and Immunity, Melbourne, Victoria, 3000, Australia
[4]University of Greifswald, Institute of Microbiology, Department of Microbial Physiology and Molecular Biology, 17489 Greifswald, Germany
[5]University of Cologne, Institute for Genetics, 50674 Cologne, Germany
[6]Current address: Institute of Chemical Sciences and Engineering (ISIC), School of Basic Sciences (SB), EPFL, 1015 Lausanne, Switzerland

[*]correspondence should be addressed to Michael Lammers; Email: michael.lammers@uni-greifswald.de; Tel.: 03834-420-4356; Fax: 03834-420-4373

Supplementary Figure 1-28

Supplementary Table 1-10

a

```
1a_Arabidopsis_thaliana_HDA14                                                                     β1
                              1
1a_Arabidopsis_thaliana_HDA14 MSMALI...........................................................VRP..........
1b_Klebsiella_pneumoniae_HdaH MKRKTG.........................................................FFFDE..........
1c_Shinella_sp_DD12_DAC       MTTCLF...........................................................ENP..........
1d_Neisseria_zoodegmatis_DAC  MNKIVRYM................................RLQLRRMLGRQ.......A.RAAWISHP..........
1e_Stentor_coeruleus_HdaH     MSKGINEI.EVDIHEICCLGRHSDLEEIL........NSIDFYADIMLERDEEGFIPYH..LSLIYCRHKCVDLILSL
1f_S._cerevisiae_HOS3         MSSKHSDP.L.ER.......FYKQFQA....FVQNNPNVISAA..RAAAQIPE.....SAKAVVVLSP..........
1g_Homo_sapiens_HDAC4         MSSQSHPDGL.SG.......RDQPVELLNPARVNHMPSTVDVATAL................PLQVAP..........
2a_Homo_sapiens_HDAC2         MAYSQGGG.KK..........................KVCYYYDG.................................
2b_S._cerevisiae_HOS1         MSKLVIST.S......................................................IFQ...........
2c_Bacillus_subtilis_AcuC     MRDSV...................................FIYSP.................................
2d_Chromatiales_bacterium_Acu MFVAK......................................DE.................................
3_Legionella_cherrii_ApaH     MRHKYFF...........................................................KE..........
4_Pseudomonas_sp_M30-35_ApaH  MLTIYS.............................................................D..........
5a_Acinetobacter_wuhouensis_H MLNVCF............................................................SP..........
5b_Vibrio_cholerae_HdaH       MIPLIYHP.IY...................................................................
5c_Moraxella_bovis_HDAC       MFFKIAHC.HE...................................................................
5d_Turneriella_parva_HdaH     MSRKSAEL........................................................VYSP..........
5e_Nannocystis_exedens_AcuC   MTTGEKIF.R.QL...........................RRRANQMFARLRP.....P.PAAFVYGP..........
5f_Homo_sapiens_HDAC11        MLHTTQLY.Q.H......................................VPE.....T.RWPIVYSP..........
5g_Bacteriovorax_stolpii_AcuC MIFYHPDC.DLR..................................................................
```

```
1a_Arabidopsis_thaliana_HDA14
                                10                      20
1a_Arabidopsis_thaliana_HDA14 ..F...........F...VPGSAGISGSRN................................................
1b_Klebsiella_pneumoniae_HdaH ..R...........C...FWHSTG.LHAVTLPVGG..........................................
1c_Shinella_sp_DD12_DAC       ..I...........F...LE..........................................................
1d_Neisseria_zoodegmatis_DAC  ..L...........F...LHYGC.......................................................
1e_Stentor_coeruleus_HdaH     SIDFQVPYQGFMPIHLAMACAGFPEC....HEDL........I.........Q.........................
1f_S._cerevisiae_HOS3         ..Y...........S...............................................................
1g_Homo_sapiens_HDAC4         ..S...........A...VPMDLRLDHQFSLPVAEPALREQQL.........QQELLALKQKQQIQRQILIAEFQRQH
2a_Homo_sapiens_HDAC2         ..DI..............GNYYYGQG....HPMK........P...................................
2b_S._cerevisiae_HOS1         ...S..............QVA..D.L....LPCN........N...................................
2c_Bacillus_subtilis_AcuC     ..SY..............QTYMFHQE....HPFN........Q...................................
2d_Chromatiales_bacterium_Acu ..RI..............AAYGFGEG....HPFG........L...................................
3_Legionella_cherrii_ApaH     ..K..................VSPES....HKEK........NQECLIQIPSA.........................
4_Pseudomonas_sp_M30-35_ApaH  ..D...........H...HLHHG......SCELID...........................................
5a_Acinetobacter_wuhouensis_H ..R..................YFAQT....HTNS........ME..................................
5b_Vibrio_cholerae_HdaH       ..................SQLDLPVG....HRYP........I...................................
5c_Moraxella_bovis_HDAC       ...F...............ALPLPDG....HRFP........M...................................
5d_Turneriella_parva_HdaH     ..IY..............DLS..DYT....HVIS........A...................................
5e_Nannocystis_exedens_AcuC   ..H...........Y...ECSW........................................................
5f_Homo_sapiens_HDAC11        ..R...........Y...NITFM.......................................................
5g_Bacteriovorax_stolpii_AcuC ...................FSEYGIE....IPIV........D...................................
```

```
1a_Arabidopsis_thaliana_HDA14

1a_Arabidopsis_thaliana_HDA14 ..............................................................................
1b_Klebsiella_pneumoniae_HdaH ..............................................................................
1c_Shinella_sp_DD12_DAC       ..............................................................................
1d_Neisseria_zoodegmatis_DAC  ..............................................................................
1e_Stentor_coeruleus_HdaH     .................TVNCL.LKHKQDVTSRDR...........................................
1f_S._cerevisiae_HOS3         ..............................................................................
1g_Homo_sapiens_HDAC4         EQLSRQHEAQLHEHIKQQQEMLAMKHQQELLEHQRKLERHRQEQELEKQHREQKLQQLKNKEKGKESAVASTEVKMKL
2a_Homo_sapiens_HDAC2         ..............................................................................
2b_S._cerevisiae_HOS1         ..............................................................................
2c_Bacillus_subtilis_AcuC     ..............................................................................
2d_Chromatiales_bacterium_Acu ..............................................................................
3_Legionella_cherrii_ApaH     ..............................................................................
4_Pseudomonas_sp_M30-35_ApaH  ..............................................................................
5a_Acinetobacter_wuhouensis_H ..............................................................................
5b_Vibrio_cholerae_HdaH       ..............................................................................
5c_Moraxella_bovis_HDAC       ..............................................................................
5d_Turneriella_parva_HdaH     ..............................................................................
5e_Nannocystis_exedens_AcuC   ..............................................................................
5f_Homo_sapiens_HDAC11        ..............................................................................
5g_Bacteriovorax_stolpii_AcuC ..............................................................................
```

```
1a_Arabidopsis_thaliana_HDA14
                                                     30        40
1a_Arabidopsis_thaliana_HDA14 .................ICKKNQWRKYLLKPSGSSINC........................SFS.............
1b_Klebsiella_pneumoniae_HdaH ..........................WVQ.................................................
1c_Shinella_sp_DD12_DAC       ..............................................................................
1d_Neisseria_zoodegmatis_DAC  ..............................................................................
1e_Stentor_coeruleus_HdaH     ...........................LGRT..................ALH.IVCAYGLADLIPILVQAGVKPELKD
1f_S._cerevisiae_HOS3         ..............................................................................
1g_Homo_sapiens_HDAC4         QEFVLNKKKALAHRNLNHCISSDPRYWYGKTQHSSLDQSSPPQSGVSTSYNHPVLGMYDAKDDFPLRK.TASEPNLK.
2a_Homo_sapiens_HDAC2         ..............................................................................
2b_S._cerevisiae_HOS1         ..............................................................................
2c_Bacillus_subtilis_AcuC     ..............................................................................
2d_Chromatiales_bacterium_Acu ..............................................................................
3_Legionella_cherrii_ApaH     ..............................................................................
4_Pseudomonas_sp_M30-35_ApaH  ..............................................................................
5a_Acinetobacter_wuhouensis_H ..............................................................................
5b_Vibrio_cholerae_HdaH       ..............................................................................
5c_Moraxella_bovis_HDAC       ..............................................................................
5d_Turneriella_parva_HdaH     ..............................................................................
5e_Nannocystis_exedens_AcuC   ..............................................................................
5f_Homo_sapiens_HDAC11        ..............................................................................
5g_Bacteriovorax_stolpii_AcuC ..............................................................................
```

```
1a_Arabidopsis_thaliana_HDA14

1a_Arabidopsis_thaliana_HDA14       ..............................................................................
1b_Klebsiella_pneumoniae_HdaH       ..............................................................................
1c_Shinella_sp_DD12_DAC             ..............................................................................
1d_Neisseria_zoodegmatis_DAC        ..............................................................................
1e_Stentor_coeruleus_HdaH           FSGKMPIHYAIENYQAECFKQLLQEMG............................................SDMFFCT
1f_S._cerevisiae_HOS3               ..............................................................................
1g_Homo_sapiens_HDAC4               ..........LRS.......RLKQKVAERRSSPLLRRKDGPVVTALKKRPLDVTDSACSSAPGSGPSSPNNSSG.SVS
2a_Homo_sapiens_HDAC2               ..............................................................................
2b_S._cerevisiae_HOS1               ..............................................................................
2c_Bacillus_subtilis_AcuC           ..............................................................................
2d_Chromatiales_bacterium_Acu       ..............................................................................
3_Legionella_cherrii_ApaH           ..............................................................................
4_Pseudomonas_sp_M30-35_ApaH        ..............................................................................
5a_Acinetobacter_wuhouensis_H       ..............................................................................
5b_Vibrio_cholerae_HdaH             ..............................................................................
5c_Moraxella_bovis_HDAC             ..............................................................................
5d_Turneriella_parva_HdaH           ..............................................................................
5e_Nannocystis_exedens_AcuC         ..............................................................................
5f_Homo_sapiens_HDAC11              ..............................................................................
5g_Bacteriovorax_stolpii_AcuC       ..............................................................................
```

```
1a_Arabidopsis_thaliana_HDA14

1a_Arabidopsis_thaliana_HDA14       ..............................................................................
1b_Klebsiella_pneumoniae_HdaH       ......................................................PPA.....................
1c_Shinella_sp_DD12_DAC             ..............................................................................
1d_Neisseria_zoodegmatis_DAC        ..............................................................................
1e_Stentor_coeruleus_HdaH           DN............................................................................
1f_S._cerevisiae_HOS3               ..............................................................................
1g_Homo_sapiens_HDAC4               AENGIAPAVPSIPAETSLAHRLVAREGSAAPLPLYTSPSLPNITLGLPATGPSAGTAGQQDAERLTLPALQQRLSLFP
2a_Homo_sapiens_HDAC2               ..............................................................................
2b_S._cerevisiae_HOS1               ..............................................................................
2c_Bacillus_subtilis_AcuC           ..............................................................................
2d_Chromatiales_bacterium_Acu       ..............................................................................
3_Legionella_cherrii_ApaH           ..............................................................................
4_Pseudomonas_sp_M30-35_ApaH        ..............................................................................
5a_Acinetobacter_wuhouensis_H       ..............................................................................
5b_Vibrio_cholerae_HdaH             ..............................................................................
5c_Moraxella_bovis_HDAC             ..............................................................................
5d_Turneriella_parva_HdaH           ..............................................................................
5e_Nannocystis_exedens_AcuC         ..............................................................................
5f_Homo_sapiens_HDAC11              ..............................................................................
5g_Bacteriovorax_stolpii_AcuC       ..............................................................................
```

```
                                                                                                   α1
1a_Arabidopsis_thaliana_HDA14                                                   TT                 ℓℓℓℓℓℓ
                                                                              50                    60
1a_Arabidopsis_thaliana_HDA14       ...........................................TEKNPLL..................PSIQQLADAR
1b_Klebsiella_pneumoniae_HdaH       ..............................................................................
1c_Shinella_sp_DD12_DAC             ..............................................................................
1d_Neisseria_zoodegmatis_DAC        ..............................................................................
1e_Stentor_coeruleus_HdaH           ...........................................RGDKPIHTSVRAGSWEC..................
1f_S._cerevisiae_HOS3               ..............................................................................
1g_Homo_sapiens_HDAC4               GTHLTPYLSTSPLERDGGAAHSPLLQHMVLLEQPPAQAPLVTGLGALPLH.......AQSLVGADRVSPSIHKLRQHR
2a_Homo_sapiens_HDAC2               ..............................................................................
2b_S._cerevisiae_HOS1               ..............................................................................
2c_Bacillus_subtilis_AcuC           ..............................................................................
2d_Chromatiales_bacterium_Acu       ..............................................................................
3_Legionella_cherrii_ApaH           ..............................................................................
4_Pseudomonas_sp_M30-35_ApaH        ..............................................................................
5a_Acinetobacter_wuhouensis_H       ..............................................................................
5b_Vibrio_cholerae_HdaH             ..............................................................................
5c_Moraxella_bovis_HDAC             ..............................................................................
5d_Turneriella_parva_HdaH           ..............................................................................
5e_Nannocystis_exedens_AcuC         ..............................................................................
5f_Homo_sapiens_HDAC11              ..............................................................................
5g_Bacteriovorax_stolpii_AcuC       ..............................................................................
```

```
                                                                                   β2
1a_Arabidopsis_thaliana_HDA14                                                   ------>
                                                                                     70
1a_Arabidopsis_thaliana_HDA14       ...............................................LIYSVSA........................
1b_Klebsiella_pneumoniae_HdaH       ..............................................................................
1c_Shinella_sp_DD12_DAC             ..............................................................................
1d_Neisseria_zoodegmatis_DAC        ..............................................................................
1e_Stentor_coeruleus_HdaH           ...........................................FNILITFGSEEML......................
1f_S._cerevisiae_HOS3               ..............................................................................
1g_Homo_sapiens_HDAC4               PLGRTQSAPLPQNAQALQHLVIQQQHQQFLEKHKQQFQQQQLQMNKIIPKPSEPARQPESHPEETEEELREHQALLDE
2a_Homo_sapiens_HDAC2               ..............................................................................
2b_S._cerevisiae_HOS1               ..............................................................................
2c_Bacillus_subtilis_AcuC           ..............................................................................
2d_Chromatiales_bacterium_Acu       ..............................................................................
3_Legionella_cherrii_ApaH           ..............................................................................
4_Pseudomonas_sp_M30-35_ApaH        ..............................................................................
5a_Acinetobacter_wuhouensis_H       ..............................................................................
5b_Vibrio_cholerae_HdaH             ..............................................................................
5c_Moraxella_bovis_HDAC             ..............................................................................
5d_Turneriella_parva_HdaH           ..............................................................................
5e_Nannocystis_exedens_AcuC         ..............................................................................
5f_Homo_sapiens_HDAC11              ..............................................................................
5g_Bacteriovorax_stolpii_AcuC       ..............................................................................
```

```
1a_Arabidopsis_thaliana_HDA14

1a_Arabidopsis_thaliana_HDA14      .............................................................................
1b_Klebsiella_pneumoniae_HdaH      .............................................................................
1c_Shinella_sp_DD12_DAC            .............................................................................
1d_Neisseria_zoodegmatis_DAC       .............................................................................
1e_Stentor_coeruleus_HdaH          .............................................................................
1f_S._cerevisiae_HOS3              .............................................................................
1g_Homo_sapiens_HDAC4              PYLDRLPGQKEAHAQAGVQVKQEPIESDEEEAEPPREVEPGQRQPSEQELLFRQQALLEQQRIHQLRNYQASMEAAG
2a_Homo_sapiens_HDAC2              .............................................................................
2b_S._cerevisiae_HOS1              .............................................................................
2c_Bacillus_subtilis_AcuC          .............................................................................
2d_Chromatiales_bacterium_Acu      .............................................................................
3_Legionella_cherrii_ApaH          .............................................................................
4_Pseudomonas_sp_M30-35_ApaH       .............................................................................
5a_Acinetobacter_wuhouensis_H      .............................................................................
5b_Vibrio_cholerae_HdaH            .............................................................................
5c_Moraxella_bovis_HDAC            .............................................................................
5d_Turneriella_parva_HdaH          .............................................................................
5e_Nannocystis_exedens_AcuC        .............................................................................
5f_Homo_sapiens_HDAC11             .............................................................................
5g_Bacteriovorax_stolpii_AcuC      .............................................................................
```

```
                                                                                 T..T                 
1a_Arabidopsis_thaliana_HDA14                                                       80           90
1a_Arabidopsis_thaliana_HDA14      ..................................................ALGHNKE..SHPE..CSA....RVPAI
1b_Klebsiella_pneumoniae_HdaH      .......................................................GG..GHAE..SPE....TKRRM
1c_Shinella_sp_DD12_DAC            ...................................................HH.TPE..GHPE..RSD....RIRAI
1d_Neisseria_zoodegmatis_DAC       .......................................................GN..GHPE..SAE....RIKAI
1e_Stentor_coeruleus_HdaH          ....................................................NENNEFEETPE..EVA....RNCGL
1f_S._cerevisiae_HOS3              ........................................................L..QHVF..PREWVTKSYRK.
1g_Homo_sapiens_HDAC4              IPVSFGGHRPLSRAQSSPASATFPVSVQEPPTKPRFTTGLVYDTLMLKHQCTCG.SSS..SHPE..HAG....RIQSI
2a_Homo_sapiens_HDAC2              ...................................................................H....RIRMT
2b_S._cerevisiae_HOS1              ...................................................................HQ...KSQLT
2c_Bacillus_subtilis_AcuC          ...................................................................Q....RVLLT
2d_Chromatiales_bacterium_Acu      ...................................................................D....RHAVF
3_Legionella_cherrii_ApaH          ..................................................KD..IQQ..MHGM..PAG....ADEDQ
4_Pseudomonas_sp_M30-35_ApaH       .......................................................GK..LMPCFEMPK....RADHI
5a_Acinetobacter_wuhouensis_H      ........................................................................KLLAV
5b_Vibrio_cholerae_HdaH            ...................................................................N....KYRLL
5c_Moraxella_bovis_HDAC            ...................................................................A....KYELL
5d_Turneriella_parva_HdaH          ...................................................................G....KYRQL
5e_Nannocystis_exedens_AcuC        ........................................................P..GSAV..DEV....RGERI
5f_Homo_sapiens_HDAC11             ....................................................G.LEK..LHPF..DAG....KWGKV
5g_Bacteriovorax_stolpii_AcuC      ...................................................................E....RASHV
```

```
                                   α2
1a_Arabidopsis_thaliana_HDA14

1a_Arabidopsis_thaliana_HDA14      VNALEMNEL....................................................................
1b_Klebsiella_pneumoniae_HdaH      KKLMDVSGL....................................................................
1c_Shinella_sp_DD12_DAC            NLALEHERF....................................................................
1d_Neisseria_zoodegmatis_DAC       EAEIKKQGF....................................................................
1e_Stentor_coeruleus_HdaH          YNEFVRARQ......G.............................DVFPSSAYKGTLLITDETCKLHASIHESLKTP
1f_S._cerevisiae_HOS3              TIVERPERL....................................................................
1g_Homo_sapiens_HDAC4              WSRLQETGL....................................................................
2a_Homo_sapiens_HDAC2              HNLLLNYG.....................................................................
2b_S._cerevisiae_HOS1              YSLINAYDL....................................................................
2c_Bacillus_subtilis_AcuC          YDLLKTIN.....................................................................
2d_Chromatiales_bacterium_Acu      HNELARAG.....................................................................
3_Legionella_cherrii_ApaH          FERLKHMTQVIATTQSKDPSLPVVTTDRVELPEHWNQLFAAMKKGDE..............................
4_Pseudomonas_sp_M30-35_ApaH       LQRV...KD....................................................................
5a_Acinetobacter_wuhouensis_H      AEQLQQFNQ....................................N...............................
5b_Vibrio_cholerae_HdaH            YEEIVRQRE......Q...........................SEA...............................
5c_Moraxella_bovis_HDAC            PKKLIADG.....................................................................
5d_Turneriella_parva_HdaH          YDAL....K....................................................................
5e_Nannocystis_exedens_AcuC        LTALAAEGL....................................................................
5f_Homo_sapiens_HDAC11             INFLKEEKL....................................................................
5g_Bacteriovorax_stolpii_AcuC      FSALKYLY.....................................P...............................
```

```
                                                              α3       η1  β3                      α4
1a_Arabidopsis_thaliana_HDA14
                                                             100       110                     120
1a_Arabidopsis_thaliana_HDA14      .............................TPKFRGSQI.LEL....ANFKT.............ATV.....EDIAN
1b_Klebsiella_pneumoniae_HdaH      .............................TP......Q.LAL....RSAAP.............ASL.....EDLRR
1c_Shinella_sp_DD12_DAC            .............................SP......L.L.R....EKAPQ.............ANE.....DFVLL
1d_Neisseria_zoodegmatis_DAC       .............................WD......V.FQK....IEAAE.............VSD.....SQLAL
1e_Stentor_coeruleus_HdaH          QTLYKQIKIQPENPRRLEILVDLPFGALLTS......D.LSF.ATWKKSSNP............AHI.....SDILR
1f_S._cerevisiae_HOS3              .............................LA......S.SMG....ISAAITMYPSLFTLKSSHQRKGSLMAPHVLK
1g_Homo_sapiens_HDAC4              .............................RG......K.CEC....IRGRK.............ATL.....EELQT
2a_Homo_sapiens_HDAC2              ............................LYR......K.MEI....YRPHK.............ATA.....EEMTK
2b_S._cerevisiae_HOS1              .............................LQ......HFDEV....LTFPY.............ARK.....DDLLE
2c_Bacillus_subtilis_AcuC          ............................AFD......D.GDI....VTPRL.............ASE.....EELSL
2d_Chromatiales_bacterium_Acu      ............................LDA......N.IEF....AAART.............ATR.....EELLA
3_Legionella_cherrii_ApaH          ............................NVA......L.TLFAE..FPEED.............QIL.....QALLA
4_Pseudomonas_sp_M30-35_ApaH       .............................RN......I.GEI....REPQD.............FGR.....DPIQR
5a_Acinetobacter_wuhouensis_H      ............................NVK......N.FEF....HEPKI.............LDP.....EILKA
5b_Vibrio_cholerae_HdaH            ............................WQA......S.FEF....HTPIA.............AEL.....SRITP
5c_Moraxella_bovis_HDAC            ............................IAT......P.EQF....FTPKM.............ASV.....NEILT
5d_Turneriella_parva_HdaH          .............................FS......R.WNW....NEPVA.............ATK.....EQLLL
5e_Nannocystis_exedens_AcuC        .............................VT......F.EQV....RRPPL.............ATY.....HAIRR
5f_Homo_sapiens_HDAC11             .............................LS......D.SML....VEARE.............ASE.....EDLLV
5g_Bacteriovorax_stolpii_AcuC      ............................FLE......Y.TDL....KTLPR.............ITR.....EDLER
```

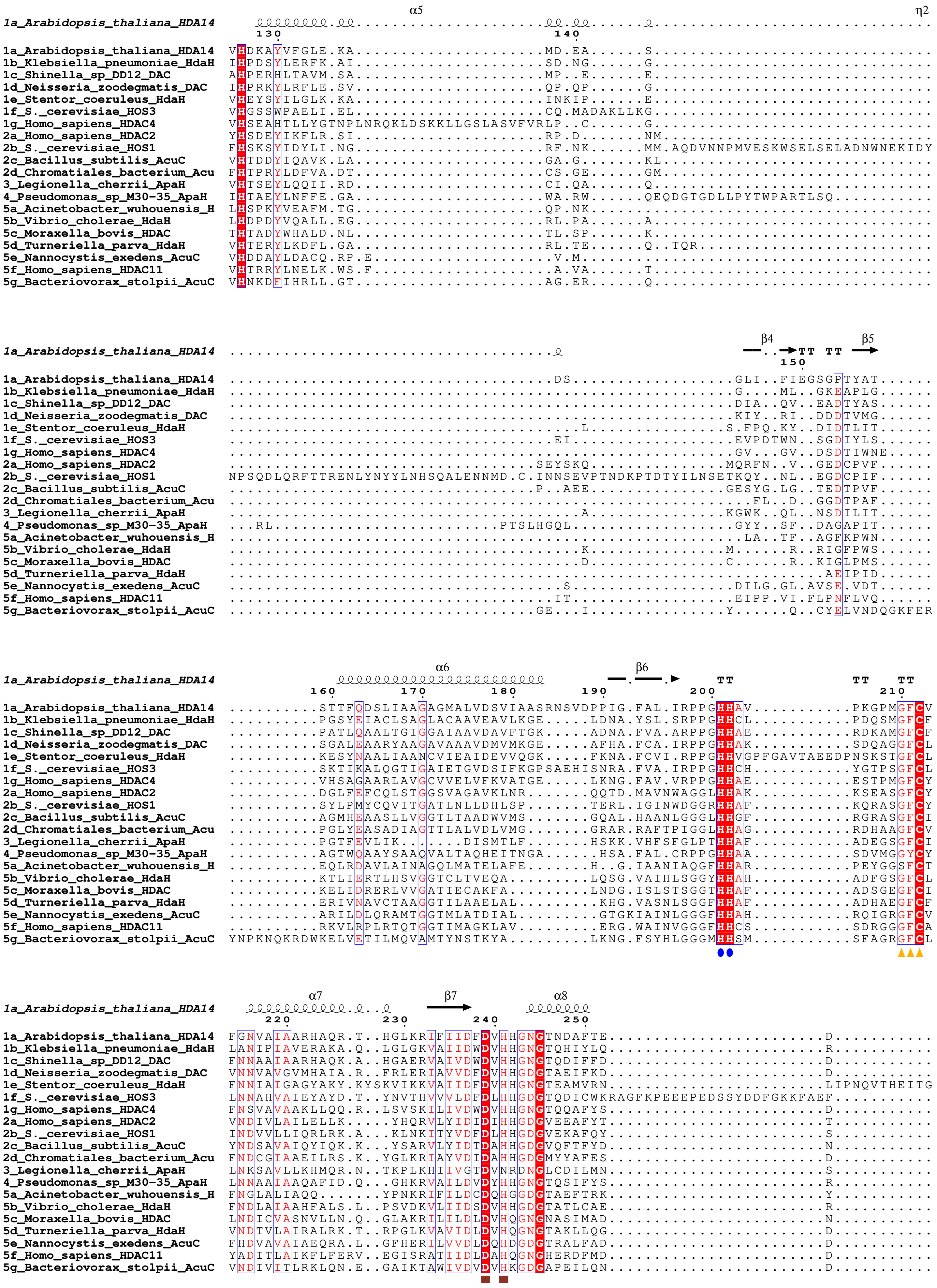
1a_Arabidopsis_thaliana_HDA14
α5
η2
β4
β5
α6
β6
α7
β7
α8
1a_Arabidopsis_thaliana_HDA14
1b_Klebsiella_pneumoniae_HdaH
1c_Shinella_sp_DD12_DAC
1d_Neisseria_zoodegmatis_DAC
1e_Stentor_coeruleus_HdaH
1f_S._cerevisiae_HOS3
1g_Homo_sapiens_HDAC4
2a_Homo_sapiens_HDAC2
2b_S._cerevisiae_HOS1
2c_Bacillus_subtilis_AcuC
2d_Chromatiales_bacterium_Acu
3_Legionella_cherrii_ApaH
4_Pseudomonas_sp_M30-35_ApaH
5a_Acinetobacter_wuhouensis_H
5b_Vibrio_cholerae_HdaH
5c_Moraxella_bovis_HDAC
5d_Turneriella_parva_HdaH
5e_Nannocystis_exedens_AcuC
5f_Homo_sapiens_HDAC11
5g_Bacteriovorax_stolpii_AcuC

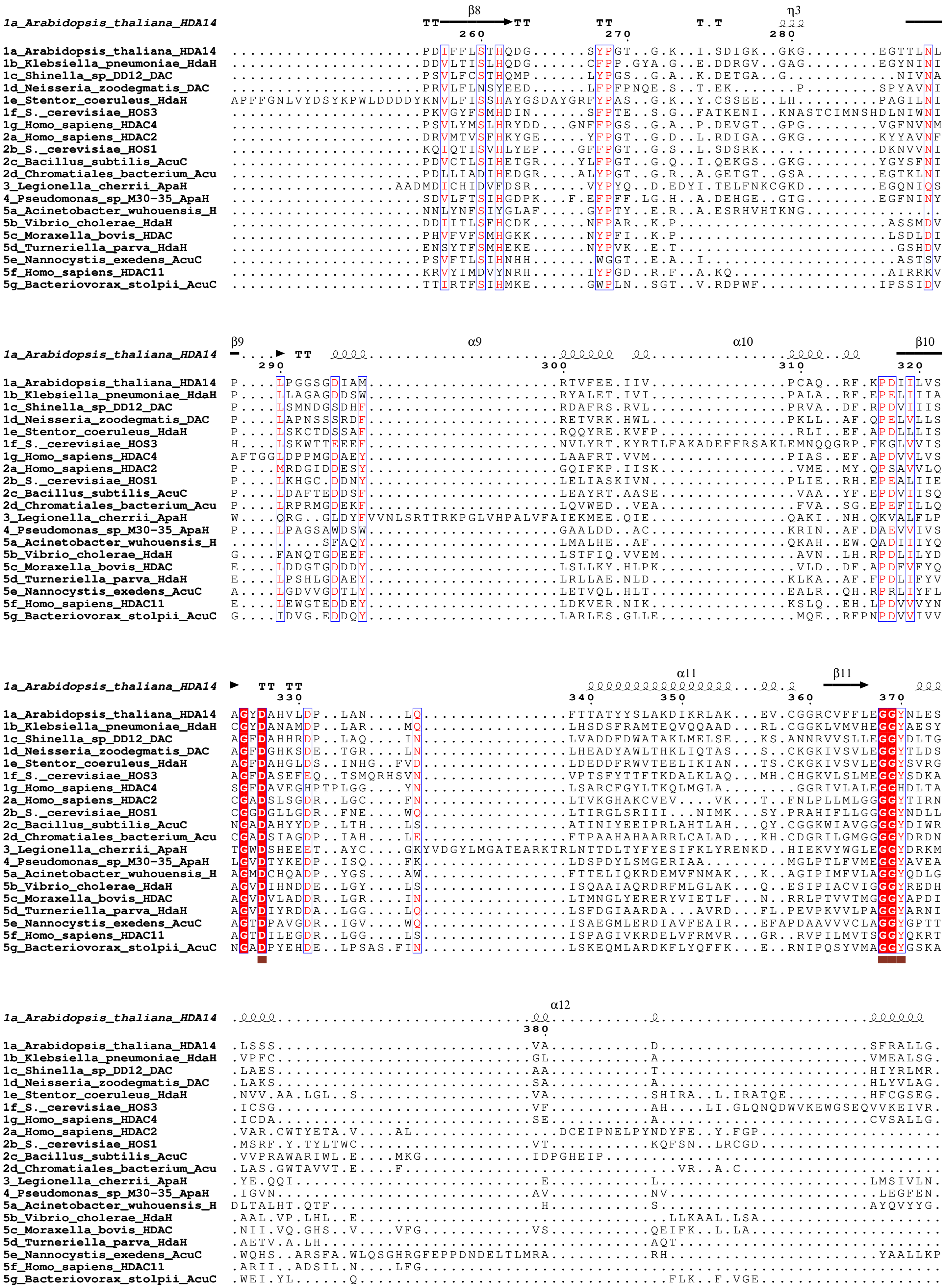

1a_Arabidopsis_thaliana_HDA14
β8 η3
TT TT TT T.T
260 270 280
1a_Arabidopsis_thaliana_HDA14 ....................PDIFFLSTHQDG......SYPGT..G.K..I.SDIGK..GKG.......EGTTLNL
1b_Klebsiella_pneumoniae_HdaH ....................DDVLTISLHQDG......CFPP.GYA.G..E.DDRGV..GAG.......EGYNINI
1c_Shinella_sp_DD12_DAC ....................PSVLFCSTHQMP......LYPGS..G.A..K.DETGA..G...........NIVNA
1d_Neisseria_zoodegmatis_DAC ....................PRVLFLNSYEED......LFPFPNQE.S..T.EK......P........SPYAVNI
1e_Stentor_coeruleus_HdaH APFFGNLVYDSYKPWLDDDDYKNVLFISSHAYGSDAYGRFYPAS..G.K..Y.CSSEE..LH........PAGILNI
1f_S._cerevisiae_HOS3 ....................PKVGYFSMHDIN......SFPTE..S.G..FATKENI..KNASTCIMNSHDLNIWNI
1g_Homo_sapiens_HDAC4 ....................PSVLYMSLHRYDD...GNFFPGS..G.A..P.DEVGT..GPG.......VGFNVNM
2a_Homo_sapiens_HDAC2 ....................DRVMTVSFHKYGE.....YFPGT..G.D..L.RDIGA..GKG.......KYYAVNF
2b_S._cerevisiae_HOS1 ....................KQIQTISVHLYEP.....GFFPGT..G.S..L.SDSRK...........DKNVVNI
2c_Bacillus_subtilis_AcuC ....................PDVCTLSIHETGR....YLFPGT..G.Q..I.QEKGS..GKG.......YGYSFNI
2d_Chromatiales_bacterium_Acu ....................PDLLIADIHEDGR....ALYPGT..G.R..A.GETGT..GSA.......EGTKLNI
3_Legionella_cherrii_ApaH .................AADMDICHIDVFDSR......VYPYQ..D.EDYI.TELFNKCGKD.......EGQNIQS
4_Pseudomonas_sp_M30-35_ApaH ....................SDVLFTSIHGDPK...F.EFPFF.LG.H..A.DEHGE..GTG.......EGFNINY
5a_Acinetobacter_wuhouensis_H ....................NNLYNFSIYGLAF.....GYPTY..E.R..A.ESRHVHTKNG..............
5b_Vibrio_cholerae_HdaH ....................DDIITLSFHCDK......NFPAR..K.P...................ASSMDV
5c_Moraxella_bovis_HDAC ....................PHVFVFSMHGKK......NYPFI..K.P...................LSDLDI
5d_Turneriella_parva_HdaH ....................ENSYTFSMHEKE......NYPVK..E.T...................GSHDV
5e_Nannocystis_exedens_AcuC ....................PSVFTLSIHNHH.......WGGT..E.A..I...................ASTSV
5f_Homo_sapiens_HDAC11 ....................KRVYIMDVYNRH......IYPGD..R.F..A.KQ...................AIRRKV
5g_Bacteriovorax_stolpii_AcuC ....................TTIRTFSIHMKE......GWPLN..SGT..V.RDPWF...............IPSSIDV
β9 α9 α10 β10
TT
290 300 310 320
1a_Arabidopsis_thaliana_HDA14 P....LPGGSGDIAM....................RTVFEE.IIV..............PCAQ..RF.KPDIILVS
1b_Klebsiella_pneumoniae_HdaH P....LLAGAGDDSW....................RYALET.IVI..............PALA..RF.EPELIIIA
1c_Shinella_sp_DD12_DAC P....LSMNDGSDHF....................RDAFRS.RVL..............PRVA..DF.RPDVIIIS
1d_Neisseria_zoodegmatis_DAC P....LAPNSSSRDF....................RETVRK.HWL..............PKLL..AF.QPELVLLS
1e_Stentor_coeruleus_HdaH P....LSKCTDSSAF....................RQQYRE.KVFP.................RLI..EF.APDLLLIS
1f_S._cerevisiae_HOS3 H....LSKWTTEEEF....................NVLYRT.KYRTLFAKADEFFRSAKLEMNQQGRP.FKGLVVIS
1g_Homo_sapiens_HDAC4 AFTGGLDPPMGDAEY....................LAAFRT.VVM..............PIAS..EF.APDVVLVS
2a_Homo_sapiens_HDAC2 P....MRDGIDDESY....................GQIFKP.IISK...............VME..MY.QPSAVVLQ
2b_S._cerevisiae_HOS1 P....LKHGC.DDNY....................LELIASKIVN..............PLIE..RH.EPEALIIE
2c_Bacillus_subtilis_AcuC P....LDAFTEDDSF....................LEAYRT.AASE...............VAA..YF.EPDVIISQ
2d_Chromatiales_bacterium_Acu P....LRPRMGDEKF....................LQVWED..VEA...............FVA..SG.EPEFILLQ
3_Legionella_cherrii_ApaH W....QRG..GLDYFVVNLSRTTRKPGLVHPALVFAIEKMEE.QIE..............QAKI..NH.QKVALFLP
4_Pseudomonas_sp_M30-35_ApaH P....LPAGSAWDSW....................GAALDD..AC...............KRIN..AF.DAEVVIVS
5a_Acinetobacter_wuhouensis_H ..........SFAQY....................LMALHE..AF...............QKAH..EW.QADIIIYQ
5b_Vibrio_cholerae_HdaH G....FANQTGDEEF....................LSTFIQ.VVEM...............AVN..LH.RPDLILYD
5c_Moraxella_bovis_HDAC E....LDDGTGDDDY....................LSLLKY.HLPK...............VLD..DF.APDFVFYQ
5d_Turneriella_parva_HdaH E....LPSHLGDAEY....................LRLLAE.NLD..............KLKA..AF.FPDLIFYV
5e_Nannocystis_exedens_AcuC A....LGDVVGDTLY....................LETVQL.HLT..............EALR..QH.RPRLIYFL
5f_Homo_sapiens_HDAC11 E....LEWGTEDDEY....................LDKVER.NIK..............KSLQ..EH.LPDVVVYN
5g_Bacteriovorax_stolpii_AcuC G....IDVG.EDDQY....................LARLES.GLLE..............MQE..RFPNPDVVIVV
α11 β11
TT TT
330 340 350 360 370
1a_Arabidopsis_thaliana_HDA14 AGYDAHVLDP..LAN....LQ..................FTTATYYSLAKDIKRLAK...EV.CGGRCVFFLEGGYNLES
1b_Klebsiella_pneumoniae_HdaH CGYDANAMDP..LAR....MQ..................LHSDSFRAMTEQVQQAAD...RL.CGGKLVMVHEGGYAESY
1c_Shinella_sp_DD12_DAC AGFDAHHRDP..LAQ....IN..................LVADDFDWATAKLMELSE...KS.ANNRVVSLLEGGYDLTG
1d_Neisseria_zoodegmatis_DAC AGFDGHKSDE..TGR....LN..................LHEADYAWLTHKLIQTAS...S..CKGKIVSVLEGGYTLDS
1e_Stentor_coeruleus_HdaH AGFDAHGLDS..INHG..FVD..................LDEDDFRWVTEELIKIAN...TS.CKGKIVSVLEGGYSVRG
1f_S._cerevisiae_HOS3 AGFDASEFEQ..TSMQRHSVN..................VPTSFYTTFTKDALKLAQ...MH.CHGKVLSLMEGGYSDKA
1g_Homo_sapiens_HDAC4 SGFDAVEGHPTPLGG....YN..................LSARCFGYLTKQLMGLA.......GGRIVLALEGGHDLTA
2a_Homo_sapiens_HDAC2 CGADSLSGDR..LGC....FN..................LTVKGHAKCVEV....VK...T..FNLPLLMLGGGGYTIRN
2b_S._cerevisiae_HOS1 CGGDGLLGDR..FNE....WQ..................LTIRGLSRIII...NIMK...SY.PRAHIFLLGGGGYNDLL
2c_Bacillus_subtilis_AcuC NGADAHYYDP..LTH....LS..................ATINIYEEIPRLAHTLAH...QY.CGGKWIAVGGGGYDIWR
2d_Chromatiales_bacterium_Acu CGADSIAGDP..IAH....LE..................FTPAAHAHAARRLCALAD...KH.CDGRILGMGGGGYDRDN
3_Legionella_cherrii_ApaH TGWDSHEEET..AYC....GKYVDGYLMGATEARKTRLNTTDLTYFYESIFKLYRENKD..HIEKVYWGLEGGYDRKM
4_Pseudomonas_sp_M30-35_ApaH LGVDTYKEDP..ISQ....FK..................LDSPDYLSMGERIAA........MGLPTLFVMEGGYAVEA
5a_Acinetobacter_wuhouensis_H AGMDCHQADP..YGS....AW..................FTTELIQKRDEMVFNMAK...K..AGIPIMFVLAGGYQDLG
5b_Vibrio_cholerae_HdaH AGVDIHNDDE..LGY....LS..................ISQAAIAQRDRFMLGLAK...Q..ESIPIACVIGGGYREDH
5c_Moraxella_bovis_HDAC AGVDVLADDR..LGR....IN..................LTMNGLYERERYVIETLF...N..RRLPTVVTMGGGYAPDI
5d_Turneriella_parva_HdaH AGVDIYRDDA..LGG....LQ..................LSFDGIAARDA...AVRD...FL.PEVPKVVLPAGGYARNI
5e_Nannocystis_exedens_AcuC AGTDPAVGDR..IGV....WQ..................ISAEGMLERDIAVFEAIR...EFAPDAAVVVCLAGGYGPTT
5f_Homo_sapiens_HDAC11 AGTDILEGDR..LGG....LS..................ISPAGIVKRDELVFRMVR...GR..RVPILMVTSGGYQKRT
5g_Bacteriovorax_stolpii_AcuC NGADPYEHDE..LPSAS.FIN..................LSKEQMLARDKFLYQFFK...E..RNIPQSYVMAGGYGSKA
α12
380
1a_Arabidopsis_thaliana_HDA14 .LSSS.........................VA..........D......................SFRALLG.
1b_Klebsiella_pneumoniae_HdaH .VPFC.........................GL..........A......................VMEALSG.
1c_Shinella_sp_DD12_DAC .LAES.........................AA..........T......................HIYRLMR.
1d_Neisseria_zoodegmatis_DAC .LAKS.........................SA..........A......................HLYVLAG.
1e_Stentor_coeruleus_HdaH .NVV.AA.LGL..S................VA..........SHIRA..L.IRATQE........HFCGSEG.
1f_S._cerevisiae_HOS3 .ICSG.........................VF..........AH....LI.GLQNQDWVKEWGSEQVVKEIVR.
1g_Homo_sapiens_HDAC4 .ICDA.........................SE..........A......................CVSALLG.
2a_Homo_sapiens_HDAC2 .VAR.CWTYETA.V....AL..........DCEIPNELPYNDYFE..Y.FGP......................
2b_S._cerevisiae_HOS1 .MSRF.Y.TYLTWC................VT..........KQFSN..LRCGD.....................
2c_Bacillus_subtilis_AcuC .VVPRAWARIWL.E....MKG.........IDPGHEIP....................................
2d_Chromatiales_bacterium_Acu .LAS.GWTAVVT.E....F.......................VR..A.C..........................
3_Legionella_cherrii_ApaH .YE.QQI.......................E...........L......................LMSIVLN.
4_Pseudomonas_sp_M30-35_ApaH .IGVN.........................AV..........NV......................LEGFEN.
5a_Acinetobacter_wuhouensis_H DLTALHT.QTF...................H...........S......................AYQVYYG.
5b_Vibrio_cholerae_HdaH .AAL.VP.LHL..E..............................LLKAAL.LSA.....................
5c_Moraxella_bovis_HDAC .NII.VQ.GHS..V....VFG.........VS..........QEIFK..L.LA.....................
5d_Turneriella_parva_HdaH .AETV.A.LH....................AQT...
5e_Nannocystis_exedens_AcuC .WQHS..ARSFA.WLQSGHRGFEPPDNDELTLMRA.......RH......................YAALLKP
5f_Homo_sapiens_HDAC11 .ARII..ADSIL.N....LFG...
5g_Bacteriovorax_stolpii_AcuC .WEI.YL......Q............................FLK..F.VGE.......................

```
1a_Arabidopsis_thaliana_HDA14
                                   390
1a_Arabidopsis_thaliana_HDA14  .....ED.......................................................................
1b_Klebsiella_pneumoniae_HdaH  .....IR.......................................................................
1c_Shinella_sp_DD12_DAC        .....G........................................................................
1d_Neisseria_zoodegmatis_DAC   .....LG.......................................................................
1e_Stentor_coeruleus_HdaH      .....ND.......................................................................
1f_S._cerevisiae_HOS3          .....GC..................................................KPAWKPYKTKRAKDVIR....
1g_Homo_sapiens_HDAC4          .....NE.......................................................................
2a_Homo_sapiens_HDAC2          ..............................................................................
2b_S._cerevisiae_HOS1          ..............................................................................
2c_Bacillus_subtilis_AcuC      ..............................................................................
2d_Chromatiales_bacterium_Acu  ..............................................................................
3_Legionella_cherrii_ApaH      .....DL.......................................................................
4_Pseudomonas_sp_M30-35_ApaH   .....VC.......................................................................
5a_Acinetobacter_wuhouensis_H  ..............................................................................
5b_Vibrio_cholerae_HdaH        ..............................................................................
5c_Moraxella_bovis_HDAC        ..............................................................................
5d_Turneriella_parva_HdaH      ..............................................................................
5e_Nannocystis_exedens_AcuC    AELTGEDDDLGLTLEDLVPSLAATAREPRFLGYYSRQGLELALERHGFLGRLRGKGFQPSLEFNLVDPQRHILKIFGD
5f_Homo_sapiens_HDAC11         .....LG...................................................................LI.G
5g_Bacteriovorax_stolpii_AcuC  ..............................................................................
```

```
                                                                                   η4                    η5
1a_Arabidopsis_thaliana_HDA14  ...............................................ℓ.......ℓℓ...ℓ.................

1a_Arabidopsis_thaliana_HDA14  .............................................SLA.......SEFDNP.................
1b_Klebsiella_pneumoniae_HdaH  ...............................................T.......EVQ.DPLL...............
1c_Shinella_sp_DD12_DAC        ..............................................................................
1d_Neisseria_zoodegmatis_DAC   ..............................................................................
1e_Stentor_coeruleus_HdaH      .NEKMILKSS....................VNESFRALKRNRKMYIE...............................
1f_S._cerevisiae_HOS3          .....IWAEEVIRLGRAMIPEFDDIIFKDAVN.............SAPSN.SLLKATV.EPAS...............
1g_Homo_sapiens_HDAC4          .........................................................L.DPLP...............
2a_Homo_sapiens_HDAC2          ........................................DFKLHISPSN.MTNQN...TPEY...............
2b_S._cerevisiae_HOS1          ........................................NNSFQID.............P.................
2c_Bacillus_subtilis_AcuC      ............................................................PEW...............
2d_Chromatiales_bacterium_Acu  ..............................................................................
3_Legionella_cherrii_ApaH      ...........................................VHQD...............................
4_Pseudomonas_sp_M30-35_ApaH   ..............................................................................
5a_Acinetobacter_wuhouensis_H  ..............................................................................
5b_Vibrio_cholerae_HdaH        ..............................................................................
5c_Moraxella_bovis_HDAC        ..............................................................................
5d_Turneriella_parva_HdaH      ..............................................................................
5e_Nannocystis_exedens_AcuC    PEHKELLFELVVRRDRRTIPGFELLAIDWLLL.............QNPRE.HFSED...KPALPGQRHPGLGLFEETV
5f_Homo_sapiens_HDAC11         PE............................................SPSVSAQNSD...TPL................
5g_Bacteriovorax_stolpii_AcuC  ........................................NAPFHLK...............................
```

```
1a_Arabidopsis_thaliana_HDA14  ....ℓℓ......................................................ℓℓℓℓℓℓ............
                                   400                                                  410
1a_Arabidopsis_thaliana_HDA14  ....AY....................L.............................YDEPMRKVRD............
1b_Klebsiella_pneumoniae_HdaH  ....EFI...................Q.............................QQQPRATFAQ............
1c_Shinella_sp_DD12_DAC        ..............................................................................
1d_Neisseria_zoodegmatis_DAC   ..........................K.............................PE....................
1e_Stentor_coeruleus_HdaH      .................................N.......LEVS.................ME.............N
1f_S._cerevisiae_HOS3          ....TST...................IAQRIIR...SHRS......N.ASPEKELHENKPRSTEKQ............
1g_Homo_sapiens_HDAC4          ....EKV...................L.............................QQRPNANAVR............
2a_Homo_sapiens_HDAC2          ....MEK...................IKQRLFENLRMLPHAPGVQMQ.AIPEDAVHEDSGDEDGEDPDKRISIRASDK
2b_S._cerevisiae_HOS1          ....................................................F.....D...................
2c_Bacillus_subtilis_AcuC      ....IVK...................WQKQ................................................
2d_Chromatiales_bacterium_Acu  ..............................................................................
3_Legionella_cherrii_ApaH      ..........................................................T...................
4_Pseudomonas_sp_M30-35_ApaH   ..........................................................RPNNSKKD............
5a_Acinetobacter_wuhouensis_H  ..............................................................................
5b_Vibrio_cholerae_HdaH        ..............................................................................
5c_Moraxella_bovis_HDAC        ..............................................................................
5d_Turneriella_parva_HdaH      ..............................................................................
5e_Nannocystis_exedens_AcuC    ALLIIACERVGLAGLVVTPSQYHLAVQWQKRLR...FVDP......RAGARMRAIREALPGASLTE............
5f_Homo_sapiens_HDAC11         ......................................................LPP.....................
5g_Bacteriovorax_stolpii_AcuC  ..............................................................................
```

```
                                                                 α13
1a_Arabidopsis_thaliana_HDA14  .ℓℓℓ......ℓℓℓℓ................................................................

1a_Arabidopsis_thaliana_HDA14  .AIQ......RAKS................................................................
1b_Klebsiella_pneumoniae_HdaH  .FQR......QAID................................................................
1c_Shinella_sp_DD12_DAC        ..............................................................................
1d_Neisseria_zoodegmatis_DAC   ...........CAV................................................................
1e_Stentor_coeruleus_HdaH      QLTAEV.......................AEEF.NGEKTQEVVSEESGGDEDQDEN.SEDESG.....NESE.....E
1f_S._cerevisiae_HOS3          .EQREIRSDTKVKQLSSNNRAAETQIPFLQQE..............F.SSEDEDEEYVYDEELNKTFNRTVEDITIDD
1g_Homo_sapiens_HDAC4          .SME......KVME................................................................
2a_Homo_sapiens_HDAC2          RI..AC.......................DEEFSDS..............EDEG.....EGGR....RNVA......D
2b_S._cerevisiae_HOS1          ..............................................V.CDGDDSEQFIREHDLVEMYNE.........
2c_Bacillus_subtilis_AcuC      .....C...............................................................PVA.....L
2d_Chromatiales_bacterium_Acu  ..............................................................................
3_Legionella_cherrii_ApaH      ..............................................................................
4_Pseudomonas_sp_M30-35_ApaH   .A............................................................................
5a_Acinetobacter_wuhouensis_H  ..............................................................................
5b_Vibrio_cholerae_HdaH        ..............................................................................
5c_Moraxella_bovis_HDAC        ..............................................................................
5d_Turneriella_parva_HdaH      ..............................................................................
5e_Nannocystis_exedens_AcuC    .AARALQEGRVVDQSG..............................R.TCSYEPSPM....................I
5f_Homo_sapiens_HDAC11         ..............................................................................
5g_Bacteriovorax_stolpii_AcuC  ..............................................................................
```

```
1a_Arabidopsis_thaliana_HDA14      ..ℓ.....ℓ
                                   420
                                     .
1a_Arabidopsis_thaliana_HDA14      ..I.....H............................C........................................
1b_Klebsiella_pneumoniae_HdaH      ..RLAQQFG............................LL.......................................
1c_Shinella_sp_DD12_DAC            ..............................................................................
1d_Neisseria_zoodegmatis_DAC       ..IYDKFLK............................SEH......................................
1e_Stentor_coeruleus_HdaH          GEEGEEREDNEEDDEE......ESEEEDSQESKKTDKANN.......GE....................PED..N...
1f_S._cerevisiae_HOS3              ISRHLETLE..IEKKGDEDSDHELKEKNWK.......NSHQRRLQGNGMYKIPSNTKPHRIRQPQNANTPTYDDSDIS
1g_Homo_sapiens_HDAC4              ..IHSKYWR............................CLQRTT.STAGR.........SLIEAQTC......ENEEAE
2a_Homo_sapiens_HDAC2              HKKGAKKARIEEDKKETEDKKTDVKEEDK......SKDNS.......GE....................KTD..T...
2b_S._cerevisiae_HOS1              ..ENYQYWI...........................YEME.......GS.........SRMKML..............
2c_Bacillus_subtilis_AcuC          PSSWSDPADLYPP....IPRKPEITEKNAQTVSKALYAIR.......SE.........................Q...
2d_Chromatiales_bacterium_Acu      ..............................................................................
3_Legionella_cherrii_ApaH          .............................HQM...............GK.............................
4_Pseudomonas_sp_M30-35_ApaH       ..............................................................................
5a_Acinetobacter_wuhouensis_H      ..............................................................................
5b_Vibrio_cholerae_HdaH            ...............................................GY.............................
5c_Moraxella_bovis_HDAC            ..............................................................................
5d_Turneriella_parva_HdaH          ..............................................................................
5e_Nannocystis_exedens_AcuC        FPISDE.LK............................AEL..................................D...
5f_Homo_sapiens_HDAC11             ..............................................................................
5g_Bacteriovorax_stolpii_AcuC      ..............................................................................


1a_Arabidopsis_thaliana_HDA14

1a_Arabidopsis_thaliana_HDA14      ....................................L
1b_Klebsiella_pneumoniae_HdaH      .....................................
1c_Shinella_sp_DD12_DAC            .....................................
1d_Neisseria_zoodegmatis_DAC       ....................................A
1e_Stentor_coeruleus_HdaH          .....................................
1f_S._cerevisiae_HOS3              MISHVSRKHTTRSGG.RW...................
1g_Homo_sapiens_HDAC4              TVTAMASL....SVG.VKPAEKRPDE.EPMEEEPP.L
2a_Homo_sapiens_HDAC2              .....................KGT...KSEQLSN..P
2b_S._cerevisiae_HOS1              .................RNDNKDRDMVELMKFYEL..
2c_Bacillus_subtilis_AcuC          .....................QR..........T..K
2d_Chromatiales_bacterium_Acu      .....................................
3_Legionella_cherrii_ApaH          ...............................TSSP..
4_Pseudomonas_sp_M30-35_ApaH       .....................................
5a_Acinetobacter_wuhouensis_H      ..................................E..
5b_Vibrio_cholerae_HdaH            .....................................
5c_Moraxella_bovis_HDAC            .....................................
5d_Turneriella_parva_HdaH          ..........................IKIMH...GRQ
5e_Nannocystis_exedens_AcuC        ............PENYLRAIDEAADE.YRFQLSSP.R
5f_Homo_sapiens_HDAC11             ................................AVP..
5g_Bacteriovorax_stolpii_AcuC      .....................................
```

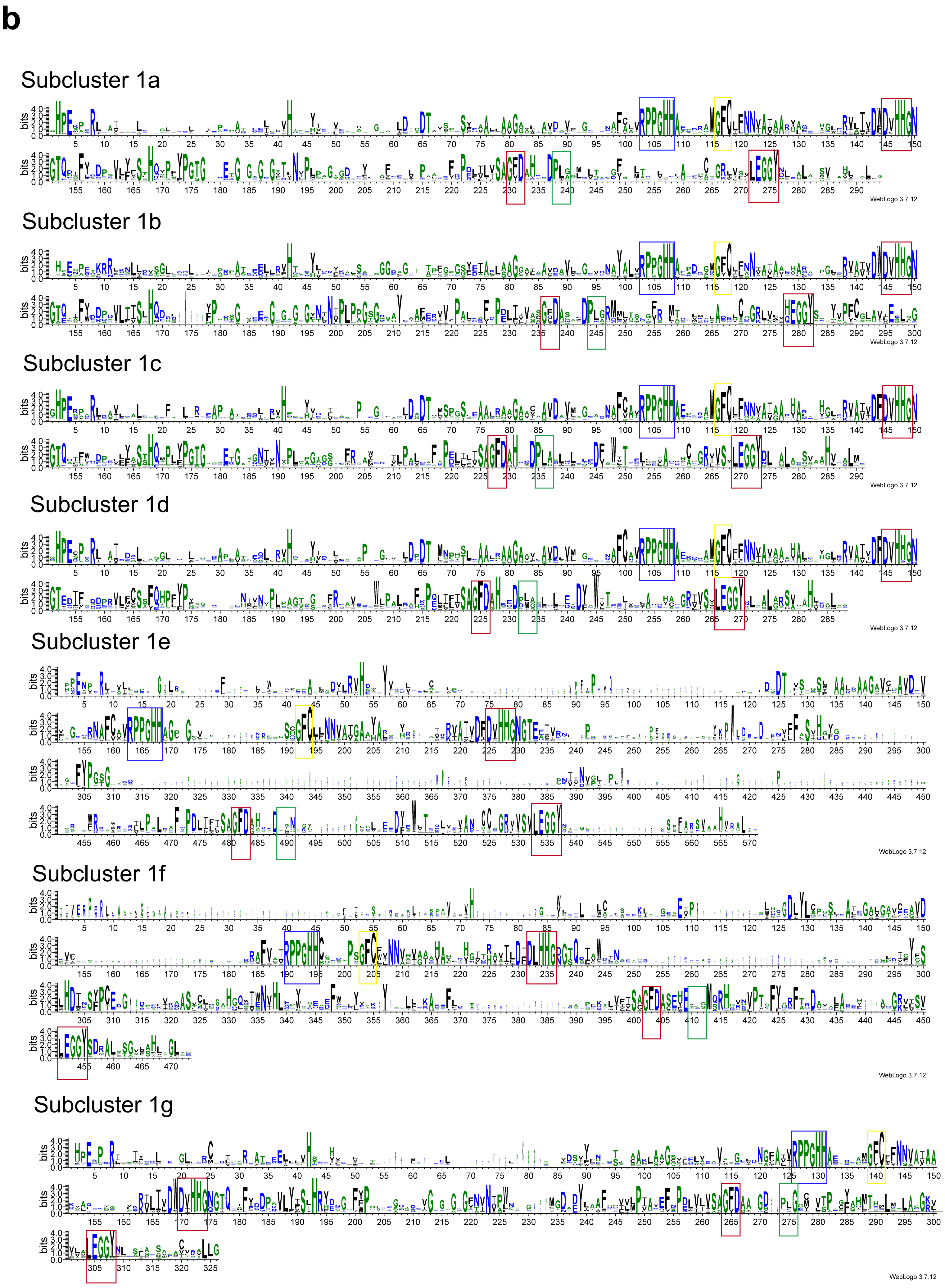
b
Subcluster 1a
Subcluster 1b
Subcluster 1c
Subcluster 1d
Subcluster 1e
Subcluster 1f
Subcluster 1g
WebLogo 3.7.12

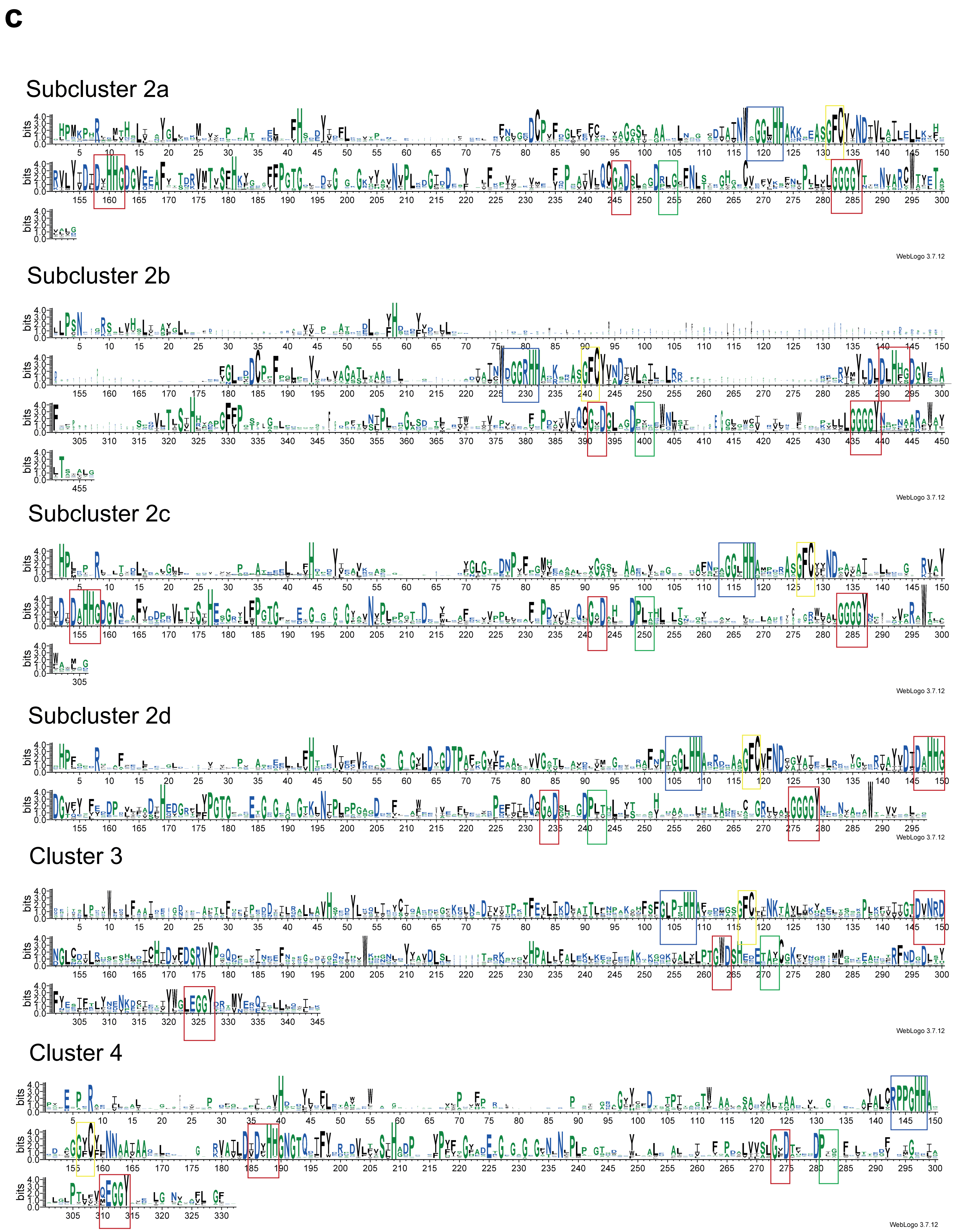
c
Subcluster 2a
Subcluster 2b
Subcluster 2c
Subcluster 2d
Cluster 3
Cluster 4
bits
WebLogo 3.7.12

d

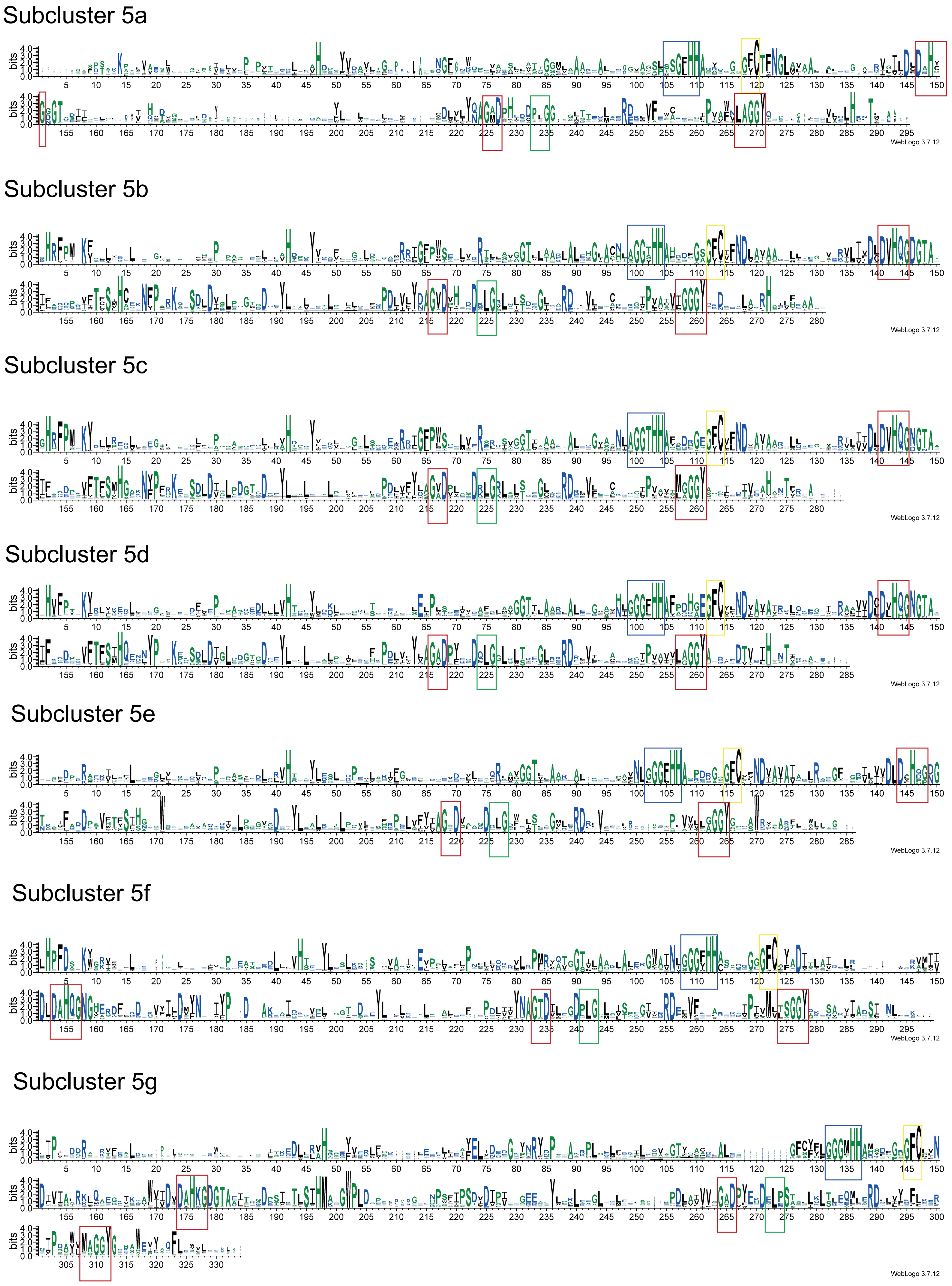
Subcluster 5a
Subcluster 5b
Subcluster 5c
Subcluster 5d
Subcluster 5e
Subcluster 5f
Subcluster 5g
bits
WebLogo 3.7.12

Supplementary Figure 1: Primary sequence alignment and sequence logo representations of selected deacylases representing all clusters 1-5 and the sub-clusters.

a Show are the following sequences of the following deacylases representing the five clusters and the sub-clusters (code: sub-cluster, accession number/protein name/spcies): 1a, AT4G33470.1/HDA14/*Arabidopsis thaliana*; 1b, A0A378F8Q8/HdaH/*Klebsiella pneumoniae*; 1c, A0A021X4K7/Deacetylase (DAC)/*Shinella sp.* DD12;1d, A0A1X3CVE4/Deacetylase (DAC)/*Neisseria zoodegmatis*; 1e, A0A1R2CWG0/HdaH/*Stentor coeruleus*; 1f, Q02959/HOS3/Saccharomyces cerevisiae (strain ATCC 204508 / S288c); 1g, P56524/HDAC4/*Homo sapiens*; 2a, Q92769/HDAC2/*Homo sapiens*; 2b, Q12214/HOS1/*Saccharomyces cerevisiae* (strain ATCC 204508 / S288c); 2c,P39067/AcuC/*Bacillus subtilis* (strain 168); 2d, A0A2E9D287/AcuC/*Chromatiales bacterium*; 3, A0A0W0SGS1/ApaH/*Legionella cherrii*; 4, A0A1Y0KY79/ApaH/*Pseudomonas sp.* M30-35; 5a, A0A385C7X8/HDAC/*Acinetobacter wuhouensis*; 5b, A0A395TF31/HDAC/*Vibrio cholerae*; 5c, A0A2Z4RAM2/HDAC/*Moraxella bovis*; 5d, I4B797HdaH/*Turneriella parva* (strain ATCC BAA-1111 / DSM 21527 / NCTC 11395 / H) (*Leptospira parva*); 5e, A0A1I2D6R7/AcuC/*Nannocystis exedens*; 5f, Q96DB2/HDAC11/*Homo sapiens*; 5g, A0A2K9NTX8/AcuC/*Bacteriovorax stolpii* (*Bdellovibrio stolpii*). All sequences were aligned using the T-COFFEE multiple sequence alignment server[1]. The important catalytic residues are highlighted. The secondary structure elements and numbering is shown for the deacylase shown at the top of the amino acid sequences above the sequence alignment. The alignment was created by ESPript version 3.0[2].

b Logo representation of enzymes of cluster 1. For each subcluster a sequence logo representation is shown. The important residues are highlighted. Blue box: double-His motif with the second His acting as catalytic base/acid (*Kp*HdaH: H143-H144); yellow box: conserved GFC-motif lining the substrate binding channel; brown boxes: Asp-His-Asp for coordination of the catalytic Zn^{2+}-ion and the XGGY-motif lining the foot pocket and the catalytic Tyr (*Kp*HdaH: Y313); green box: RPP-motif lining the foot pocket.

c Logo representation of enzymes of cluster 2, 3 and 4. For each cluster 2 a sequence logo representation is shown for each subcluster. The important residues are highlighted. Colour coding as described in b.

d Logo representation of enzymes of cluster 5. For each subcluster a sequence logo representation is shown. The important residues are highlighted. Colour coding as described in b.

a

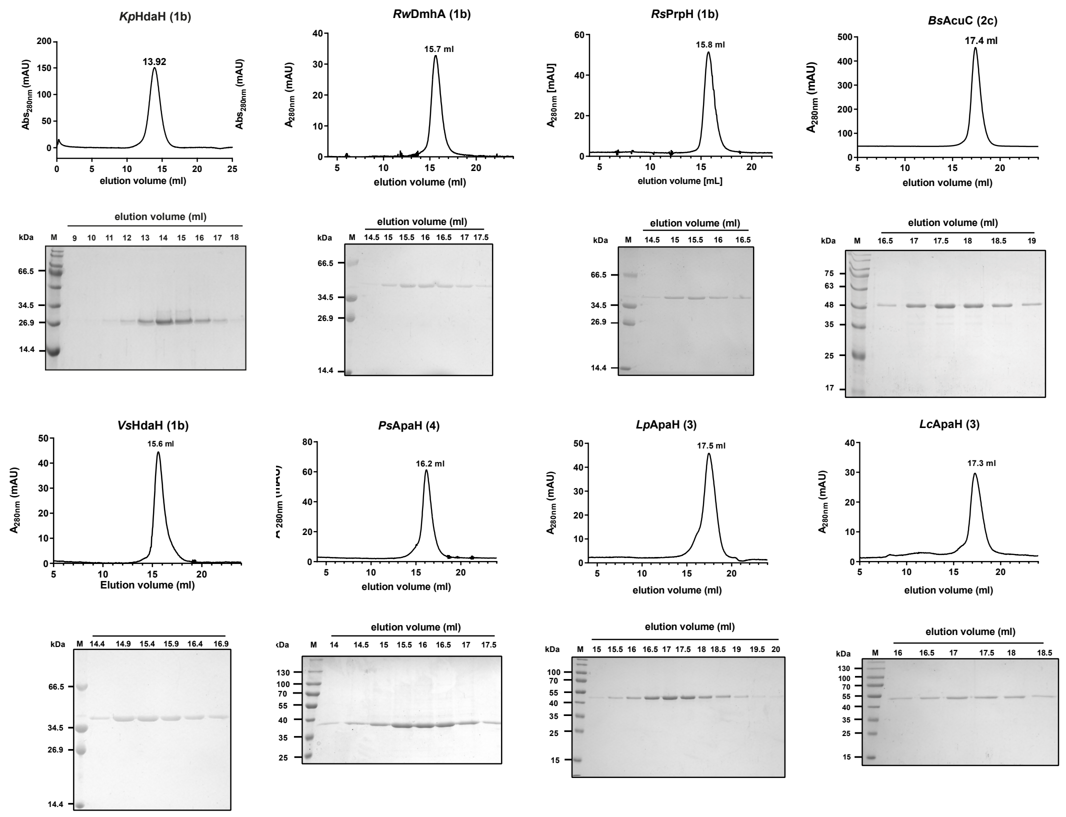

b

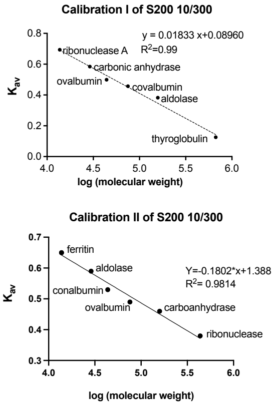

protein	exp. MW (Da)	elution volume (ml)	calc. MW (Da)	oligomeric state (calc. MW/exp. MW)
*Kp*HdaH (1b)	41569	13.9	136432	3.3
*Vs*HdaH (1b)	41435	15.6	157562	3.8
*Rs*PrpH (1b)	41227	15.8	133166	3.2
*Rw*DmhA (1b)	40563	15.7	146074	3.6
*Bs*AcuC (2c)	44273	17.4	34377	0.8
*Lp*ApaH (3)	49947	17.5	37868	0.8
*Lc*ApaH (3)	48902	17.3	32140	0.7
*Ps*ApaH (4)	39914	16.2	95121	2.4
*Vc*HdaH (5b)	35245	17.1	42780	1.2

Supplementary Figure 2: Purification and analytical size-exclusion chromatography (SEC) of all purified bacterial deacylases.

a Analytical size-exclusion chromatography (SEC) of all bacterial deacylases. 300 µg of bacterial deacylases were analysed on a Superdex 200 Increase 10/300 GL column. The elution volumes and calculated molecular weights in comparison to expected molecular weights are used to calculate an oligomeric state. The calculated molecular weights were obtained based on the elution volume using a calibration curve. Below the SEC chromatograms, the SDS-PAGE gels were shown analysing fractions of the observed absorption peak at 280 nm (A_{280nm}). SDS-PAGE was stained using Coomassie Brilliant Blue (CBB). Source data are provided as Source Data file.

b The elution volumes and calculated molecular weights (calc. MW) in comparison to expected molecular weights (exp. MW) are used to calculate the apparent oligomeric states of the enzymes. The calculated molecular weights were obtained based on the elution volume using a calibration curve. Calibration curve I was used for *Kp*HdaH (1b) and the mutants thereof and calibration curve II for the other enzymes. Source data are provided as Source Data file.

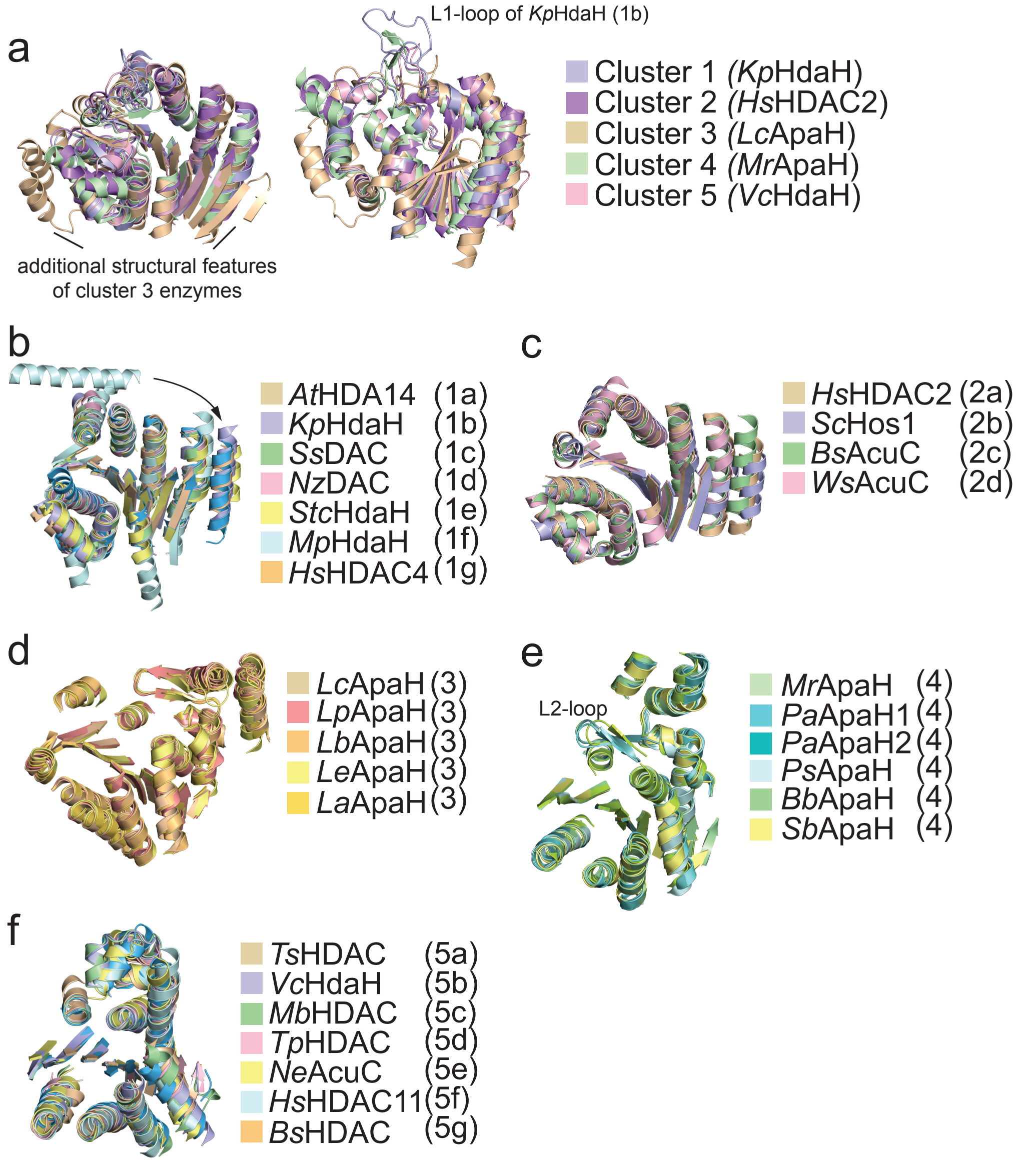

Supplementary Fig. 3: Structural alignments of core domains of deacylases reveal high structural similarity of enzymes within each main cluster and between the clusters.

a AlphaFold2 structure predictions were performed for selected enzymes representing the sub-clusters. a Superposition of representative enzymes representing the five main clusters (cluster 1-5). All structures show r.m.s.d. values close to 1 Å or lower to the reference structure (Supplementary Data 4). L1-loop of *Kp*HdaH (1b) and the additional structural features of cluster 3 enzymes, i.e. the extended β-sheet and the additional α-helices, enzymes are highlighted.

b Superposition of representative enzymes of cluster 1 (sub-clusters 1a-1g). All structures show r.m.s.d. values close to 1 Å or lower to the reference structure (Supplementary Data 4). We observed two exceptions: Firstly, the eukaryotic enzyme HdaH of *Malassezia pachydermatis* (1f) compared to the bacterial *Kp*HdaH (1b) with an r.m.s.d value of 7 Å in the comparison of cluster 1 enzymes. *M. pachydermatis* (1f) shows a reorganization of the catalytic core domain, i.e. the a-helix usually lining the central β-sheet is displaced by approximatly 90°. Moreover, thos enzyme shows an extended α-helical N-terminal domain preceding the catalytic domain.

c Superposition of representative enzymes of cluster 2 (sub-clusters 2a-2d). All structures show r.m.s.d. values close to 1 Å or lower to the reference structure (Supplementary Data 4).

d Superposition of representative enzymes of cluster 3. All structures show r.m.s.d. values close to 1 Å or lower to the reference structure (Supplementary Data 4). The cluster 3 enzyme of *L. cherrii* shows an r.m.s.d to the structure of *Kp*HdaH (1b) of 5.5 Å in the comparison of enzymes representing the main-clusters. The structural dissimilarity of the cluster 3 enzymes is due to the additional structural features, i.e. the extended β-sheet and the additional α-helices compared in the catalytic core.

e Superposition of representative enzymes of cluster 4. All structures show r.m.s.d. values close to 1 Å or lower to the reference structure (Supplementary Data 4).

f Superposition of representative enzymes of cluster 5 (sub-clusters 5a-5g). All structures show r.m.s.d. values close to 1 Å or lower to the reference structure (Supplementary Data 4).

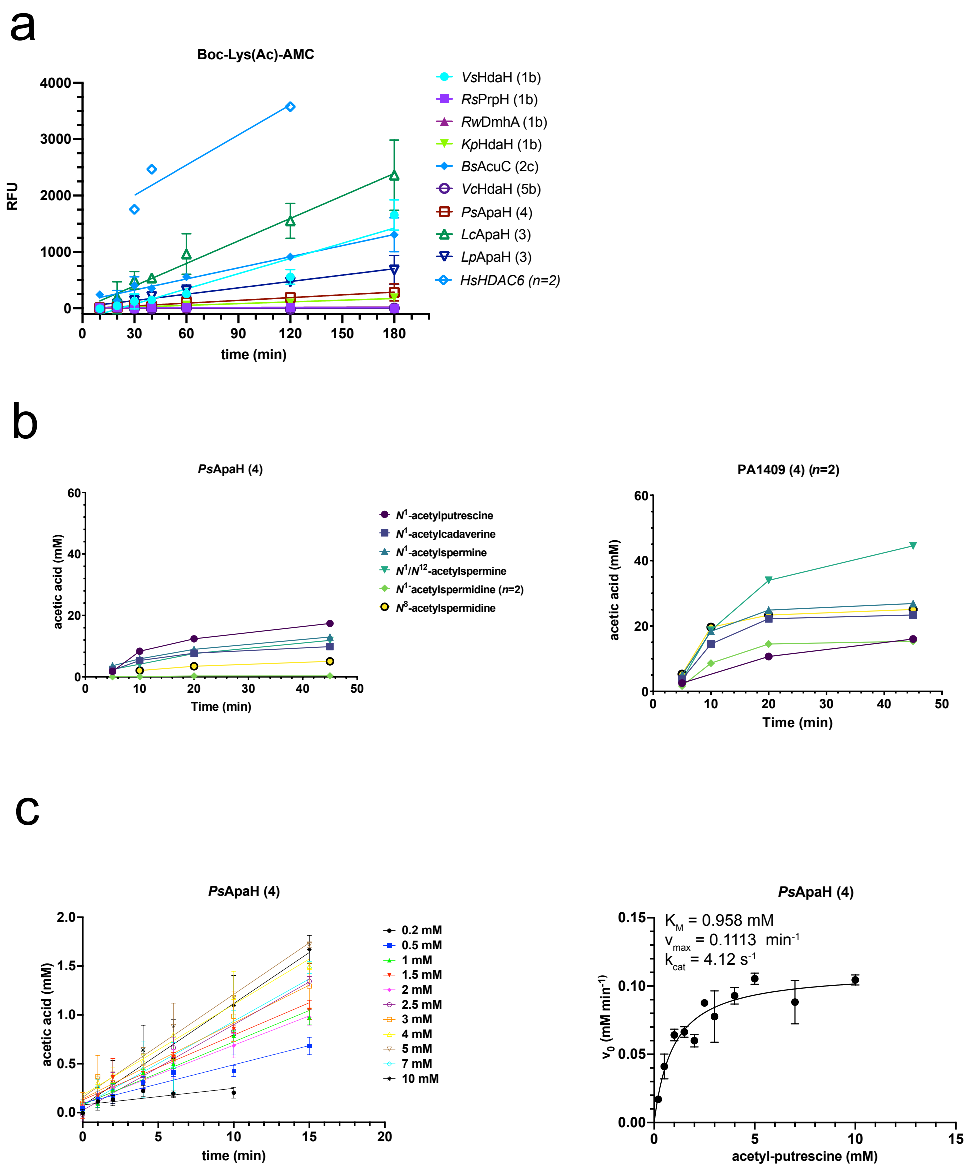

Supplementary Figure 4: Determination of the dynamic ranges for the pre-screening assays.

a Determination of the dynamic range for the pre-screening of the bacterial deacylases to use Boc-Lys(Ac)-AMC as a substrate. 20 nM enzyme and 20 µM Boc-Lys(Ac)-AMC substrate was used and the fluorescence of the released AMC-fluorophore was measured. An assay-incubation time of 30 min was selected as enyzmes show a linear correlation of the fluorescence signal and assay incubation time. The experiments were conducted in three biologically independent replicates (*n*=3) except for *Hs*HDAC6 (1g) (*n*=2). Shown are means±SD. For *Hs*HDAC6 (1g) means are shown. Source data are provided as Source Data file.

b Determination of the dynamic range for the screening of *Ps*ApaH (4) on its capacity to deacetylate acetylated polyamines as substrates. 200 nM *Ps*ApaH (4) (left panel) and 100 nM PA1409 enzyme (right panel) and 3 mM acetylated polyamines as indicated. Acetylated polyamines were used as substrates and the released acetate was detected by a coupled enzymatic assay by formation of NADH+H^+ from NAD^+ (K-ACET, Megazyme). An assay-incubation time of 20 min was selected as enyzmes show a linear correlation of the absorption at 340 nM and assay incubation time. The release of acetate was normalized to the enzyme concentration used. The experiments were conducted in three or two biologically independent replicates (*n*=3 and *n*=2) for *Ps*ApaH (4) and PA1409, respectively. Shown are means±SD for *Ps*ApaH (4) and means for PA1409. Source data are provided as Source Data file.

c Michaelis-Menten kinetics for the acetyl-putrescine deacetylation by *Ps*ApaH (4). Initial reaction rates were (v_0) determined using 200 nM *Ps*ApaH (4) and increasing concentrations of acetyl-putrescine as indicated and measuring the released acetate. Linearity was present up to 15 min assay time. The reaction rates (v_0) were plotted as a funtion of the acetylputrescine concentration resulting in a hyperbolic curve that was fitted by a Michelis-Menten equation resulting K_M, v_{max} and k_{cat}. The experiments were conducted in three biologically independent replicates (*n*=3). Shown are means±SD. Source data are provided as Source Data file.

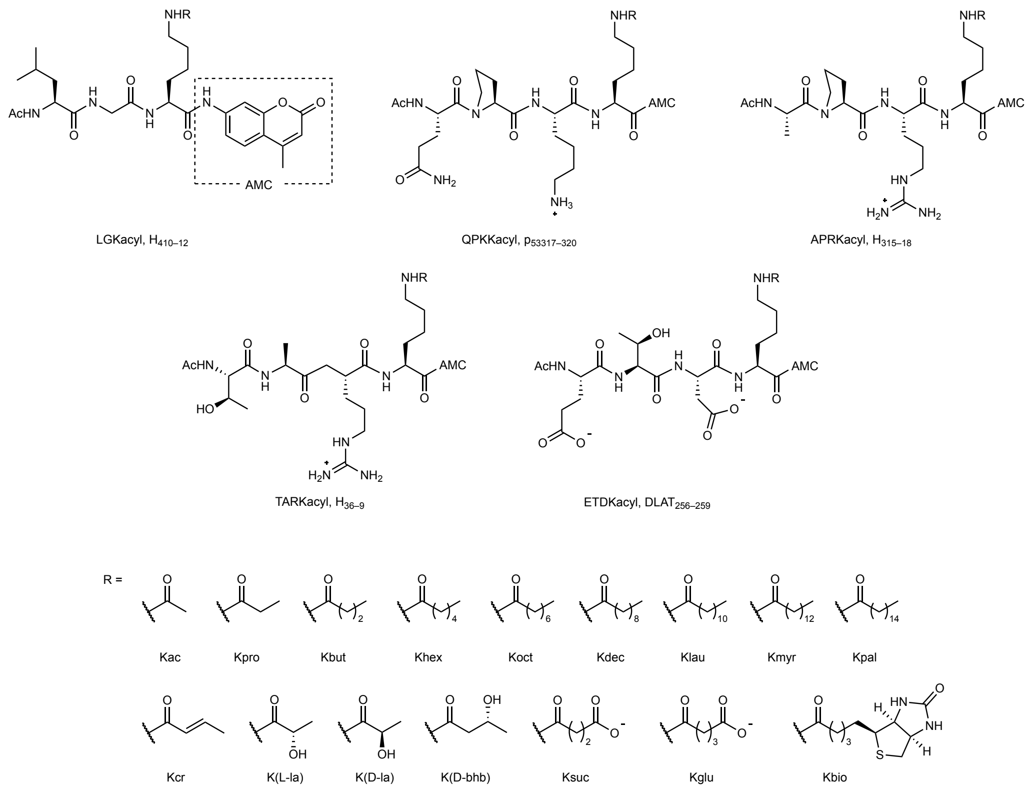

Supplementary Figure 5: Chemical structures of peptides and acyl-chain types used as substrates for deacylation by selected bacterial deacylases.

a. Peptides with varying sequences used to study substrate preference for various bacterial deacylases. The peptides suitable to detect a successful lysine deacylation by release of an C-terminally attached AMC (7-amino-4-methylcoumarin) fluorophore upon trypsin cleavage (Fluor-deLys-assay). The AMC fluorophore is quenched in the peptide but is fluorescent in its free form. The peptides used were derived from histone H3 (APRK$_{acyl}$, H3$_{15\text{-}18}$ or TARK$_{acyl}$, H3$_{6\text{-}9}$), histone H4 (LGK$_{acyl}$, H4$_{10\text{-}12}$) tumor suppressor protein p53 (QPKK$_{acyl}$, p53$_{317\text{-}320}$) and DLAT **(**Dihydrolipoyllysine-residue acetyltransferase component of pyruvate dehydrogenase complex; ETDK$_{acyl}$; DLAT$_{256\text{-}259}$). Charges were shown as expected at physiological pH.

b. The tri- or tetrapeptide sequences were modified at the C-terminal lysine side chain by various acyl-chain types . These acyl-chains range from short aliphatic acylations, i.e. acetyl (C-2; K$_{ac}$), propionyl (C-3; K$_{pro}$), butyryl (C-4; K$_{but}$), over medium-sized aliphatic acylations, i.e. hexanoly (C-6; K$_{hex}$), octanoyl (C-8; K$_{oct}$), decanoyl (C-10; K$_{dec}$), to longer-chain aliphatic acylations, i.e. lauroyl (C-10; K$_{lau}$), myristoyl (C-12; K$_{myr}$) and palmitoyl (C-14; K$_{pal}$). Moreover, we also analyzed acylations such as the unsaturated crotonyl (K$_{cro}$), the branched acylations L-lactyl (K$_{L\text{-}la}$), D-β-hydroxybutyryl K$_{D\text{-}bhb}$), charged-acylations, such as succinyl (K$_{suc}$) and glutaryl (K$_{glu}$) and biotinyl (K$_{bio}$) (Fig. 3a; Supplementary Fig. 5; Supplementary Fig. 6). As positive controls, we analyzed the conversion of these lysine acylations by human HDAC3/NCoR2 (K$_{ac}$, K$_{pro}$, K$_{but}$, K$_{cro}$, K$_{L\text{-}la}$, K$_{D\text{-}bhb}$), human SIRT2 (K$_{hex}$, K$_{oct}$, K$_{dec}$, K$_{lau}$, K$_{myr}$, K$_{pal}$) and human SIRT5 (K$_{suc}$, K$_{glu}$). For deacylation of K$_{bio}$, we did not have a positive control (Fig. 3a; Supplementary Fig. 5). Charges were shown as expected at physiological pH. As AMC fluorescence does not follow a linear trend at high concentrations, we obtained a calculated conversions >100% for some enzyme-peptide combinations. To this end, we categorized these values in a single category to all conversions >48% on the heat map (Fig. 3a; Supplementary Fig. 6).

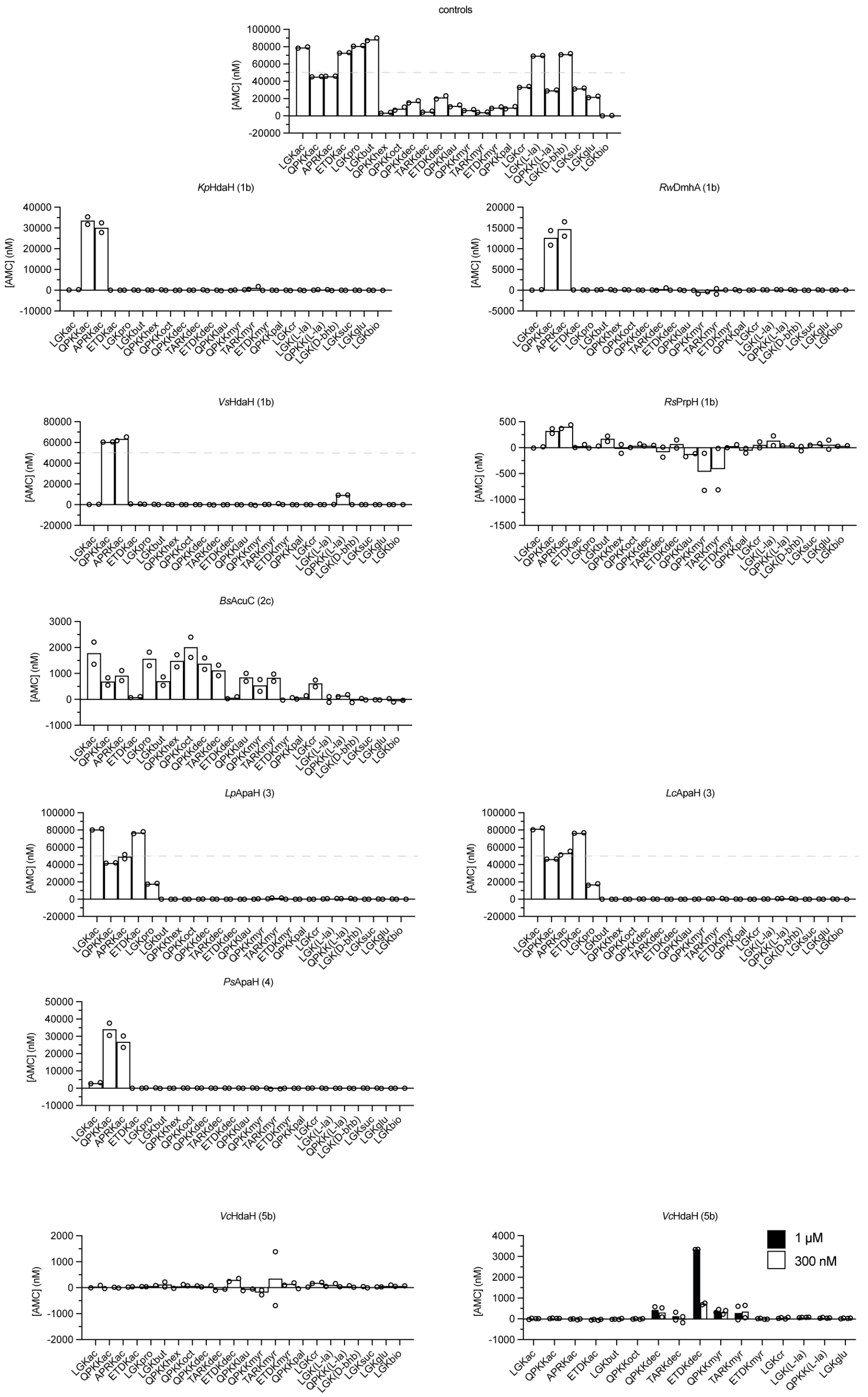

Supplementary Figure 6: Bar graphs obtained for the pre-screening to uncover acyl-chain preferences of selected bacterial deacylases. A Fluor-de-Lys assay-based screening was performed using histone H3 (APRK$_{acyl}$, H3$_{15\text{-}18}$ or TARK$_{acyl}$, H3$_{6\text{-}9}$), histone H4 (LGK$_{acyl}$, H4$_{10\text{-}12}$), p53 (QPKK$_{acyl}$, p53$_{317\text{-}320}$) and DLAT derived tri- or tetrapeptide sequences. Depicted is the conversion of peptide in released [AMC] in µM. AMC fluorescence does not follow a linear trend at high concentrations, which explains calculated conversions >100%. To this end, a single category is given to all conversions >48% and the bar graphs show a maximal conversion of 50 µM AMC (dashed grey line). Positive controls were: HDAC3/NCoR2 (K$_{ac}$, K$_{pro}$, K$_{but}$, K$_{cr}$, K$_{(L\text{-}la)}$, K$_{(D\text{-}bhb)}$), SIRT2 (K$_{hex}$, K$_{oct}$, K$_{dec}$, K$_{lau}$, K$_{myr}$, K$_{pal}$) and SIRT5 (K$_{suc}$, K$_{glu}$). For the H4-derived peptide LGK$_{bio}$ no positive contral was available. Enzyme concentration was set to 100 nM. As we did not observe a strong conversion for *Vc*HdaH (5b), follow-up screening was performed at higher enzyme concentrations of 300 nM and 1 µM. This data is the basis for the heatmap shown in Fig. 3a. The experiment was performed in two independent replicates (*n*=2). Bars depict means. Source data are provided as Source Data file.

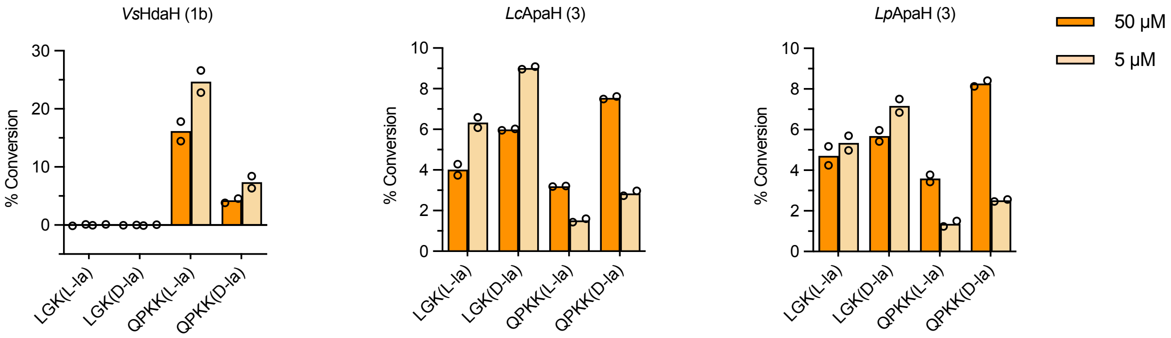

Supplementary Figure 7: Bar graphs obtained for the follow-up screening to uncover de-D/L-lactylase activity for *Vs*HdaH (1b), *Lc*ApaH (3) and *Lp*ApaH (3). A Fluor-de-Lys assay-based screening was performed using histone H4 (LGK$_{L\text{-la}}$/LGK$_{D\text{-la}}$, H4$_{10\text{-}12}$) and p53 (QPKK$_{L\text{-la}}$/QPKK$_{D\text{-la}}$, p53$_{317\text{-}320}$) tri- and tetrapeptide sequences. Depicted is the conversion of peptide in released [AMC] in µM. The de-D/L-lactylase selectivity assays were performed at 100 nM (*Vs*HdaH (1b) or 300 nM (*Lc*ApaH (3), *Lp*ApaH (3)), and at 50 µM and 5 µM substrate concentration as indicated. This data is the basis for the heatmap shown in Fig. 3a. The experiment was performed in two independent replicates (*n*=2). Bars depict means. Source data are provided as Source Data file.

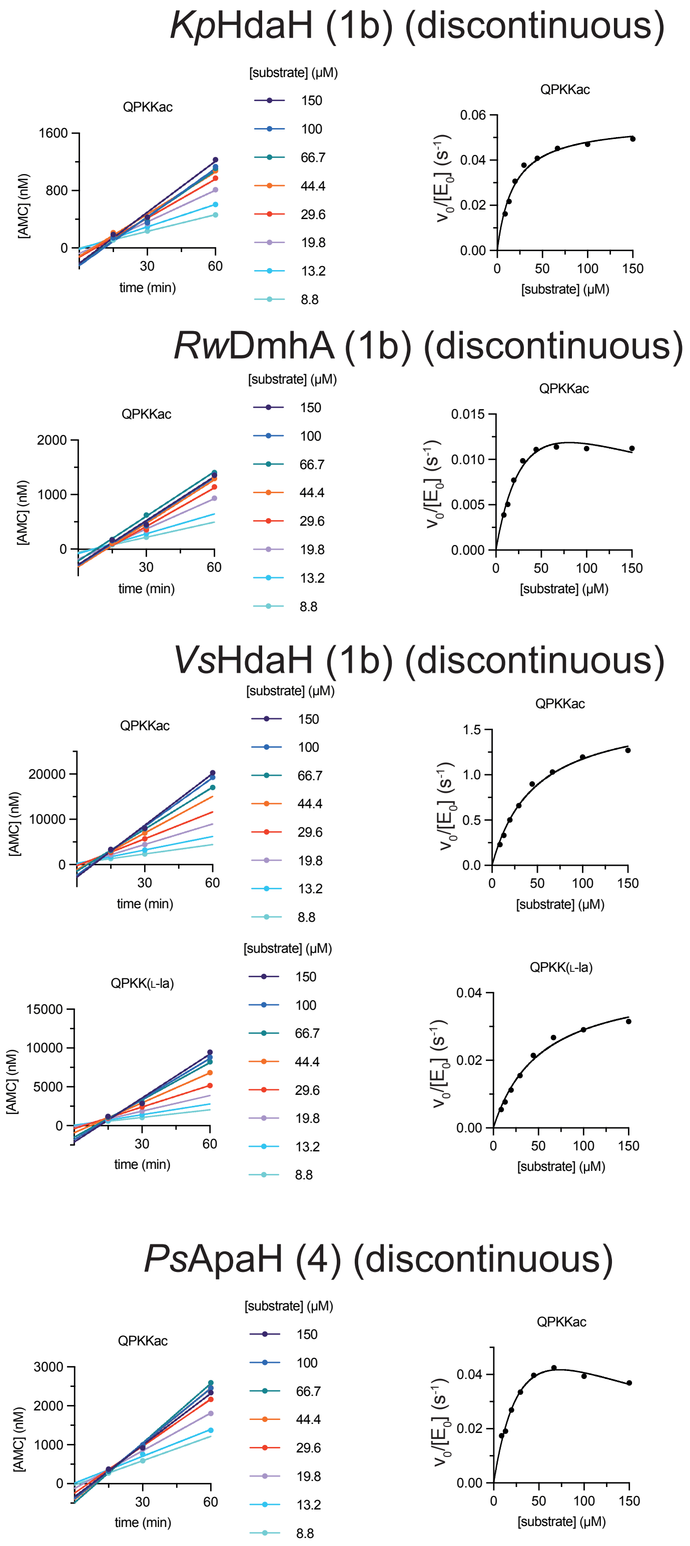

Supplementary Figure 8: Michaelis-Menten kinetics obtained for deacylation of the p53-derived QPKK-based peptides and acyl-chain types by a discontinuous assay format for bacterial deacylases *Kp*HdaH (1b), *Ps*ApaH (4), *Rw*DmhA (1b) and *Vs*HdaH (1b). For the QPKK-based substrates the discontinuous method was used for enzyme kinetics. Michaelis-Menten kinetics were performed to reveal the peptide substrate and acyl-chain preference of bacterial deacylases. The peptide sequences and acyl-chain types are indicated ($p53_{317\text{-}320}$; $QPKK_{ac}$/ $QPKK_{L\text{-}la}$). Left panels: Conversion of the AMC fluorophore as a function of time results in the initial reaction rates, v_0, (unit: $nM{*}s^{-1}$). Right panels: The initial reaction rates, v_0, were divided by the total enzyme concentration, $[E_0]$ (unit: nM) to yield the observed rate constants, $v_0/[E_0]$, (units: s^{-1}, i.e. $v_0/[E_0] = [AMC]s^{-1}/[E_0]$). The values obtained for $v_0/[E_0]$ were plotted as a function of the substrate concentration. The data was fitted to the hyperbolic Michaelis-Menten equation resulting the k_{cat}, i.e. the first-order rate constant under conditions of substrate saturation ($k_{cat}=v_{max}/[E_0]$; unit: s^{-1}), and K_M value (unit: nM). The experiment was performed in duplicates and the graph depicts the means of both recorded independent replicates (*n*=2). Source data are provided as Source Data file.

*Bs*AcuC (2c) (continuous)

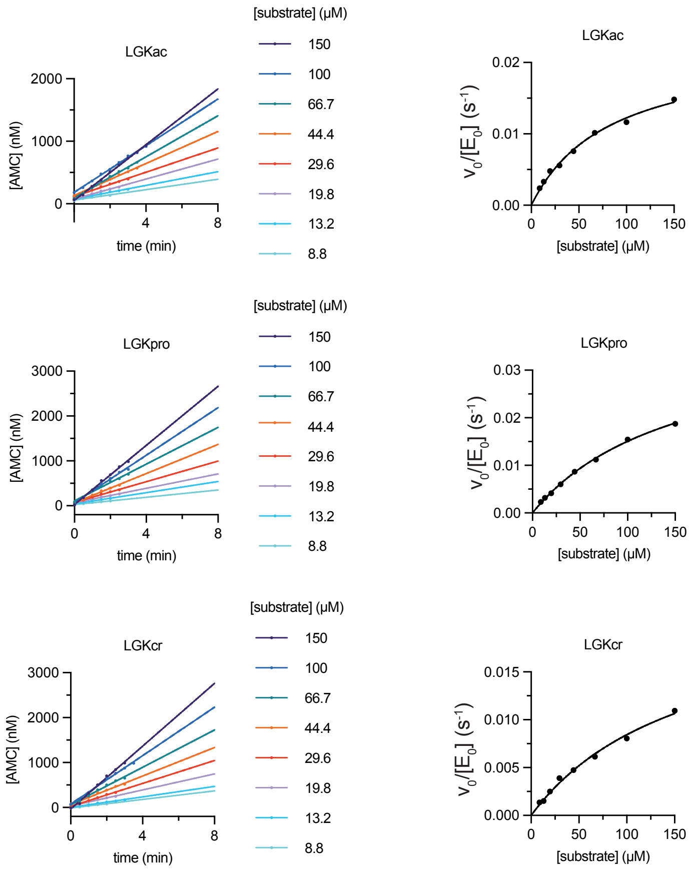

Supplementary Figure 9: Michaelis-Menten kinetics obtained for deacylation of the LGK-based peptides and acyl-chain types by a continuous assay format for bacterial deacylase *Bs*AcuC (2c)
For the LGK-based substrates the continuous method was used for enzyme kinetics. Michaelis-Menten kinetics were performed to reveal the acyl-chain preference of *Bs*AcuC (2c). The peptide sequences and acyl-chain types are indicated (histone $H4_{10-12}$; LGK_{ac}/LGK_{pro}/LGK_{cro}). Left panels: Conversion of the AMC fluorophore as a function of time results in the initial reaction rates, v_0, (unit: $nM*s^{-1}$). Right panels: The initial reaction rates, v_0, were divided by the total enzyme concentration, $[E_0]$ (unit: nM) to yield the observed rate constants, $v_0/[E_0]$, (units: s^{-1}, i.e. $v_0/[E_0] = [AMC]s^{-1}/[E_0]$). The values obtained for $v_0/[E_0]$ were plotted as a function of the substrate concentration. The data was fitted to the hyperbolic Michaelis-Menten equation resulting the k_{cat}, i.e. the first-order rate constant under conditions of substrate saturation ($k_{cat}=v_{max}/[E_0]$; unit: s^{-1}), and K_M value (unit: nM). The experiment was performed in duplicates and the graph depicts the means of both recorded independent replicates (*n*=2). Source data are provided as Source Data file.

*Lp*ApaH (3) (discontinuous)

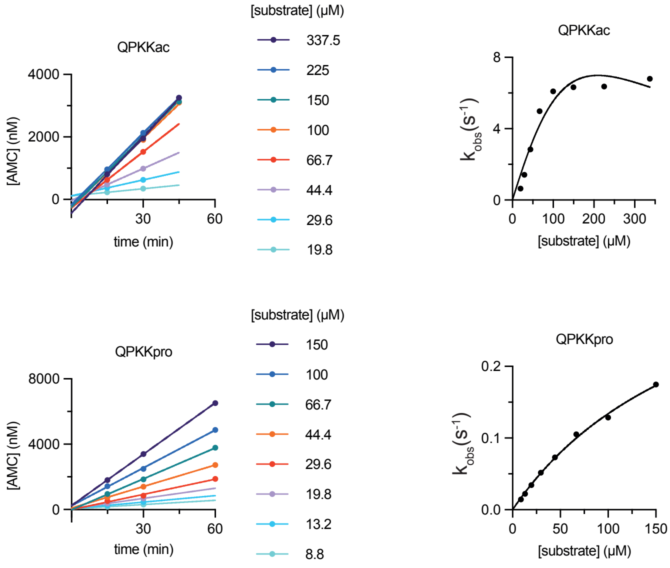

*Lc*ApaH (3) (discontinuous)

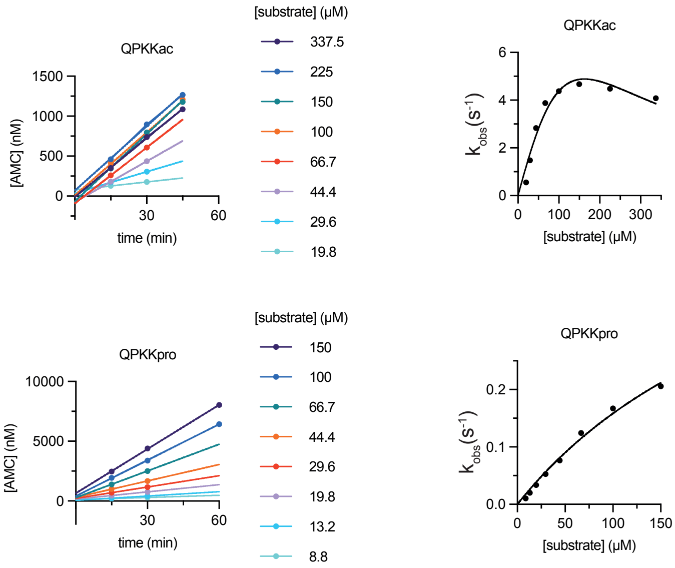

Supplementary Figure 10: Michaelis-Menten kinetics obtained for deacetylation and depropionylation of the p53-derived QPKK-based peptides and acyl-chain types by a discontinuous assay format for bacterial deacylases *Lp*ApaH (3) and *Lc*ApaH (3). For the QPKK-based substrates the discontinuous method was used for enzyme kinetics. Michaelis-Menten kinetics were performed to reveal the peptide substrate and acyl-chain preference of bacterial deacylases. The peptide sequences and acyl-chain types are indicated ($p53_{317\text{-}320}$; $QPKK_{ac}$/$QPKK_{pro}$). Left panels: Conversion of the AMC fluorophore as a function of time results in the initial reaction rates, v_0, (unit: $nM*s^{-1}$). Right panels: The initial reaction rates, v_0, were divided by the total enzyme concentration, $[E_0]$ (unit: nM) to yield the observed rate constants, $v_0/[E_0]$, (units: s^{-1}, i.e. $v_0/[E_0] = [AMC]s^{-1}/[E_0]$). The values obtained for $v_0/[E_0]$ were plotted as a function of the substrate concentration. The data was fitted to the hyperbolic Michaelis-Menten equation resulting the k_{cat}, i.e. the first-order rate constant under conditions of substrate saturation ($k_{cat}=v_{max}/[E_0]$; unit: s^{-1}), and K_M value (unit: nM). The experiment was performed in duplicates and the graph depicts the means of both recorded independent replicates (*n*=2). Source data are provided as Source Data file.

*Lc*ApaH (3) (continuous)

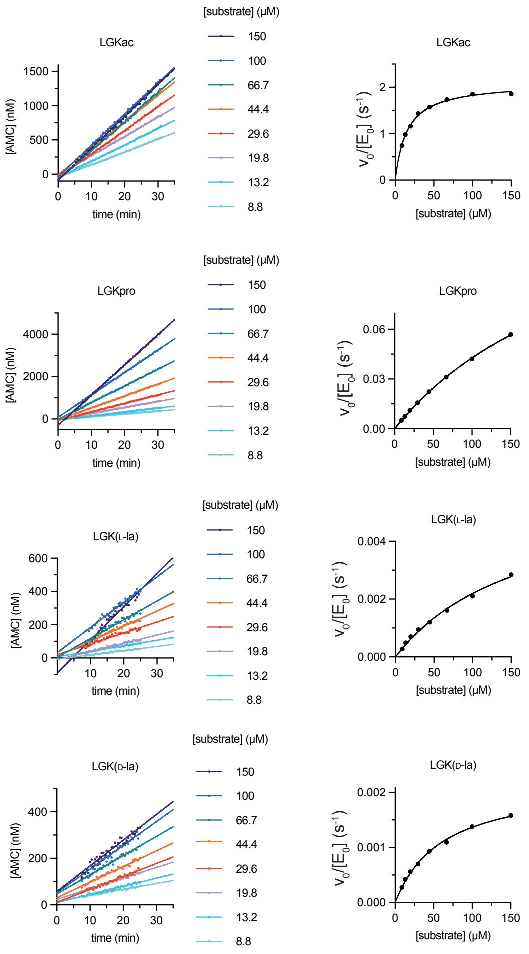

*Lp*ApaH (3) (continuous)

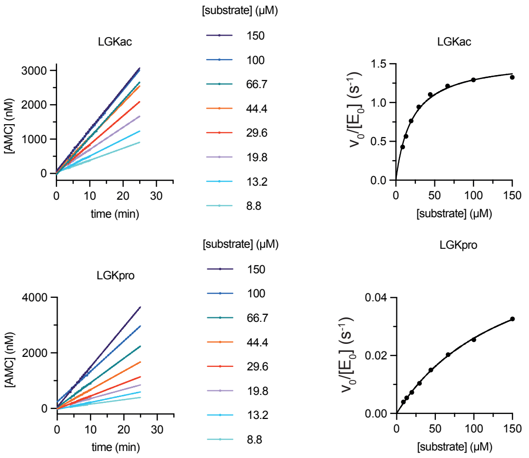

Supplementary Figure 11: Michaelis-Menten kinetics obtained for deacylation of the LGK-based peptides and acyl-chain types for bacterial deacylases *Lc*ApaH (3) and *Lp*ApaH (3). For the histone H4-derived LGK_{ac}-based substrate peptide the continuous method was used for enzyme kinetics. Michaelis-Menten kinetics were performed to reveal the acyl-chain preference of *Lc*ApaH (3) *and* LpApaH (3). The peptide sequences and acyl-chain types are indicated (histone $H4_{10-12}$; LGK_{ac}/LGK_{pro}/$LGK_{L\text{-}la}$/$LGK_{D\text{-}la}$). Left panels: Conversion of the AMC fluorophore as a function of time results in the initial reaction rates, v_0, (unit: $nM*s^{-1}$). Right panels: The initial reaction rates, v_0, were divided by the total enzyme concentration, $[E_0]$ (unit: nM) to yield the observed rate constants, $v_0/[E_0]$, (units: s^{-1}, i.e. $v_0/[E_0] = [AMC]s^{-1}/[E_0]$). The values obtained for $v_0/[E_0]$ were plotted as a function of the substrate concentration. The data was fitted to the hyperbolic Michaelis-Menten equation resulting the k_{cat}, i.e. the first-order rate constant under conditions of substrate saturation ($k_{cat}=v_{max}/[E_0]$; unit: s^{-1}), and K_M value (unit: nM). The experiment was performed in duplicates and the graph depicts the means of both recorded independent replicates (*n*=2). Source data are provided as Source Data file.

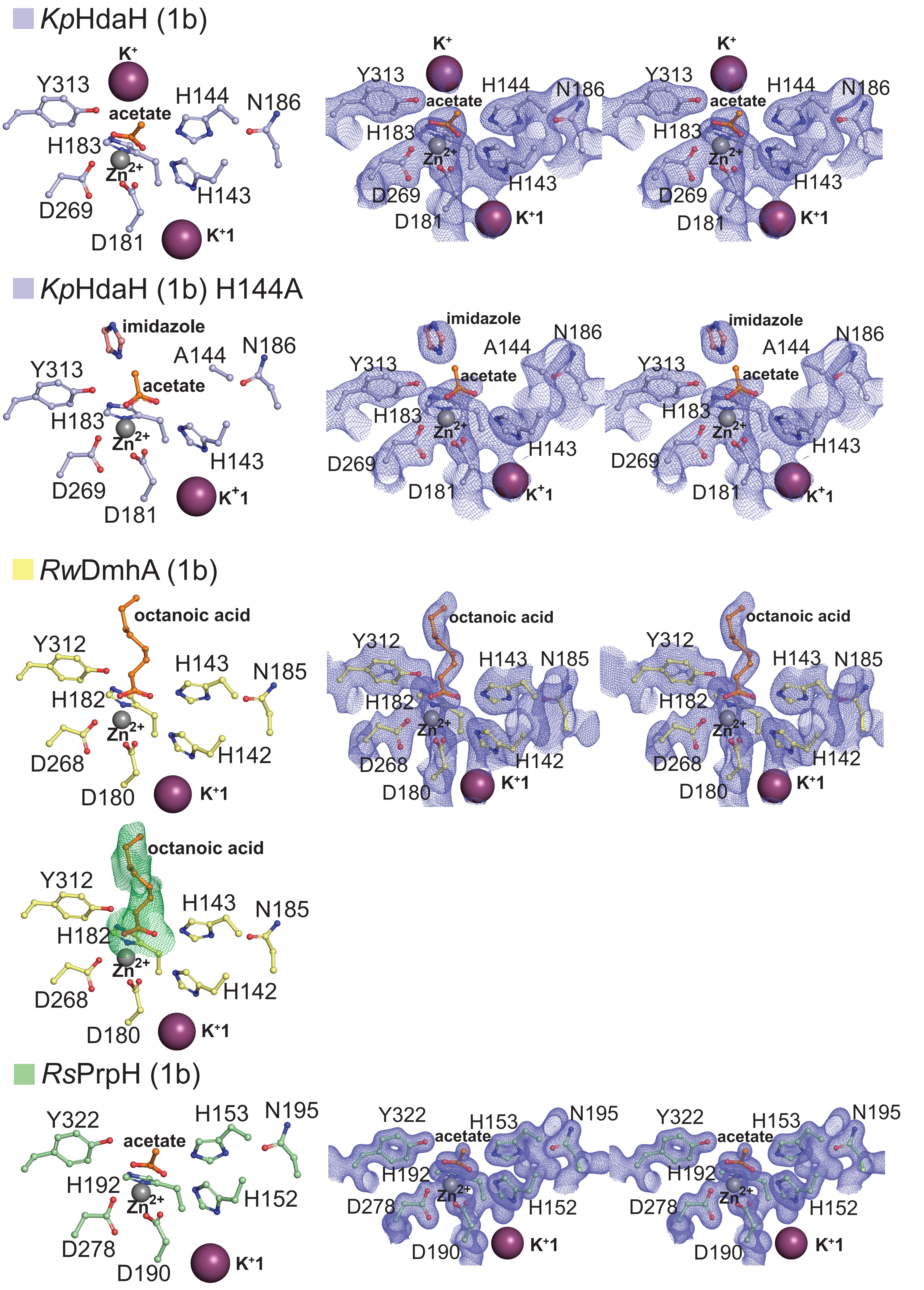
KpHdaH (1b)
K+
Y313
H144
N186
acetate
H183
Zn2+
H143
D269
D181
K+1
KpHdaH (1b) H144A
imidazole
A144
N186
Y313
acetate
H183
Zn2+
H143
D269
D181
K+1
RwDmhA (1b)
octanoic acid
Y312
H143
N185
H182
Zn2+
H142
D268
D180
K+1
RsPrpH (1b)
Y322
H153
N195
acetate
H192
Zn2+
H152
D278
D190
K+1

Supplementary Figure 12: Stereo images of representative electron density for structures of bacterial deacylases solved in this study.

Stereo figures for the structures of bacterial deacylases of sub-cluster 1b: *Kp*HdaH, *Kp*HdaH H144A, *Rw*DmhA and *Rs*PrpH of subcluster 1b. For all structures a representative closeup of the active site is shown on the left, including the catalytic Zn^{2+}-ion, the coordinating His and the two Asp residues, the catalytic base His, except for the inactivated *Kp*HdaH mutant here the His is mutated to an Ala, and the active site Tyr important to orient and polarize the carbonyl-group of the substrate and to stabilize the tetrahedral intermediate. As stereo images the closeup of the active site is shown with the is the electron density of the $2F_o$-F_c map contoured at 1σ in blue. For *Rw*DmhA (1b) the F_o-F_c omit map is shown in green for the bound octanoic acid (contour level 3σ). In violet spheres the potassium ions are shown, the catalytic Zn^{2+}-ion is shown as grey sphere. The stereo diagrams were prepared in wall-eyed viewing mode with PyMOL version 2.3.4[3].

a

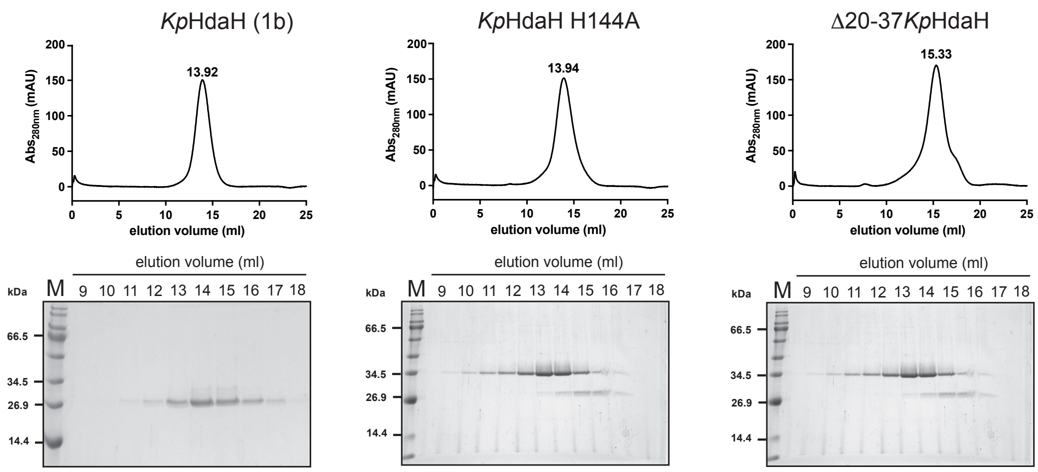

b

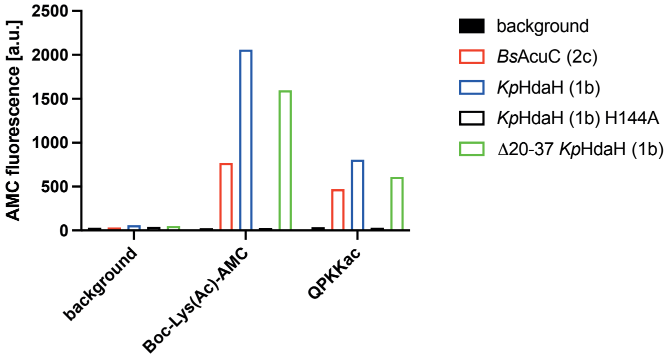

c

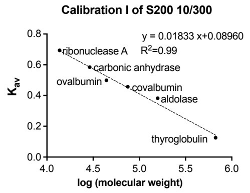

protein	exp. MW (Da)	elution volume (ml)	calc. MW (Da)	oligomeric state (calc. MW/exp. MW)
*Kp*HdaH (1b)	41569	13.9	136432	3.3
*Kp*HdaH (1b) H144A	41502	13.4	135166	3.3
Δ20-37 *Kp*HdaH (1b)	39830	15.3	70731	1.8

Supplementary Figure 13: Analytical size exclusion chromatography of the L1-loop deleted mutant Δ20-37*Kp*HdaH shows formation of a dimer and the Δ20-37*Kp*HdaH (1b) is still catalytically active albeit with a lower activity compared to wildtype *Kp*HdaH (1b).

a The analytical SEC experiment on a calibrated S200 10/300 GL column reveals the deletion of the loop abolishes tetramer formation. Instead, Δ20-37*Kp*HdaH (1b) elutes as an apparent dimer. Analyses of the crystal structure of *Kp*HdaH (1b) and interface areas suggests the side-by-side dimer being abolished upon L1-loop deletion while the head-to-head dimer stays intact.

b A Fluor-de-Lys assay shows Δ20-37*Kp*HdaH being active as lysine deacylase, however, the activity is reduced compared to wildtype *Kp*HdaH. *Bs*AcuC (2c) shows lower activity compared to Δ20-37*Kp*HdaH and wildtype *Kp*HdaH. The experiment was conducted in assay buffer (50 mM Tris/HCl, 137 mM NaCl, 2.7 mM KCl, 1 mM $MgCl_2$, 0.5 mg/mL BSA, pH 8.0). The experiment was performed once (n=1). Source data are provided as Source Data file.

c Evaluation of the oligomeric state of *Kp*HdaH (1b) and Δ20-37*Kp*HdaH as determined by analytical size exclusion chromatography. Source data are provided as Source Data file.

```
1a_Arabidopsis_thaliana_HDA14
                                        β1
                                       ──►
                               1        10
                               .        .
1a_Arabidopsis_thaliana_HDA14  MSMALI....VRP..........FFVP...................................................
1a_Candidatus_Hinthialibacter  MSFGLI.....VD..........SCF....................................................
1a_Actinobacteria_bacterium_R  MCESCGVRLLLY..................................................................
1b_Arabidopsis_thaliana_HDA8   MVTNRV......DV...............................................................F
1b_Klebsiella_pneumoniae_HdaH  MKRKTG........................................................................
1b_Vibrio_sp_vnigr-6D03_HdaH   MSKRTG........................................................................
1c_Shinella_sp_DD12_HdaH       MTTC....LF.E..................................................................
1c_Haematospirillum_jordaniae  MPT....LLV.S..................................................................
1c_Micavibrio_sp_TMED2_Acu     MSL...........................................................................
1d_Legionella_londiniensis_Ap  MAI........................................................................AFI
1d_Thioploca_ingrica_DAC       MPIA....YF.H..................................................................
1d_Neisseria_zoodegmatis_DAC   MNKIVR.....YMR...................L......................QLRRM.............LGRQ
1e_Stentor_coeruleus_HdaH      MSKGIN.....EIE...................V......................DIHEICCL..GRHSDLEEILNS
1e_Chlorella_variabilis_HdaH   MSADGM.....SG..........GSES..EDE.GPS..................SFPLHDAAQA..GDAELIHQ....
1e_Babesia_microti__strain_RI  MASGSQ.....VGT..........CYR..KFQ.P......................PLHKHILR..NDYDAIRYILSS
1f_Pneumycystis_carinii_HdaH   MANPNY.....SLN..........LSDSFYDE.TK................EASYLTSHETDSL..LDA.........
1f_Malassezia_pachydermatis_H  MVFLVK.....LPARKPADGRPPTTNE..QES..............................................
1f_Saccharomyces_cerevisiae_H  MSSKHS.....D...........PLER...................................................
1g_Homo_sapiens_HDAC4          MSSQ....SH.PD..........GLSG..RDQ.PVELLNPARVNHMPSTVDVATALPLQVAPSAVPMDLRLDHQ....
1g_Saccharomyces_cerevisiae_H  MDSVMV.....K................................................KEVL..EN..........
1g_Arabidopsis_thaliana_HDA5   MAMAGE.....SS..........GKKI..GDCDGKV..................A...........GNRQ........


1a_Arabidopsis_thaliana_HDA14

1a_Arabidopsis_thaliana_HDA14  ..............................................................................
1a_Candidatus_Hinthialibacter  ..............................................................................
1a_Actinobacteria_bacterium_R  ..............................................................................
1b_Arabidopsis_thaliana_HDA8   W..HEGMLR.....................................................................
1b_Klebsiella_pneumoniae_HdaH  ..............................................................................
1b_Vibrio_sp_vnigr-6D03_HdaH   ..............................................................................
1c_Shinella_sp_DD12_HdaH       ..............................................................................
1c_Haematospirillum_jordaniae  ..............................................................................
1c_Micavibrio_sp_TMED2_Acu     ..............................................................................
1d_Legionella_londiniensis_Ap  T..HKDCLL.....................................................................
1d_Thioploca_ingrica_DAC       ..............................................................................
1d_Neisseria_zoodegmatis_DAC   A..RAAWIS.....................................................................
1e_Stentor_coeruleus_HdaH      IDFYADIMLERDEEGFIPYHLSLIYCRHKCVDL..ILS........................................
1e_Chlorella_variabilis_HdaH   ..............................LMTPPPDAKPGPPSDDLVGLDDI.LARYVAEASAPQ.LPSINRRDHHE
1e_Babesia_microti__strain_RI  H..HRDCINDMDHDGRNAFHLALQTANSKALYL..LLYYPFVHTNQVF..ATLDWTCDSDDEIDRDIVHVG.......
1f_Pneumycystis_carinii_HdaH   ..............................................................................
1f_Malassezia_pachydermatis_H  ..............................................................................
1f_Saccharomyces_cerevisiae_H  ..............................................................................
1g_Homo_sapiens_HDAC4          ..............................FSL..PVAEPALREQQLQ..QEL.LALKQKQQIQRQILIAEFQRQHEQ
1g_Saccharomyces_cerevisiae_H  ..............................................................................
1g_Arabidopsis_thaliana_HDA5   ..............................................................................


1a_Arabidopsis_thaliana_HDA14

1a_Arabidopsis_thaliana_HDA14  ...GSA........................................................................
1a_Candidatus_Hinthialibacter  ..............................................................................
1a_Actinobacteria_bacterium_R  ..............................................................................
1b_Arabidopsis_thaliana_HDA8   ..............................................................HDAVEGV.........
1b_Klebsiella_pneumoniae_HdaH  ..............................................................................
1b_Vibrio_sp_vnigr-6D03_HdaH   ..............................................................................
1c_Shinella_sp_DD12_HdaH       ..............................................................................
1c_Haematospirillum_jordaniae  ..............................................................................
1c_Micavibrio_sp_TMED2_Acu     ..............................................................................
1d_Legionella_londiniensis_Ap  ..............................................................HNT.............
1d_Thioploca_ingrica_DAC       ..............................................................................
1d_Neisseria_zoodegmatis_DAC   ..............................................................HPL.............
1e_Stentor_coeruleus_HdaH      ..............................................................................
1e_Chlorella_variabilis_HdaH   CTPLHVALLHGQLE........................................AARA.........LLEHGA.....
1e_Babesia_microti__strain_RI  .............NAKPAYQAGDRLLMKWNETPDLYKKAAFSALEGYSIRGCGLRIVEDLYEQHYKYILDKPN.....
1f_Pneumycystis_carinii_HdaH   .......ILNISL...........................................................KDEETK
1f_Malassezia_pachydermatis_H  ..............................................................................
1f_Saccharomyces_cerevisiae_H  ..............................................................................
1g_Homo_sapiens_HDAC4          LSRQHEAQLHEHIK........................................QQQEMLAMKHQQELLEHQR.....
1g_Saccharomyces_cerevisiae_H  ............P...........................................................DHDLKR
1g_Arabidopsis_thaliana_HDA5   ...RKV........................................................................
```

1a_Arabidopsis_thaliana_HDA14

```
1a_Arabidopsis_thaliana_HDA14   .............................................................................
1a_Candidatus_Hinthialibacter   .............................................................................
1a_Actinobacteria_bacterium_R   .............................................................................
1b_Arabidopsis_thaliana_HDA8    .............................................................................
1b_Klebsiella_pneumoniae_HdaH   .............................................................................
1b_Vibrio_sp_vnigr-6D03_HdaH    .............................................................................
1c_Shinella_sp_DD12_HdaH        .............................................................................
1c_Haematospirillum_jordaniae   .............................................................................
1c_Micavibrio_sp_TMED2_Acu      .............................................................................
1d_Legionella_londiniensis_Ap   .............................................................................
1d_Thioploca_ingrica_DAC        .............................................................................
1d_Neisseria_zoodegmatis_DAC    .............................................................................
1e_Stentor_coeruleus_HdaH       .............................................................................
1e_Chlorella_variabilis_HdaH    SLE..........................................................................
1e_Babesia_microti__strain_RI   TLERSAREY........TAKYARLYSKEINFPEKIETID..ELKEKNYISTIITSEIVMEKDD...NTRVSFASDFI
1f_Pneumycystis_carinii_HdaH    KLSDAVDEQ....................................................................
1f_Malassezia_pachydermatis_H   .VP..........................................................................
1f_Saccharomyces_cerevisiae_H   .............................................................................
1g_Homo_sapiens_HDAC4           KLERHRQEQELEKQHREQK..................LQQLKNKEKGKESAVASTEVKMKLQEFVLNKKKALAHRNL
1g_Saccharomyces_cerevisiae_H   KLEENKEEE.....................................NSLSTTSKSKRQVIV................
1g_Arabidopsis_thaliana_HDA5    .............................................................................
```

1a_Arabidopsis_thaliana_HDA14

```
                                                                                   20
1a_Arabidopsis_thaliana_HDA14   ..................................................................GISGSRNICKK
1a_Candidatus_Hinthialibacter   .............................................................................
1a_Actinobacteria_bacterium_R   .............................................................................
1b_Arabidopsis_thaliana_HDA8    .............................................................................
1b_Klebsiella_pneumoniae_HdaH   .............................................................................
1b_Vibrio_sp_vnigr-6D03_HdaH    .............................................................................
1c_Shinella_sp_DD12_HdaH        .............................................................................
1c_Haematospirillum_jordaniae   .............................................................................
1c_Micavibrio_sp_TMED2_Acu      ..................................................................SI.........
1d_Legionella_londiniensis_Ap   .............................................................................
1d_Thioploca_ingrica_DAC        .............................................................................
1d_Neisseria_zoodegmatis_DAC    .............................................................................
1e_Stentor_coeruleus_HdaH       .............LSIDFQVPYQGFMPIHLAMACAG.........................................
1e_Chlorella_variabilis_HdaH    ..................LGCEGSPPLHVAVCVAAHGAMQEFALAAMALLLQFGADPYERNNNGRTAL.....H...
1e_Babesia_microti__strain_RI   DSCDNFELQPIPLETVDLLVSFDMTSPIHLLFSNIY.........................................
1f_Pneumycystis_carinii_HdaH    .............................................................................
1f_Malassezia_pachydermatis_H   ..................SGVHAVRPVTIVPATAASTTTTETPAH................................
1f_Saccharomyces_cerevisiae_H   .............................................................................
1g_Homo_sapiens_HDAC4           NHCISSDPR..YWYGKTQHSSLDQSSPPQSGV.............................................
1g_Saccharomyces_cerevisiae_H   .............................................................................
1g_Arabidopsis_thaliana_HDA5    ..................................................................GL.........
```

1a_Arabidopsis_thaliana_HDA14

```
                                  30
1a_Arabidopsis_thaliana_HDA14   NQWRKYL......................................................................
1a_Candidatus_Hinthialibacter   .............................................................................
1a_Actinobacteria_bacterium_R   .............................................................................
1b_Arabidopsis_thaliana_HDA8    .............................................................................
1b_Klebsiella_pneumoniae_HdaH   .............................................................................
1b_Vibrio_sp_vnigr-6D03_HdaH    .............................................................................
1c_Shinella_sp_DD12_HdaH        .............................................................................
1c_Haematospirillum_jordaniae   .............................................................................
1c_Micavibrio_sp_TMED2_Acu      ....FTH......................................................................
1d_Legionella_londiniensis_Ap   .............................................................................
1d_Thioploca_ingrica_DAC        .............................................................................
1d_Neisseria_zoodegmatis_DAC    .............................................................................
1e_Stentor_coeruleus_HdaH       ...............................................FPECHEDLIQTVNCL...LK..........
1e_Chlorella_variabilis_HdaH    .....WAA.....QMDFSAAAEAVLA................................................AAE
1e_Babesia_microti__strain_RI   .........................IESYREKILDCLKILITFFRVFDHKE...TVDSVKCMHLKNI..........
1f_Pneumycystis_carinii_HdaH    .............................................................................
1f_Malassezia_pachydermatis_H   .......MMPAAEDTDGAAFQEAVPA................................................PAL
1f_Saccharomyces_cerevisiae_H   ....FYK......................................................................
1g_Homo_sapiens_HDAC4           .........................STSYNHPVL.........GMYD..................AKDDFPLRKTAS
1g_Saccharomyces_cerevisiae_H   .....PV.................................................................CMPKI
1g_Arabidopsis_thaliana_HDA5    ....IYD......................................................................
```

1a_Arabidopsis_thaliana_HDA14

```
1a_Arabidopsis_thaliana_HDA14 ..............................................................................
1a_Candidatus_Hinthialibacter ..............................................................................
1a_Actinobacteria_bacterium_R ..............................................................................
1b_Arabidopsis_thaliana_HDA8  ..............................................................................
1b_Klebsiella_pneumoniae_HdaH ..............................................................................
1b_Vibrio_sp_vnigr-6D03_HdaH  ..............................................................................
1c_Shinella_sp_DD12_HdaH      ..............................................................................
1c_Haematospirillum_jordaniae ..............................................................................
1c_Micavibrio_sp_TMED2_Acu    ..............................................................................
1d_Legionella_londiniensis_Ap ..............................................................................
1d_Thioploca_ingrica_DAC      ..............................................................................
1d_Neisseria_zoodegmatis_DAC  .............................................................F................
1e_Stentor_coeruleus_HdaH     ..................HK.............................QDVTSRDRLGRTALHIVCAYGL..AD.LI
1e_Chlorella_variabilis_HdaH  K...KR...............................AQLRAEQAAAAAAAKAASAAAAAGAPAPAAPAAG..EG.ED
1e_Babesia_microti__strain_RI ..................EKR.DCSSSLLEMMLRKNDYLKPMI...SWNEFISYRDHTLSTVLHRACQVKS..GE.LV
1f_Pneumycystis_carinii_HdaH  .EILEN...............................YSTPET...KHFDFLSSNI....LL..PIEKEESK..VSST
1f_Malassezia_pachydermatis_H S...TH...............................TSMPSTPGPAPGDETSQNLPRTPPAAEPPTPAPS..KAAAL
1f_Saccharomyces_cerevisiae_H ...............................................QFQAFVQNNPNVISAARAAAQIPE..SA.KA
1g_Homo_sapiens_HDAC4         E...PNLKLRSRLKQKVAE.RRSSPLLRRKDGPVVTALKKRP....LDVTDSACS...SAPGSGPSSPNNSSGSV.SA
1g_Saccharomyces_cerevisiae_H HYSPL.......................................................................KT
1g_Arabidopsis_thaliana_HDA5  ..............................................................................
```

1a_Arabidopsis_thaliana_HDA14

```
1a_Arabidopsis_thaliana_HDA14 .............................................L................................
1a_Candidatus_Hinthialibacter ..............................................................................
1a_Actinobacteria_bacterium_R ..............................................................................
1b_Arabidopsis_thaliana_HDA8  ...FDTGYDP....................................................................
1b_Klebsiella_pneumoniae_HdaH ..............................................................................
1b_Vibrio_sp_vnigr-6D03_HdaH  ..............................................................................
1c_Shinella_sp_DD12_HdaH      ..............................................................................
1c_Haematospirillum_jordaniae ..............................................................................
1c_Micavibrio_sp_TMED2_Acu    ..............................................................................
1d_Legionella_londiniensis_Ap ..............................................................................
1d_Thioploca_ingrica_DAC      ..............................................................................
1d_Neisseria_zoodegmatis_DAC  ..............................................................................
1e_Stentor_coeruleus_HdaH     PILVQAGVKPELKDFSGKMPIHYAIENYQAECFKQL.........LQEMGS...........................
1e_Chlorella_variabilis_HdaH  DDSVPPPIFVFQD.KQGCTALHLAARQGHVAAVRLL.........L................................
1e_Babesia_microti__strain_RI SMLLKSGFDPLAINENGELPVHLAVDSLDPDLFKTI.........LDATLVAIFAKYEDESAEICKSSIVNQNSLQLD
1f_Pneumycystis_carinii_HdaH  L.............................................................................
1f_Malassezia_pachydermatis_H D.............................................................................
1f_Saccharomyces_cerevisiae_H VV............................................................................
1g_Homo_sapiens_HDAC4         ENGIAPAVPSIP..AETSLAHRLVAREGSAAPLPLYTSPSLPNITL................................
1g_Saccharomyces_cerevisiae_H G.............................................................................
1g_Arabidopsis_thaliana_HDA5  ..............................................................................
```

1a_Arabidopsis_thaliana_HDA14

```
1a_Arabidopsis_thaliana_HDA14 ............K.................................................................
1a_Candidatus_Hinthialibacter ..............................................................................
1a_Actinobacteria_bacterium_R ..............................................................................
1b_Arabidopsis_thaliana_HDA8  .....................................GFL......................................
1b_Klebsiella_pneumoniae_HdaH ..............................................................................
1b_Vibrio_sp_vnigr-6D03_HdaH  ..............................................................................
1c_Shinella_sp_DD12_HdaH      ..............................................................................
1c_Haematospirillum_jordaniae ..............................................................................
1c_Micavibrio_sp_TMED2_Acu    ..............................................................................
1d_Legionella_londiniensis_Ap ..............................................................................
1d_Thioploca_ingrica_DAC      ..............................................................................
1d_Neisseria_zoodegmatis_DAC  ..............................................................................
1e_Stentor_coeruleus_HdaH     .........................................D...................................M
1e_Chlorella_variabilis_HdaH  ............GA.AAGGGGGPEA.................................................VAAL
1e_Babesia_microti__strain_RI RRILSAIG.....NTQV..................LLKLLDG...................................L
1f_Pneumycystis_carinii_HdaH  ..............................................................................
1f_Malassezia_pachydermatis_H ..............................................................................
1f_Saccharomyces_cerevisiae_H ..............................................................................
1g_Homo_sapiens_HDAC4         .......GLPATGP.SAGTAGQQDAERLTLPALQQRLSLFPGTHLTPYLSTSPLERDGGAAHSPLLQHMVLLEQPPAQ
1g_Saccharomyces_cerevisiae_H ..............................................................................
1g_Arabidopsis_thaliana_HDA5  ..............................................................................
```

1a_Arabidopsis_thaliana_HDA14

```
1a_Arabidopsis_thaliana_HDA14  ..............................................................................
1a_Candidatus_Hinthialibacter  ..............................................................................
1a_Actinobacteria_bacterium_R  ..............................................................................
1b_Arabidopsis_thaliana_HDA8   ..................DVLEK.......................................................
1b_Klebsiella_pneumoniae_HdaH  ..............................................................................
1b_Vibrio_sp_vnigr-6D03_HdaH   ..............................................................................
1c_Shinella_sp_DD12_HdaH       ..............................................................................
1c_Haematospirillum_jordaniae  ..............................................................................
1c_Micavibrio_sp_TMED2_Acu     ..............................................................................
1d_Legionella_londiniensis_Ap  ..............................................................................
1d_Thioploca_ingrica_DAC       ..............................................................................
1d_Neisseria_zoodegmatis_DAC   ..............................................................................
1e_Stentor_coeruleus_HdaH      FFCTDN.......................................................................R
1e_Chlorella_variabilis_HdaH   CRTRSKA.......................................................................
1e_Babesia_microti__strain_RI  LTCDNNSSKVRDGSRAEMDYLKNNSQKDKIFNNKLILKEDHTFLKQVPGEVIFERLFSKYGECFITTISSLFSELLLF
1f_Pneumycystis_carinii_HdaH   ..............................................................................
1f_Malassezia_pachydermatis_H  ..............................................................................
1f_Saccharomyces_cerevisiae_H  ..............................................................................
1g_Homo_sapiens_HDAC4          APLVTGL.......................................................................
1g_Saccharomyces_cerevisiae_H  ..............................................................................
1g_Arabidopsis_thaliana_HDA5   ..............................................................................
```

1a_Arabidopsis_thaliana_HDA14

```
1a_Arabidopsis_thaliana_HDA14  ..............................................................................
1a_Candidatus_Hinthialibacter  ..............................................................................
1a_Actinobacteria_bacterium_R  ..............................................................................
1b_Arabidopsis_thaliana_HDA8   ..............................................................................
1b_Klebsiella_pneumoniae_HdaH  ..............................................................................
1b_Vibrio_sp_vnigr-6D03_HdaH   ..............................................................................
1c_Shinella_sp_DD12_HdaH       ..............................................................................
1c_Haematospirillum_jordaniae  ..............................................................................
1c_Micavibrio_sp_TMED2_Acu     ..............................................................................
1d_Legionella_londiniensis_Ap  ..............................................................................
1d_Thioploca_ingrica_DAC       ..............................................................................
1d_Neisseria_zoodegmatis_DAC   ..............................................................................
1e_Stentor_coeruleus_HdaH      GDKPIHTSVRAGSWECFNILITF...........................................GSEEMLNENNEF
1e_Chlorella_variabilis_HdaH   GHNALHLAALAGSSRCAALLAAA............................................AL.........
1e_Babesia_microti__strain_RI  FEQLFYRCIHRNSWRVFLALASFNNTLTFHMFSSPLCVHRFIKFAHSMGNVYAFFSILNYSVRLLYPNETKCVENMDF
1f_Pneumycystis_carinii_HdaH   ..............................................................................
1f_Malassezia_pachydermatis_H  ..............................................................................
1f_Saccharomyces_cerevisiae_H  ..............................................................................
1g_Homo_sapiens_HDAC4          GALPLHAQSLVGADRVSPSIHKL............................................RQ.........
1g_Saccharomyces_cerevisiae_H  ..............................................................................
1g_Arabidopsis_thaliana_HDA5   ..............................................................................
```

1a_Arabidopsis_thaliana_HDA14

```
1a_Arabidopsis_thaliana_HDA14  ..............................................................................
1a_Candidatus_Hinthialibacter  ..............................................................................
1a_Actinobacteria_bacterium_R  ..............................................................................
1b_Arabidopsis_thaliana_HDA8   ..............................................................................
1b_Klebsiella_pneumoniae_HdaH  ..............................................................................
1b_Vibrio_sp_vnigr-6D03_HdaH   ..............................................................................
1c_Shinella_sp_DD12_HdaH       ..............................................................................
1c_Haematospirillum_jordaniae  ..............................................................................
1c_Micavibrio_sp_TMED2_Acu     ..............................................................................
1d_Legionella_londiniensis_Ap  ..............................................................................
1d_Thioploca_ingrica_DAC       ..............................................................................
1d_Neisseria_zoodegmatis_DAC   ..............................................................................
1e_Stentor_coeruleus_HdaH      E.......................ETP........................................EEVA.......
1e_Chlorella_variabilis_HdaH   ..............DAAAGKSKLGLTP........................................ADLAAR.....
1e_Babesia_microti__strain_RI  ELGDMSMTFNRRNVKVEKGQDKITPAP........................................TNFAQY.....
1f_Pneumycystis_carinii_HdaH   ..............................................................................
1f_Malassezia_pachydermatis_H  ..............................................................................
1f_Saccharomyces_cerevisiae_H  ..............................................................................
1g_Homo_sapiens_HDAC4          ..............HRPLGRTQSAPLPQNAQALQHLVIQQQHQQFLEKHKQQFQQQQLQMNKIIPKPSEPARQPESHP
1g_Saccharomyces_cerevisiae_H  ..............................................................................
1g_Arabidopsis_thaliana_HDA5   ..............................................................................
```

1a_Arabidopsis_thaliana_HDA14

```
1a_Arabidopsis_thaliana_HDA14   ..............................................................................
1a_Candidatus_Hinthialibacter   ..............................................................................
1a_Actinobacteria_bacterium_R   ..............................................................................
1b_Arabidopsis_thaliana_HDA8    ..............................................................................
1b_Klebsiella_pneumoniae_HdaH   ..............................................................................
1b_Vibrio_sp_vnigr-6D03_HdaH    ..............................................................................
1c_Shinella_sp_DD12_HdaH        ..............................................................................
1c_Haematospirillum_jordaniae   ..............................................................................
1c_Micavibrio_sp_TMED2_Acu      ..............................................................................
1d_Legionella_londiniensis_Ap   ..............................................................................
1d_Thioploca_ingrica_DAC        ..............................................................................
1d_Neisseria_zoodegmatis_DAC    ..............................................................................
1e_Stentor_coeruleus_HdaH       ........................................RNCGLY..............NE............FVR.
1e_Chlorella_variabilis_HdaH    .......R................................RHTGLQ..............TALRGQRPVQALQALAA.
1e_Babesia_microti__strain_RI   ......NNNKTEMNSSSNYSAGIGSKRGVDDIKQIVNDYRGCGYPETPLRRVKIPCKPFSK.............FEN.
1f_Pneumycystis_carinii_HdaH    ..............................................................................
1f_Malassezia_pachydermatis_H   ..............................................................................
1f_Saccharomyces_cerevisiae_H   ..............................................................................
1g_Homo_sapiens_HDAC4           EETEEELR................................EHQALL..............DEP............YLD
1g_Saccharomyces_cerevisiae_H   ..............................................................................
1g_Arabidopsis_thaliana_HDA5    ..............................................................................
```

1a_Arabidopsis_thaliana_HDA14

```
1a_Arabidopsis_thaliana_HDA14   ..............................................................................
1a_Candidatus_Hinthialibacter   ..............................................................................
1a_Actinobacteria_bacterium_R   ..............................................................................
1b_Arabidopsis_thaliana_HDA8    ..............................................................................
1b_Klebsiella_pneumoniae_HdaH   .......................................................FFF....................
1b_Vibrio_sp_vnigr-6D03_HdaH    .......................................................FLY....................
1c_Shinella_sp_DD12_HdaH        ..............................................................................
1c_Haematospirillum_jordaniae   ..............................................................................
1c_Micavibrio_sp_TMED2_Acu      ..............................................................................
1d_Legionella_londiniensis_Ap   ..............................................................................
1d_Thioploca_ingrica_DAC        ..............................................................................
1d_Neisseria_zoodegmatis_DAC    .........................................................L....................
1e_Stentor_coeruleus_HdaH       ..............................ARQG.........DVFPSSAYKGTLLIT....................
1e_Chlorella_variabilis_HdaH    ..............................VQEA.........EAGQREAQQRTLLMAP...................
1e_Babesia_microti__strain_RI   ..............................INLSNFEGCNVNQLLSNASINKRTWIIT....................
1f_Pneumycystis_carinii_HdaH    .......................................................VIL....................
1f_Malassezia_pachydermatis_H   .......................................................ILL....................
1f_Saccharomyces_cerevisiae_H   ........................................................VL....................
1g_Homo_sapiens_HDAC4           RLPGQKEAHAQAGVQVKQEPIESDEEEAEPPREV.........EPGQRQPSEQELLFRQQALLLEQQRIHQLRNYQAS
1g_Saccharomyces_cerevisiae_H   .......................................................LCY....................
1g_Arabidopsis_thaliana_HDA5    ..............................................................................
```

1a_Arabidopsis_thaliana_HDA14

```
                                                                                TT    ℓℓℓℓ.....
                                                                    40           50           60
1a_Arabidopsis_thaliana_HDA14   ................................................PSG.SSINCSFSTEKNPLLPSIQQL.....
1a_Candidatus_Hinthialibacter   ....................................................RDHAT.....................
1a_Actinobacteria_bacterium_R   ...............................................THPACWEHD......................
1b_Arabidopsis_thaliana_HDA8    ..............................................................................
1b_Klebsiella_pneumoniae_HdaH   ...............................................DERC.FWHST...............GLHAVT
1b_Vibrio_sp_vnigr-6D03_HdaH    ...............................................DEQC.FWHTA...............GEHALV
1c_Shinella_sp_DD12_HdaH        ...............................................NPIF.LEHHT.....................
1c_Haematospirillum_jordaniae   ...............................................HHDCL.EHE......................
1c_Micavibrio_sp_TMED2_Acu      .................................................DSGLTHDT...............G.....
1d_Legionella_londiniensis_Ap   ........................................................................G.....
1d_Thioploca_ingrica_DAC        ...............................................HPECL.LHD......................
1d_Neisseria_zoodegmatis_DAC    ...............................................HYGC.....................G.....
1e_Stentor_coeruleus_HdaH       ...............................................DETC.KLHAS...............IHESLK
1e_Chlorella_variabilis_HdaH    ................................................PEC.LFHFT...............C....P
1e_Babesia_microti__strain_RI   ...............................................HPIC.LEHLA...............IPEPTD
1f_Pneumycystis_carinii_HdaH    ...............................................HEGC.IKHEF.....................
1f_Malassezia_pachydermatis_H   ...............................................APSV.LRHRY...............V.....
1f_Saccharomyces_cerevisiae_H   ...............................................SPYS.LQHVF.....................
1g_Homo_sapiens_HDAC4           MEAAGIPVSFGGHRPLSRAQSSPASATFPVSVQEPPTKPRFTTGLVYDTLML.KHQC.....................
1g_Saccharomyces_cerevisiae_H   ...............................................DVRM.RYHAK.....................
1g_Arabidopsis_thaliana_HDA5    ................................................ETM.CKHDT...............P.....
```

```
                                     α1           β2                     α2
1a_Arabidopsis_thaliana_HDA14    ................     -->     TT      .....................
                                                     70         80     90                  100

1a_Arabidopsis_thaliana_HDA14    ................ADARLIYSVSAALGHNKES...HPECSARVPAIVN....AL....EM..N......E..LT.
1a_Candidatus_Hinthialibacter    .......G.......R....................HPERPERLDSIDS....AF....KE..H...........
1a_Actinobacteria_bacterium_R    ......TG.......G.................W...HPERAARLDAAIE....GA....RR..S......S..L..
1b_Arabidopsis_thaliana_HDA8     .....................................HPENADRVRN....ML.SILRR.GP.........I..
1b_Klebsiella_pneumoniae_HdaH    LPVGGWVQP....PAGG................G...HAESPETKRRMKK....LM....DV..S......G..L..
1b_Vibrio_sp_vnigr-6D03_HdaH     LPVGDWVQP....MSAG................G...HAESPETKRRLKN....LL....DA..S......G..I..
1c_Shinella_sp_DD12_HdaH         ........P.......E................G...HPERSDRIRAINL....AL....EH..E......R..F..
1c_Haematospirillum_jordaniae    ......TG.......G................Y...HPENPDRLRSVL.....QI....LE..S......E..D..
1c_Micavibrio_sp_TMED2_Acu       ...............P................G...HPE...RI..DR......L....V...S......V..L..
1d_Legionella_londiniensis_Ap    ...............D................F...HPECPERLQVIND....NL....SQ.SA.........I..
1d_Thioploca_ingrica_DAC         ......MG.......K................G...HPERASRINAIED....QL....QA..S......R..L..
1d_Neisseria_zoodegmatis_DAC     ...............N................G...HPESAERIKAIEA....EI.......KK.........QGF
1e_Stentor_coeruleus_HdaH        TPQTLYKQI......K................I...QPENPRRLEILVD....LPFGALLT.SD.........L..
1e_Chlorella_variabilis_HdaH     APITRETSD......S................A...PPENVERLTVLTKPGWGIL....RC..D......E..F..
1e_Babesia_microti__strain_RI    MPQRRKKMT......S................N...FVENPTRLEVIIS....NDNAILRT.DI.........L..
1f_Pneumycystis_carinii_HdaH     ......ERNK...HDLK................Y...IVERPRRYRAAII....GT....LSAKAHLDLLDK..S..
1f_Malassezia_pachydermatis_H    ......RG....VDKS................N...IVERPERIRAVLL....GI....A...G......VQGM..
1f_Saccharomyces_cerevisiae_H    ......PREWVTKSYRK................T...IVERPERLLASSM....GI....SA.........AITM..
1g_Homo_sapiens_HDAC4            ....TCGSS.......S................S...HPEHAGRIQSIWS....RL....QE..T......G..L..
1g_Saccharomyces_cerevisiae_H    .....I.......FTSY..............FEYIDPHPEDPRRIYRIYK....IL....AE..N......G..L..
1g_Arabidopsis_thaliana_HDA5     ...............D..............GED...HPECPDRI.RVI......W....EK..L......Q..LA.


                                                α3                          η1                           β3
1a_Arabidopsis_thaliana_HDA14                    .......................................................   -..-->
                                                                                                         110

1a_Arabidopsis_thaliana_HDA14    ..............PKFRG......................................SQ.I..LELA.
1a_Candidatus_Hinthialibacter    .................KI...................................LE..SC.HCLSFEP.
1a_Actinobacteria_bacterium_R    .................EV......................................AD.R...EAP.
1b_Arabidopsis_thaliana_HDA8     A...............PHV......................................NWF....T.G.
1b_Klebsiella_pneumoniae_HdaH    .T.............P.Q.L.....................................AL.R...S.A.
1b_Vibrio_sp_vnigr-6D03_HdaH     .T.............K.H.L.....................................TL.S...S.A.
1c_Shinella_sp_DD12_HdaH         .S.............P.L.L......................................R.E...K.A.
1c_Haematospirillum_jordaniae    .F.............F..ML.....................................HR.E...E.A.
1c_Micavibrio_sp_TMED2_Acu       .A.............A...L.....................................EE.E..PFNAC
1d_Legionella_londiniensis_Ap    QS...............SLV.....................................W.E....T.A.
1d_Thioploca_ingrica_DAC         .L.............E..LV.....................................RY.Y...E.A.
1d_Neisseria_zoodegmatis_DAC     W................DVF.....................................QKIE...A.A.
1e_Stentor_coeruleus_HdaH        S................FAT.....................................WKK....S.S.
1e_Chlorella_variabilis_HdaH     .G.............G..AL.....................................RWDE....RS.
1e_Babesia_microti__strain_RI    E................GVY.....................................ILQ....S.P.
1f_Pneumycystis_carinii_HdaH     .N...........SFD..IY.....................................QSVR...Y.I.
1f_Malassezia_pachydermatis_H    .G.............E.K.LAATKPKSDDDVAAMLAGMSIAERESVLRVFTTTRTLRLSEPSVALADVHA.H...E.S.
1f_Saccharomyces_cerevisiae_H    .Y.............P.S.LF....................................TL.K....S..
1g_Homo_sapiens_HDAC4            .R.............G..KC.....................................EC.I...R.G.
1g_Saccharomyces_cerevisiae_H    .INDPTLSGVDDLGD..LM......................................LK.I...P.V.
1g_Arabidopsis_thaliana_HDA5     GV.............S.QRC.....................................VV.L....GS.


                                                                      α4
1a_Arabidopsis_thaliana_HDA14               .............................................
                                          120

1a_Arabidopsis_thaliana_HDA14    ...NFKTAT.VE..............................................DIANVHDKA
1a_Candidatus_Hinthialibacter    .....AEEE.................................................FLEKVHR..
1a_Actinobacteria_bacterium_R    .....EA.P.LE..............................................ALYLVHRPA
1b_Arabidopsis_thaliana_HDA8     .....LPAI.VS..............................................ELLMFHTSE
1b_Klebsiella_pneumoniae_HdaH    .....APAS.LE..............................................DLRRIHPDS
1b_Vibrio_sp_vnigr-6D03_HdaH     .....PSCS.QA..............................................QLLRVHPEH
1c_Shinella_sp_DD12_HdaH         .....PQAN.ED..............................................FVLLAHPER
1c_Haematospirillum_jordaniae    .....PRAT.LE..............................................QLTRVHPQS
1c_Micavibrio_sp_TMED2_Acu       PRGPVPKAS.RE..............................................QLERVHEPV
1d_Legionella_londiniensis_Ap    .....HKAT.RA..............................................QLERVHDKQ
1d_Thioploca_ingrica_DAC         .....PLAT.RA..............................................QLTRVHDSE
1d_Neisseria_zoodegmatis_DAC     .....EVSDSQL..............................................AL..IHPRK
1e_Stentor_coeruleus_HdaH        .....NPAH.IS..............................................DILRVHEYS
1e_Chlorella_variabilis_HdaH     .....QRAA.IG..............................................DVLRVHDWN
1e_Babesia_microti__strain_RI    .....PPAC.LA..............................................DILRVHDIG
1f_Pneumycystis_carinii_HdaH     .....SIIN.NP..............................................IIEFIHGEK
1f_Malassezia_pachydermatis_H    .....EPAT.YDLTTAYAPTSAATETASAKHCERISQLAAKAPSHPPGDVPRYSSLDGALSDASSSDGEGDERMHESE
1f_Saccharomyces_cerevisiae_H    SHQRKGSLM.AP..............................................HVLKVHGSS
1g_Homo_sapiens_HDAC4            .....RKAT.LE..............................................ELQTVHSEA
1g_Saccharomyces_cerevisiae_H    .....RAAT.SE..............................................EILEVHTKE
1g_Arabidopsis_thaliana_HDA5     .....SKAE.DK..............................................HLQLVHTKD
```

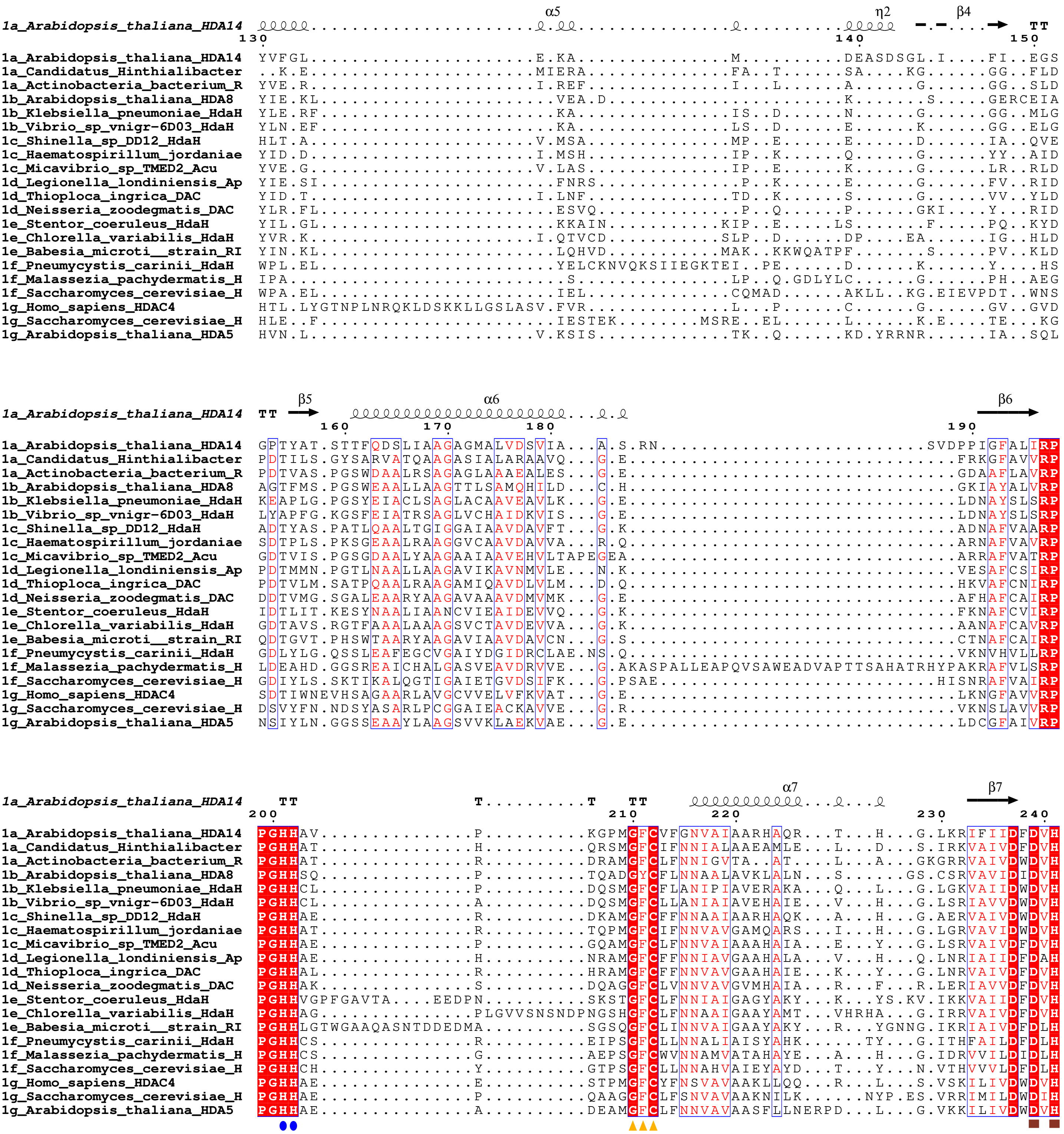
α5
η2
β4
1a_Arabidopsis_thaliana_HDA14
130
140
150
1a_Arabidopsis_thaliana_HDA14 YVFGL......................E.KA...............M.........DEASDSGL.I....FI..EGS
1a_Candidatus_Hinthialibacter ..K.E......................MIERA..............FA..T.......SA....KG.....GG..FLD
1a_Actinobacteria_bacterium_R YVE.R......................I.REF..............I...L......A.....G.....GG..SLD
1b_Arabidopsis_thaliana_HDA8 YIE.KL.......................VEA.D...............K.......S.....GERCEIA
1b_Klebsiella_pneumoniae_HdaH YLE.RF.......................KA...............IS..D.....N.....G.....GG..MLG
1b_Vibrio_sp_vnigr-6D03_HdaH YLN.EF.......................KA...............LS..D.....E.....K.....GG..ELG
1c_Shinella_sp_DD12_HdaH HLT.A......................V.MSA..............MP..E.....E.....D.....IA..QVE
1c_Haematospirillum_jordaniae YID.D......................I.MSH..............IP..K.....Q.....G.....YY..AID
1c_Micavibrio_sp_TMED2_Acu YVE.G......................V.LAS..............IP..E.....K.....G.....LR..RLD
1d_Legionella_londiniensis_Ap YIE.SI.......................FNRS...............P..K.....E.....G.....FV..RID
1d_Thioploca_ingrica_DAC YID.T......................I.LNF..............TD..K.....S.....G.....VV..YLD
1d_Neisseria_zoodegmatis_DAC YLR.FL.......................ESVQ...............P..Q.....P.....GKI....Y..RID
1e_Stentor_coeruleus_HdaH YIL.GL.......................KKAIN............KIP..E....LS......F.....PQ..KYD
1e_Chlorella_variabilis_HdaH YVR.K......................I.QTVCD............SLP..D.....DP....EA.....IG..HLD
1e_Babesia_microti__strain_RI YIN.KL.......................LQHVD............MAK..KKWQATPF.....S.....PV..KLD
1f_Pneumycystis_carinii_HdaH WPL.EL.......................YELCKNVQKSIIEGKTEI..PE.....D.....K.....Y...HS
1f_Malassezia_pachydermatis_H IPA...........................S...............LP..Q.GDLYLC.....G.....PH..AEG
1f_Saccharomyces_cerevisiae_H WPA.EL.......................IEL..............CQMAD.....AKLL..KG.EIEVPDT..WNS
1g_Homo_sapiens_HDAC4 HTL.LYGTNPLNRQKLDSKKLLGSLASV.FVR..............L...P.....C.....G.....GV..GVD
1g_Saccharomyces_cerevisiae_H HLE..F.......................IESTEK........MSRE..EL.....L.....K.E....TE..KG
1g_Arabidopsis_thaliana_HDA5 HVN.L......................V.KSIS..............TK..Q.....KD.YRRNR.....IA..SQL
β5
α6
β6
1a_Arabidopsis_thaliana_HDA14 TT
160
170
180
190
1a_Arabidopsis_thaliana_HDA14 GPTYAT.STTFQDSLIAAGAGMALVDSVIA...A.S.RN.....................SVDPPIGFALIRP
1a_Candidatus_Hinthialibacter PDTILS.GYSARVATQAAGASIALARAAVQ...G.E...........................FRKGFAVVRP
1a_Actinobacteria_bacterium_R PDTVAS.PGSWDAALRSAGAGLAAAEALES...G.E...........................GDAAFLAVRP
1b_Arabidopsis_thaliana_HDA8 AGTFMS.PGSWEAALLAAGTTLSAMQHILD...C.H...........................GKIAYALVRP
1b_Klebsiella_pneumoniae_HdaH KEAPLG.PGSYEIACLSAGLACAAVEAVLK...G.E...........................LDNAYSLSRP
1b_Vibrio_sp_vnigr-6D03_HdaH LYAPFG.KGSFEIATRSAGLVCHAIDKVIS...G.E...........................LDNAYSLSRP
1c_Shinella_sp_DD12_HdaH ADTYAS.PATLQAALTGIGGAIAAVDAVFT...G.K...........................ADNAFVAARP
1c_Haematospirillum_jordaniae SDTPLS.PKSGEAALRAAGGVCAAVDAVVA...R.Q...........................ARNAFVAVRP
1c_Micavibrio_sp_TMED2_Acu GDTVIS.PGSGDAALYAAGAAIAAVEHVLTAPEGEA...........................ARRAFVATRP
1d_Legionella_londiniensis_Ap PDTMMN.PGTLNAALLAAGAVIKAVNMVLE...N.K...........................VESAFCSIRP
1d_Thioploca_ingrica_DAC PDTVLM.SATPQAALRAAGAMIQAVDLVLM...D.Q...........................HKVAFCNIRP
1d_Neisseria_zoodegmatis_DAC DDTVMG.SGALEAARYAAGAVAAAVDMVMK...G.E...........................AFHAFCAIRP
1e_Stentor_coeruleus_HdaH IDTLIT.KESYNAALIAANCVIEAIDEVVQ...G.K...........................FKNAFCVIRP
1e_Chlorella_variabilis_HdaH GDTAVS.RGTFAAALAAGSVCTAVDEVVA...G.K...........................AANAFCAVRP
1e_Babesia_microti__strain_RI QDTGVT.PHSWTAARYAAGAVIAAVDAVCN...G.S...........................CTNAFCAIRP
1f_Pneumycystis_carinii_HdaH GDLYLG.QSSLEAFEGCVGAIYDGIDRCLAE.NS.Q...........................VKNVHVLLRP
1f_Malassezia_pachydermatis_H LDEAHD.GGSREAICHALGASVEAVDRVVE...G.AKASPALLEAPQVSAWEADVAPTTSAHATRHYPAKRAFVLSRP
1f_Saccharomyces_cerevisiae_H GDIYLS.SKTIKALQGTIGAIETGVDSIFK...G.PSAE.......................HISNRAFVAIRP
1g_Homo_sapiens_HDAC4 SDTIWNEVHSAGAARLAVGCVVELVFKVAT...G.E...........................LKNGFAVVRP
1g_Saccharomyces_cerevisiae_H DSVYFN.NDSYASARLPCGGAIEACKAVVE...G.R...........................VKNSLAVVRP
1g_Arabidopsis_thaliana_HDA5 NSIYLN.GGSSEAAYLAAGSVVKLAEKVAE...G.E...........................LDCGFAIVRP
α7
β7
1a_Arabidopsis_thaliana_HDA14 TT T.........T TT
200
210
220
230
240
1a_Arabidopsis_thaliana_HDA14 PGHHAV.............P.........KGPMGFCVFGNVAIAARHAQR...T...H...G.LKRIFIIDFDVH
1a_Candidatus_Hinthialibacter PGHHAT.............H.........QRSMGFCIFNNIALAAEAMLE...L...D...S.IRKVAIVDFDVH
1a_Actinobacteria_bacterium_R PGHHAT.............R.........DRAMGFCLFNNIGVTA..AT....L...A...GKGRRVAIVDWDVH
1b_Arabidopsis_thaliana_HDA8 PGHHSQ.............P.........TQADGYCFLNNAALAVKLALN...S......GS.CSRVAVIDIDVH
1b_Klebsiella_pneumoniae_HdaH PGHHCL.............P.........DQSMGFCFLANIPIAVERAKA...Q...L...G.LGKVAIIDWDVH
1b_Vibrio_sp_vnigr-6D03_HdaH PGHHCL.............A.........DQSMGFCLLANIAIAVEHAIE...V...H...G.LSRIAVVDWDVH
1c_Shinella_sp_DD12_HdaH PGHHAE.............R.........DKAMGFCFFNNAAIAARHAQK...A...H...G.AERVAIVDWDVH
1c_Haematospirillum_jordaniae PGHHAT.............R.........TQPMGFCIFNNVAVGAMQARS...I...H...G.LGRVAVIDWDVH
1c_Micavibrio_sp_TMED2_Acu PGHHAE.............P.........GQAMGFCLFNNVAIAAAHAIA...E...Y...G.LSRVAIVDFDVH
1d_Legionella_londiniensis_Ap PGHHAE.............H.........NRAMGFCFFNNIAVGAAHALA...V...H...Q.LNRIAIIDFDAH
1d_Thioploca_ingrica_DAC PGHHAL.............R.........HRAMGFCFFNNVAVGAAHAIE...K...Y...G.LNRVAIVDFDVH
1d_Neisseria_zoodegmatis_DAC PGHHAK.............S.........DQAGGFCLVNNVAVGVMHAIA...R...F...R.LERIAVVDFDVH
1e_Stentor_coeruleus_HdaH PGHHVGPFGAVTA....EEDPN.........SKSTGFCLFNNIAIGAGYAKY...K...YS.KV.IKKVAIIDFDVH
1e_Chlorella_variabilis_HdaH PGHHAG..............PLGVVSNSNDPNGSHGFCLFNNAAIGAAYAMT...VHRHA...G.IRRVAIVDFDVH
1e_Babesia_microti__strain_RI PGHHLGTWGAAQASNTDDEDMA.........SGSQGFCLINNVAIGAAYAKY...R...YGNNG.IKRIAIVDFDLH
1f_Pneumycystis_carinii_HdaH PGHHCS.............R.........EIPSGFCLLNNALIAISYAHK.....TY...G.ITHFAILDFDLH
1f_Malassezia_pachydermatis_H PGHHCS.............G.........AEPSGFCWVNNAMVATAHAYE...A...H...G.IDRVVILDIDLH
1f_Saccharomyces_cerevisiae_H PGHHCH.............Y.........GTPSGFCLLNNAHVAIEYAYD...T...Y...N.VTHVVVLDFDLH
1g_Homo_sapiens_HDAC4 PGHHAE.............E.........STPMGFCYFNSVAVAAKLLQQ...R...L...S.VSKILIVDWDVH
1g_Saccharomyces_cerevisiae_H PGHHAE.............P.........QAAGGFCLFSNVAVAAKNILK......NYP.ES.VRRIMILDWDIH
1g_Arabidopsis_thaliana_HDA5 PGHHAE.............A.........DEAMGFCLFNNVAVAASFLLNERPD...L...G.VKKILIVDWDVH

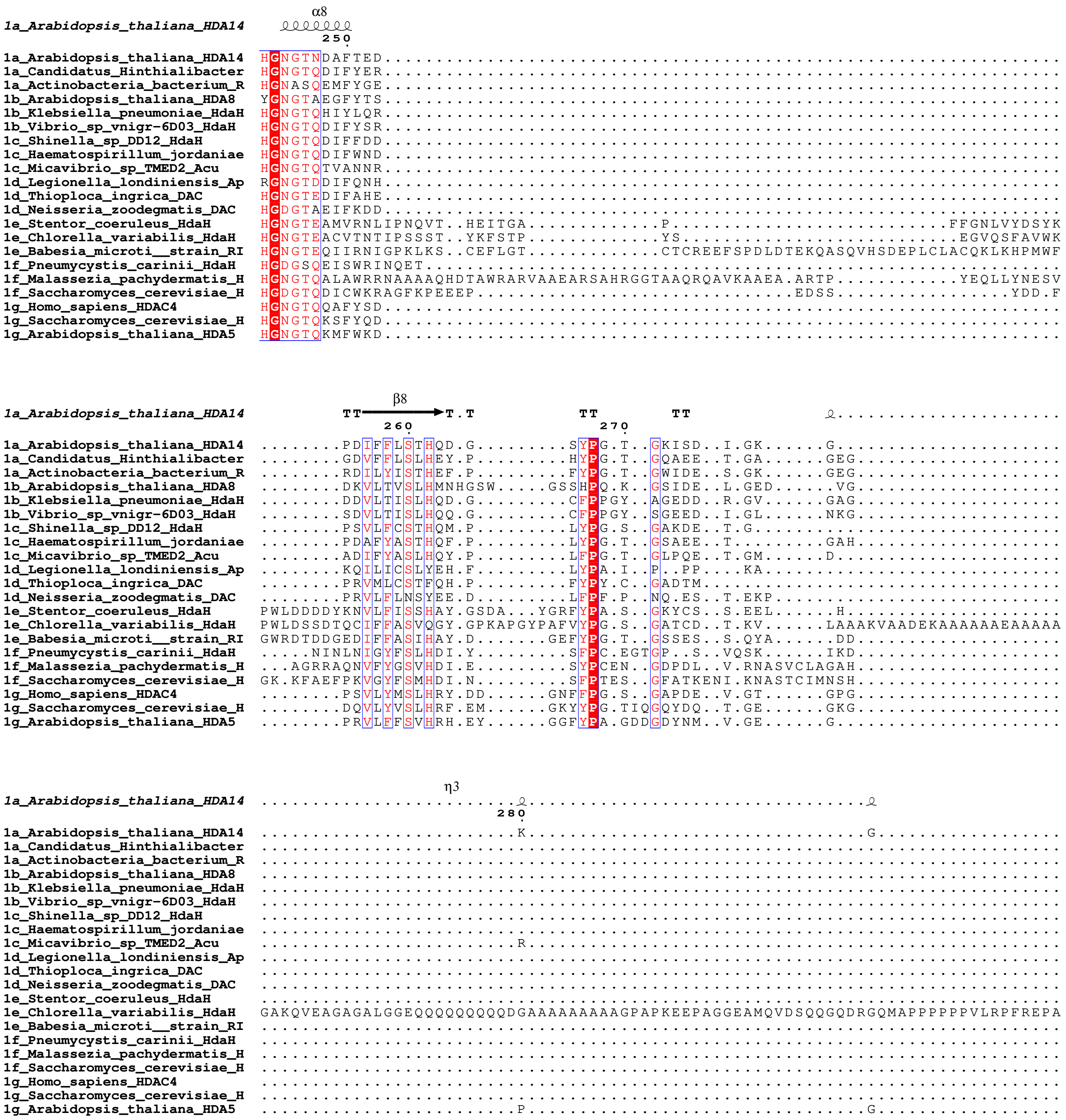
α8
β8
η3
250
260
270
280
1a_Arabidopsis_thaliana_HDA14
1a_Candidatus_Hinthialibacter
1a_Actinobacteria_bacterium_R
1b_Arabidopsis_thaliana_HDA8
1b_Klebsiella_pneumoniae_HdaH
1b_Vibrio_sp_vnigr-6D03_HdaH
1c_Shinella_sp_DD12_HdaH
1c_Haematospirillum_jordaniae
1c_Micavibrio_sp_TMED2_Acu
1d_Legionella_londiniensis_Ap
1d_Thioploca_ingrica_DAC
1d_Neisseria_zoodegmatis_DAC
1e_Stentor_coeruleus_HdaH
1e_Chlorella_variabilis_HdaH
1e_Babesia_microti__strain_RI
1f_Pneumycystis_carinii_HdaH
1f_Malassezia_pachydermatis_H
1f_Saccharomyces_cerevisiae_H
1g_Homo_sapiens_HDAC4
1g_Saccharomyces_cerevisiae_H
1g_Arabidopsis_thaliana_HDA5

```
1a_Arabidopsis_thaliana_HDA14                                                              β9              α9
                                                                                     ━━━━━━━....▶  TT  ℓℓℓℓℓℓℓℓℓℓ ℓℓ
                                                                                              290       300
1a_Arabidopsis_thaliana_HDA14      ...................................E.......G.T.TLNLP....LP.GG.SGDIAMRTVFEEIIV
1a_Candidatus_Hinthialibacter      ...........................................Q.GTTLNVP....LPP.G.IDQETWFEAFHKG.L
1a_Actinobacteria_bacterium_R      ...................................E.......GFN.I.NIP....LP.AG.TAGEVYEEAWVRLVV
1b_Arabidopsis_thaliana_HDA8       ..........................................LG.Y.NLNVP....LP.NG.TGDRGYEYAMNELVV
1b_Klebsiella_pneumoniae_HdaH      ...................................E.......G.Y.NINIP....LL.AG.AGDDSWRYALETIVI
1b_Vibrio_sp_vnigr-6D03_HdaH       ...................................K.......G.S.NINIP....LM.PG.GGHNSYLNAMEKIVI
1c_Shinella_sp_DD12_HdaH           ...................................A.......G.N.IVNAP....LS.MN.DGSDHFRDAFRSRVL
1c_Haematospirillum_jordaniae      ...................................N.........N.IVNCP....LP.SG.SDGTVFMEALDNRII
1c_Micavibrio_sp_TMED2_Acu         .............................................N.VLNIL....MR.ES.STGAEYRRAFDETLL
1d_Legionella_londiniensis_Ap      ...........................................D.N.IIDLP....LP.AR.ATGSQLRAKFSQKVI
1d_Thioploca_ingrica_DAC           ...................................S.......N.H.IINVP....LA.AG.TGGDKFREAVLMHWI
1d_Neisseria_zoodegmatis_DAC       ..........................................SP.Y.AVNIP....LA.PN.SSSRDFRETVRKHWL
1e_Stentor_coeruleus_HdaH          ..........................................PA.G.ILNIP....LS.KC.TDSSAFRQQYREKVF
1e_Chlorella_variabilis_HdaH       NGAVAGVAEVGRDEEIEEDPDREFEYKGGQVPPLEG.......P.R.VIDVG....IPGFG.SKPALWRRSWRDKIL
1e_Babesia_microti__strain_RI      ..........................................GP.R.IINVG....VP.KN.TTSADFRTLFETNIC
1f_Pneumycystis_carinii_HdaH       ..................................ASICIMNHNQ.A.IWNVH....IE.KY.TTYEEFEELYNTSYI
1f_Malassezia_pachydermatis_H      ...................................G.......Q.W.IWNVH....LD.TH.RDDADFDEVYRTKYA
1f_Saccharomyces_cerevisiae_H      ...................................D.......L.N.IWNIH....LSK.W.TTEEEFNVLYRTKYR
1g_Homo_sapiens_HDAC4              ...................................V.......G.F.NVNMAFTGGLD.PP.MGDAEYLAAFRTVVM
1g_Saccharomyces_cerevisiae_H      ...................................E.......G.F.NCNIT....WP.VGGVGDAEYMWAFEQVVM
1g_Arabidopsis_thaliana_HDA5       ...................................E.......G.F.NINVP....WDQGR.CGDADYLAAWDHILI
```

```
                                                   α10                        β10                          α11
1a_Arabidopsis_thaliana_HDA14      ...............ℓℓℓℓℓ...........ℓ          ━━━━━▶  TT TT          ℓℓℓℓℓℓℓ...ℓℓℓ
                                                  310                 320        330            340
1a_Arabidopsis_thaliana_HDA14      ...............PCAQR...........FK..PDIILVSAGYDAHVLDPL..AN....LQFTTATYYSL...AKD
1a_Candidatus_Hinthialibacter      ...............EAISQ...........YN..PDAILVSAGFDSHRRDPL...G.D..F...PLDEKAYARIGGL
1a_Actinobacteria_bacterium_R      ...............PVLQE...........MR..PDWILVSAGFDGHADDPL..AN....IRLREPDYGRM...ACR
1b_Arabidopsis_thaliana_HDA8       ...............PAVRR...........FG..PDMVVLVVGQDSSAFDPN..G..R..QSLTMNGYRRI...GQI
1b_Klebsiella_pneumoniae_HdaH      ...............PALAR...........FE..PELIIIACGYDANAMDPL..AR....MQLHSDSFRAM...TEQ
1b_Vibrio_sp_vnigr-6D03_HdaH       ...............PALDA...........YK..PELILVASGYDACGVDPL..AR....MLAHSETYRTM...TQM
1c_Shinella_sp_DD12_HdaH           ...............PRVAD...........FR..PDVIIISAGFDAHHRDPL..AQ....INLVADDFDWA...TAK
1c_Haematospirillum_jordaniae      ...............PALKN...........FR..PHILFISAGFDAHGRDPL..AN....MKLTVKDYAEA...TRK
1c_Micavibrio_sp_TMED2_Acu         ...............PALEE...........YL..PELLIISAGFDAHERDPL..AG....IMLTDEDFGWM...TRR
1d_Legionella_londiniensis_Ap      ...............HAIEA...........FN..PELILISAGFDGHADDAI..S..T..LRFIESDYHWL...TQQ
1d_Thioploca_ingrica_DAC           ...............PNLYK...........FA..PQLILISAGFDAHAEDDM..SQ....LRLRENDYAWV...TRE
1d_Neisseria_zoodegmatis_DAC       ...............PKLLA...........FQ..PELVLLSAGFDGHKSDET..G..R..LNLHEADYAWL...THK
1e_Stentor_coeruleus_HdaH          ...............PRLIE...........FA..PDLLLISAGFDAHGLDSI..NHGF..VDLDEDDFRWV...TEE
1e_Chlorella_variabilis_HdaH       ...............PSLSN...........FK..PDLIFVCAGFDAHKKDEI..NFRY..IGVTERDYEWL...TEQ
1e_Babesia_microti__strain_RI      ...............PYLLH...........FS..PDLIFISAGFDGHYRDSV..NVGF..THYRDRDFFYV...TER
1f_Pneumycystis_carinii_HdaH       ...............NLLKKADFFFESTKALPE..QSLLVISAGFDSSEFESE..HMQRHGYHLPALFYWKF...SKD
1f_Malassezia_pachydermatis_H      QLFAQARRF.....LRETHA...........VP.PRTLVVISCGFDACTYEYP..GMQRHGKHVPPHFYARF...AQD
1f_Saccharomyces_cerevisiae_H      TLFAKADEFFRSAKLEMNQQ...........GRPFKGLVVISAGFDASEFEQT..SMQRHSVNVPTSFYTTF...TKD
1g_Homo_sapiens_HDAC4              ...............PIASE...........FA..PDVVLVSSGFDAVEGHPTPLGG...YNLSARCFGYL...TKQ
1g_Saccharomyces_cerevisiae_H      ...............PMGRE...........FK..PDLVIISSGFDAADGDTI..G..Q..CHVTPSCYGHM...THM
1g_Arabidopsis_thaliana_HDA5       ...............PVARE...........FN..PDVIFLSAGFDAAINDPL..GG...CCVTPYGYSVM...LKK
```

```
                                             β11                      α12                     η4         η5
1a_Arabidopsis_thaliana_HDA14      ℓℓℓℓℓℓℓℓℓ    ━━━━▶      ℓℓℓℓℓℓℓ.....ℓ.ℓℓℓℓℓℓℓℓ      ℓℓℓ     ℓ.ℓ..........ℓ
                                  350        360       370              380       390         400
1a_Arabidopsis_thaliana_HDA14      IKRLAKEVCGGRCVFFLEGGYNLESLSSS....V.ADSFRALLGEDSL.ASEFDNP.A.........Y..LYDE..
1a_Candidatus_Hinthialibacter      LADLAG...EHPLISILEGGYNVDVLGSS....V.VAYIDGLRGR..............................
1a_Actinobacteria_bacterium_R      LARLA....PSRLIFFLEGGYDLNAISSS....VKVAM.DGVGGE......FPDL.T.........G..D..E..
1b_Arabidopsis_thaliana_HDA8       MRGVAEEHSHGRLLMVQEGGYHVTYAAYCLHA.....MLEGVLKIPE..PHLSDPI.A.........YYPE.E....
1b_Klebsiella_pneumoniae_HdaH      VQQAADRLCGGKLVMVHEGGYAESYVPFC....G.LAVMEALSGIRTE...VQDPL.L.........EFIQQQQP..
1b_Vibrio_sp_vnigr-6D03_HdaH       IMEVSGRHANNKLVIVHEGGYSEAYVPFC....G.LAVIETLCGVKTG...IEDPM.L.........ELIQAQQP..
1c_Shinella_sp_DD12_HdaH           LMELSEKSANNRVVSLLEGGYDLTGLAES....A.ATHIYRLMRG..............................
1c_Haematospirillum_jordaniae      LMAVADSLCGGRIISVLEGGYDLIATASS....A.AAHIRV..................................
1c_Micavibrio_sp_TMED2_Acu         LREVAEAHAKGRLVSVLEGGYDLHALASG....V.RAHLSELI................................
1d_Legionella_londiniensis_Ap      IKTIARKSCAGRLVSVLEGGYALNVLGAC....VE.THLEALK................................
1d_Thioploca_ingrica_DAC           ILVIADKYAQGRVISTLEGGYELQSLGRS....V.AAHLKVLMRMD.............................
1d_Neisseria_zoodegmatis_DAC       LIQTASS.CKGKIVSVLEGGYTLDSLAKS....SA.AHLYVLAGLGKPECAVIYDK...................
1e_Stentor_coeruleus_HdaH          LIKIANTSCKGKIVSVLEGGYSVRGNVVAALGLSVA.SHIRALIRAT...QEHFCGSEG........ND.......
1e_Chlorella_variabilis_HdaH       LVAVANRCCQGRIVS...GGI.MSAFARS....V.AAHVKALAEPNAT..VWDPR.E.........AK..AERE..
1e_Babesia_microti__strain_RI      LQSIANATCGGKVVSVLEGGYNTRLNSSSPFAQSVL.QHVIALAQTN...KGMVYPL.M.........FPLS.TV...
1f_Pneumycystis_carinii_HdaH       TLLLSQKRCCSRIFSIMEGGYSDRAIASG....TF.TLLCGLTNLTQE..QMDQCK.S.........LWKM.EYFSW
1f_Malassezia_pachydermatis_H      AAALADEYAEGKLISLLEGGYSDRALTSG....T.LAHIGALAALPWA..HTAWPR.A.........QAPW.RVE..
1f_Saccharomyces_cerevisiae_H      ALKLAQMHCHGKVLSLMEGGYSDKAICSG....V.FAHLIGLQNQDWV..KEWGSE.QVVKEIVRGCKPAW.K.P..
1g_Homo_sapiens_HDAC4              LMGLA....GGRIVLALEGGHDLTAICDA....S.EACVSALLGNELD..P.LPEK.V.........L..QQRP..
1g_Saccharomyces_cerevisiae_H      LKSLA....RGNLCVVLEGGYNLDAIARS....AL.SVAKVLIGEPPD..ELPDPL.S.........DPKP.EVIEM
1g_Arabidopsis_thaliana_HDA5       LMEFA....QGKIVLALEGGYNLDSIAKS....S.LACVQVLLEDKQI...QGPPE.A.........Y.....P..
```

```
                                                α13
1a_Arabidopsis_thaliana_HDA14                          QQQQQQQQQQQQ.QQQQ
                                                        410       420
                                                          .         .
1a_Arabidopsis_thaliana_HDA14   .........................PMRKVRDAIQRA.KSIHCL..................................
1a_Candidatus_Hinthialibacter   ..............................................................................
1a_Actinobacteria_bacterium_R   .........................SPPRARH.MVQVSLAQAAKYWDV.........G....................
1b_Arabidopsis_thaliana_HDA8    ..............................................................................
1b_Klebsiella_pneumoniae_HdaH   .........................RA..TFA.QFQR.QA......................................
1b_Vibrio_sp_vnigr-6D03_HdaH    .........................CQ..SFN.QLQA.EK......................................
1c_Shinella_sp_DD12_HdaH        ..............................................................................
1c_Haematospirillum_jordaniae   ......................................LMEN....................................
1c_Micavibrio_sp_TMED2_Acu      ..............................................................................
1d_Legionella_londiniensis_Ap   ..............................................................................
1d_Thioploca_ingrica_DAC        ..............................................................................
1d_Neisseria_zoodegmatis_DAC    ..............................................................................
1e_Stentor_coeruleus_HdaH       ..............................................................................
1e_Chlorella_variabilis_HdaH    .........................HEKKVRA.ELEA.KKAEAAHQKA..............................
1e_Babesia_microti__strain_RI   .............................KNL.MDSI.SVLE.GILR...............................
1f_Pneumycystis_carinii_HdaH    IDKSI.........................................................................
1f_Malassezia_pachydermatis_H   .........................NL..MQL.ERMA.KR....VM.A.........EA...................
1f_Saccharomyces_cerevisiae_H   .........................Y.KTKRA.KDVI..RIWAEEVIRL........GRAMI................
1g_Homo_sapiens_HDAC4           .........................NANAVRS.MEKV.MEIHSKYWRC.........LQ...................
1g_Saccharomyces_cerevisiae_H   IDKVIRLQSKYWNCFRRRHANSGCNFNEPIND.SIIS.KNFPLQKAIR.........QQ...........QQHYLSD.
1g_Arabidopsis_thaliana_HDA5    .........................FESTWRV.IQAV.RKRLCTYWPSLADELSWKLINQKTPTPIILISSS...DSE

1a_Arabidopsis_thaliana_HDA14

1a_Arabidopsis_thaliana_HDA14   ..............................................................................
1a_Candidatus_Hinthialibacter   ..............................................................................
1a_Actinobacteria_bacterium_R   ..............................................................................
1b_Arabidopsis_thaliana_HDA8    ..............................................................................
1b_Klebsiella_pneumoniae_HdaH   ..............................................................................
1b_Vibrio_sp_vnigr-6D03_HdaH    ..............................................................................
1c_Shinella_sp_DD12_HdaH        ..............................................................................
1c_Haematospirillum_jordaniae   ..............................................................................
1c_Micavibrio_sp_TMED2_Acu      ..............................................................................
1d_Legionella_londiniensis_Ap   ..............................................................................
1d_Thioploca_ingrica_DAC        ..............................................................................
1d_Neisseria_zoodegmatis_DAC    ............................................................................FL
1e_Stentor_coeruleus_HdaH       ...NEK....MILKSSVNESFRALKRNRKMYIE.N........................................LEV
1e_Chlorella_variabilis_HdaH    ..............................................................................
1e_Babesia_microti__strain_RI   ...CDK....GIVEKANFD.......LPSSHLY.S........................................MD.
1f_Pneumycystis_carinii_HdaH    ............SKGTISR.......LPKNALL.N........................................IQI
1f_Malassezia_pachydermatis_H   .................................AA........................................HAT
1f_Saccharomyces_cerevisiae_H   .PEFDD....IIFKDAVNS.......APSNSLL.K........................................ATV
1g_Homo_sapiens_HDAC4           ..............................................................................
1g_Saccharomyces_cerevisiae_H   ..EFNF....VTLPLVSMD.......LPDNTVL.CTPNISESNTIIIVVHDTSDIWAKRNVISGTIDLSSSVIIDNSL
1g_Arabidopsis_thaliana_HDA5    TEDNAQGLLDQMSKLSIEN........PQGTLLEN........................................HQV

1a_Arabidopsis_thaliana_HDA14

1a_Arabidopsis_thaliana_HDA14   ..............................................................................
1a_Candidatus_Hinthialibacter   ..............................................................................
1a_Actinobacteria_bacterium_R   ..............................................................................
1b_Arabidopsis_thaliana_HDA8    ..........................E.ANAVAAVES.IKTYHTEFVPFLRGT.........................
1b_Klebsiella_pneumoniae_HdaH   ......................................I.......................................
1b_Vibrio_sp_vnigr-6D03_HdaH    ......................................I.......................................
1c_Shinella_sp_DD12_HdaH        ..............................................................................
1c_Haematospirillum_jordaniae   ..............................................................................
1c_Micavibrio_sp_TMED2_Acu      ..............................................................................
1d_Legionella_londiniensis_Ap   ..............................................................................
1d_Thioploca_ingrica_DAC        ..............................................................................
1d_Neisseria_zoodegmatis_DAC    KSEHA.........................................................................
1e_Stentor_coeruleus_HdaH       SMENQLTAEVAE.......EFNGEK...TQEVVSEESGGDEDQDENSEDESGN.........................
1e_Chlorella_variabilis_HdaH    .AA...................................REAYVK.........ARLAA....VAA.AAA.........
1e_Babesia_microti__strain_RI   DMTNQIS.ELVE.......SIYSKKCCISHILRSEDR.VDTINSEYNPFYSGNAWYSFFCHTFQPIECD.........
1f_Pneumycystis_carinii_HdaH    DWI................................KN.IIQMAQ.........KYTTI....L...............
1f_Malassezia_pachydermatis_H   QTA.......SATRPRRTTQYQ.............P..WIIRAS.........EYFAA....FQQ.ACG.........
1f_Saccharomyces_cerevisiae_H   EPA.......ST.......STI.............A..Q.......................................
1g_Homo_sapiens_HDAC4           ......................................RTTSTA.........GRSLI....EAQ.TCE.........
1g_Saccharomyces_cerevisiae_H   DFI................................KW.G..LDR.........KYGI.....I...............
1g_Arabidopsis_thaliana_HDA5    EPA.......ST.......SWR.............A..DLAKVD.........VWYAS....FGS.NMWKPRFLCYIQ
```

1a_Arabidopsis_thaliana_HDA14

```
1a_Arabidopsis_thaliana_HDA14   ..............................................................................
1a_Candidatus_Hinthialibacter   ..............................................................................
1a_Actinobacteria_bacterium_R   ..............................................................................
1b_Arabidopsis_thaliana_HDA8    ..............................................................................
1b_Klebsiella_pneumoniae_HdaH   ..............................................................................
1b_Vibrio_sp_vnigr-6D03_HdaH    ..............................................................................
1c_Shinella_sp_DD12_HdaH        ..............................................................................
1c_Haematospirillum_jordaniae   ..............................................................................
1c_Micavibrio_sp_TMED2_Acu      ..............................................................................
1d_Legionella_londiniensis_Ap   ..............................................................................
1d_Thioploca_ingrica_DAC        ..............................................................................
1d_Neisseria_zoodegmatis_DAC    ..............................................................................
1e_Stentor_coeruleus_HdaH       ..............................................................................
1e_Chlorella_variabilis_HdaH    ...APAAPAGTAPAAAGTSAPA.ASPPT..................................................
1e_Babesia_microti__strain_RI   ...DDIRQNNN......SPSPM.CI..........P..........................................
1f_Pneumycystis_carinii_HdaH    .........ET......KTDLI.L.........S............................................
1f_Malassezia_pachydermatis_H   ...IDARPLTL......TPSAT.TT..........PRRAVL.....................................
1f_Saccharomyces_cerevisiae_H   ........RII......RSHRSNASPEK.ELHENKPRSTE........................KQEQREIR....SD
1g_Homo_sapiens_HDAC4           ..............................................................................
1g_Saccharomyces_cerevisiae_H   .........DV......NIPLT.L.........F....................................EPDNYSGM
1g_Arabidopsis_thaliana_HDA5    GGQVDGLKKVC......VGSMD.KSPPKETVWETFPHRLFFGRESSVGWGVGGVAFTNPLANLID.QTHMCLYRITLE
```

1a_Arabidopsis_thaliana_HDA14

```
1a_Arabidopsis_thaliana_HDA14   ..............................................................................
1a_Candidatus_Hinthialibacter   ..............................................................................
1a_Actinobacteria_bacterium_R   ..............................................................................
1b_Arabidopsis_thaliana_HDA8    ..............................................................................
1b_Klebsiella_pneumoniae_HdaH   ..............................................................................
1b_Vibrio_sp_vnigr-6D03_HdaH    ..............................................................................
1c_Shinella_sp_DD12_HdaH        ..............................................................................
1c_Haematospirillum_jordaniae   ..............................................................................
1c_Micavibrio_sp_TMED2_Acu      ..............................................................................
1d_Legionella_londiniensis_Ap   ..............................................................................
1d_Thioploca_ingrica_DAC        ..............................................................................
1d_Neisseria_zoodegmatis_DAC    ..............................................................................
1e_Stentor_coeruleus_HdaH       .............................................................................E
1e_Chlorella_variabilis_HdaH    ..............................................................................
1e_Babesia_microti__strain_RI   ............................................FD.........................IDSSTIN
1f_Pneumycystis_carinii_HdaH    ................................EDR.........MTL...RERK...............KPLDSSLIS
1f_Malassezia_pachydermatis_H   ..........................................RGLDL...DLAT...............PSRVTAGGH
1f_Saccharomyces_cerevisiae_H   T.................................KVKQLSSNNR.AA...ETQIPFLQQEFSSEDEDEEYVYDEELNK
1g_Homo_sapiens_HDAC4           ..................................................NE..........................
1g_Saccharomyces_cerevisiae_H   ITSQEVLIYLWDNYIKYFPSVAKIAFIGIGDSYSG.........IVHLLGHRDT...............RAVTKTVIN
1g_Arabidopsis_thaliana_HDA5    Q.................................FNDVLSQENGLNV...DSDS...............PVFDLAALQ
```

1a_Arabidopsis_thaliana_HDA14

```
1a_Arabidopsis_thaliana_HDA14   ..............................................................................
1a_Candidatus_Hinthialibacter   ..............................................................................
1a_Actinobacteria_bacterium_R   ..............................................................................
1b_Arabidopsis_thaliana_HDA8    ..............................................................................
1b_Klebsiella_pneumoniae_HdaH   ..............................................................................
1b_Vibrio_sp_vnigr-6D03_HdaH    ..............................................................................
1c_Shinella_sp_DD12_HdaH        ..............................................................................
1c_Haematospirillum_jordaniae   ..............................................................................
1c_Micavibrio_sp_TMED2_Acu      ..............................................................................
1d_Legionella_londiniensis_Ap   ..............................................................................
1d_Thioploca_ingrica_DAC        ..............................................................................
1d_Neisseria_zoodegmatis_DAC    ..............................................................................
1e_Stentor_coeruleus_HdaH       SEEG...........EEGEEREDNE.....................................................
1e_Chlorella_variabilis_HdaH    ..............................................................................
1e_Babesia_microti__strain_RI   L.DGNNMHGKFIPLFTDPGYSESQELTHGHDVDWIDNIKFEIM.....KANYTCALLLKEFF..........TRYVHL
1f_Pneumycystis_carinii_HdaH    L..............................IDQIQKIKI......................................
1f_Malassezia_pachydermatis_H   ..................................AL.................................RD..RSV..
1f_Saccharomyces_cerevisiae_H   ............................................................TFNRTV.......EDITI
1g_Homo_sapiens_HDAC4           ..........................................................................EAET
1g_Saccharomyces_cerevisiae_H   F..............................LGDKQLKPLVPLVDETLS..........EW........YFKNSLI..
1g_Arabidopsis_thaliana_HDA5    L..............................VDNKGSILEAPL.....N..........SWYGNVVCLGKE..RDIPI
```

1a_Arabidopsis_thaliana_HDA14

```
1a_Arabidopsis_thaliana_HDA14   ..............................................................................
1a_Candidatus_Hinthialibacter   ..............................................................................
1a_Actinobacteria_bacterium_R   ..............................................................................
1b_Arabidopsis_thaliana_HDA8    ..............................................................................
1b_Klebsiella_pneumoniae_HdaH   ..............................................................................
1b_Vibrio_sp_vnigr-6D03_HdaH    ..............................................................................
1c_Shinella_sp_DD12_HdaH        ..............................................................................
1c_Haematospirillum_jordaniae   ..............................................................................
1c_Micavibrio_sp_TMED2_Acu      ..............................................................................
1d_Legionella_londiniensis_Ap   ..............................................................................
1d_Thioploca_ingrica_DAC        ..............................................................................
1d_Neisseria_zoodegmatis_DAC    ..............................................................................
1e_Stentor_coeruleus_HdaH       .....................E.DDEE.................................................ES
1e_Chlorella_variabilis_HdaH    ..........................A.PAPAPAPAPAAGAEEAEDGGTRRKRRRG.AGSGVDYVEL.......NRQL
1e_Babesia_microti__strain_RI   TSYNCKLHCD...........M......................................................RF
1f_Pneumycystis_carinii_HdaH    ..................................................KPIK.....................SPQ
1f_Malassezia_pachydermatis_H   ..........RRRQSHAAI.....STE.PTPARARASARSRNDGERGTSHDPAQW.........VPG.......ALPV
1f_Saccharomyces_cerevisiae_H   DDISRHLETLEIEKKGDE....DSDHELKEKNWKNSHQRRL................QGNGMYKIPSNTKPHRIRQPQ
1g_Homo_sapiens_HDAC4           VTAM......ASLSVGVKPAEKRPDEE.PM................................................
1g_Saccharomyces_cerevisiae_H   ..........FSNN.........................SHQCWKENESRKPRKKFGRVLRCDTDGLNNII.E..ERF
1g_Arabidopsis_thaliana_HDA5    LTMTCTLSAVEKFKSGEIP.....IRP.PAKAYANTLIRGLVEGG..............................RLS
```

1a_Arabidopsis_thaliana_HDA14

```
1a_Arabidopsis_thaliana_HDA14   ..............................................
1a_Candidatus_Hinthialibacter   ..............................................
1a_Actinobacteria_bacterium_R   ..............................................
1b_Arabidopsis_thaliana_HDA8    ..............................................
1b_Klebsiella_pneumoniae_HdaH   ........................DRLAQQFGLL............
1b_Vibrio_sp_vnigr-6D03_HdaH    ........................DLLAKNTPLLV...........
1c_Shinella_sp_DD12_HdaH        ..............................................
1c_Haematospirillum_jordaniae   ..............................................
1c_Micavibrio_sp_TMED2_Acu      ..............................................
1d_Legionella_londiniensis_Ap   ..............................................
1d_Thioploca_ingrica_DAC        ..............................................
1d_Neisseria_zoodegmatis_DAC    ..............................................
1e_Stentor_coeruleus_HdaH       EEEDSQESKKTDKA.NNGEPE..............DN.........
1e_Chlorella_variabilis_HdaH    EEEA.AARR..AAGGSG..GG.........................
1e_Babesia_microti__strain_RI   DQK............C...................QI.........
1f_Pneumycystis_carinii_HdaH    ...............................TKNTDKHS.......
1f_Malassezia_pachydermatis_H   ESAG.PA.T..PAAPTQ.RPSHDADTLATLLGQWQLNDTT......
1f_Saccharomyces_cerevisiae_H   NANT...............PTYDDSDI.SMISHVSRKHTTRSGGRW
1g_Homo_sapiens_HDAC4           EEEP...............PL.........................
1g_Saccharomyces_cerevisiae_H   EEAT...............DF.....ILDSFEEWSDEE........
1g_Arabidopsis_thaliana_HDA5    KEEA.EAYI..DKA.VS.KPL.........................
```

Supplementary Figure 14: Primary sequence alignment of selected bacterial deacylases of cluster 1 (UniProt accession numbers are shown).

Show are the following sequences of the following deacylases representing enzymes of cluster 1 and the sub-clusters (code: sub-cluster, accession number/protein name/spcies): 1a, AT4G33470.1/HDA14/*Arabidopsis thaliana*; 1a, A0A136MHB6/AphA/*Candidatus Hinthialibacteria bacterium* OLB16; 1a, A0A1F2W7Q1/HdaH/*Actinobacteria bacterium* RBG_16_70_17; 1b, Q94EJ2/HDA8/*Arabidopsis thaliana*; 1b, A0A378F8Q8/HdaH/*Klebsiella pneumoniae*; 1b, A0A2N0XRJ0/HdaH/*Vibrio sp.* vnigr-6D03; 1c, A0A021X4K7/Deacetylase (DAC)/*Shinella sp.* DD12; 1c, A0A143DCS4/AcuC/*Haematospirillum jordaniae*; 1c, A0A1Z8KYS1/Acu protein/*Micavibrio sp.* TMED2; 1d, A0A0W0VIH3/ApaH/*Legionella londiniensis*; 1d, A0A090BV44/Deacetylase/*Thioploca ingrica*; 1d, A0A1X3CVE4/Deacetylase/*Neisseria zoodegmatis*; 1e, A0A1R2CWG0/HdaH/*Stentor coeruleus*; 1e, E1ZJU5/HdaH/*Chlorella variabilis* (green alga); 1e, A0A1N6LWP9/HOS3/*Babesia microti* (strain RI); 1f, A0A0W4ZQY2/HdaH/*Pneumocystis carinii* (strain B80); 1f, A0A0M9VQN5/HdaH/*Malassezia pachydermatis*; 1f, Q02959/HOS3/*Saccharomyces cerevisiae* (strain ATCC 204508 / S288c); 1g, P56524/HDAC4/*Homo sapiens*; 1g, P53973/Hda1/*Saccharomyces cerevisiae* (strain ATCC 204508 / S288c); 1g, AT5G61060.1 (Q8RX28)/HDA5/*Arabidopsis thaliana*. All sequences were aligned using the T-COFFEE multiple sequence alignment server[1]. The important catalytic residues are highlighted. The secondary structure elements and numbering is shown for the deacylase shown at the top of the amino acid sequences above the sequence alignment. The alignment was created by ESPript version 3.0[2].

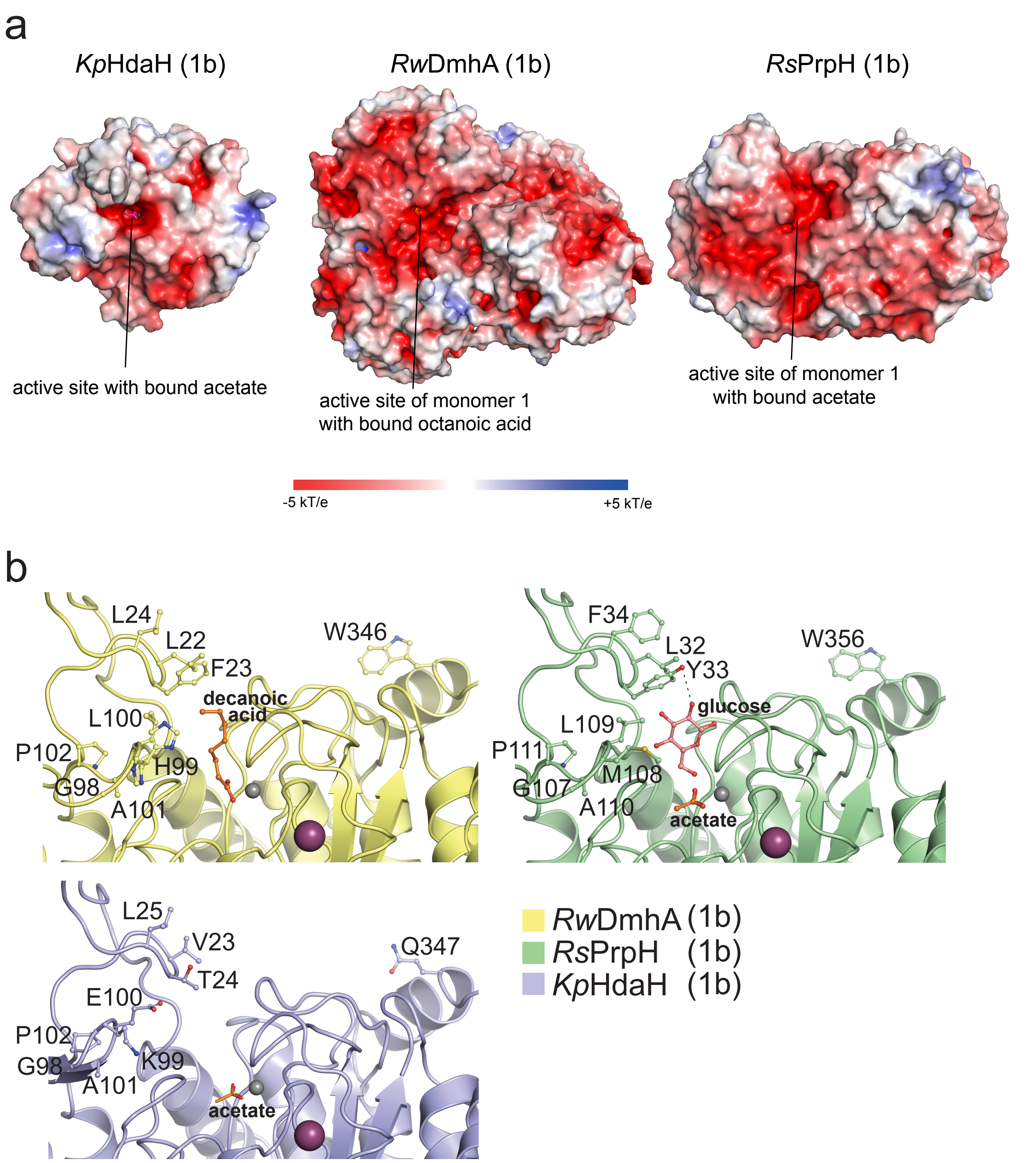

Supplementary Figure 15: Molecular determinant of substrate specificity for cluster 1 enzymes *Kp*HdaH (1b), *Rw*DmhA (1b) and *Rs*PrpH (1b).

a To show the electrostatics at the active site the electrostatic surface potential was plotted onto the surface of the structures of *Kp*HdaH (1b), *Rw*DmhA (1b) and *Rs*PrpH (1b). As visible, all enzymes show a highly negatively-charged surface surrounding the entry to the active site suggesting to play a role in substrate specificity towards proteins/molecules with neutral/positively charged electrostatic potential. All enzymes show a preference for peptides with a positively-charged residue at -1 position from the acetylated lysine side chain. For *Kp*HdaH (1b) a monomer is shown, for *Rw*DmhA (1b) a tetramer and for *Rs*PrpH (1b) a head-to-head dimer is shown. The electrostatics was calculated using the APBS plugin in PyMOL[4].

b Residues lining the substrate binding tunnel mediate substrate specificity of sub-cluster 1b enzymes. For *Rw*DmhA (1b) and *Rs*PrpH (1b) we observed differences in sequence motifs lining the substrate binding pocket in the L1-loop (22-L<u>F</u>L-24/32-L<u>Y</u>F-34 motif, and the motif 98-G<u>HL</u>AP-102/107-G<u>ML</u>AP-111 as well as W346/W356 in the C-terminal α-helix lining the active site rim. This is substituted by polar motifs in *Kp*HdaH (1b) (23-V<u>T</u>L-25; *Kp*HdaH: 99-G<u>KE</u>AP-102; 343-<u>F</u>IOO<u>Q</u>-347; Q347 corresponding to W346/W356 in *Rw*DmhA (1b) and *Rs*PrpH (1b). This is supported by the observation in *Rw*DmhA (1b) a hydrophobic octanoic acid and in *Rs*PrpH (1b) a glucose molecule contacted by Y33 of the 32-L<u>Y</u>F-34 motif is bound in the substrate binding channel. The substrate access to the active site is determined by the oligomerization of cluster 1b enzymes forming tetramers, the sequence of motifs lining the substrate binding tunnel affecting substrate binding sterically and electrostatically.

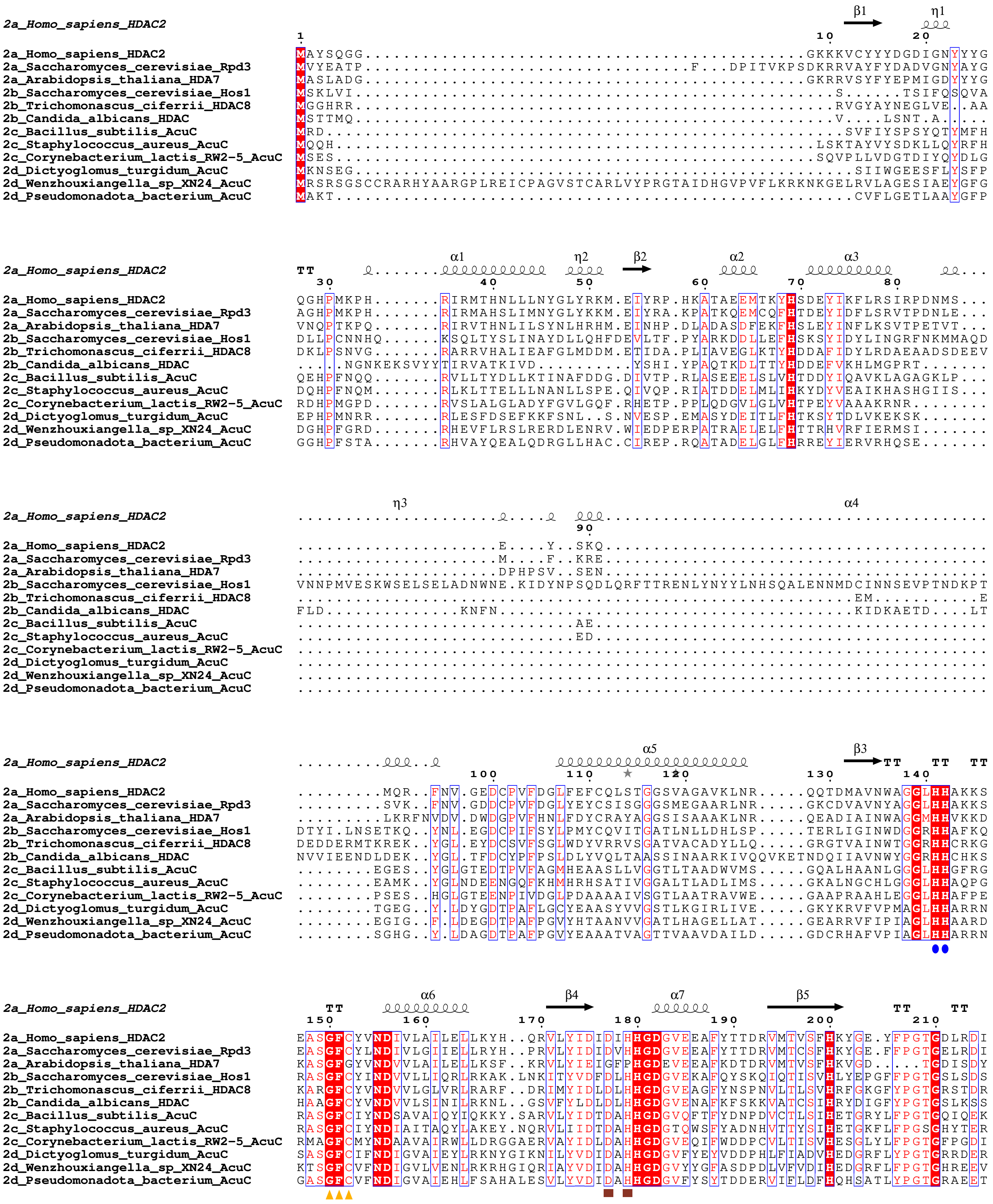
2a_Homo_sapiens_HDAC2
2a_Saccharomyces_cerevisiae_Rpd3
2a_Arabidopsis_thaliana_HDA7
2b_Saccharomyces_cerevisiae_Hos1
2b_Trichomonascus_ciferrii_HDAC8
2b_Candida_albicans_HDAC
2c_Bacillus_subtilis_AcuC
2c_Staphylococcus_aureus_AcuC
2c_Corynebacterium_lactis_RW2-5_AcuC
2d_Dictyoglomus_turgidum_AcuC
2d_Wenzhouxiangella_sp_XN24_AcuC
2d_Pseudomonadota_bacterium_AcuC
β1
η1
α1
η2
β2
α2
α3
η3
α4
α5
β3
α6
β4
α7
β5

```
2a_Homo_sapiens_HDAC2                 η4    β6  TT         α8            β7   η5  TT          α9
                                     220       230       240   ★   250       260       270   ★   280
2a_Homo_sapiens_HDAC2                GAGKGKYYAVNFPMRDGIDDESYGQIFKPIISKVMEMYQPSAVVLQCGADSLSGDRLGCFNLTVKGHAKCVE
2a_Saccharomyces_cerevisiae_Rpd3     GVGAGKNYAVNVPLRDGIDDATYRSVFEPVIKKIMEWYQPSAVVLQCGGDSLSGDRLGCFNLSMEGHANCVN
2a_Arabidopsis_thaliana_HDA7         GEGKGQYYSLNAPLKDGLDDFSLRGLFIPVIHRAMEIYEPEVIVLQCGADSLAGDPFGTFNLSIKGHGDCLQ
2b_Saccharomyces_cerevisiae_Hos1     RKD...KNVVNIPLKHGCDDNYLELIASKIVNPLIERHEPEALIIECGGDGLLGDRFNEWQLTIRGLSRIII
2b_Trichomonascus_ciferrii_HDAC8     GSGRGTGYTRNIPCRRGLSDESLKTIVQRAIVPFKQRFDPQAVVVVCGADGLSRDPHKEWNLTCEGLTSAVT
2b_Candida_albicans_HDAC             R.....ENTYNIPTEKGLNDSSMLWIIKEIVAPLISNFGPRAIVIQCGCDGLALDTHKEWNMTIKGYRDSID
2c_Bacillus_subtilis_AcuC            GSGKGYGYSFNIPLDAFTEDDSFLEAYRTAASEVAAYFEPDVIISQNGADAHYYDPLTHLSATINIYEEIPR
2c_Staphylococcus_aureus_AcuC        GEDIGYGHTVNVPLEPYTEDSSFLECFKLTVEPVVKSFKPDIILSVNGVDIHYRDPLTHLNCTLHSLYEIPY
2c_Corynebacterium_lactis_RW2-5_AcuC GGPSAAGTAVNIALPRDADDHTWLQAIHAAVPPLLQKFSPQLIISQHGADPHRADPLADLEVSIDAMGIAYR
2d_Dictyoglomus_turgidum_AcuC        GEKQAYGTKLNIPLLPGSGDDELLLAFEEV.KNFAWEVKIEFIILQAGVDGISGDPITHLNYSIEGYGKFVE
2d_Wenzhouxiangella_sp_XN24_AcuC     GIGPAEGRKLNIPLHPGAGDEEFNAAWEEC.EAFLAAARPEFYILAAGADSLAGDPLTHLQYSEGAHGRAAR
2d_Pseudomonadota_bacterium_AcuC     GTGGAQGKKLNIELLPQSRDETFFEQWSRA.RKFIDGFSPQFVLLQCGVDSLRGDPITDLHLSEAAHRFVTE
```

```
2a_Homo_sapiens_HDAC2                              β8           α10
                                         290   ★    300       310       320       330
2a_Homo_sapiens_HDAC2                VV....KTF.NLPLLMLGGGGYTIR.NVARCWTYETAVALDCEIP..NELPY....................
2a_Saccharomyces_cerevisiae_Rpd3     YV....KSF.GIPMMVVGGGGYTMR.NVARTWCFETGLLNNVVLD..KDLPY....................
2a_Arabidopsis_thaliana_HDA7         YV....RSF.NVPLMILGGGGYTLP.NVARCWCYETAIAVGEQLD..NDLPG....................
2b_Saccharomyces_cerevisiae_Hos1     NI...MKSYPRAHIFLLGGGGYNDL.LMSRFYTYLTWCVTKQF.S..N.LRCGDNNSFQIDPFDVCDGDDSE
2b_Trichomonascus_ciferrii_HDAC8     TV...L.EW.NLPSLLLGGGGYHHQ.DTARLWTLITAKAIHGP...............D........SGNNW
2b_Candida_albicans_HDAC             WI...LSHFSEIPIMLLGGGGYSHT.ETAKCWTYLTGSVLGVS...............D.........IDTW
2c_Bacillus_subtilis_AcuC            LAHTLAHQYCGGKWIAVGGGGYDIWRVVPRAWARIWLEMKGID.P.GHEIPP..EW............IVKW
2c_Staphylococcus_aureus_AcuC        FVKYLADTYTNGKIIMFGGGGYNIWRVVPRAWSHVFLSLIDQPIQ.SGYLPL..EW............INKW
2c_Corynebacterium_lactis_RW2-5_AcuC SMSSWARRFAGGKWVALGGGGYSRD.SVARAWTHVLAAVAEVELPADSRMPA..DW............G...
2d_Dictyoglomus_turgidum_AcuC        GMVDVANQTCGGKILILGGGGYNPE.NTKNGWMRVLRVIYGEF.............................
2d_Wenzhouxiangella_sp_XN24_AcuC     AVCRLADDHAQGRVLALGAGGYNRR.NVAAAWSRVVAEFADSL.............................
2d_Pseudomonadota_bacterium_AcuC     ELIGYAEQHCSGRLLALGGGGYNLE.NISNGWTAVTESMLN.A.............................
```

```
2a_Homo_sapiens_HDAC2                                                                    η6
                                                                                          340
2a_Homo_sapiens_HDAC2                .....N..............................................DYFEYFGPD.FKL....HIS
2a_Saccharomyces_cerevisiae_Rpd3     .....N..............................................EYYEYYGPD.YKL....SVR
2a_Arabidopsis_thaliana_HDA7         .....N..............................................DYMKYFRPD.YKL....HIL
2b_Saccharomyces_cerevisiae_Hos1     QFIREH..............................................DLVEMYNEENYQYWIYEMEG
2b_Trichomonascus_ciferrii_HDAC8     SDIPDH..............................................DFLSEYASDAYQFSFP....
2b_Candida_albicans_HDAC             DILPEH..............................................KNLDAYEKDGFRFWTDHNSG
2c_Bacillus_subtilis_AcuC            QKQCPVALPSSWSDPADLYPPIP.RKPEITEKNAQTVSKALYAIRSEQQRTK....................
2c_Staphylococcus_aureus_AcuC        KHYSSELLPKRWEDRLNDYTYVP.RTKEISEKNKKLALHIASWYES..TR.Q....................
2c_Corynebacterium_lactis_RW2-5_AcuC ....................HAPGRTLGDEDVDIGSLTSAFDAHQVMAERPHPALVQTSRAVFPYWGLPAYG
2d_Dictyoglomus_turgidum_AcuC        ........................................................................
2d_Wenzhouxiangella_sp_XN24_AcuC     ........................................................................
2d_Pseudomonadota_bacterium_AcuC     ........................................................................
```

```
2a_Homo_sapiens_HDAC2                              α11
                                     350      ★ 360          370 ★      380       390       400       410
2a_Homo_sapiens_HDAC2                PSN.MT...NQNTPEYMEKIKQR..LF...ENLRMLPHAPGVQMQAIPEDAVHEDSGDEDGEDPDKRISIRA
2a_Saccharomyces_cerevisiae_Rpd3     PSN.MF...NVNTPEYLDKVMTN..IFANLENTKYA....................................
2a_Arabidopsis_thaliana_HDA7         PTN.R.....QNLNTRLDIITMRETLLA...QLSLVM...................................
2b_Saccharomyces_cerevisiae_Hos1     SSR.MKMLRNDN...KDRD.MVE..LM......KF.....................................
2b_Trichomonascus_ciferrii_HDAC8     PNFNLP...DENK........FA.................................................
2b_Candida_albicans_HDAC             PSK.MK...DHNSVEYLNDIKTH..LL.............................................
2c_Bacillus_subtilis_AcuC            ........................................................................
2c_Staphylococcus_aureus_AcuC        ........................................................................
2c_Corynebacterium_lactis_RW2-5_AcuC S.......................................................................
2d_Dictyoglomus_turgidum_AcuC        ........................................................................
2d_Wenzhouxiangella_sp_XN24_AcuC     ........................................................................
2d_Pseudomonadota_bacterium_AcuC     ........................................................................
```

```
2a_Homo_sapiens_HDAC2
                                            420       430       440       450       460       470
2a_Homo_sapiens_HDAC2                SDKRIACDEEFSDSEDEGEGGRRNVADHKKGAKKARIEEDKKETEDKKTDVKEEDKSKDNSG.......EKT
2a_Saccharomyces_cerevisiae_Rpd3     .............PSVQLN..........HTPRDAEDLG......DVEEDSAEAKDTKGGSQYARDLHVEHD
2a_Arabidopsis_thaliana_HDA7         .............................HA...PSVPFQDTPSSSQATE.............AAEVDMEKR
2b_Saccharomyces_cerevisiae_Hos1     .................................................Y...................EL.
2b_Trichomonascus_ciferrii_HDAC8     ........................................................................
2b_Candida_albicans_HDAC             .....................................................................SL.
2c_Bacillus_subtilis_AcuC            ........................................................................
2c_Staphylococcus_aureus_AcuC        ........................................................................
2c_Corynebacterium_lactis_RW2-5_AcuC ........................................................................
2d_Dictyoglomus_turgidum_AcuC        ........................................................................
2d_Wenzhouxiangella_sp_XN24_AcuC     ........................................................................
2d_Pseudomonadota_bacterium_AcuC     ........................................................................
```

2a_Homo_sapiens_HDAC2

	480
**2a_Homo_sapiens_HDAC2**	D T K G T K S E Q L S N P
**2a_Saccharomyces_cerevisiae_Rpd3**	. . . . . N . E F Y . . .
**2a_Arabidopsis_thaliana_HDA7**	N . . . . D . P R . . . I
**2b_Saccharomyces_cerevisiae_Hos1**	. . . .
**2b_Trichomonascus_ciferrii_HDAC8**	. . . .
**2b_Candida_albicans_HDAC**	. . . .
**2c_Bacillus_subtilis_AcuC**	. . . .
**2c_Staphylococcus_aureus_AcuC**	. . . .
**2c_Corynebacterium_lactis_RW2-5_AcuC**	. . . .
**2d_Dictyoglomus_turgidum_AcuC**	. . . .
**2d_Wenzhouxiangella_sp_XN24_AcuC**	. . . .
**2d_Pseudomonadota_bacterium_AcuC**	. . . .

Supplementary Figure 16: Primary sequence alignment of selected bacterial deacylases of cluster 2 (UniProt accession numbers are shown).

Show are the following sequences of the following deacylases representing enzymes of cluster 2 and the sub-clusters (code: sub-cluster, accession number/protein name/spcies): 2a, Q92769/HDAC2/*Homo sapiens*; 2a, P32561/Rpd3/*Saccharomyces cerevisiae* (strain ATCC 204508 / S288c) (Baker's yeast); 2a, Q9FH09/Hda7/*Arabidopsis thaliana*; 2, bQ12214/Hos1/*Saccharomyces cerevisiae* (strain ATCC 204508 / S288c) (Baker's yeast); 2b, A0A642V618/HDAC8/*Trichomonascus ciferrii*; 2b, A0A8H6F6H7/HDAC/*Candida albicans*; 2c, P39067/AcuC/*Bacillus subtilis* (strain 168); 2c, Q6GFX3/AcuC/*Staphylococcus aureus* (strain MRSA252); 2c, A0A0K2H387/AcuC/*Corynebacterium lactis* RW2-5; 2d, B8DYU0/AcuC/*Dictyoglomus turgidum* (strain DSM 6724 / Z-1310); 2d, A0A6M1Y8Z6/AcuC/*Wenzhouxiangella sp.* XN24; 2d, A0A6L7MC72/AcuC/*Pseudomonadota bacterium*. All sequences were aligned using the T-COFFEE multiple sequence alignment server[1]. The important catalytic residues are highlighted. The secondary structure elements and numbering is shown for the deacylase shown at the top of the amino acid sequences above the sequence alignment. The alignment was created by ESPript version 3.0[2].

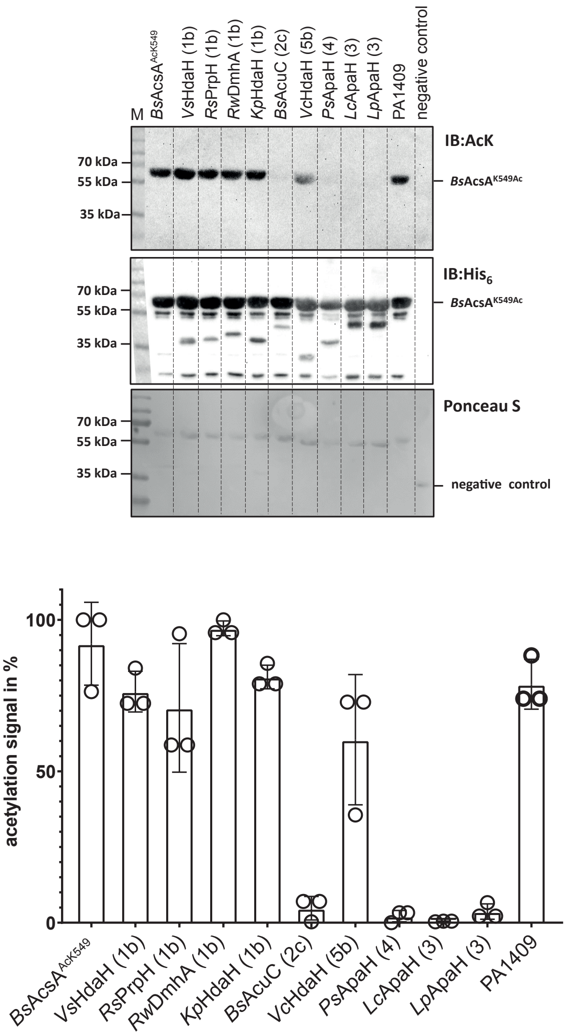

Supplementary Figure 17: Deacetylation of acetylated *Bacillus subtilis* deacetylase AcsA by bacterial deacylases.

Indicated bacterial deacylases were analysed for their capacity to deacetylate K549-acetylated *B. subtilis* AcsA. As a readout an immunoblot was performed and stained with an anti-acetyl-lysine antibody (IB: AcK). Lys549-acetylated *B. subtilis* AMP-forming acetyl-CoA synthetase (BsAcsA^{AcK549}) was used as positive control and non-acetylated His_6-Ran as negative control. Ponceau S-red staining was performed as loading control (LC: PoS). The quantification was done using ImageJ. The signal of the anti-AcK-blot was normalized to the anti-His_6-blot (IB: His_6) used as loading control. The highest signal of each blot was set to 100%. One representative immunoblot is shown. Ponceau S-red staining of the membrane was done as second loading control. Three biologically independent replicates were performed (*n*=3). The bar graph shows the values mean±SD. Source data are provided as Source Data file.

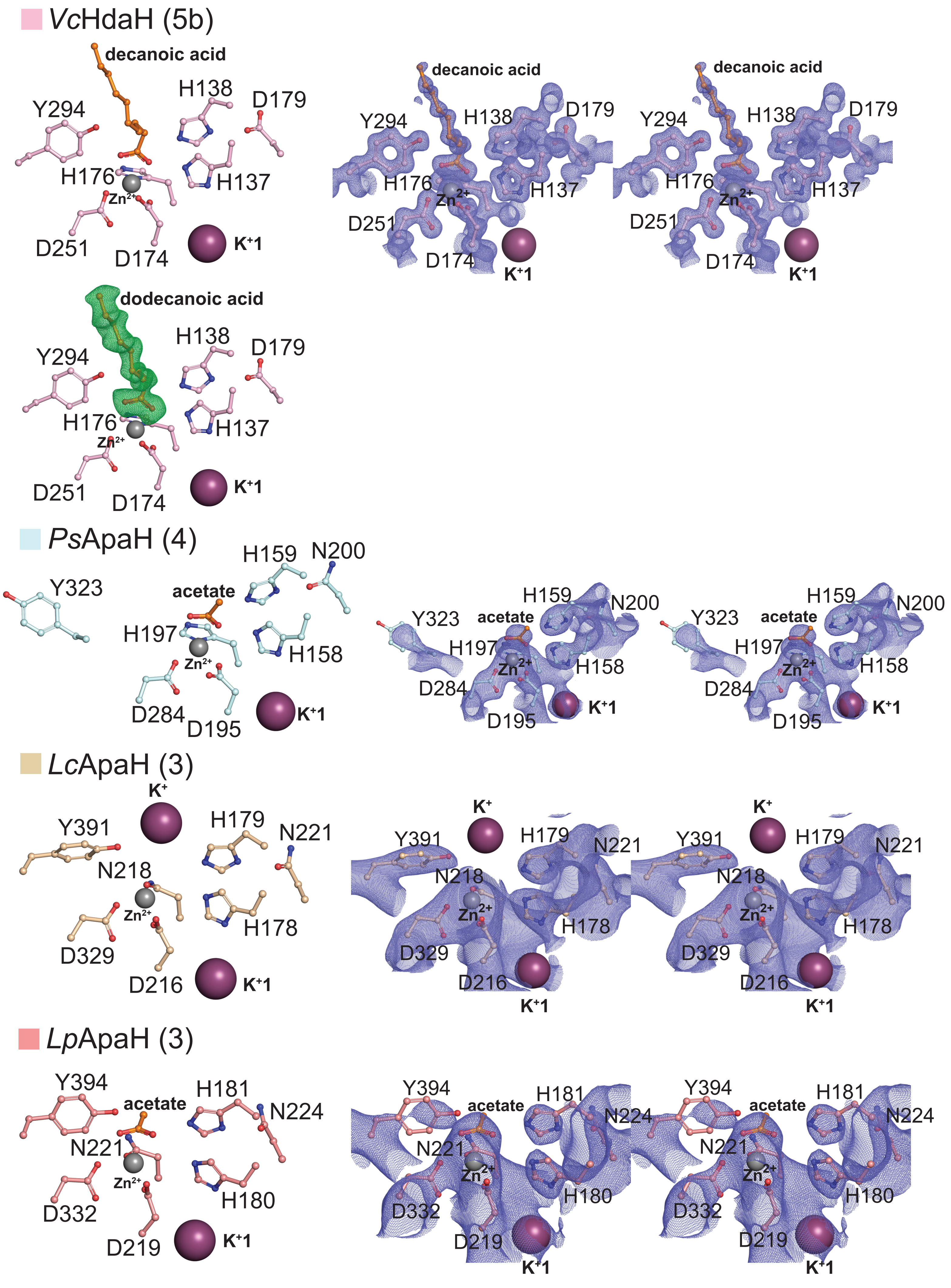
VcHdaH (5b)
decanoic acid
Y294
H138
D179
H176
Zn2+
H137
D251
D174
K+1
dodecanoic acid
PsApaH (4)
Y323
acetate
H159
N200
H197
H158
D284
D195
LcApaH (3)
K+
Y391
H179
N221
N218
H178
D329
D216
LpApaH (3)
Y394
H181
N224
N221
H180
D332
D219

Supplementary Figure 18: Stereo images of representative electron density for structures of bacterial deacylases solved in this study.

Stereo figures for the structures of bacterial deacylases: *Vc*HdaH of sub-cluster 5b, *Lc*ApaH as well as *Lp*ApaH of cluster 3 and *Ps*ApaH of cluster 4. For all structures a representative closeup of the active site is shown on the left, including the catalytic Zn^{2+}-ion, including the coordinating His and the two Asp residues, the catalytic base His and the active site Tyr important to orient and polarize the carbonyl-group of the substrate and to stabilize the tetrahedral intermediate. As stereo images the closeup of the active site is shown with the is the electron density of the $2F_o$-F_c map contoured at 1σ in blue. For *Vc*HdaH (5b) the F_o-F_c omit map is shown in green for the bound decanoic acid (contour level 3σ). In violet spheres the potassium ions are shown, the catalytic Zn^{2+}-ion is shown as grey sphere. The stereo diagrams were prepared in wall-eyed viewing mode with PyMOL version 2.3.4[3].

```
                                                      β1
5a_Acinetobacter_wuhouensis_HDAC
                                        1
5a_Acinetobacter_wuhouensis_HDAC        MLN................................................................
5a_Neisseriaceae_bacterium_HDAC         MK.................................................................
5a_Treponema_sp_HDAC                    MRNPG..............................................................
5b_Vibrio_cholerae_HdaH                 MIP................................................................
5b_Pseudomonas_alcaligenes_AcuC         MLP................................................................
5b_Leptospiraceae_bacterium_HDAC        MVP................................................................
5c_Moraxella_bovis_HDAC                 MFF................................................................
5c_Flavobacterium_indicum_HDAC-family   MYS................................................................
5c_Bdellovibrionales_bacterium_HDAC     MR.................................................................
5d_Turneriella_parva_HdaH               MSRK...............................................................
5d_Leptospira_interrogans_HdaH          MEKHL..............................................................
5d_Leptospira_brenneri_HDAC             MASN...............................................................
5e_Archangium_gephyra_HDAC              MNPLLALLA..K.......................................................
5e_Nannocystis_exedens_AcuC             MTTGEKIFR..QL.........................RRRA....................NQMFA
5e_Deltaproteobacteria_bacterium_HDAC   ILE................................................................
5f_Micavibrio_sp_HDAC_domain            MNRLTFIIT..LL.........................IQ..........................K
5f_Homo_sapiens_HDAC11                  MLHTTQLY..........................................................Q
5f_Arabidopsis_thaliana_HDA2            MTHTRVISTWTELTRDLAIYLLFTFFAIKVFKFLFSCNRTSEISSFSMATHPEALRRERILNSKLYF
5g_Bacteriovorax_stolpii_AcuC-like      MI.................................................................
5g_Gracilinema_caldarium_HdaH           MIL................................................................
5g_Bacteriovorax_sp_MedPE-SWde_HDAC_domain MSGKR..............................................................

                                                     η1                α1
5a_Acinetobacter_wuhouensis_HDAC
                                                     10        20        30
5a_Acinetobacter_wuhouensis_HDAC        ..........VCFS.PRYF.AQT.......HTN.SME..KLLAVAEQLQQFNQN.............
5a_Neisseriaceae_bacterium_HDAC         ..........VFFD.ERQN.VEQNE.....SFSPSAK..KPKACLESWKK...I.............
5a_Treponema_sp_HDAC                    ......LPVPVFYD.PRQS.AARNE.....SLSPSAQ..KPARVVEDWKA..TG.............
5b_Vibrio_cholerae_HdaH                 ..........LIYH.PIYSQLDLPV....GHRY.PIN..KYRLLYEEIVR..QREQSEA........
5b_Pseudomonas_alcaligenes_AcuC         ..........LVYH.DDYS.PPFPA....NHRF.PME..KFRLLRDHLVD..SG.............
5b_Leptospiraceae_bacterium_HDAC        ..........LVYH.PDYS.FAFPR....DHRF.TMS..KFKLTYENLVR..YG.............
5c_Moraxella_bovis_HDAC                 ..........KIAHCHEFA.LPLPD....GHRF.PMA..KYELLPKKLIA..DG.............
5c_Flavobacterium_indicum_HDAC-family   ..........IAYH.PIYK.HPLPE....GHRF.PMI..KYELLPQQLLY..EG.............
5c_Bdellovibrionales_bacterium_HDAC     ...........VYQSDQYT.LPLPE....NHRF.PMQ..KYRILKEELVK..QQ.............
5d_Turneriella_parva_HdaH               .......SAELVYS.PIYD.LSDY.....THVI.SAG..KYRQLYDALK..................
5d_Leptospira_interrogans_HdaH          ......ERIGLVYH.PDYN.LDLG.....PHVF.PAR..KYQMVYDLVKR..DS.............
5d_Leptospira_brenneri_HDAC             .......ALALIYH.SSYN.LELP.....GHVF.PAH..KYSHLYNRVKR..DP.............
5e_Archangium_gephyra_HDAC              .GR..RRPLRIFYD.EAFR.LPISGVEAGPRSM.ETR..RADDALHYLQS..AH.............
5e_Nannocystis_exedens_AcuC             RLR..PPPAAFVYG.PHYE.CSWPG.....SAV.DEV..RGERILTALAA..EG.............
5e_Deltaproteobacteria_bacterium_HDAC   .......................................RGAQVLDNLVH..RG.............
5f_Micavibrio_sp_HDAC_domain            FMPDTTIKHPVIYH.PRYK.LGSGWLKY.LHKL.DMD..KPGDLYRALLQ..KG.............
5f_Homo_sapiens_HDAC11                  HVP..ETRWPIVYS.PRYN.ITFMGLEK.LHPF.DAG..KWGKVINFLKE..EK.............
5f_Arabidopsis_thaliana_HDA2            DVP..LSKVSIIYS.SSYD.ISFMGIEK.LHPF.DSS..KWGRVCKFLVS..DG.............
5g_Bacteriovorax_stolpii_AcuC-like      ...........FYH.PDCD.LRFSEY...GIEI.PIVDERASHVFSALKY.LYP.............
5g_Gracilinema_caldarium_HdaH           ...........LYD.PALD.MRFPDY...GIQI.PIRDRRALAVLEYLKK..KI....IPPMRPVHS
5g_Bacteriovorax_sp_MedPE-SWde_HDAC_domain ........NLVIYN.PCSL.QHYKEY...GIGI.PMLNERVEKPFAYISQ..KY.............

                                          β2              α2          α3          α4
5a_Acinetobacter_wuhouensis_HDAC
                                           40             50          60          70
5a_Acinetobacter_wuhouensis_HDAC        NVKNFEFHEP....KILDPEILKALHSPKYVEAFMTGQP.NK.L.A................TFAGF
5a_Neisseriaceae_bacterium_HDAC         V..PVEIMEV....TPVTAEDLSLIHDQDHVLKVLSCEK.EN....................GFQNK
5a_Treponema_sp_HDAC                    F..PLEFRGF....QPLAPGEIARAHDPDYVRGVLACEI.SN....................GFGNT
5b_Vibrio_cholerae_HdaH                 WQASFEFHTP....IAAELSRITPLHDPDYVQALLEGRL.PA.AKM.................RRIG
5b_Pseudomonas_alcaligenes_AcuC         LVRDGELHRP....EICPADVLALAHCPAYIERFASGEL.TY.QEQ.................RRLG
5b_Leptospiraceae_bacterium_HDAC        LVH.NNLFTP....EKPEIEQFHLAHDPGYVKALCKNTL.DE.KAW.................RRIG
5c_Moraxella_bovis_HDAC                 IATPEQFFTP....KMASVNEILTTHTADYWHALDNLTL.SP.KDC.................RKIG
5c_Flavobacterium_indicum_HDAC-family   TVVDRQFFSP....ELPNLEDIIAVHDKKYVQQLLDLTL.DP.RAV.................RKIG
5c_Bdellovibrionales_bacterium_HDAC     ILTVDQIFEA....PLIEIEDLYLAHDKQYVDHIINGTL.DA.KEQ.................RKIG
5d_Turneriella_parva_HdaH               .FSRWNWNEP....VAATKEQLLLVHTERYLKDFLGARL.TE.QTQ..................RAE
5d_Leptospira_interrogans_HdaH          KLSNLYIYKP....DLAKTKDLSLVHTQEFLDDFFSLNI.TE.RTQ..................YSE
5d_Leptospira_brenneri_HDAC             VYASWDILLP....KKAEDADLELVHTKEYLDDLFSYEH.TS.RTM..................YSE
5e_Archangium_gephyra_HDAC              AVSRREIINP....TPASYEALGRVHTAAYLESLFQAET.LA.HIF................AVDPS
5e_Nannocystis_exedens_AcuC             LVTFEQVRRP....PLATYHAIRRVHDDAYLDACQRPEV.MS.DIL...............GGLAVS
5e_Deltaproteobacteria_bacterium_HDAC   FLRLSDVRAP....GLVPLDELAWVHTEAYLDTTVTPDAL......................AHIFG
5f_Micavibrio_sp_HDAC_domain            LIDADHIYTA....PQATNAQLKLAHNQDYIDNKIGNKVWLA.DVF.............QLNL.IKW
5f_Homo_sapiens_HDAC11                  LLSDSMLVEA....REASEEDLLVVHTRRYLNELKWSFA.VA.TIT.............EIPP.VIF
5f_Arabidopsis_thaliana_HDA2            FLEEKAIVEP....LEASKIDLLVVHSENYLNSLKSSAT.VA.RIT.............EVAP.VAF
5g_Bacteriovorax_stolpii_AcuC-like      FLEYTDLKTL....PRITREDLERVHNKDFIHRLLGTAGERQGEIYQCYELVNDQGKFERYNPKNQK
5g_Gracilinema_caldarium_HdaH           LASAADMLSIPEGDRHISREDIARAHDANFVAALFGEGLEK..ELIKTYELMDEQGRFRRYSPGDAV
5g_Bacteriovorax_sp_MedPE-SWde_HDAC_domain GVQATDEALF....SKFGGEILKLAHSKEYCEKVENNPD.Q..VVLDTYELIKDDGSYNRYDPSCAT
```

```
                                                                      α5                        β3
5a_Acinetobacter_wuhouensis_HDAC                                                                         TT
                                        80        90        100                          110
5a_Acinetobacter_wuhouensis_HDAC        KP....WNEQLRDAVLAINAGQLMATELAFE..................H.GI.A.ANIAQGFHH
5a_Neisseriaceae_bacterium_HDAC         L.......KTIADSLLWTNGSFVSAARYAVK..................N.KI.STVSPTSGFHH
5a_Treponema_sp_HDAC                    F.......PEVAAALPWTTGSMYAAAWAAE...................RGGF.A.ASPTSGFHH
5b_Vibrio_cholerae_HdaH                 FP....WSKTLIERTLHSVGGTCLTVEQALQ..................S.GV.A.IHLSGGYHH
5b_Pseudomonas_alcaligenes_AcuC         LP....WSEPLARRTVRAVGGSLLAAELALK..................H.GA.A.CHLAGGTHH
5b_Leptospiraceae_bacterium_HDAC        LP....FSEQLIHRTLIAPAGTLLASKLALE..................H.GV.A.CHIGGGTHH
5c_Moraxella_bovis_HDAC                 LP....MSKELIDRERLVVGATIECAKFALN..................D.GI.S.LSTSGGTHH
5c_Flavobacterium_indicum_HDAC-family   FP....LSSELIEREFRLAQGTIEGAKNACI..................D.KI.A.FNIAGGTHH
5c_Bdellovibrionales_bacterium_HDAC     FP....WSPIMPLRSRASVGGFMAACRYALE..................N.GF.A.GNLSGGTHH
5d_Turneriella_parva_HdaH               IP....IDERIVNAVCTAAGGTILAAELALK..................H.GV.A.SNLSGGFHH
5d_Leptospira_interrogans_HdaH          LP....LTKQIVHSFVLAVGGTILSMELTQK..................Y.KF.V.YHIGGGFHH
5d_Leptospira_brenneri_HDAC             LP....LNRSIVESFMYGVGGTVMAAELSKN..................F.QF.A.FNMGGGYHH
5e_Archangium_gephyra_HDAC              DV....YVDEVLRTLRLAVGATIAAASFSLR..................R.RVPT.INLFGGFHH
5e_Nannocystis_exedens_AcuC             EV....DTARILDLQRAMTGGTMLATDIALG..................TGKI.A.INLGGGFHH
5e_Deltaproteobacteria_bacterium_HDAC   VPPETVDV.DALVRGQRRAVGGTVEAALKVTRG................EV.KV.G.FNLGGGFHH
5f_Micavibrio_sp_HDAC_domain            VP.FT.LTQHLLVTPLLYHTGGTILAGELAAE..................K.GW.A.MNLGGAHH
5f_Homo_sapiens_HDAC11                  LP.NF.LVQRKVLRPLRTQTGGTIMAGKLAVE..................R.GW.A.INVGGGFHH
5f_Arabidopsis_thaliana_HDA2            FP.NF.LVQQKVLYPFRKQVGGTILAAKLATE..................R.GW.A.INIGGGFHH
5g_Bacteriovorax_stolpii_AcuC-like      RD....W.KELVETILMQVAMTYNSTKYALK..................N.GF.S.YHLGGGMHH
5g_Gracilinema_caldarium_HdaH           RP....L.SELFTTIRAQVWGTYLACRLALAHRSKPSGVSSEPLSTNNSNP.GF.C.YYLGGGMHH
5g_Bacteriovorax_sp_MedPE-SWde_HDAC_domain LP....L.SDFVKKALSHINGTILACESAMD..................S.GF.S.YHLGGGMHH

                                              α6                β4          α7          β5
5a_Acinetobacter_wuhouensis_HDAC           TT    TT
                                        120       130       140       150       160       170
5a_Acinetobacter_wuhouensis_HDAC        ARPEYGGSFCTFNGLALIAQ....Q.YPNKRIFILDCDQHGGDGTAEFTRKYNNLYN.FSIYGLA..
5a_Neisseriaceae_bacterium_HDAC         AEYKKCIGFCTFNGLMLAVLLKKE.GLANKIAILDTDMHYGNGTDNIINKLKIDY.VSHYTLG...
5a_Treponema_sp_HDAC                    ACWDRGMGFCTFNGLAIAAVLLRER.GLARRVGILDLDMHYGNGTAEILDRLGLSGEVLHYTFG...
5b_Vibrio_cholerae_HdaH                 AHADFGSGFCLFNDLAIAAHFALSL.PSVDKVLIIDSDVHHGDGTATLCAERDDIIT.LSFHCDK..
5b_Pseudomonas_alcaligenes_AcuC         AHFDEASGFCIFNDLAVVARYMLES.GRAGRVLIYDCDVHQGDGTARLLENVPDAIT.VSLHCEK..
5b_Leptospiraceae_bacterium_HDAC        AHYSFGSGFCLLNDLAFSSLALNQM.KPDLKILIFDLDVHQGDGTASILKNEKNIYT.VSVHCEK..
5c_Moraxella_bovis_HDAC                 AFADSGEGFCILNDICVASNVLLNQ.GLAKRILILDLDVHQGNGNASIMADNPHVFV.FSMHGKK..
5c_Flavobacterium_indicum_HDAC-family   AYSNRGEAFCLLNDQAIAAQYLLNQ.KLAKQILIIDLDVHQGNGTAEIFQNNPRVFT.FSVHGKS..
5c_Bdellovibrionales_bacterium_HDAC     AHRAHGGGFCVFNDFAVAAYVLKQK.HKITQLAIVDLDVHQGDGNASMLQGSQGIFI.FSMHGEK..
5d_Turneriella_parva_HdaH               AFADHAEGFCFVNDTVLAIRALRKT.RPGLKVAVIDLDVHQGNGTAKLLQGDENSYT.FSMHEKE..
5d_Leptospira_interrogans_HdaH          SMPDRAEGFCYLNDAAIASKLYQKE.YPDKKILFIDLDLHQGNGNSFIFQNDPDVFT.FSMHQEN..
5d_Leptospira_brenneri_HDAC             SFPDKAEGFCYLNDVAVAIRKQKET.NPDLNALIIDLDLHQGNGNSYIFQYDDKVFT.FSMHQGN..
5e_Archangium_gephyra_HDAC              AAPNRGAGFCALNDMAVAIAELRAQ.GFQGKVGILDLDFHPPDGTSACLGEDASVWL.GSISGAD..
5e_Nannocystis_exedens_AcuC             AHRQIGRGFCVFHDVAVAIAEQRAL.GFHERILVVDLDQHDGDGTRALFADDPSVFT.LSIHNH...
5e_Deltaproteobacteria_bacterium_HDAC   AEPSKGSGFSIYNDVAVAIARVRHD.GYKGRIAIVDLDFHQGNGSLVAFADDPTVFT.YSVHGSA..
5f_Micavibrio_sp_HDAC_domain            AHANDASGFCMVADITMAIKNLRQRHKHMRNIMIIDLDAHQGNGPERDFANDPDVYI.IDMFTYGVM
5f_Homo_sapiens_HDAC11                  CSSDRGGGFCAYADITLAIKFLFERVEGISRATIIDLDAHQGNGHERDFMDDKRVYI.MDVYNRH..
5f_Arabidopsis_thaliana_HDA2            CTAERGGGFCAFADISLCIHFAFLR.LRISRVMIIDLDAHQGNGHETDLGDDNRVYI.LDMYNPE..
5g_Bacteriovorax_stolpii_AcuC-like      SMSFAGRGFCLVNDIVITLRKLQNE.GAIKTAWIVDVDVHKGDGAPEILQNDTTIRT.FSIHMKE..
5g_Gracilinema_caldarium_HdaH           ARYDSGAGFCLLNDIIIAARRLKAE.GLISSIWIIDLDAHKGNGTAELASGDPCILT.LSIHMAL..
5g_Bacteriovorax_sp_MedPE-SWde_HDAC_domain AMSFRPGGFCMFNDMVTAIKYLQQN.GKIKNAVIIDMDAHKGDGTAQMTYSDDSIRS.FSIHMKG..

                                                              β6                α8              β7
5a_Acinetobacter_wuhouensis_HDAC                  TT                  TTT
                                            180                 190       200       210       220
5a_Acinetobacter_wuhouensis_HDAC        ....FGYP.TYER..........AESRHVHTKNG.SF.AQYLMALHEAFQK...AH.E.WQADIII
5a_Neisseriaceae_bacterium_HDAC         ....KHYP.TQDE..................QEVVA.AKFLKELPSIVED...LI...KDVDVLF
5a_Treponema_sp_HDAC                    ....G.DPKTADR..................EGFDG.ELWLALLDGIVRG...F....ADCEVLL
5b_Vibrio_cholerae_HdaH                 .....NFP.ARKP..........ASSMDVGFANQTGD.EEFLSTFIQVVEM...AVNL.HRPDLIL
5b_Pseudomonas_alcaligenes_AcuC         .....NFP.ARKA..........KSDWDIGLPIGMGD.TDYLKVVHDSLGY...LLAL.YQPDIVL
5b_Leptospiraceae_bacterium_HDAC        .....NFP.YRKE..........TSTVDVGLYAGMSD.DEYLTIVESTLNE...SISR.FSPDIVL
5c_Moraxella_bovis_HDAC                 .....NYP.FIKP..........LSDLDIELDDGTGD.DDYLSLLKYHLPK...VLDD.FAPDFVF
5c_Flavobacterium_indicum_HDAC-family   .....NYP.FKKE..........QSDLDIALPDQTSD.ADYLALISKEIPK...LIQQ.VQPDFIF
5c_Bdellovibrionales_bacterium_HDAC     .....NYP.FKKI..........NSDLDIALPNQCQD.DEYLTKLSDALPK...VIA..TRPQIIL
5d_Turneriella_parva_HdaH               .....NYP.VKE...........TGSHDVELPSHLGD.AEYLRLLAENLDK...LKAA.FFPDLIF
5d_Leptospira_interrogans_HdaH          .....LYP.KKE...........KSDLDISLEEGIGD.KEYLELLEKSLRK...IESD.FKPDLIF
5d_Leptospira_brenneri_HDAC             .....LYP.KKE...........VSNLDVNLEPNTKD.DEYLSTLESSLNR...IRKD.FDSNIIY
5e_Archangium_gephyra_HDAC              .....WGS.L.P...........G.VDEVRLPPGTSD.GAYLDALDGLLDR...MP....SLELVF
5e_Nannocystis_exedens_AcuC             ....HWGG.TEAI...........ASTSVALGDVVGD.TLYLETVQLHLTE...ALRQ.HRPRLIY
5e_Deltaproteobacteria_bacterium_HDAC   ......WV.QRQAT.........ADAGILPPRTGD.RAYLEALRYSLPD...VFAE.FKPEVVF
5f_Micavibrio_sp_HDAC_domain            PDGDMFYP.DDRRAM.......QRINIPVKLNFNTKD.DTYLQKLQNALKT...AKTD.FAPDMIF
5f_Homo_sapiens_HDAC11                  .....IYP.GDRFAK.......QAIRRKVELEWGTED.DEYLDKVERNIKK...SLQE.HLPDVVV
5f_Arabidopsis_thaliana_HDA2            .....IYP.FDYRAR.......RFIDQKVEVMSGTTT.DEYLRKLDEALEV...ASRN.FQPELVI
5g_Bacteriovorax_stolpii_AcuC-like      .....GWP.LNSGTVRDP...WFIPSSIDVGIDV.GED.DQYLARLESGLLE...MQERFPNPDVVI
5g_Gracilinema_caldarium_HdaH           .....GWP.LDPETLAHAVPSRAPLVASDVDIPVSKEDQASYVSRLASGLLQLEMLSGG.KKPDLAI
5g_Bacteriovorax_sp_MedPE-SWde_HDAC_domain .....GWP.LDKEDKNDP..S.YIPSSFDI..EV.ERD.DDYLSLLKKGLED...FN...PREDFAI
```

```
5a_Acinetobacter_wuhouensis_HDAC                  TT TT TT    TT      α9                β8
                                                  230         240       250       260       270       280

5a_Acinetobacter_wuhouensis_HDAC            YQAGMDCHQADPYGS...AWFTTELIQKRDEMVFNMAKKAG..IPIMFVLAGGYQD.LGDLTALHT.
5a_Neisseriaceae_bacterium_HDAC             YQAGADPHVDDPYGG...L.LTTEDLKQRDKIVFSIAKEKG..IPVVWNLAGGYQD.FDVVLE....
5a_Treponema_sp_HDAC                        YQAGADPHKDDPLGG...L.LTTGQLRERDRVVFRTCAELG..LPAAWNLAGGYRKPFEGVLE....
5b_Vibrio_cholerae_HdaH                     YDAGVDIHNDDELGY...LSISQAAIAQRDRFMLGLAKQES..IPIACVIGGGYREDHAALVPLHLE
5b_Pseudomonas_alcaligenes_AcuC             YDGGVDVHKDDALGY...LQLTDAGIAARDEAVFRHCLERD..IPVVGLIGGGYDKDRAVLARRHGI
5b_Leptospiraceae_bacterium_HDAC            YDAGVDIWEHDKLGL...LNISWQGIFERDKLVFEKFKQAG..IPIASVIGGGYDPDEHELARRHSI
5c_Moraxella_bovis_HDAC                     YQAGVDVLADDRLGR...INLTMNGLYERERYVIETLFNRR..LPTVVTMGGGYAPDINIIVQGHSV
5c_Flavobacterium_indicum_HDAC-family       YLSGVDILATDKLGK...LGCTINGCKERDVIVFENCLKFQ..IPVQVSMGGGYSPDIKTIIEAHAN
5c_Bdellovibrionales_bacterium_HDAC         FQAGVDPLFSDKLGH...LALSFEGLKQRDQLVLGLCKEYG..IPVAIAMGGGYAEPIDDSIKAHLN
5d_Turneriella_parva_HdaH                   YVAGVDIYRDDALGG...LQLSFDGIAARDAAVRDFLPE....VPKVVLPAGGYARNIAETVALHAQ
5d_Leptospira_interrogans_HdaH              YIAGADPFEGDSLGD...LKLTFQGLRKRDQIVRDFAYSLN.DTRVVILPAGGYAKDFYDTVTIHYN
5d_Leptospira_brenneri_HDAC                 YVAGADPYEDDSLGE...LKISMKGLKERDLMVRKFAETLN..VPCVVTLAGGYARDFRDTVEIHFN
5e_Archangium_gephyra_HDAC                  VLSGADVLAGDRLGQ...FSMSLPGVRERDLRVARRL..GS..MPQVWLPAGGYSPHAWKVLAGVGL
5e_Nannocystis_exedens_AcuC                 FLAGTDPAVGDRIGV...WQISAEGMLERDIAVFEAIREFAPDAAVVVCLAGGYGPTTWQHSARSFA
5e_Deltaproteobacteria_bacterium_HDAC       YLAGNDVLAGDALGD...FVLSPRGVLDRDLYVTRLTREHS..AGLVVVIAGGYGKFAWQCTANYLR
5f_Micavibrio_sp_HDAC_domain                YIAGSDILEDDALGM...QAITKSGLIERDEMVFAFAKELG..APIVSVQAGGYQPNLGKIVADSIE
5f_Homo_sapiens_HDAC11                      YNAGTDILEGDPLGG...LSISPAGIVKRDELVFRMVRGRR..VPILMVTSGGYQKRTARIIADSIL
5f_Arabidopsis_thaliana_HDA2                YNAGTDILDGDPLGL...LKISPDGITSRDEKVFRFAREKN..IPLVMLTSGGYMKSSARVIADSIE
5g_Bacteriovorax_stolpii_AcuC-like          VVNGADPYEHDELPSASFINLSKEQMLARDKFLYQFFKERN..IPQSYVMAGGYGSKAWEIYLQFLK
5g_Gracilinema_caldarium_HdaH               VVDGADPYEKDELPSSVDLQLPLDTCVKRDMTVFSFLRDRA..IPSTWLLAGGYGDYAWEPPAHFLT
5g_Bacteriovorax_sp_MedPE-SWde_HDAC_domain  VVHGADVYEKDELESASLIQLTKEEVLERDKITYNLLKDRG..IPQAWVMAGGYGKYVHEIFTQFLD


5a_Acinetobacter_wuhouensis_HDAC            ...................................................................

5a_Acinetobacter_wuhouensis_HDAC            ...................................................................
5a_Neisseriaceae_bacterium_HDAC             ...................................................................
5a_Treponema_sp_HDAC                        ...................................................................
5b_Vibrio_cholerae_HdaH                     LLKAAL..L..........................................................
5b_Pseudomonas_alcaligenes_AcuC             LHHSAARVL..........................................................
5b_Leptospiraceae_bacterium_HDAC            VFEAANSVF..........................................................
5c_Moraxella_bovis_HDAC                     VFGVSQEIF..........................................................
5c_Flavobacterium_indicum_HDAC-family       TYRVANSLF..........................................................
5c_Bdellovibrionales_bacterium_HDAC         TYIVAKELY..........................................................
5d_Turneriella_parva_HdaH                   TIKIMHGRQ..........................................................
5d_Leptospira_interrogans_HdaH              TIKIFAA.D..........................................................
5d_Leptospira_brenneri_HDAC                 TITAFGE.K..........................................................
5e_Archangium_gephyra_HDAC                  ALAFD...TDEPIP.TDYDPL..EARFSGISHSLPS.ESLGDE...PFVSEADVADALGLPRQGPHR
5e_Nannocystis_exedens_AcuC                 WLQSG...HRGFEP.PDNDELT.LMRARHYAALLKPAELTGEDD.DLGLTLEDLVPSLAATAREP.R
5e_Deltaproteobacteria_bacterium_HDAC       WLLT...DNAHAARYPDA...PLKRKFRQISRELGASELQRVDDGELAFHEEDILGDL..TNRGPPK
5f_Micavibrio_sp_HDAC_domain                NLDNKFNL...........................................................
5f_Homo_sapiens_HDAC11                      NLFGLGLIGPES.......................................................
5f_Arabidopsis_thaliana_HDA2                NLSRQGLIQTR........................................................
5g_Bacteriovorax_stolpii_AcuC-like          FVGE...............................................................
5g_Gracilinema_caldarium_HdaH               KVLT...............................................................
5g_Bacteriovorax_sp_MedPE-SWde_HDAC_domain  YSLD...............................................................


5a_Acinetobacter_wuhouensis_HDAC            ...................................................................

5a_Acinetobacter_wuhouensis_HDAC            ...................................................................
5a_Neisseriaceae_bacterium_HDAC             ................IHNNT..........................ALLHKE..............
5a_Treponema_sp_HDAC                        ................IHRNT..........................MAECVA..............
5b_Vibrio_cholerae_HdaH                     ...................................................................
5b_Pseudomonas_alcaligenes_AcuC             ...................................................................
5b_Leptospiraceae_bacterium_HDAC            ...................................................................
5c_Moraxella_bovis_HDAC                     ...................................................................
5c_Flavobacterium_indicum_HDAC-family       ...................................................................
5c_Bdellovibrionales_bacterium_HDAC         ...................................................................
5d_Turneriella_parva_HdaH                   ...................................................................
5d_Leptospira_interrogans_HdaH              ...................................................................
5d_Leptospira_brenneri_HDAC                 ...................................................................
5e_Archangium_gephyra_HDAC                  F.LEFYTAQGMEFALERYRILPILRRLGFQGLHVVIDQVAPYDR.ARLLGVDAIT.GKTA..SLIEL
5e_Nannocystis_exedens_AcuC                 F.LGYYSRQGLELALERHGFLGRLRGKGFQ.PSLEFNLVDPQRHILKIFGDP..E.HK.E..LLFEL
5e_Deltaproteobacteria_bacterium_HDAC       LIHGYYTRQGIEFALERFGFLPAIRERGFAQLRTEITGMDARAPT...LHLYGKAHGKGAELLLVEL
5f_Micavibrio_sp_HDAC_domain                ...................................................................
5f_Homo_sapiens_HDAC11                      .................PSV...............................................
5f_Arabidopsis_thaliana_HDA2                ...................................................................
5g_Bacteriovorax_stolpii_AcuC-like          ...................................................................
5g_Gracilinema_caldarium_HdaH               ...................................................................
5g_Bacteriovorax_sp_MedPE-SWde_HDAC_domain  ...................................................................
```

```
5a_Acinetobacter_wuhouensis_HDAC                 .................................................................ℓℓ

5a_Acinetobacter_wuhouensis_HDAC                 .................................................................QT
5a_Neisseriaceae_bacterium_HDAC                  ...................................................................
5a_Treponema_sp_HDAC                             ...................................................................
5b_Vibrio_cholerae_HdaH                          ...................................................................
5b_Pseudomonas_alcaligenes_AcuC                  ...................................................................
5b_Leptospiraceae_bacterium_HDAC                 ...................................................................
5c_Moraxella_bovis_HDAC                          ...................................................................
5c_Flavobacterium_indicum_HDAC-family            ...................................................................
5c_Bdellovibrionales_bacterium_HDAC              ...................................................................
5d_Turneriella_parva_HdaH                        ...................................................................
5d_Leptospira_interrogans_HdaH                   ...................................................................
5d_Leptospira_brenneri_HDAC                      ...................................................................
5e_Archangium_gephyra_HDAC                       EVEKRR.........LGEDTFLFVNWLSLRNPRAQFSDLRPQLPGQEVPGLGLAREFTEILTLMSKR
5e_Nannocystis_exedens_AcuC                      VVRRDRRT.......IPGFELLAIDWLLLQNPREHFSEDKPALPGQRHPGLGLFEETVALLIIACER
5e_Deltaproteobacteria_bacterium_HDAC            ITSRTQITAPPDLGDPPTIDSLVIEWTLLQNPTKQFEPGRPRLPGQNFPGLGLAMLLQEILIRAAHR
5f_Micavibrio_sp_HDAC_domain                     ...................................................................
5f_Homo_sapiens_HDAC11                           ................................SAQN.SDTPLLP.......................
5f_Arabidopsis_thaliana_HDA2                     ...................................................................
5g_Bacteriovorax_stolpii_AcuC-like               ...................................................................
5g_Gracilinema_caldarium_HdaH                    ...................................................................
5g_Bacteriovorax_sp_MedPE-SWde_HDAC_domain       ...................................................................


                                                                        α11
5a_Acinetobacter_wuhouensis_HDAC                 ℓℓℓℓℓℓ.............................................ℓℓℓ.............
                                                    290

5a_Acinetobacter_wuhouensis_HDAC                 FHSAYQ.............................................VYYG............
5a_Neisseriaceae_bacterium_HDAC                  .....................................................IL............
5a_Treponema_sp_HDAC                             ....................................................AQGD...........
5b_Vibrio_cholerae_HdaH                          ...................................................................
5b_Pseudomonas_alcaligenes_AcuC                  ...................................................................
5b_Leptospiraceae_bacterium_HDAC                 ...................................................................
5c_Moraxella_bovis_HDAC                          ...................................................................
5c_Flavobacterium_indicum_HDAC-family            ...................................................................
5c_Bdellovibrionales_bacterium_HDAC              ...................................................................
5d_Turneriella_parva_HdaH                        ...................................................................
5d_Leptospira_interrogans_HdaH                   ...................................................................
5d_Leptospira_brenneri_HDAC                      ...................................................................
5e_Archangium_gephyra_HDAC                       LQLAGVAFRPSWFHMAWAARHDSRFVSPERQGRFEALIRDSRRLSLLQATRAIAEGRVT.LNGKPYQ
5e_Nannocystis_exedens_AcuC                      VGLAGLVVTPSQYHLAVQWQKRLRFVDPRAGARMRAIREALPGASLTEAARALQEGRVVDQSGRTCS
5e_Deltaproteobacteria_bacterium_HDAC            LSLECLLSRPSHYHTAALAGPRYQFLDPRVEGRFRAMRRALAHLAIPVATRIIDEGRLRSKHGEPIP
5f_Micavibrio_sp_HDAC_domain                     ...................................................................
5f_Homo_sapiens_HDAC11                           ...................................................................
5f_Arabidopsis_thaliana_HDA2                     ...................................................................
5g_Bacteriovorax_stolpii_AcuC-like               ...................................................................
5g_Gracilinema_caldarium_HdaH                    ...................................................................
5g_Bacteriovorax_sp_MedPE-SWde_HDAC_domain       ...................................................................


5a_Acinetobacter_wuhouensis_HDAC

5a_Acinetobacter_wuhouensis_HDAC                 ...........................................................E...
5a_Neisseriaceae_bacterium_HDAC                  ...................................G...........................
5a_Treponema_sp_HDAC                             .....RTWAAAAEL.....................D...........................
5b_Vibrio_cholerae_HdaH                          ...................................S................AG.....Y...
5b_Pseudomonas_alcaligenes_AcuC                  ...................................A................QL.....R...
5b_Leptospiraceae_bacterium_HDAC                 ...................................N.................V.....L...
5c_Moraxella_bovis_HDAC                          ...................................K................LL.....A...
5c_Flavobacterium_indicum_HDAC-family            ...........................................................I...
5c_Bdellovibrionales_bacterium_HDAC              ...................................S.......................K...
5d_Turneriella_parva_HdaH                        ...............................................................
5d_Leptospira_interrogans_HdaH                   ...............................................................
5d_Leptospira_brenneri_HDAC                      ...............................................................
5e_Archangium_gephyra_HDAC                       WEADEMVRW.RDEN.....................DIPQDKA.LIVAERE.RSHFEFREK...
5e_Nannocystis_exedens_AcuC                      YEPSPMIFPISDEL.....................KAELDPENYLRAIDEAADEYRFQLSSPR
5e_Deltaproteobacteria_bacterium_HDAC            WEPGDHVLPVSERLTEYFASRVFSAKADEALRDLTN................QG.LVV.V...
5f_Micavibrio_sp_HDAC_domain                     ...............................................................
5f_Homo_sapiens_HDAC11                           ....................................................PA.VP......
5f_Arabidopsis_thaliana_HDA2                     ....................................................PE.........
5g_Bacteriovorax_stolpii_AcuC-like               ....................................................NA.PFHLK...
5g_Gracilinema_caldarium_HdaH                    ...........................................................P...
5g_Bacteriovorax_sp_MedPE-SWde_HDAC_domain       ...................................D................QG.VAR.V...
```

Supplementary Figure 19: Primary sequence alignment of selected bacterial deacylases of cluster 5 (UniProt accession numbers are shown).

Show are the following sequences of the following deacylases representing enzymes of cluster 5 and the sub-clusters (code: sub-cluster, accession number/protein name/spcies): 5a, A0A385C7X8/HDAC/*Acinetobacter wuhouensis*; 5a, A0A3S0JDS2/HDAC/*Neisseriaceae bacterium*; 5a, A0A7X8CU14/HDAC/*Treponema sp.*; 5b, A0A395TF317HDAC/*Vibrio cholerae*; 5b, U2ZNB5/AcuC/*Pseudomonas alcaligenes* (strain ATCC 14909 / DSM 50342 / JCM 20561 / NBRC 14159 / NCIMB 9945 / NCTC 10367 / 1577); 5b, A0A960ZHD4/HDAC/*Leptospiraceae bacterium*; 5c, A0A2Z4RAM2/HDAC/*Moraxella bovis*; 5c, H8XRQ2/HDAC family/*Flavobacterium indicum* (strain DSM 17447 / CIP 109464 / GPTSA100-9); 5c, A0A2H0QAI6/HDAC/*Bdellovibrionales bacterium* CG11_big_fil_rev_8_21_14_0_20_38_13; 5d, I4B797/HDAC Superfamily/*Turneriella parva* (strain ATCC BAA-1111 / DSM 21527 / NCTC 11395 / H) (*Leptospira parva*); 5d, A0A098MZK6/HdaH/*Leptospira interrogans*; 5d, A0A2M9Y5D1/HDAC/*Leptospira brenneri*; 5e, A0A2W5V5Z1/HDAC/*Archangium gephyra*; 5e, A0A1I2D6R7/AcuC/*Nannocystis exedens*; 5e, A0A925AY58/HDAC/*Deltaproteobacteria bacterium*; 5f, A0A2D5Y199/HDAC domain/*Micavibrio sp.*; 5f, Q96DB2/HDAC11/*Homo sapiens*; 5f, Q944K3/HDA2/*Arabidopsis thaliana*; 5g, A0A2K9NTX8/AcuC like/*Bacteriovorax stolpii* (*Bdellovibrio stolpii*); 5g, F8F277/HdaH/*Gracilinema caldarium* (strain ATCC 51460 / DSM 7334 / H1) (*Treponema caldarium*); 5g, A0A1J5KZ05/HDAC domain/*Bacteriovorax sp.* MedPE-SWde All sequences were aligned using the T-COFFEE multiple sequence alignment server[1]. The important catalytic residues are highlighted. The secondary structure elements and numbering is shown for the deacylase shown at the top of the amino acid sequences above the sequence alignment. The alignment was created by ESPript version 3.0[2].

```
                                                                β1                                                  η1
Mycoplana_ramosa_ApaH                                 --........>  ℓ...ℓ................................ℓℓ.....
                                                      1                                                        10
Mycoplana_ramosa_ApaH M......RV........IFSE...D................................HK.....
Pseudomonas_aeruginosa_ApaH2 M......LT........IYSDDHRL................................HH.....
Pseudomonas_aeruginosa_ApaH1 M......LS........VYSD...D................................HR.....
Pseudomonas_sp_M30-35_ApaH M......LT........IYSD...D................................HH.....
Candidatus_Scalindua_brodae_HDAC2 M.....IRIRILRDTGSLVSK...DRVKQIQEIFVQRFPKLVEYAEKIPLLIREPIRHK.YCTT
Burkholderia_pseudomallei_ApaH M......LT........YFH...PD................................QS.....
Ralstonia_insidiosa_ApaH M......LT........FFH...PE................................QL.....
Chloroflexi_bacterium_RBG_16_48_8_HdaH M......QI........VYT...DR.......................................
Fervidobacterium_pennivorans_HDAC MLDGKKNRL........FFCA...D..................................L....
Bordetella_hinzii_ApaH1 M.....HVV........Y.....AD................................QQ.....
Bordetella_bronchiseptica_253_ApaH M......KF........VHS...ET................................HR.....
Spirochaetae_bacterium_HGW-Spirochaetae-5_ApaH M....SFKI......RGVFDDFLPV..........................NRKAIEHN.....
Kosmotoga_pacifica_HDAC M...............KVF......................................YD.....
Chromatiales_bacterium_ApaH M......KA........VYG...EQ................................QA.....
Micavibrio_aeruginosavorus_ApaH M......KT........VYSE...K................................HT.....
Laribacter_hongkongensis_ApaH M......LT........VYSD...T................................HH.....
Pseudomonas_putida_HDAC_family M......KA........FFH...PA................................QR.....
Bacteroidetes_bacterium_GWF2_33_16_HDAC M...LRIRR........IFN...PY................................NK.....
Lentisphaerae_bacterium_GWF2_52_8_HDAC M...FRIRR........IYDSNIPI................................NKRAIAL
```

```
Mycoplana_ramosa_ApaH ....................................ℓ

Mycoplana_ramosa_ApaH ....................................LR..........................
Pseudomonas_aeruginosa_ApaH2 ....................................GR..........................
Pseudomonas_aeruginosa_ApaH1 ....................................LH..........................
Pseudomonas_sp_M30-35_ApaH ....................................LH..........................
Candidatus_Scalindua_brodae_HDAC2 LVVAEGALGRVDGFALVMHFSEINCCFLDFIATRSGLRGRGVGSALYDAVVSEYCRNLSAKGLY
Burkholderia_pseudomallei_ApaH ....................................LH..........................
Ralstonia_insidiosa_ApaH ....................................LH..........................
Chloroflexi_bacterium_RBG_16_48_8_HdaH ...................................HKL..........................
Fervidobacterium_pennivorans_HDAC ................................................................
Bordetella_hinzii_ApaH1 ....................................QQ..........................
Bordetella_bronchiseptica_253_ApaH ....................................RH..........................
Spirochaetae_bacterium_HGW-Spirochaetae-5_ApaH ...............................PVMNKMR..........................
Kosmotoga_pacifica_HDAC ....................................RR..........................
Chromatiales_bacterium_ApaH ....................................EH..........................
Micavibrio_aeruginosavorus_ApaH ....................................LH..........................
Laribacter_hongkongensis_ApaH ....................................EQ..........................
Pseudomonas_putida_HDAC_family ....................................LH..........................
Bacteroidetes_bacterium_GWF2_33_16_HDAC .................................ANIRK..........................
Lentisphaerae_bacterium_GWF2_52_8_HDAC VQQILREQFVLIRPADIDKLPDLLKNPLKYQFRSILFV..........................
```

```
                                                                                      β2
Mycoplana_ramosa_ApaH                                                         -.....................

Mycoplana_ramosa_ApaH ...................NA...................KTE.....................
Pseudomonas_aeruginosa_ApaH2 ...................H......................E.....................
Pseudomonas_aeruginosa_ApaH1 ...................FG...................QSE.....................
Pseudomonas_sp_M30-35_ApaH ...................HG...................SCE.....................
Candidatus_Scalindua_brodae_HDAC2 LEVQPDLPELTTDHAELKQAQ...............IAVRFYE.................L...
Burkholderia_pseudomallei_ApaH ...................H...................PRTY......F...........S..
Ralstonia_insidiosa_ApaH ...................H...................PQSY......Y...........S..
Chloroflexi_bacterium_RBG_16_48_8_HdaH .......................................HNTD...QVLL...........E..
Fervidobacterium_pennivorans_HDAC ...................NE...............YFVPRKE......I..............
Bordetella_hinzii_ApaH1 ...................H..................QPEHF......M...........V..
Bordetella_bronchiseptica_253_ApaH ...................D...................PQFF......I...........V..
Spirochaetae_bacterium_HGW-Spirochaetae-5_ApaH ...............................YYFFVAENNKGEILGFAQMSYAIDLKFCFLDLL
Kosmotoga_pacifica_HDAC ...................HL...............LHSPQKE.................L...
Chromatiales_bacterium_ApaH ...................D...................PQFF......L...........V..
Micavibrio_aeruginosavorus_ApaH ...................HP...................GLE.....................
Laribacter_hongkongensis_ApaH ...................AG...................KAE.....................
Pseudomonas_putida_HDAC_family ...................H...................PRSY......L...........S..
Bacteroidetes_bacterium_GWF2_33_16_HDAC .................IERIKEIIQIQFPGISQKKINKLESE......LLDPIKHKYQSMLFV
Lentisphaerae_bacterium_GWF2_52_8_HDAC .................AENIK...................GE......I..............
```

```
α1                                                          β3
Mycoplana_ramosa_ApaH ...-.>T..T   -....................->
                           20
Mycoplana_ramosa_ApaH ...L.YG..G...E....................LV............P...............
Pseudomonas_aeruginosa_ApaH2 ...L.IG..G...Q....................FT............P...............
Pseudomonas_aeruginosa_ApaH1 ...L.VD..G...K....................LQ............P...............
Pseudomonas_sp_M30-35_ApaH ...LIDG..K...L....................M.............P...............
Candidatus_Scalindua_brodae_HDAC2 .....HG..V...R....................VIENSAYSVPVGNPPTTAILLFDGLGITHT
Burkholderia_pseudomallei_ApaH ......R..G...R....................MR............M...............
Ralstonia_insidiosa_ApaH ......R..G...K....................MR............T...............
Chloroflexi_bacterium_RBG_16_48_8_HdaH ......G..N...P....................FI............T...............
Fervidobacterium_pennivorans_HDAC .....DN..G...K....................FI...........R................
Bordetella_hinzii_ApaH1 ......A..G...R....................VR............................
Bordetella_bronchiseptica_253_ApaH ......R..G...Q....................IT............R...............
Spirochaetae_bacterium_HGW-Spirochaetae-5_ApaH ANNNKKDTGG...IGGALYKRVRDEAIHLNASGLFI............E...............
Kosmotoga_pacifica_HDAC .....DN..G...E....................WI............................
Chromatiales_bacterium_ApaH ......K..G...Q....................PR............R...............
Micavibrio_aeruginosavorus_ApaH ...L.ME..G...R....................FV............E...............
Laribacter_hongkongensis_ApaH ...L.IN..G...T....................LM............P...............
Pseudomonas_putida_HDAC_family ......R..G...Q....................MR............Q...............
Bacteroidetes_bacterium_GWF2_33_16_HDAC AEDINDAIRGFAIL....................LY............M...............
Lentisphaerae_bacterium_GWF2_52_8_HDAC .....SG....FAL....................LL............................
```

```
Mycoplana_ramosa_ApaH                                                                       ..............
                                                                                          30
Mycoplana_ramosa_ApaH                            ....P..F.E..........................APFR..............
Pseudomonas_aeruginosa_ApaH2                     ....C..F.E..........................KPSR..............
Pseudomonas_aeruginosa_ApaH1                     ....C..F.E..........................MPSR..............
Pseudomonas_sp_M30-35_ApaH                       ....C..F.E..........................MPKR..............
Candidatus_Scalindua_brodae_HDAC2                LAR.....REAQEAIKMILTTRFAKTTDTGYIDQVIESFRDDPVTFR..............
Burkholderia_pseudomallei_ApaH                   .....PQ.E...........................VPER..............
Ralstonia_insidiosa_ApaH                         .....PQ.E...........................VPDR..............
Chloroflexi_bacterium_RBG_16_48_8_HdaH           .....D..E...........................VPQR..............
Fervidobacterium_pennivorans_HDAC                ...NP....E.N.........................PSR..............
Bordetella_hinzii_ApaH1                          ......PNPD...........................VPAR..............
Bordetella_bronchiseptica_253_ApaH               ......ST.E...........................QPER..............
Spirochaetae_bacterium_HGW-Spirochaetae-5_ApaH   ..CMSDN..PE.....................FFKDKSVLKQSRNRLKFYERFGANPIVN
Kosmotoga_pacifica_HDAC                          .EN......PEK..........................PMR..............
Chromatiales_bacterium_ApaH                      .....ST.E...........................QPER..............
Micavibrio_aeruginosavorus_ApaH                  ....N..F.E...........................KPER..............
Laribacter_hongkongensis_ApaH                    ....C..F.E...........................NPSR..............
Pseudomonas_putida_HDAC_family                   .....PQ.E...........................VPAR..............
Bacteroidetes_bacterium_GWF2_33_16_HDAC          ....PDH..KL...................CYLDLIAVSPEK.........A.S...
Lentisphaerae_bacterium_GWF2_52_8_HDAC           ......H..AP...................DLAFSYLDYLST.........A.KHFT
```

```
                                                         α2
Mycoplana_ramosa_ApaH                            .............................................
                                                                                          40
Mycoplana_ramosa_ApaH                            ....AEWI...........LAA......................VK.EAGF......
Pseudomonas_aeruginosa_ApaH2                     ....ADMV...........LDR......................VK.AVGL......
Pseudomonas_aeruginosa_ApaH1                     ....ADTV...........LAR......................VK.SQNL......
Pseudomonas_sp_M30-35_ApaH                       ....ADHI...........LQR......................VK.DRNI......
Candidatus_Scalindua_brodae_HDAC2                ...PLRYI...........KAVKENRIIRDFNSPLAYTFVFSPKHEIHHVK.ERGYFE.....
Burkholderia_pseudomallei_ApaH                   ....AARL...........VAA......................AF.AMGF.P.....
Ralstonia_insidiosa_ApaH                         ....ARNL...........LQA......................AR.DLGF.T.....
Chloroflexi_bacterium_RBG_16_48_8_HdaH           ....VEVLL...........QA......................F...SAA.G.....
Fervidobacterium_pennivorans_HDAC                ...L.KLVL...........NY......................LQ.EN.F.Q.....
Bordetella_hinzii_ApaH1                          ....LAGL.......REGVAA.........................MGY.A.....
Bordetella_bronchiseptica_253_ApaH               ....ADRL...........LAA......................AQ.RRGH.E.....
Spirochaetae_bacterium_HGW-Spirochaetae-5_ApaH   PEYERESLLFYGNPYFLLFDNLG......................IKKDLSL.NDSRKI
Kosmotoga_pacifica_HDAC                          ...I.ESI...........RER......................ME.NF..FG.....
Chromatiales_bacterium_ApaH                      ....AQRL...........LAG......................VA.RCGL.E.....
Micavibrio_aeruginosavorus_ApaH                  .......V...........FF.......................VR.DQIL....HRG
Laribacter_hongkongensis_ApaH                    ....ADMV...........REA......................V..DAA.......
Pseudomonas_putida_HDAC_family                   ....IEPL...........LAV......................VN.KLGY.P.....
Bacteroidetes_bacterium_GWF2_33_16_HDAC          .SGLGGAIY...........TR......................VR.EEAELLNANGL
Lentisphaerae_bacterium_GWF2_52_8_HDAC           GRGVGGALY...........ER......................VR.EEVLMLKSKGL
```

```
                                                               β4
Mycoplana_ramosa_ApaH                                          ─.►
                                                                   50
Mycoplana_ramosa_ApaH                            .D...........DV.V..A..P..................ARH.GLE...........TV
Pseudomonas_aeruginosa_ApaH2                     G............EV.R..A..P..................RDF.GLE...........PI
Pseudomonas_aeruginosa_ApaH1                     G............EV.I..A..P..................KDF.GRE...........PL
Pseudomonas_sp_M30-35_ApaH                       G............EI.R..E..P..................QDF.GRD...........PI
Candidatus_Scalindua_brodae_HDAC2                .RPIRVETIRSVIRDVGGFVSTVP..................REY.GE..........K.WI
Burkholderia_pseudomallei_ApaH                   ..............V.R..E..P..................DDF.GI..........A.PI
Ralstonia_insidiosa_ApaH                         ..............I.A..Q..P..................EDH.GL..........A.PL
Chloroflexi_bacterium_RBG_16_48_8_HdaH           WD............ST.L..Q..P............T......DH.GL..........G.PI
Fervidobacterium_pennivorans_HDAC                ...............A...IQ..P............F......EF.GEE..........H.I
Bordetella_hinzii_ApaH1                          ..............I.H..A..P..................EDF.GL..........Q.YL
Bordetella_bronchiseptica_253_ApaH               ..............RV......E..................ARDFG............E.AI
Spirochaetae_bacterium_HGW-Spirochaetae-5_ApaH   FR............AV.L..E..R.............KHERDVNDN.GLKKFISSMKGDPQ.II
Kosmotoga_pacifica_HDAC                          .............YT....IQ..E..................SEY.HF..........ESYI
Chromatiales_bacterium_ApaH                      ...............V.V..T..P..................DEF.GA..........S.PR
Micavibrio_aeruginosavorus_ApaH                  FS............EI.I..A..P..................EEF..............PA
Laribacter_hongkongensis_ApaH                    GF............SR.I..G..P..................TDH.GKE...........PI
Pseudomonas_putida_HDAC_family                   ...............L.F..E..P..................ADH.GL..........A.PL
Bacteroidetes_bacterium_GWF2_33_16_HDAC          FF............EC.LPDD..PALCKDGDILLQNQKRLAFYESF.GA..........R.PI
Lentisphaerae_bacterium_GWF2_52_8_HDAC           FF............EC.LPDD..PLLCKSPLVLRQNAQRLKFYEKY.GA..........R.PI
```

```
                                                      η2
Mycoplana_ramosa_ApaH                            .....................................................
                                                        60                                            70
Mycoplana_ramosa_ApaH                            .....LK.VHD...........AG...................YL..N.......F.....
Pseudomonas_aeruginosa_ApaH2                     .....RR.VHS...........EG...................FV..R.......F.....
Pseudomonas_aeruginosa_ApaH1                     .....LR.LHD...........AA...................YL..D.......F.....
Pseudomonas_sp_M30-35_ApaH                       .....QR.IHT...........AE...................YL..N.......F.....
Candidatus_Scalindua_brodae_HDAC2                L.....S.VHD...........KT...................FV........HY.....
Burkholderia_pseudomallei_ApaH                   A.....A.VHD...........TH...................YL..R.......F.....
Ralstonia_insidiosa_ApaH                         T.....A.VHG...........EP...................YL..R.......F.....
Chloroflexi_bacterium_RBG_16_48_8_HdaH           L.....D.VHD...........SD...................YV..D.......F.....
Fervidobacterium_pennivorans_HDAC                L.....K.VHS...........EK...................YF..N.......Y.....
Bordetella_hinzii_ApaH1                          S.....R.VHT...........AR...................YL..D.......F.....
Bordetella_bronchiseptica_253_ApaH               L....AE.VHC...........PR...................YL..D.......F.....
Spirochaetae_bacterium_HGW-Spirochaetae-5_ApaH   R.....KSVHSNDDFNHYSFTVPADRKIPLLYNDGHEIHHIRDKGYV..ESPVRIKSI.....
Kosmotoga_pacifica_HDAC                          Y.....L.IHE...........PE...................YV........SW.....
Chromatiales_bacterium_ApaH                      A.....A.VHD...........AT...................YL..D.......F.....
Micavibrio_aeruginosavorus_ApaH                  ..DIFSR.VHD...........PR...................YV..A.......F.....
Laribacter_hongkongensis_ApaH                    .....LA.VHR...........EN...................YV..R.......F.....
Pseudomonas_putida_HDAC_family                   A.....A.VHG...........QA...................YL..D.......Y.....
Bacteroidetes_bacterium_GWF2_33_16_HDAC          ANTLYEMKVNP...........ED...............DCPPYL...VYDGLNKFDTLPAK
Lentisphaerae_bacterium_GWF2_52_8_HDAC           .......INTKYETPVKEGGDNAP...................YLVVDNLGTERSF...KKS
```

```
                                                                           α4
Mycoplana_ramosa_ApaH                             .........ℓℓℓ........................ℓ.ℓℓℓ..........ℓℓ...........

Mycoplana_ramosa_ApaH                             .........LET........................A.WDR..........WK...........
Pseudomonas_aeruginosa_ApaH2                      .........LQN........................A.WQD..........WL...........
Pseudomonas_aeruginosa_ApaH1                      .........LQG........................A.WAR..........WT...........
Pseudomonas_sp_M30-35_ApaH                        .........FEG........................A.WAR..........WQ...........
Candidatus_Scalindua_brodae_HDAC2                 .........LRT........................V...........................
Burkholderia_pseudomallei_ApaH                    .........LET........................V.HRE..........WK...........
Ralstonia_insidiosa_ApaH                          .........LQD........................A.HTD..........WR...........
Chloroflexi_bacterium_RBG_16_48_8_HdaH            .........LQT........................V.YEK..........NK...........
Fervidobacterium_pennivorans_HDAC                 .........LVK........................................K...........
Bordetella_hinzii_ApaH1                           .........LEH........................A.HAL..........WS...........
Bordetella_bronchiseptica_253_ApaH                .........LKN........................G.YAQ..........WR...........
Spirochaetae_bacterium_HGW-Spirochaetae-5_ApaH    .........LK............VIEKSELFIKSKTSRYSERHITAVHDKDFV...........
Kosmotoga_pacifica_HDAC                           .........LKK........................K.SNE.......................
Chromatiales_bacterium_ApaH                       .........LDC........................A.FSE..........WS...........
Micavibrio_aeruginosavorus_ApaH                   .........LKE........................G.YRR..........WE...........
Laribacter_hongkongensis_ApaH                     .........LET........................F.WER..........WS...........
Pseudomonas_putida_HDAC_family                    .........LGS........................A.YQQ..........WH...........
Bacteroidetes_bacterium_GWF2_33_16_HDAC           TLKGIIKAILERKYGDYCPESYIKMVVNSVKDNPFLLRQPK..........YV...........
Lentisphaerae_bacterium_GWF2_52_8_HDAC            RMRQIVKAILERKYPDLC....................NEE..........YVDMVINSISDDP


Mycoplana_ramosa_ApaH                             ........ℓ
                                                         80
Mycoplana_ramosa_ApaH                             ........AAG.Y........................K......G........EA...I.....
Pseudomonas_aeruginosa_ApaH2                      ........ATG.R........................S......H........DM...L.....
Pseudomonas_aeruginosa_ApaH1                      ........AEG.H........................S......G........DL...V.....
Pseudomonas_sp_M30-35_ApaH                        ........EQD.G........................T......G........DL...L.....
Candidatus_Scalindua_brodae_HDAC2                 ........CAS.L..........................RQG...........RP...VY....
Burkholderia_pseudomallei_ApaH                    ........AM.PE..............................DW........GD.........
Ralstonia_insidiosa_ApaH                          ........RL.PE..............................DW........GD.........
Chloroflexi_bacterium_RBG_16_48_8_HdaH            ........SYY.Q..............................LA.............EP....
Fervidobacterium_pennivorans_HDAC                 ........SQE.V........................E......D........EY...L.....
Bordetella_hinzii_ApaH1                           ........RL..P..............................NA........GP.........
Bordetella_bronchiseptica_253_ApaH                ........ALPGA..............................SQ........EI.........
Spirochaetae_bacterium_HGW-Spirochaetae-5_ApaH    ........KFF.K..............................TA........SA..SV.....
Kosmotoga_pacifica_HDAC                           .......IESG.K........................................EY...FP....
Chromatiales_bacterium_ApaH                       ........AL.PE..............................ST........A..........
Micavibrio_aeruginosavorus_ApaH                   ............E........................Q......G........ESHLML.....
Laribacter_hongkongensis_ApaH                     ........RPS.R........................RSATGRDY........DA...L.....
Pseudomonas_putida_HDAC_family                    ........EV.PE..............................DW........GD.........
Bacteroidetes_bacterium_GWF2_33_16_HDAC           ........KSR.K.ISSINKNIKEKNKILLIVNNKHAIHHIKERGYVESPVRVES.........
Lentisphaerae_bacterium_GWF2_52_8_HDAC            VQLRPFKYVSN.KDIEPISRRLPVDTLIPLVYNTQHIVHHVQERG......YVESPVRVETMLS


Mycoplana_ramosa_ApaH

Mycoplana_ramosa_ApaH                             ..........................................................A.....
Pseudomonas_aeruginosa_ApaH2                      ..........................................................P.....
Pseudomonas_aeruginosa_ApaH1                      ..........................................................S.....
Pseudomonas_sp_M30-35_ApaH                        ..........................................................P.....
Candidatus_Scalindua_brodae_HDAC2                 ..........................................................P.....
Burkholderia_pseudomallei_ApaH                    .......................................E..................A.....
Ralstonia_insidiosa_ApaH                          .......................................E..................V.....
Chloroflexi_bacterium_RBG_16_48_8_HdaH            ................................................................
Fervidobacterium_pennivorans_HDAC                 ..........................................................P.....
Bordetella_hinzii_ApaH1                           .......................................E..................V.....
Bordetella_bronchiseptica_253_ApaH                .......................................T..................P.....
Spirochaetae_bacterium_HGW-Spirochaetae-5_ApaH    ..........................................................P.AGVS
Kosmotoga_pacifica_HDAC                           ..........................................................E.....
Chromatiales_bacterium_ApaH                       .......................................E..................V.....
Micavibrio_aeruginosavorus_ApaH                   ..........................................................P.....
Laribacter_hongkongensis_ApaH                     ..........................................................P.....
Pseudomonas_putida_HDAC_family                    .......................................E..................V.....
Bacteroidetes_bacterium_GWF2_33_16_HDAC           .......................................IQRELDKTGFFHQDNALEYPERFIL
Lentisphaerae_bacterium_GWF2_52_8_HDAC            ALNKTGLFNNIRERHFAEHHITSLHDRDFVDYLKKVCSAI...................PDKES


                                                                                                   α5
Mycoplana_ramosa_ApaH                                          T.......................T         ℓℓℓℓℓℓℓ     TT
                                                      90                                   100         110
Mycoplana_ramosa_ApaH                             ....TS.FP...VR.......................RTSPRIP..TDIEGQIGYYCN.AAETA
Pseudomonas_aeruginosa_ApaH2                      ....IA.WP...TR.......................RLRQTEP..DNIDGRLGYYSF.DAGAP
Pseudomonas_aeruginosa_ApaH1                      ....TT.FPGRRLR.......................RDGPI.P..TALMGELGYYSF.DTEAP
Pseudomonas_sp_M30-35_ApaH                        ....YT.WP...AR.......................TLSQRLP..TSLHGQLGYYSF.DAGAP
Candidatus_Scalindua_brodae_HDAC2                 ....DT.FPI...............R...........RPDNR.P..KELPVQAGYYC...IDTG
Burkholderia_pseudomallei_ApaH                    ....MS.NIF...............V...........REPNA.L..RGVLAQAARHLA.DGSCP
Ralstonia_insidiosa_ApaH                          ....MS.NIF...............V...........REPNA.L..RGVLAQAARYLA.DGSCP
Chloroflexi_bacterium_RBG_16_48_8_HdaH            ....VFTWA.............FATR.........YTGHK...P..KHFLGQLGYYAF.GWGTP
Fervidobacterium_pennivorans_HDAC                 ....EV.FFV...............D...........QIFDT.G..TPIKKEI.FF...AAKRA
Bordetella_hinzii_ApaH1                           ....VP.NAH...............P...........RRPDA.PYPRGIVGRAGFHMY.DLACA
Bordetella_bronchiseptica_253_ApaH                ....HV.HPP...............R...........EESGY.P..VSIIGRAGFHI...ADTS
Spirochaetae_bacterium_HGW-Spirochaetae-5_ApaH    LYPDTF.PIR.............KN.............VKP..P..KKLVSRAGYYCI.DIYSP
Kosmotoga_pacifica_HDAC                           ....VF...G...............H.....................D...KV..FD...TGTP
Chromatiales_bacterium_ApaH                       ....IA.NVH...............P.........MHRRTNY.P..HSIIGRAGWHMA.DTACP
Micavibrio_aeruginosavorus_ApaH                   ....SV.FN...LQ.......................NFDAPEP..RALYGQMGYYIADGA.AP
Laribacter_hongkongensis_ApaH                     ....LI.WP...TR.......................CFRQVEP..EDIDGQLGYFSM.DAGTP
Pseudomonas_putida_HDAC_family                    ....MS.NIY...............I...........REGNP.L..RGILGKTARYLA.DGSCP
Bacteroidetes_bacterium_GWF2_33_16_HDAC           KVHDRD.YVN...YFKKVCKNLSKGKSVYPYVFPIRNGTKA..P..KELSVRAGYYCF.DTFTP
Lentisphaerae_bacterium_GWF2_52_8_HDAC            VYPYVF.PI..............RN............NTRP..P..KDLPMRAGYYCI.DTFTP
```

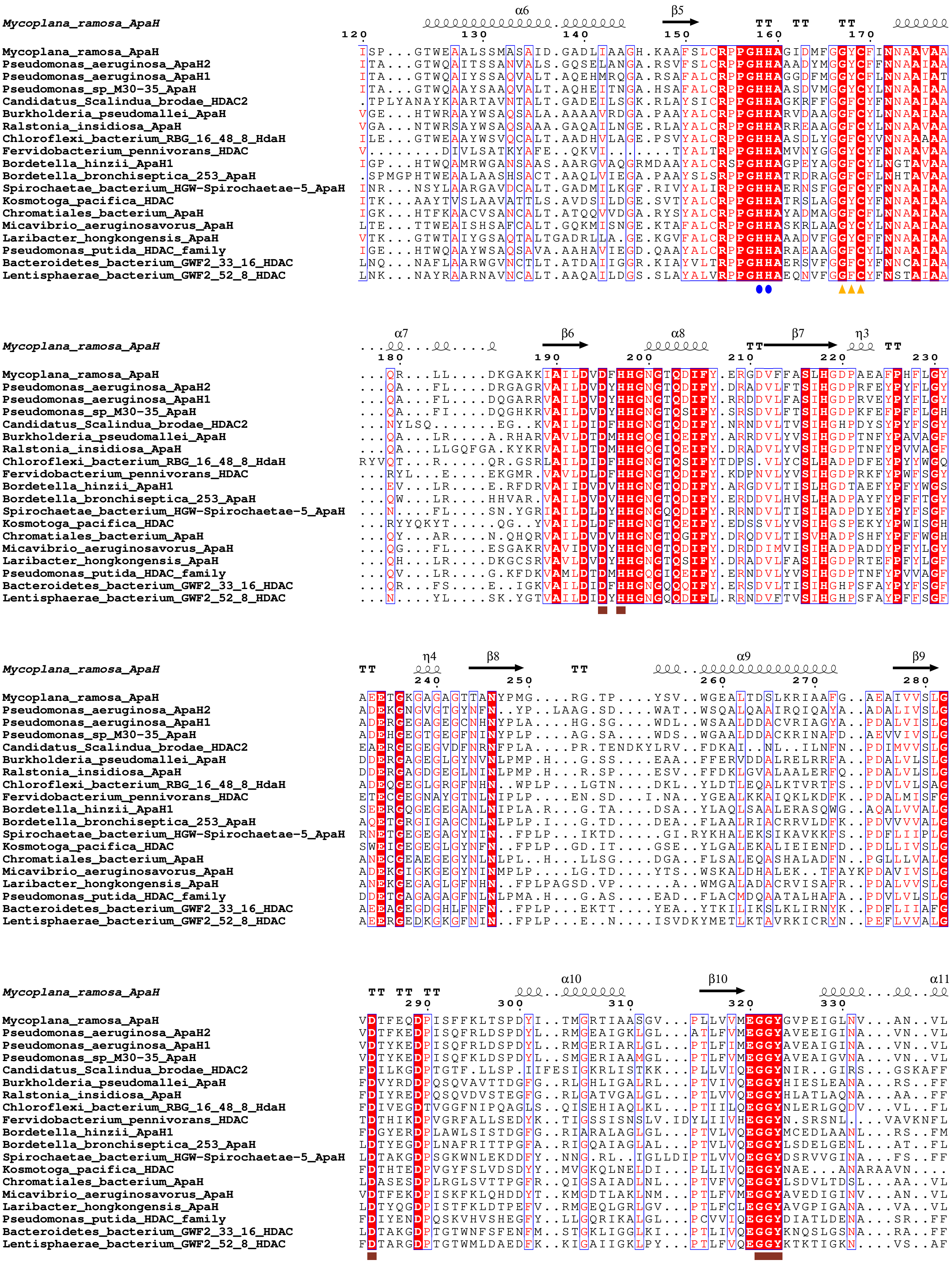
Mycoplana_ramosa_ApaH
α6 β5 TT TT TT
120 130 140 150 160 170
Mycoplana_ramosa_ApaH ISP...GTWEAALSSMASAID.GADLIAAGH.KAAFSLCRPPGHHAGIDMFGGYCFINNAAVAA
Pseudomonas_aeruginosa_ApaH2 ITA...GTWQAITSSANVALS.GQSELANGA.RSVFSLCRPPGHHAAADYMGGYCFFNNAAIAA
Pseudomonas_aeruginosa_ApaH1 ITA...GTWQAIYSSAQVALT.AQEHMRQGA.RSAFALCRPPGHHAGGDFMGGYCFLNNAAIAT
Pseudomonas_sp_M30-35_ApaH ITA...GTWQAAYSAAQVALT.AQHEITNGA.HSAFALCRPPGHHAASDVMGGYCYLNNAAIAA
Candidatus_Scalindua_brodae_HDAC2 .TPLYANAYKAARTAVNTALT.GADEILSGK.RLAYSICRPPGHHAGKRFFGGFCYFNNAAIAA
Burkholderia_pseudomallei_ApaH VGE...HTWRAAYWSAQSALA.AAAAVRDGA.PAAYALCRPPGHHARVDAAGGFCYLNNAAIAA
Ralstonia_insidiosa_ApaH VGA...NTWRSAYWSAQSAAA.GAQAILNGE.RLAYALCRPPGHHARREAAGGFCYLNNAAVAA
Chloroflexi_bacterium_RBG_16_48_8_HdaH ILE...GTWEAAYWSVQCALT.AADHVLAGE.PSVYALCRPPGHHAASDLYGGFCYVNNAAAAA
Fervidobacterium_pennivorans_HDAC V........DIVLSATKYAFE..QKVI.......TYALTRPPGHHAMVNYGGGYCYFNNAAVAA
Bordetella_hinzii_ApaH1 IGP...HTWQAMRWGANSAAS.AARGVAQGRMDAAYALCRSPGHHAGPEYAGGFCYLNGTAVAA
Bordetella_bronchiseptica_253_ApaH .SPMGPHTWEAALAASHSACT.AAQLVIEGA.PAAYSLSRPPGHHATRDRAGGFCFLNHTAVAA
Spirochaetae_bacterium_HGW-Spirochaetae-5_ApaH INR...NSYLAARGAVDCALT.GADMILKGF.RIVYALIRPPGHHAERNSFGGFCYFNNAAIAA
Kosmotoga_pacifica_HDAC ITK...AAYTVSLAAVATTLS.AVDSILDGE.SVTYALCRPPGHHATRSLAGGYCYFNNAAIAA
Chromatiales_bacterium_ApaH IGK...HTFKAACVSANCALT.ATQQVVDGA.RYSYALCRPPGHHAYADMAGGFCFLNNAAIAA
Micavibrio_aeruginosavorus_ApaH LTE...TTWEAISHSAFCALT.GQKMISNGE.KTAFALCRPPGHHASKRLAAGYCYLNNAAIAA
Laribacter_hongkongensis_ApaH VTK...GTWTAIYGSAQTALTGADRLLA.GE.KGVFALCRPPGHHAAADVFGGYCFFNNAAIAA
Pseudomonas_putida_HDAC_family IGE...HTWQAAYWSAQSAVA.AAHAVIEGD.QAAYALCRPPGHHARAEAAGGFCYLNNAAIAA
Bacteroidetes_bacterium_GWF2_33_16_HDAC LNQ...NAFLAARWGVNCTLT.ATDAIIGGR.KIAYVLTRPPGHHAERSVFGGFCYFNNCAIAA
Lentisphaerae_bacterium_GWF2_52_8_HDAC LNK...NAYRAARNAVNCALT.AAQAILDGS.SLAYALVRPPGHHAEQNVFGGFCYFNSTAIAA
Mycoplana_ramosa_ApaH
α7 β6 α8 β7 η3
TT TT
180 190 200 210 220 230
Mycoplana_ramosa_ApaH ...QR...LL....DKGAKKIAILDVDFHHGNGTQDIFY.ERGDVFFASLHGDPAEAFPHFLGY
Pseudomonas_aeruginosa_ApaH2 ...QA...FL....DRGAGRVAILDVDYHHGNGTQDIFY.DRADVLFTSIHGDPRFEYPYFLGY
Pseudomonas_aeruginosa_ApaH1 ...QA...FL....DQGARRVAILDVDYHHGNGTQDIFY.RRDDVLFASIHGDPRVEYPYFLGY
Pseudomonas_sp_M30-35_ApaH ...QA...FI....DQGHKRVAILDVDYHHGNGTQSIFY.SRSDVLFTSIHGDPKFEFPFFLGH
Candidatus_Scalindua_brodae_HDAC2 ...NYLSQ.......EG..KVAILDIDFHHGNGTQDIFY.NRNDVLTVSIHGHPDYSYPYFSGY
Burkholderia_pseudomallei_ApaH ...QA...LR....A.RHARVAVLDTDMHHGQGIQEIFY.ARRDVLYVSIHGDPTNFYPAVAGF
Ralstonia_insidiosa_ApaH ...QA...LLGQFGA.KYKRVAILDTDMHHGQGIQEIFY.DRADVLYVSIHGDPTNFYPVVAGF
Chloroflexi_bacterium_RBG_16_48_8_HdaH RYVQT...R.....QR.GSRLAILDIDFHHGNGTQSIFYTDPS.VLYCSLHADPDFEYPYYWGQ
Fervidobacterium_pennivorans_HDAC ...RYL...E....EKGMR.VAVLDLDFHHGNGTQDIFY.KDPNVLYVSIHGDPRKFYPWFSGY
Bordetella_hinzii_ApaH1 ...EV...LR....E.RFDRVAIIDVDVHHGNGTQDIFY.ARGDVLTISLHGDTAEFYPFYWGS
Bordetella_bronchiseptica_253_ApaH ...QW...LR....HHVAR.VAILDVDVHHGNGTQDIFY.ERDDVLHVSLHADPAYFYPFFTGY
Spirochaetae_bacterium_HGW-Spirochaetae-5_ApaH ...N....FL....SN.YGRIAILDLDYHHGNGQQDIFY.RRNDVLTISIHADPDYEYPYFSGF
Kosmotoga_pacifica_HDAC ...RYYQKYT.....QG..YVAILDLDFHHGNGTQEIFY.EDSSVLYVSIHGSPEKYYPWISGH
Chromatiales_bacterium_ApaH ...QY...AR....N.QHQRVAILDVDVHHGNGTQGIFY.DRQDVLTISVHADPSHFYPFFWGH
Micavibrio_aeruginosavorus_ApaH ...QG...FL....ESGAKRVAVIDVDYHHGNGTQDIFY.DRDDIMVISIHADPADDYPFYLGY
Laribacter_hongkongensis_ApaH ...QH...LR....DKGCSRVAVLDVDYHHGNGTQSIFY.DRADVLFASIHGDPRTEFPFYLGF
Pseudomonas_putida_HDAC_family ...QV...LR....G.KFDKVAMLDTDMHHGQGIQEIFY.ERNDVLYVSIHGDPTNFYPVVAGF
Bacteroidetes_bacterium_GWF2_33_16_HDAC ...QR...FS....E..IGKVAILDIDFHHGNGQQQIFY.ERSDVLTISIHGHPSFAYPYFSGF
Lentisphaerae_bacterium_GWF2_52_8_HDAC ...N....YL....SK.YGTVAILDIDYHHGNGQQDIFL.RRNDVFTVSIHGHPSFAYPFFSGF
Mycoplana_ramosa_ApaH
η4 β8 α9 β9
TT TT
240 250 260 270 280
Mycoplana_ramosa_ApaH AEETGKGAGAGTTANYPMG....RG.TP....YSV..WGEALTDSLKRIAAFG..AEAIVVSLG
Pseudomonas_aeruginosa_ApaH2 ADEKGNGVGTGYNFN..YP..LAAG.SD....WAT..WSQALQAAIRQIQAYA..ADALIVSLG
Pseudomonas_aeruginosa_ApaH1 ADERGEGAGEGCNHNYPLA....HG.SG....WDL..WSAALDDACVRIAGYA..PDALVISLG
Pseudomonas_sp_M30-35_ApaH ADEHGEGTGEGFNINYPLP....AG.SA....WDS..WGAALDDACKRINAFD..AEVVIVSLG
Candidatus_Scalindua_brodae_HDAC2 EAERGEGEGVDFNRNFPLA....PR.TENDKYLRV..FDKAI..NL..ILNFN..PDIMVVSLG
Burkholderia_pseudomallei_ApaH DDERGAGEGLGYNVNLPMP.H...G.SS....EAA..FFERVDDALRELRRFA..PDALVLSLG
Ralstonia_insidiosa_ApaH DDERGAGDGEGLNINLPMP.H...R.SP....ESV..FFDKLGVALAALERFQ..PDALVLSLG
Chloroflexi_bacterium_RBG_16_48_8_HdaH ADEQGEGLGRGFNHN..WPLP...LGTN....DKL..YLDTLEQALKTVRTFS..PDVLVLSAG
Fervidobacterium_pennivorans_HDAC ETECGEGNAYGTNLNIPLP....EN.SD...I.NA..YGEALKKAIQKLKDFK..PDALMISFG
Bordetella_hinzii_ApaH1 SEERGQGEGEGANLNIPLA.R...G.TA....DSA..YLAQLSAALERASQWG..AQALVVALG
Bordetella_bronchiseptica_253_ApaH AQETGRGIGAGCNLNLPLP.I...G.TD....DEA..FLAALRIACRRVLDFK..PDVVVVALG
Spirochaetae_bacterium_HGW-Spirochaetae-5_ApaH RNETGEGEGAGYNIN..FPLP...IKTD.....GI.RYKHALEKSIKAVKKFS..PDFLIIPLG
Kosmotoga_pacifica_HDAC SWEIGEGEGLGYNFN..FPLP..GD.IT....GSE..YLGALEKALIEIENFD..PDLLIVSLG
Chromatiales_bacterium_ApaH ANECGEAEGEGYNLNLPL..H...LLSG....DGA..FLSALEQASHALADFN..PGLLLVALG
Micavibrio_aeruginosavorus_ApaH ADEKGIGKGEGYNINMPLP....LG.TD....YTS..WSKALDHALEK..TFAYKPDAVIVSLG
Laribacter_hongkongensis_ApaH ANEKGEGAGLGFNHN..FPLPAGSD.VP......A..WMGALADACRVISAFR..PDALVVSLG
Pseudomonas_putida_HDAC_family DDETGAGAGAGFNLNLPMA.H...G.AS....EAD..FLACMDQAATALHAFA..PDVLVLSLG
Bacteroidetes_bacterium_GWF2_33_16_HDAC AEEAGEGDGHLFNFN..FPLP...EKTT....YEA..YTKILIKSLKLIRNYK..PDFLIIAFG
Lentisphaerae_bacterium_GWF2_52_8_HDAC AEERGEDKGKGFNIN..FPLP...E..N....ISVDKYMETLKTAVRKICRYN..PEFLVVALG
Mycoplana_ramosa_ApaH
α10 β10 α11
TT TT TT
290 300 310 320 330
Mycoplana_ramosa_ApaH VDTFEQDPISFFKLTSPDYI..TMGRTIAASGV...PLLVVMEGGYGVPEIGLNV...AN..VL
Pseudomonas_aeruginosa_ApaH2 VDTFKEDPISQFRLDSPDYL..RMGEAIGKLGL...ATLFVMEGGYAVEEIGINA...VN..VL
Pseudomonas_aeruginosa_ApaH1 VDTYKEDPISQFRLDSPDYL..RMGERIARLGL...PTLFIMEGGYAVEAIGINA...VN..VL
Pseudomonas_sp_M30-35_ApaH VDTYKEDPISQFKLDSPDYL..SMGERIAAMGL...PTLFVMEGGYAVEAIGVNA...VN..VL
Candidatus_Scalindua_brodae_HDAC2 FDILKGDPTGTF.LLSP.IIFESIGKRLISTKK...PLLVIQEGGYNIR..GIRS...GSKAFF
Burkholderia_pseudomallei_ApaH FDVYRDDPQSQVAVTTDGFG..RLGHLIGALRL...PTVIVQEGGYHIESLEANA...RS..FF
Ralstonia_insidiosa_ApaH FDIYREDPQSQVDVSTEGFG..RLGATVGALGL...PTLVVQEGGYHLATLAQNA...AA..FF
Chloroflexi_bacterium_RBG_16_48_8_HdaH FDIVEGDTVGGFNIPQAGLS..QISEHIAQLKL...PTIILQEGGYNLERLGQDV...VL..FL
Fervidobacterium_pennivorans_HDAC TDTHIKDPVGRFALLSEDYK..TIGSSISNSLV.IDYLIIVHEGGYN.SRSNL....VAVKNFL
Bordetella_hinzii_ApaH1 FDGYERDPLAWLSISTDGFG..RIARALAGLGL...PTVLVQEGGYMCEDLAANL...RS..FM
Bordetella_bronchiseptica_253_ApaH LDTYEGDPLNAFRITTPGFA..RIGQAIAGLAL...PTVLVQEGGYLSDELGENL...AT..FL
Spirochaetae_bacterium_HGW-Spirochaetae-5_ApaH LDTAKGDPSGKWNLEKDDFY..NNG.RL..IGLLDIPTLVVQEGGYDSRVVGINA...FS..FF
Kosmotoga_pacifica_HDAC FDTHTEDPVGYFSLVDSDYY..MVGKQLNELDI...PLLIVQEGGYNAE...ANARAAVN....
Chromatiales_bacterium_ApaH LDASESDPLRGLSVTTPGFR..QIGSAIADLNL...PTVFVQEGGYLSDVLTDSL...AA..VL
Micavibrio_aeruginosavorus_ApaH VDTFEKDPISKFKLQHDDYT..KMGDTLAKLNM...PTLFVMEGGYAVEDIGINV...AN..VL
Laribacter_hongkongensis_ApaH LDTYQGDPISTFKLDTPEFV..RMGEQLGRLGV...PTLFCLEGGYAVGPIGANA...VA..VL
Pseudomonas_putida_HDAC_family FDIYENDPQSKVHVSHEGFY..LLGQRIKALGL...PCVVIQEGGYDIATLDENA...SR..FF
Bacteroidetes_bacterium_GWF2_33_16_HDAC LDTAKGDPTGTWNFSFENFM..STGKLIGGLKI...PTLIVQEGGYKNQSLGSNA...RA..FF
Lentisphaerae_bacterium_GWF2_52_8_HDAC FDTARGDPTGTWMLDAEDFK..KIGAIIGKLPY...PTLFVQEGGYKTKTIGKN....VS..AF

```
Mycoplana_ramosa_ApaH
                                                              340

Mycoplana_ramosa_ApaH                                 .KG......V.A.G...............
Pseudomonas_aeruginosa_ApaH2                          .QG......F.E.GV....HR........
Pseudomonas_aeruginosa_ApaH1                          .QG......Y.E.GA....AR........
Pseudomonas_sp_M30-35_ApaH                            .EG......F.E.NV....CRPNNSKKDA
Candidatus_Scalindua_brodae_HDAC2                     .KG......C.N.ENRT............
Burkholderia_pseudomallei_ApaH                        .GG.......FG.ALRG............
Ralstonia_insidiosa_ApaH                              .SG.......LQ.SPHQPAG.........
Chloroflexi_bacterium_RBG_16_48_8_HdaH                .SH......FIQSNRT.............
Fervidobacterium_pennivorans_HDAC                     .AG.......LL.GE..............
Bordetella_hinzii_ApaH1                               .TN.......FE.AAHRVERG........
Bordetella_bronchiseptica_253_ApaH                    .DGFCQHHRPTA.TLR.............
Spirochaetae_bacterium_HGW-Spirochaetae-5_ApaH        .QG......LYE.GEYGRKNQ........
Kosmotoga_pacifica_HDAC                               ..F......F.S.GMLK............
Chromatiales_bacterium_ApaH                           .EG.......VL.S...............
Micavibrio_aeruginosavorus_ApaH                       .TG......F.L.NR..............
Laribacter_hongkongensis_ApaH                         .SG......Y.D.ST....AHG.......
Pseudomonas_putida_HDAC_family                        .AG.......MA.A...............
Bacteroidetes_bacterium_GWF2_33_16_HDAC               .SG.......FY.T....ANN........
Lentisphaerae_bacterium_GWF2_52_8_HDAC                FRG......VWE.G....SFR........
```

Supplementary Figure 20: Primary sequence alignment of selected bacterial deacylases of cluster 4 (UniProt accession numbers are shown).

Show are the following sequences of the following deacylases representing enzymes of cluster 4 and the sub-clusters (code: sub-cluster, accession number/protein name/spcies): 4, Q48935/ApaH/*Mycoplana ramose* (*Mycoplana bullata*); 4, Q9I6H0/ApaH2/*Pseudomonas aeruginosa* (strain ATCC 15692 / DSM 22644 / CIP 104116 / JCM 14847 / LMG 12228 / 1C / PRS101 / PAO1); 4, Q9I3T5/ApaH1/*Pseudomonas aeruginosa* (strain ATCC 15692 / DSM 22644 / CIP 104116 / JCM 14847 / LMG 12228 / 1C / PRS 101 / PAO1); 4, A0A1Y0KY79/ApaH/*Pseudomonas sp.* M30-35; 4, A0A0B0EJX1/HdaC2/*Candidatus Scalindua brodae*; 4, Q3JUN4/ApaH/*Burkholderia pseudomallei* (strain 1710b); 4, A0A191ZTI7/ApaH/*Ralstonia insidiosa*; 4, A0A1F8P1D9/HdaH/*Chloroflexi bacterium* RBG_16_48_8; 4, A0A172T1R4/HDAC/*Fervidobacterium pennivorans*; 4, A0A0H4W2H7/ApaH1/*Bordetella hinzii*; 4, A0A0C6P6V8/ApaH/*Bordetella bronchiseptica* 253; 4, A0A2N1RQT6/ApaH/*Spirochaetae bacterium* HGW-Spirochaetae-5; 4, A0A0G2Z8U2/HDAC/*Kosmotoga pacifica*; 4, A0A1E5A0H0/ApaH/*Chromatiales bacterium* (ex *Bugula neritina* AB1); 4, A0A2W5MSH4/ApaH/*Micavibrio aeruginosavorus*; 4, A0A248LJH0/ApaH/*Laribacter hongkongensis*; 4, A0A1L7NGC7/HDAC family/*Pseudomonas putida* (*Arthrobacter siderocapsulatus*); 4, A0A1F3IP43/HDAC/*Bacteroidetes bacterium* GWF2_33_16; 4, A0A1G0YY82/HDAC/*Lentisphaerae bacterium* GWF2_52_8All sequences were aligned using the T-COFFEE multiple sequence alignment server[1]. The important catalytic residues are highlighted. The secondary structure elements and numbering is shown for the deacylase shown at the top of the amino acid sequences above the sequence alignment. The alignment was created by ESPript version 3.0[2].

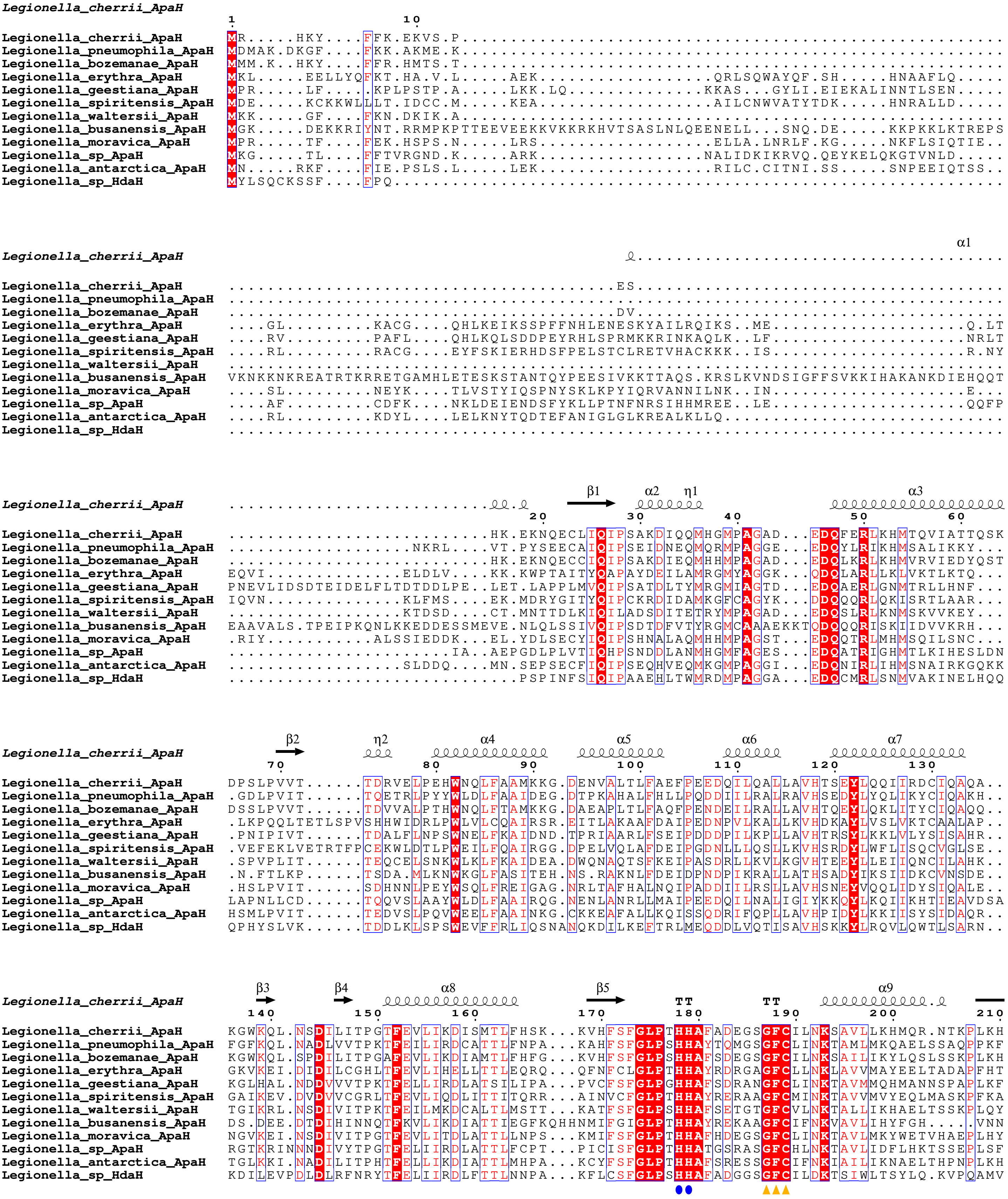
Legionella_cherrii_ApaH
Legionella_pneumophila_ApaH
Legionella_bozemanae_ApaH
Legionella_erythra_ApaH
Legionella_geestiana_ApaH
Legionella_spiritensis_ApaH
Legionella_waltersii_ApaH
Legionella_busanensis_ApaH
Legionella_moravica_ApaH
Legionella_sp_ApaH
Legionella_antarctica_ApaH
Legionella_sp_HdaH
α1
β1 α2 η1 α3
β2 η2 α4 α5 α6 α7
β3 β4 α8 β5 TT TT α9

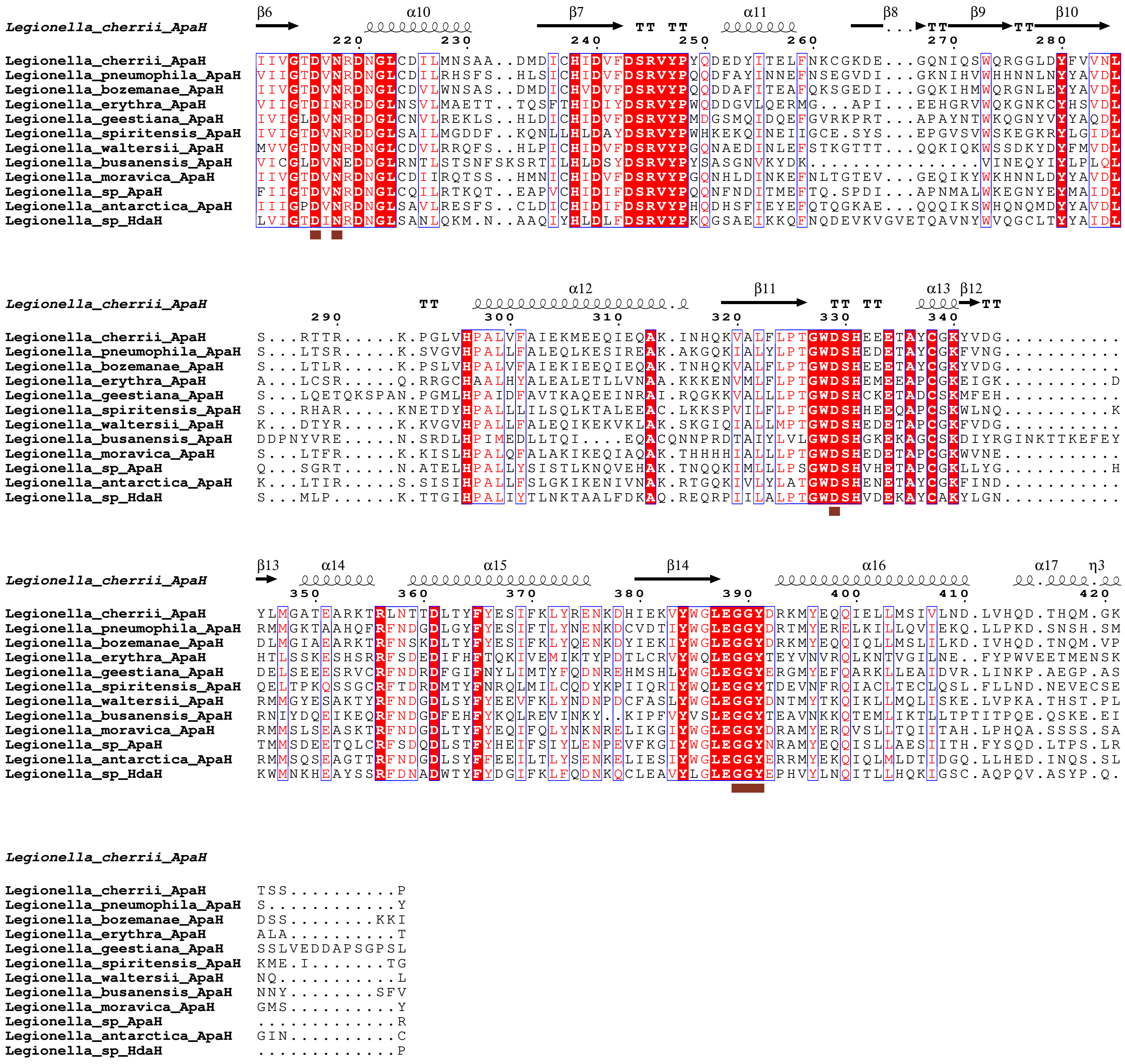
β6 α10 β7 TT TT α11 β8 TT β9 TT β10
Legionella_cherrii_ApaH
220 230 240 250 260 270 280
Legionella_cherrii_ApaH IIVGTDVNRDNGLCDILMNSAA..DMDICHIDVFDSRVYPYQDEDYITELFNKCGKDE...GQNIQSWQRGGLDYFVVNL
Legionella_pneumophila_ApaH VIIGTDVNRDNGLCDILRHSFS..HLSICHIDVFDSRVYPQQDFAYINNEFNSEGVDI...GKNIHVWHHNNLNYYAVDL
Legionella_bozemanae_ApaH IIVGTDVNRDNGLCDVLWNSAS..DMDICHVDVFDSRVYPQQDDAFITEAFQKSGEDI...GQKIHMWQRGNLEYYAVDL
Legionella_erythra_ApaH VIIGTDINRDDGLNSVLMAETT..TQSFTHIDIYDSRVYPWQDDGVLQERMG...API...EEHGRVWQKGNKCYHSVDL
Legionella_geestiana_ApaH IVIGLDVNRDDGLCNVLREKLS..HLDICHIDVFDSRVYPMDGSMQIDQEFGVRKPRT...APAYNTWKQGNYVYYAQDL
Legionella_spiritensis_ApaH IVIGTDVNRDDGLSAILMGDDF..KQNLLHLDAYDSRVYPWHKEKQINEIIGCE.SYS...EPGVSVWSKEGKRYLGIDL
Legionella_waltersii_ApaH MVVGTDVNRDNGLCDVLRRQFS..HLPICHIDVFDSRVYPGQNAEDINLEFSTKGTTT...QQKIQKWSSDKYDYFMVDL
Legionella_busanensis_ApaH VICGLDVNEDDGLRNTLSTSNFSKSRTILHLDSYDSRVYPYSASGNVKYDK...............VINEQYIYLPLQL
Legionella_moravica_ApaH IIVGTDVNRDNGLCDIIRQTSS..HMNICHIDVFDSRVYPGQNHLDINKEFNLTGTEV.......GEQIKYWKHNNLDYYAVDL
Legionella_sp_ApaH FIIGTDVNRDNGLCQILRTKQT..EAPVCHIDIFDSRVYPQQNFNDITMEFTQ.SPDI...APNMALWKEGNYEYMAIDL
Legionella_antarctica_ApaH IIIGPDVNRDNGLSAVLRESFS..CLDICHIDIFDSRVYPHQDHSFIEYEFQTQGKAE...QQQIKSWHQNQMDYYAVDL
Legionella_sp_HdaH LVIGTDINRDNGLSANLQKM.N..AAQIYHLDLFDSRVYPKQGSAEIKKQFNQDEVKVGVETQAVNYWVQGCLTYYAIDL
TT α12 β11 TT TT α13 β12 TT
Legionella_cherrii_ApaH
290 300 310 320 330 340
Legionella_cherrii_ApaH S...RTTR.....K.PGLVHPALVFAIEKMEEQIEQAK.INHQKVALFLPTGWDSHEEETAYCGKYVDG..........
Legionella_pneumophila_ApaH S...LTSR.....K.SVGVHPALLFALEQLKESIREAK.AKGQKIALYLPTGWDSHEDETAYCGKFVNG..........
Legionella_bozemanae_ApaH S...LTLR.....K.PSLVHPALVFAIEKIEEQIEQAK.TNHQKVALFLPTGWDSHEEETAYCGKYVDG..........
Legionella_erythra_ApaH A...LCSR.....Q.RRGCHAALHYALEALETLLVNAA.KKKENVMLFLPTGWDSHEMEEAPCGKEIGK.........D
Legionella_geestiana_ApaH S...LQETQKSPAN.PGMLHPAIDFAVTKAQEEINRAI.RQGKKVALLLPTGWDSHCKETADCSKMFEH..........
Legionella_spiritensis_ApaH S...RHAR.....KNETDYHPALLLILSQLKTALEEAC.LKKSPVILFLPTGWDSHHEEQAPCSKWLNQ.........K
Legionella_waltersii_ApaH K...DTYR.....K.KVGVHPALLFALEQIKEKVKLAK.SKGIQIALLMPTGWDSHEDETAPCGKFVDG..........
Legionella_busanensis_ApaH DDPNYVRE.....N.SRDLHPIMEDLLTQI....EQACQNNPRDTAIYLVLGWDSHGKEKAGCSKDIYRGINKTTKEFEY
Legionella_moravica_ApaH S...LTFR.....K.KISLHPALQFALAKIKEQIAQAK.THHHHIALLLPTGWDSHEDETAPCGKWVNE..........
Legionella_sp_ApaH Q...SGRT.....N.ATELHPALLYSISTLKNQVEHAK.TNQQKIMLLLPSGWDSHVHETAPCGKLLYG.........H
Legionella_antarctica_ApaH K...LTIR.....S.SISIHPALLFSLGKIKENIVNAK.RTGQKIVLYLATGWDSHENETAYCGKFIND..........
Legionella_sp_HdaH S...MLP......K.TTGIHPALIYTLNKTAALFDKAQ.REQRPITLALPTGWDSHVDEKAYCAKYLGN..........
β13 α14 α15 β14 α16 α17 η3
Legionella_cherrii_ApaH
350 360 370 380 390 400 410 420
Legionella_cherrii_ApaH YLMGATEARKTRLNTTDLTYFYESIFKLYRENKDHIEKVYWGLEGGYDRKMYEQQIELLMSIVLND.LVHQD.THQM.GK
Legionella_pneumophila_ApaH RMMGKTAAHQFRFNDGDLGYFYESIFTLYNENKDCVDTIYWGLEGGYDRTMYERELKILLQVIEKQ.LLPKD.SNSH.SM
Legionella_bozemanae_ApaH DLMGIAEARKTRFNSKDLTYFYESIFKLYQENKDYIEKIYWGLEGGYDRKMYEQQIQLLMSLILKD.IVHQD.TNQM.VP
Legionella_erythra_ApaH HTLSSKESHSRRFSDEDIFHFTQKIVEMIKTYPDTLCRVYWQLEGGYTEYVNVRQLKNTVGILNE..FYPWVEETMENSK
Legionella_geestiana_ApaH DELSEEESRVCRFNDRDFGIFNYLIMTYFQDNREHMSHLYWGLEGGYERGMYEFQARKLLEAIDVR.LINKP.AEGP.AS
Legionella_spiritensis_ApaH QELTPKQSSGCRFTDRDMTYFNRQLMILCQDYKPIIQRIYWQLEGGYTDEVNFRQIACLTECLQSL.FLLND.NEVECSE
Legionella_waltersii_ApaH RMMGYESAKTYRFNDGDLSYFYEEVFKLYNDNPDCFASLYWGLEGGYDNTMYTKQIKLLMQLISKE.LVPKA.THST.PL
Legionella_busanensis_ApaH RNIYDQEIKEQRFNDGDFEHFYKQLREVINKY..KIPFVYVSLEGGYTEAVNKKQTEMLIKTLLTPTITPQE.QSKE.EI
Legionella_moravica_ApaH RMMSLSEASKTRFNDGDLTYFYEQIFQLYNKNRELIKGIYWGLEGGYDRAMYERQVSLLTQIITAH.LPHQA.SSSS.SA
Legionella_sp_ApaH TMMSDEETQLCRFSDQDLSTFYHEIFSIYLENPEVFKGIYWGLEGGYNRAMYEQQISLLAESIITH.FYSQD.LTPS.LR
Legionella_antarctica_ApaH RMMSQSEAGTTRFNDGDLSYFFEEILTLYSENKELIESIYWGLEGGYERRMYEKQIQLMLDTIDGQ.LLHED.INQS.SL
Legionella_sp_HdaH KWMNKHEAYSSRFDNADWTYFYDGIFKLFQDNKQCLEAVYLGLEGGYEPHVYLNQITLLHQKIGSC.AQPQV.ASYP.Q.
Legionella_cherrii_ApaH
Legionella_cherrii_ApaH TSS..........P
Legionella_pneumophila_ApaH S............Y
Legionella_bozemanae_ApaH DSS........KKI
Legionella_erythra_ApaH ALA..........T
Legionella_geestiana_ApaH SSLVEDDAPSGPSL
Legionella_spiritensis_ApaH KME.I.......TG
Legionella_waltersii_ApaH NQ...........L
Legionella_busanensis_ApaH NNY........SFV
Legionella_moravica_ApaH GMS..........Y
Legionella_sp_ApaH ....R
Legionella_antarctica_ApaH GIN..........C
Legionella_sp_HdaH ....P

Supplementary Figure 21: Primary sequence alignment of selected bacterial deacylases of cluster 3 (UniProt accession numbers are shown).
Show are the following sequences of the following deacylases representing enzymes of cluster 3 and the sub-clusters (code: sub-cluster, accession number/protein name/spcies): 3, A0A0W0SGS1/ApaH/*Legionella cherrii*; 3, A0A2S6EWV0/ApaH/*Legionella pneumop*hila; 3, A0A0W0RV07/ApaH/*Legionella bozemana*e (*Fluoribacter bozemanae*); 3, A0A0W0TS12/ApaH/*Legionella erythra*; 3, A0A0W0TY70/ApaH/*Legionella geestiana*; 3, A0A0W0YXX3/ApaH/*Legionella spiritensis*; 3, A0A0W1A0K6/ApaH/*Legionella waltersii*; 3, A0A378JU93/ApaH/*Legionella busanensis*; 3, A0A378K274/ApaH/*Legionella moravica*; 3, A0A522FB57/ApaH/*Legionella sp.*; 3, A0A6F8T350/ApaH/*Legionella antarctica*; 3, A0A8I1Q8A3/HdaH/*Legionella sp.* All sequences were aligned using the T-COFFEE multiple sequence alignment server[1]. The important catalytic residues are highlighted. The secondary structure elements and numbering is shown for the deacylase shown at the top of the amino acid sequences above the sequence alignment. The alignment was created by ESPript version 3.0[2].

a

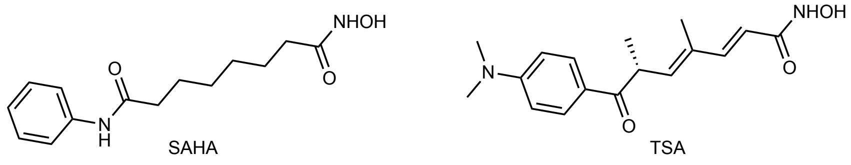

b

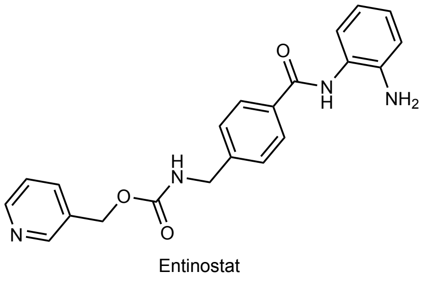

c

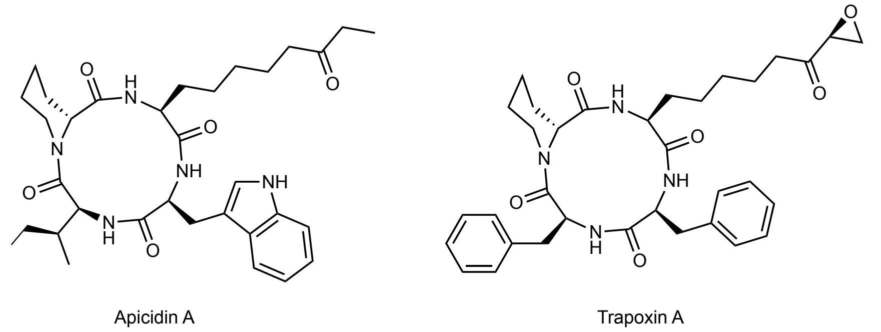

Supplementary Figure 22: Overview of structures of deacylase hydroxamate inhibitors SAHA, trichostatin A (TSA), benzamide etinostat (MS-275) and the cyclic peptides apicidin A and trapoxin A.

a. The hydroxamate inhibitors trichostatin A (TSA) and SAHA (vorinostat, suberoylanilide hydroxamic acid (SAHA)) are potent class I and class II classical HDAC pan-inhibitors. The hydroxamate moiety is used to complex the catalytic Zn^{2+}-ion. Bacterial deacylases of all clusters, except from cluster B, are potently inhibited by these hydroxamate inhibitors.

b. The benzamide inhibitor entinostat (MS-275) shows selectivity towards class I HDACs. Our data show no bacterual deacylase being potently inhibited by this benzamide inhibitor.

c. The cyclic peptide inhibitors trapoxin A and apicidin A were shown to selectively inhibit class I HDACs. This is in agreement with our data revealing only cluster B enzyme *Bs*AcuC being selectively and potently inhibited by both cyclic peptide inhibitors.

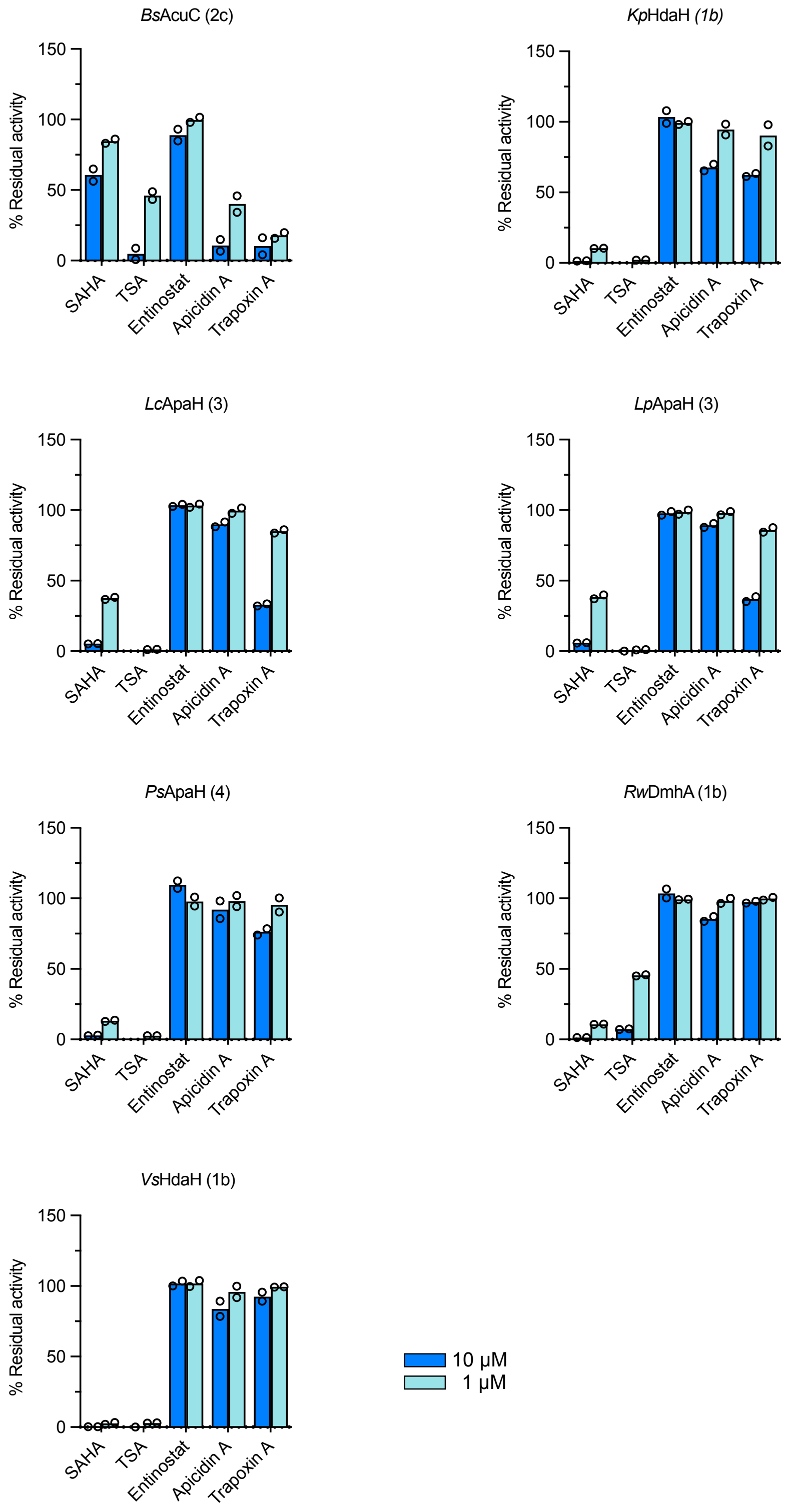

Supplementary Figure 23: Screening of hydroxamate, benzamide and cyclic peptide inhibitors to inhibit selected bacterial deacylases.
The hydroxamate inhibitors trichostatin A (TSA) and SAHA (vorinostat), the benzamide inhibitor entinostat (MS-275) and the cyclic peptide inhibitors trapoxin A and apicidin A were used at concentrations of 1 µM and 10 µM and varying enzyme concentrations (cluster A: 15 nM *Kp*HdaH, 30 nM *Rw*DmhA 1.5 nM *Vs*HdaH; cluster B: 200 nM *Bs*AcuC; cluster D: 40 nM *Ps*ApaH; cluster E: 30.5 nM *Lc*ApaH, 0.5 nM *Lp*ApaH) to screen selected bacterial deacylases. The assay was conducted for 1h at 37°C before stopping the reaction. The measured residual activity was determined in % based on the AMC fluorescence obtained defining the 100% value by measuring the fluorescence of the free AMC-fluorophore at the substrate concentrations (40 µM LGKac *Bs*AcuC (2c), *Lc*ApaH (3), *Lp*ApaH (3); 60 µM QPKKac: *Kp*HdaH (1b), *Rw*DmhA (1b), *Vs*HdaH (1b), *Ps*ApaH (1b)). The data obtained for 1 µM inhibitor was used to create the heat map shown in Fig. 8a. The experiment was performed in two independent replicates (*n*=2). Bars depict means. Source data are provided as Source Data file.

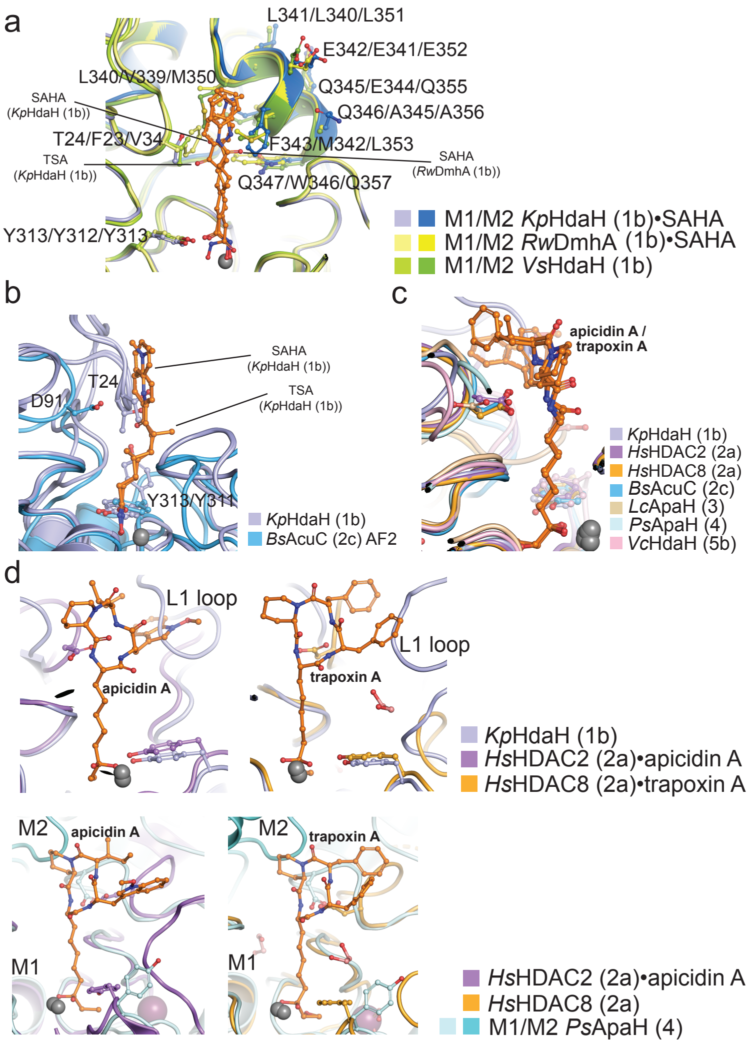

Supplementary Figure 24: Structural determinants for selectivity of hydroxamate inhibitors SAHA and TSA and of trapoxin A and apicidin A in inhibition of bacterial deacylases.

- **a** *Rw*DmhA (1b) is the only enzyme showing better inhibition by SAHA compared to TSA and *Vs*HdaH (1b) is equally well inhibited by SAHA and TSA. To reveal the underlying mechanism, the structures of *Rw*DmhA (1b) and *Vs*HdaH (1b) (AF2) were superimposed with *Kp*HdaH (1b)•TSA and *Kp*HdaH (1b)•SAHA. Shown is a closeup to provide details on binding of the inhibitors.
- **b** To reveal molecular determinants underlying the low potency of inhibition of *Bs*AcuC (2c) activity observed for SAHA and TSA, the AlphaFold2 model of *Bs*AcuC (2c) was superimposed with the structures of *Kp*HdaH (1b)•TSA and *Kp*HdaH (1b)•SAHA.
- **c** D101/D100 in *Hs*HDAC8/*Hs*HDAC2 is needed for inhibition by trapoxin A and apicidin A. Superposition of the structures of the core domains of bacterial deacylases *Kp*HdaH (1b), *Rw*DmhA (1b), *Rs*PrpH (1b) *Bs*AcuC (2c), *Lp*ApaH (3), *Lc*ApaH (3), *Ps*ApaH (4) VcHdaH (5b) and *Hs*HDAC2 (2a) and *Hs*HDAC8 (2a) and modelling of trapoxin A and apicidin A reveals, except from *Ps*ApaH (4) and *Vc*HdaH (5b), either a negatively charged D or E is present at analogue localization as in *Hs*HDAC2 (2a) and *Hs*HDAC8 (2a). The observed lack of inhibition of *Kp*HdaH (1b), *Rw*DmhA (1b), *Rs*PrpH (1b), *Lp*ApaH (3), *Lc*ApaH (3), *Ps*ApaH (4) *Vc*HdaH (5b) is due to other determinants in structure or sequence.
- **d** Except from *Bs*AcuC (2c) none of the bacterial enzymes is inhibited by apicidin A or trapoxin A. Superposition of structures of bacterial deacylases with apicidin A and trapoxin A were done to reveal molecular mechanisms explaining the lack of inhibition of bacterial deacylases *Kp*HdaH (1b), and *Ps*ApaH (4) by apicidin A and trapoxin A. *Ps*ApaH (4) and *Vc*HdaH (5b) do not contain an D/E at the analogous positiion to D101/D100 in *Hs*HDAC8/*Hs*HDAC2 explaining their lack in inhibition by cyclic peptides (*Kp*HDAH (1b): E101; *Bs*AcuC (2c): D91 ; *Lp*ApaH (3): D146; *Lc*ApaH (3): D144; *Ps*ApaH (4): G117; *Vc*HdaH (5b): G97; *Hs*HDAC2: D100; *Hs*HDAC8: D101). For the complexes the crystal structures of the indicated enyzmes were superimposed with the structures of HDAC2•apicidinA (PDB: 7LTG [https://doi.org/10.2210/pdb7LTG/pdb]) and HDAC8•trapoxin A (PDB: 5VI6 [https://doi.org/10.2210/pdb5VI6/pdb]) to model the localization of apicidin A and trapoxin A. For *Bs*AcuC (2c) the AlphaFold2 model was used.

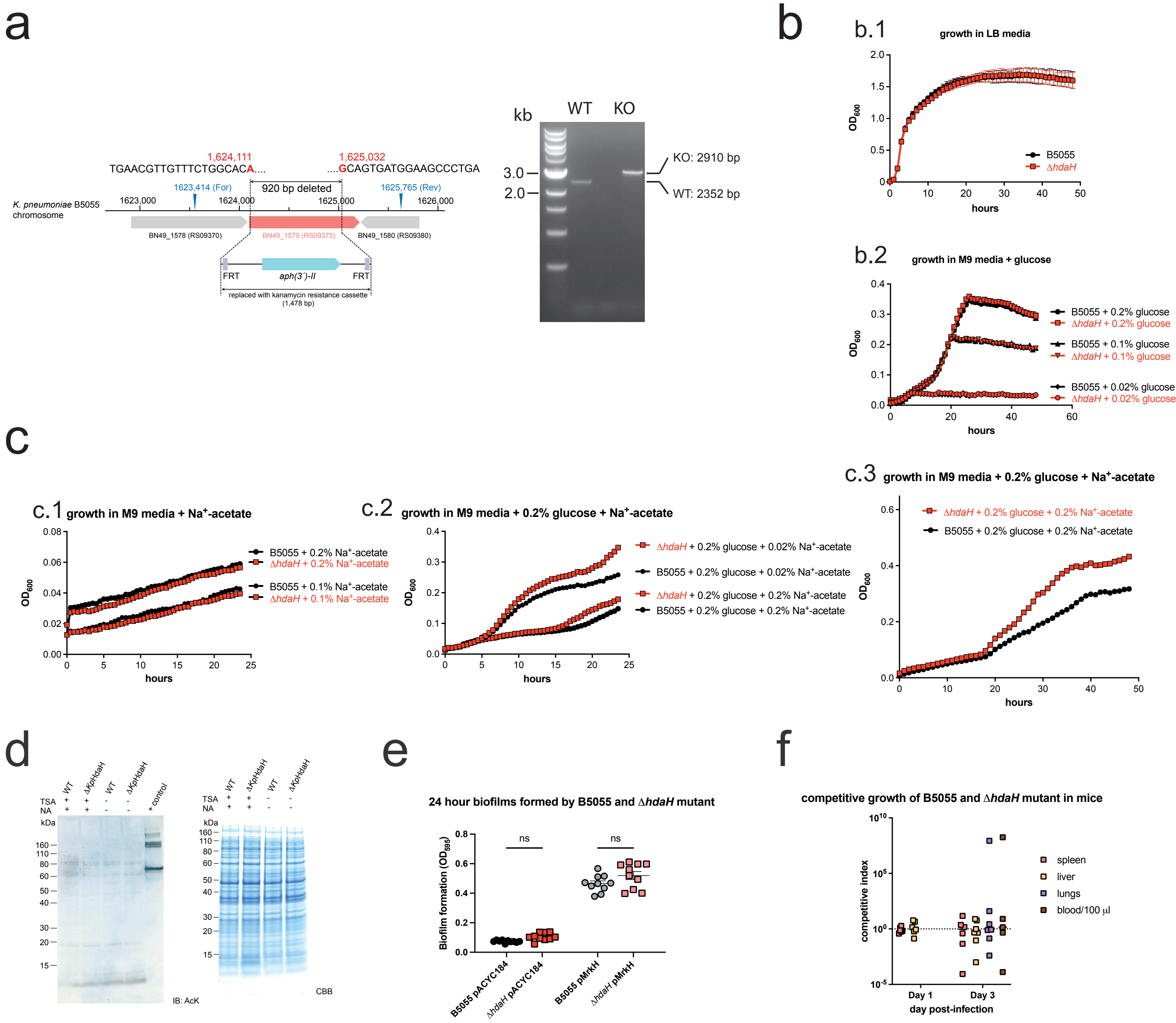
a
1,624,111
TGAACGTTGTTTCTGGCACA....
1,625,032
....GCAGTGATGGAAGCCCTGA
920 bp deleted
1623,414 (For)
1625,765 (Rev)
K. pneumoniae B5055 chromosome
1623,000
1624,000
1625,000
1626,000
BN49_1578 (RS09370)
BN49_1579 (RS09375)
BN49_1580 (RS09380)
FRT
aph(3´)-II
FRT
replaced with kanamycin resistance cassette (1,478 bp)
kb
WT
KO
3.0
2.0
KO: 2910 bp
WT: 2352 bp
b
b.1
growth in LB media
B5055
ΔhdaH
hours
b.2
growth in M9 media + glucose
B5055 + 0.2% glucose
ΔhdaH + 0.2% glucose
B5055 + 0.1% glucose
ΔhdaH + 0.1% glucose
B5055 + 0.02% glucose
ΔhdaH + 0.02% glucose
hours
c
c.1 growth in M9 media + Na+-acetate
B5055 + 0.2% Na+-acetate
ΔhdaH + 0.2% Na+-acetate
B5055 + 0.1% Na+-acetate
ΔhdaH + 0.1% Na+-acetate
hours
c.2 growth in M9 media + 0.2% glucose + Na+-acetate
ΔhdaH + 0.2% glucose + 0.02% Na+-acetate
B5055 + 0.2% glucose + 0.02% Na+-acetate
ΔhdaH + 0.2% glucose + 0.2% Na+-acetate
B5055 + 0.2% glucose + 0.2% Na+-acetate
hours
c.3 growth in M9 media + 0.2% glucose + Na+-acetate
ΔhdaH + 0.2% glucose + 0.2% Na+-acetate
B5055 + 0.2% glucose + 0.2% Na+-acetate
hours
d
WT
ΔKpHdaH
WT
ΔKpHdaH
+ control
TSA
NA
kDa
IB: AcK
CBB
e
24 hour biofilms formed by B5055 and ΔhdaH mutant
Biofilm formation (OD595)
ns
ns
B5055 pACYC184
ΔhdaH pACYC184
B5055 pMrkH
ΔhdaH pMrkH
f
competitive growth of B5055 and ΔhdaH mutant in mice
competitive index
spleen
liver
lungs
blood/100 μl
Day 1
Day 3
day post-infection

Supplementary Figure 25: Impact of genomic deletion of *hdaH* in *K. pneumoniae* on growth, biofilm formation and virulence.

a Genomic deletion of the gene *hdaH* encoding for *Kp*HdaH (1b) in the genome of *K. pneumoniae* B5055 (B5055). To determine whether the gene encoded a product that was functional in the metabolism and pathogenesis of site-specific mutagenesis deleting the *hdaH* gene in B5055 was conducted. The *Kp*HdaH enzyme is annotated as a putative histone deacetylase encoded by BN49_1579 (BN49_RS09375) on the chromosome of *Klebsiella pneumoniae*. The BN49_1579 gene was disrupted by replacing a 920 bp coding region with a 1,478 bp kanamycin resistance cassette (flanked by FRT sites) using λ-red recombinase-mediated gene gorging method[5]. The location of BN49_1579 and key sites for mutagenesis are shown, with genomic coordinates obtained from www.biocyc.org. Block arrows depict annotated transcription units in the direction of transcription. Dotted lines indicate the excision points immediately adjacent to the deleted sequence. The up- and downstream sequences flanking the deletion site are shown above, with the last bp retained and their coordinates shown in red. Blue triangles show primer binding sites (For, forward primer; Rev, reverse primer) for PCR that confirms gene deletion. The agarose gel electrophoresis image shows PCR products of expected size for wildtype (WT) and knockout (KO) *K. pneumoniae*. The B5055 *hdaH* mutant (Δ*hdaH*) and wildtype (WT) B5055 were analysed by whole-genome sequencing using the Illumina platform. No secondary mutation outside of BN49_1579 was detected, thus validating site-specific mutation in the mutant strain. Source data are provided as Source Data file.

b Growth of B5055 in LB-medium and M9-medium supplemented with glucose is not affected by the absence of HdaH. Top panel, b.1: The B5055 WT and B5055 Δ*hdaH* were grown in Luria-Bertani broth, a rich medium containing multiple carbon sources. The growth curves of the WT and Δ*hdaH* were determined using a CLARIOstar Plus plate reader over 48 hours. There was no difference in the fundamental growth kinetics. Both isolates demonstrated similarities in lag, log and stationary phases. Lower panel, b.2: To determine whether the multiple carbon sources and high glucose concentrations of LB obscured differences in growth kinetics, the B5055 WT and B5055 Δ*hdaH* were grown in minimal medium (M9) with varying concentrations of glucose. Terminal OD_{600} was determined by glucose concentration and were approximately 25% of OD_{600} generated by growth in LB broth. Again, there were no differences in the growth kinetics of the B5055 WT and B5055 Δ*hdaH*. The analysis of growth in rich and simple media comparing B5055 WT and B5055 Δ*hdaH* in central glucose metabolism of B5055 suggested that *Kp*HdaH (1b) had no role in determining the kinetics of growth when glucose was provided as the sole source of carbon. The experiment was performed with three biological replicates (n=3) each containing 4 technical replicates. Data are presented as means±SD of the three biological replicates. Source data are provided as Source Data file.

c We next explored whether B5055 Δ*hdaH* increased the rate or level of growth when sodium acetate was provided as the sole carbon source. Left pane, c.1: Growth of B5055 in sodium acetate was very limited albeit the level of growth was determined by the concentration by the amount of sodium acetate added. Again there was no difference in growth rate when the WT was compared with the Δ*hdaH* mutant. Middle panel, c.2: When glucose was added to acetate in M9 medium, differences in growth rate were observed between the B5055 WT and the B5055 Δ*hdaH* mutant. The level of growth for the B5055 Δ*hdaH* was increased over that of the B5055 WT for the first 24 hours, suggesting that the presence of an intact *hdaH* locus, together with sodium acetate reduced growth of *K. pneumoniae* B5055. Right panel,c.3: When the analysis was extended by a further 24 hours, a second lag and renewed exponential growth was seen in an additional phase of growth (diauxic growth). We hypothesized that once glucose was consumed B5055 swiched to using acetate as the carbon source. The experiment was performed with two biological replicates (n=2), each containing 4 technical replicates. Data are represented as means. Source data are provided as Source Data file.

d Genomic deletion *of K. pneumoniae hdaH* does not result in a generalised alteration of the acetylation pattern in cells grown in M9 medium. To determine whether any deacylation activity of *Kp*HdaH could be identified in cultured *K. pneumoniae* B5055, the B5055 WT and B50555 Δ*hdaH* mutant from stationary phase cultures of bacteria grown in M9 medium were analysed by Western immunoblotting using an acetyl-group specific antibody (IB: AcK). The positive control was acetylated bovine serum albumin (BSA), which was clearly positive in the Western blot, with a MW consistent with that of BSA (67 kDa). Different additives including the classical deacetylase inhibitor trichostatin A (TSA; 200 nM) and nicotinamide (NA; 2 mM) were supplied but there was no difference in the Western blot profile of B5055 WT and B5055 Δ*hdaH*. Source data are provided as Source Data file.

e *K. pneumoniae* HdaH does not affect biofilm formation. Two further analyses were undertaken of the B5055 WT and B5055 Δ*hdaH*. Firstly, the impact of the putative de-acetylase on biofilm formation was determined. *K. pneumoniae* B5055 is a poor biofilm former because it lacks an intact

copy of MrkH, the transcriptional activator of Mrk fimbriae expression. The Mrk fimbria is a key mediator of *K. pneumoniae* biofilm formation[6]. To address this issue, B5055 and the Δ*hdaH* mutant of B5055 were transfected with a functional plasmid copy of MrkH (pMrkH). The supply of *mrkH* in trans resulted in an increase in biofilm formation, however there was no significant difference in the level of biofilm produced by the WT complemented with pMrkH) and the Δ*hdaH* mutant. Ten independent biological experiments were conducted and the data shown are the technical replicates from one representative experiment. Ten individual data points for each isolate (B5055 WT and B5055 Δ*hdaH*) were collected from each of two independent experiments. A representative experiment is presented (technical replicates, *n*=10). There was no statistical difference (ns) in the level of biofilm produced by B5055 and B5055 Δ*hdaH* using an ordinary one-way ANOVA test (Tukey's multiple comparison test) with a significance level of $p<0.05$. Exact values can be found in the Source Data. Source data are provided as Source Data file.

f *K. pneumoniae* HdaH has no direct effect on *K. pneumoniae* virulence. Lastly, the virulence of the WT and the Δ*hdaH* mutant was tested in C57BL/6 (BL/6) mice. B5055 is a known virulent isolate in BL/6 mice[7,8]. Animals were infected with 3 different doses of WT and Δ*hdaH* mutant. *In toto*, these studies failed to reveal a dose-dependent phenotype associated with loss of the *hdaH* locus. While in some experiments, it appeared that there may have been some growth attenuation of Δ*hdaH*, the effects were not consistent. At low doses (10^3 colony forming units (CFU) injected intravenously (i.v.)), there may have been some growth deficit in Δ*hdaH* but this was not seen when doses were increased 10-fold, where survival and weight loss post-infection were not increased by loss of the locus. Competitive infections were also assessed, where C57BL/6 mice were infected with equal numbers of the WT and *Kp*HdaH Δ*hdaH* mutant and then killed at day 1 or day 3 post-infection, and the number of *K. pneumoniae* in various tissues analysed by viable counts (right panel). However, again no significant effect was observed and the data shows quite huge variation. Data points represent individual mice (*n*=6) in one experiment. The result is representative of two independent experiments. Source data are provided as Source Data file.

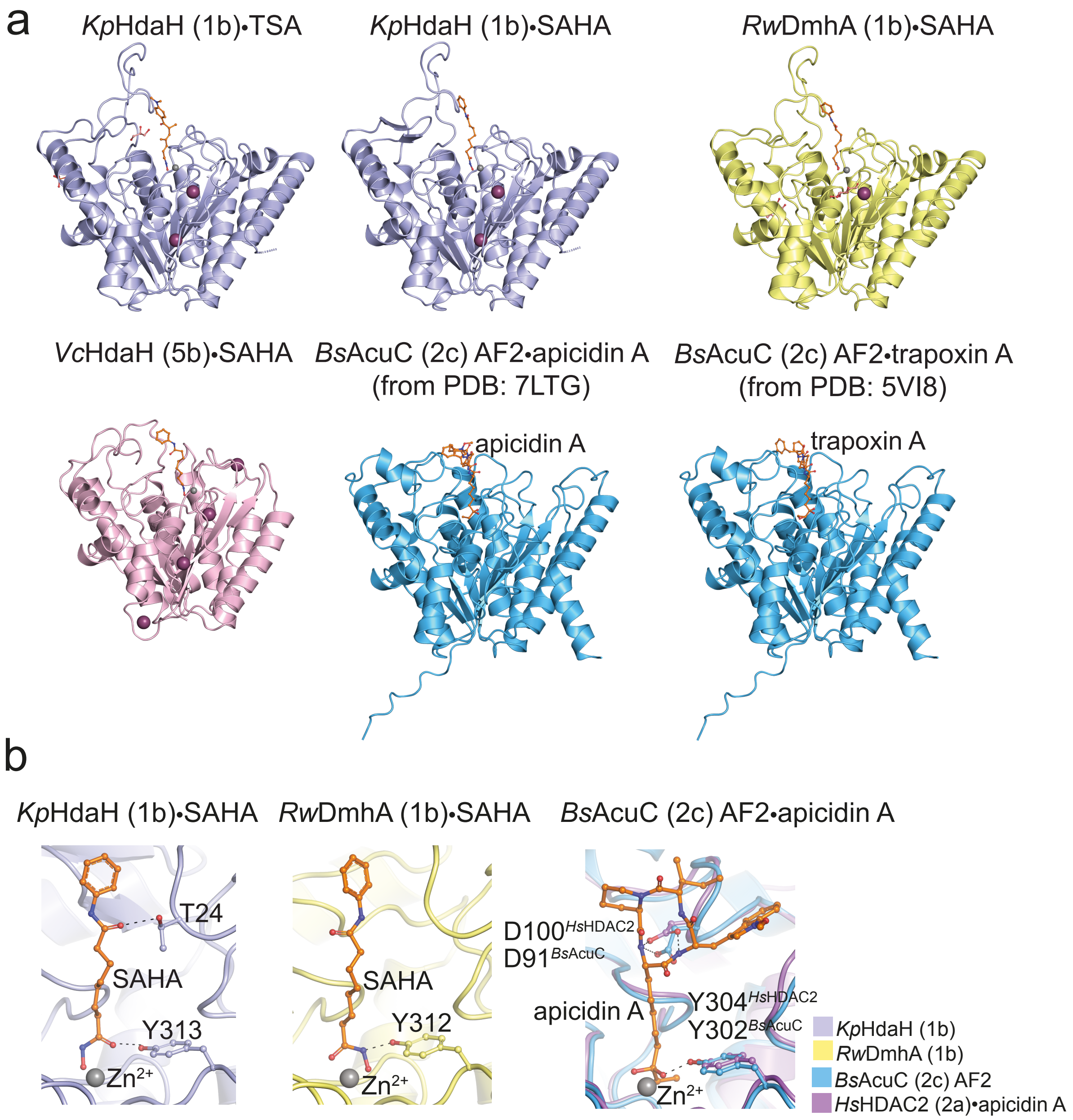

Supplementary Figure 26: Overview of structures of bacterial deacylases in complexes with the hydroxamate inhibitors SAHA, and trichostatin A (TSA).

a. Overview of all inhibitor bound-structures of bacterial deacylases of all sub-clusters. Shown are the following complexes of the deacylases in complexes with the hydroxamates SAHA and/or TSA: *Kp*HdaH•TSA, *Kp*HdaH•SAHA and *Rw*DmhA•SAHA of sub-cluster 1b and *Vc*HdaH•SAHA of cluster 5b. For the complexes of *Bs*AcuC of sub-cluster 2c with apixidin A and trapoxin A, the AlphaFold2 model of *Bs*AcuC was superimposed with the structures of HDAC2•apicidinA (PDB: 7LTG [https://doi.org/10.2210/pdb7LTG/pdb]) and HDAC8•trapoxin A (PDB: 5VI6 [https://doi.org/10.2210/pdb5VI6/pdb]).

b. Closeup of the structures of *Kp*HdaH•TSA, *Rw*DmhA•SAHA and of the AlphaFold2 model of *Bs*AcuC in complex with apicidin A (from PDB: 7LTG [https://doi.org/10.2210/pdb7LTG/pdb]). The conserved Tyr (KpHdaH: Tyr313) directly contacts the oxygen /nitrogen of the hydroxamate groups of SAHA or TSA, respectively, or the gemdiol(ate) function of the apicidin A.. This is at the analogous position of the substrates' carbonyl oxygen of the acyl group. The hydroxamate and gemdiol(ate) function furthermore directyl coordinates the catalytic Zn^{2+}-ion in a bidentate fashion. As dexcribed for the interaction with SAHA, Thr24 in *Kp*HdaH form an interaction to the TSA carbonyl oxygen. The interaction of the cyclic peptide inhibitor apicidin A is created by formation of a hydrogen bound with the conserved Asp91 in *Bs*AcuC (2c) towards the peptide bonds of the cyclic peptide inhibitor. This Asp contributes to the selectively to inhibit class I HDACs.

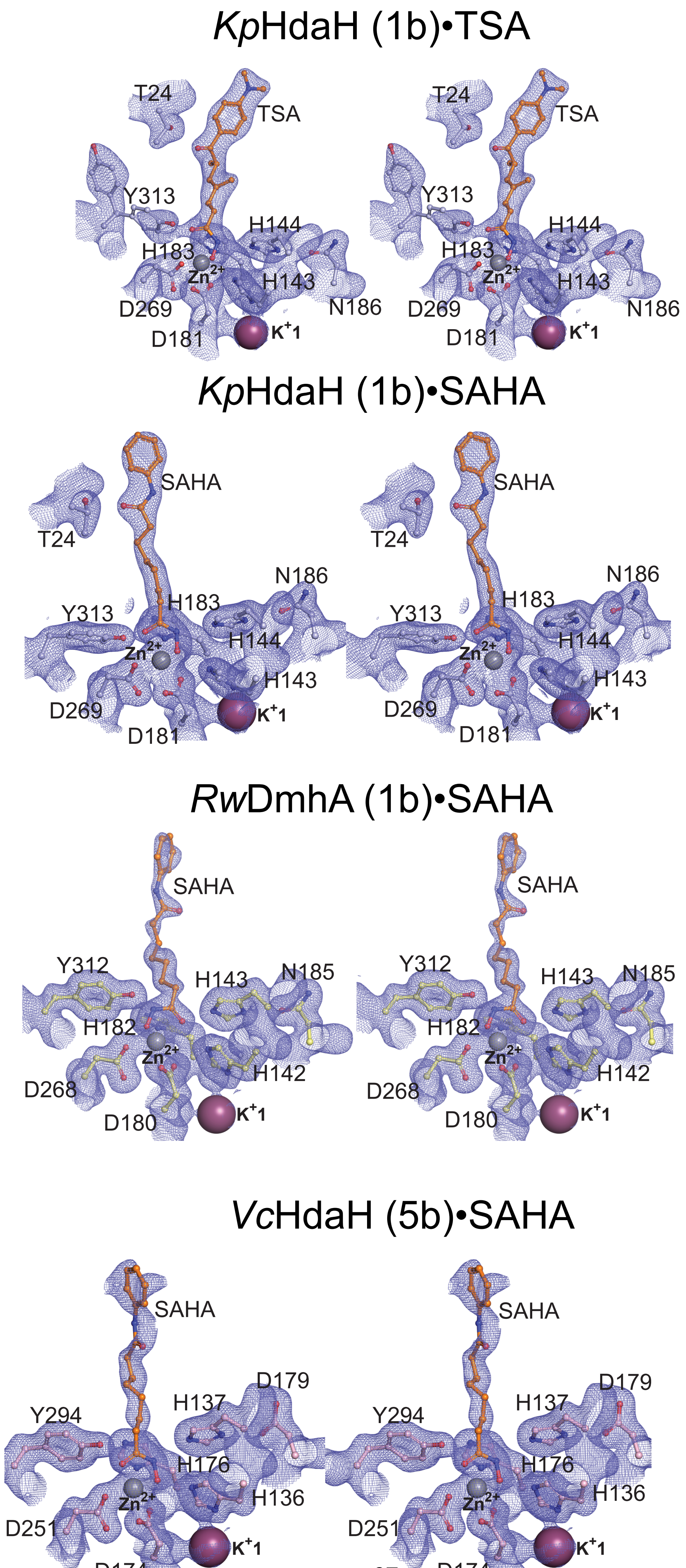
KpHdaH (1b)•TSA
T24
TSA
Y313
H144
H183
Zn2+
H143
D269
N186
D181
K+1
KpHdaH (1b)•SAHA
SAHA
T24
N186
H183
Y313
H144
Zn2+
H143
D269
K+1
D181
RwDmhA (1b)•SAHA
SAHA
Y312
H143
N185
H182
Zn2+
H142
D268
D180
K+1
VcHdaH (5b)•SAHA
SAHA
D179
H137
Y294
H176
H136
Zn2+
D251
K+1
D174

Supplementary Figure 27: Stereo images of representative electron density for inhibitor-bound structures of bacterial deacylases of sub-cluster 1b solved in this study.

Stereo figures for the inhibitor bound-structures of bacterial deacylases of all sub-clusters. Shown are closeups of the following complexes: *Kp*HdaH (1b)•SAHA, *Kp*HdaH (1b)•TSA and *Rw*DmhA (1b)•SAHA and *Vc*HdaH (5b)•SAHA of subcluster 1b. For all structures a representative closeup of the active site is shown, including the bidentate Zn^{2+}-binding of the hydroxamate inhibitor, the coordinating His and the two Asp residues, the catalytic base His and the active site Tyr important to orient and polarize the carbonyl-group of the substrate and to stabilize the tetrahedral intermediate. Shown in blue is the electron density of the $2F_o$-F_c map contoured at 1σ. The stereo diagrams were prepared in wall-eyed viewing mode with PyMOL version 2.3.4[3].

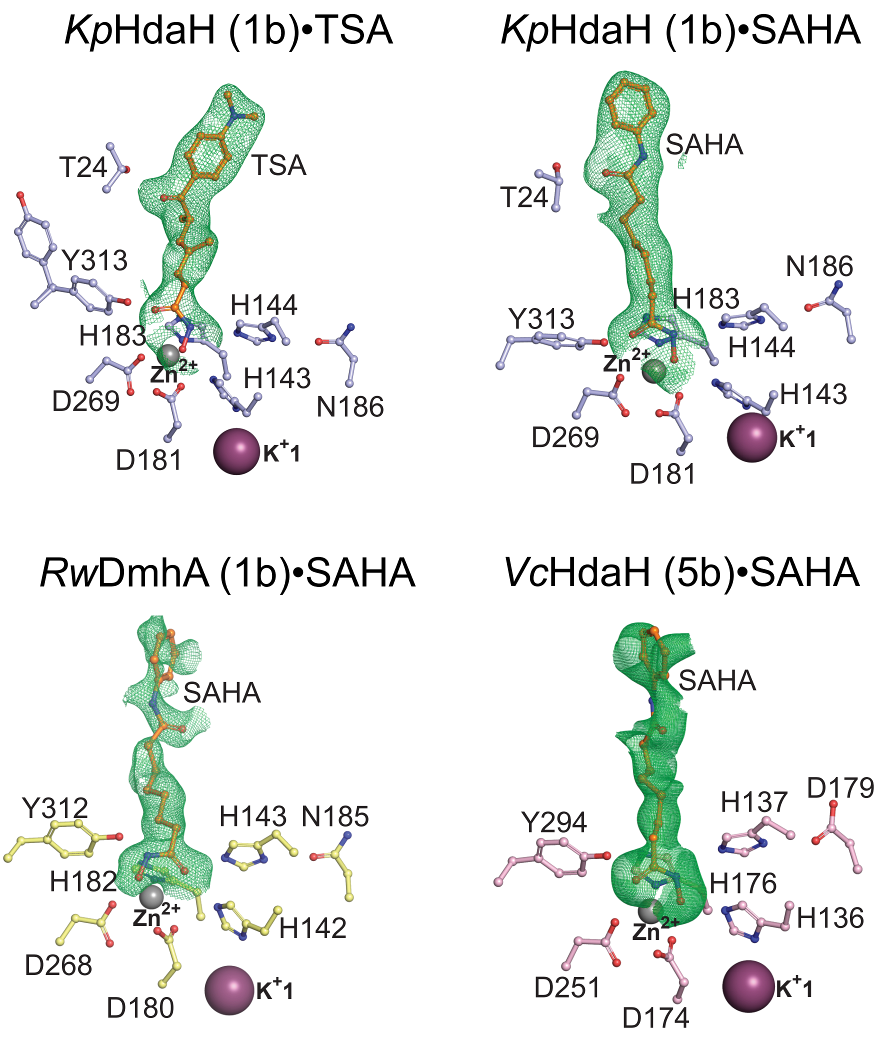

Supplementary Figure 28: F_o-F_c omit maps for the electron density of the hydroxamates TSA and SAHA for all inhibitor-bound structures of bacterial deacylases solved in this study.
Shown are the F_o-F_c omit maps (contour level 3σ) for the inhibitors TSA and SAHA for the structures of the deacylases in complexes with the hydroxamate inhibitors: *Kp*HdaH (1b)•SAHA, *Kp*HdaH (1b)•TSA, *Rs*PrpH (1b)•SAHA, *Rw*DmhA (1b)•SAHA and *Vc*HdaH (5b)•SAHA. For all structures a representative closeup of the active site is shown, including the bidentate Zn^{2+}-binding of the hydroxamate inhibitor, the coordinating His and the two Asp residues, the catalytic base His and the active site Tyr important to orient and polarize the carbonyl-group of the substrate and to stabilize the tetrahedral intermediate. Shown in green is the electron density of the F_o-F_c map contoured at 3σ. The figures were prepared with PyMOL version 2.3.4[3].
and the XGGY-motif lining the foot pocket and the catalytic Tyr (*Kp*HdaH: Y313); green box: RPP-motif lining the foot pocket.

Supplementary Table 1: *Escherichia coli* strains used in this study.

Strain	Genotype	Reference/construction
BL21 (DE3)	*B F⁻ ompT gal dcm lon hsdS$_B$(r$_B$⁻m$_B$⁻) λ(DE3 [lacI lacUV5-T7p07 ind1 sam7 nin5]) [malB⁺]$_{K-12}$(λ^S)*	[9]
DH5α	*F⁻ endA1 glnV44 thi-1 recA1 relA1 gyrA96 deoR nupG purB20 φ80dlacZΔM15 Δ(lacZYA-argF)U169, hsdR17(r$_K$⁻m$_K$⁺), λ⁻*	[10]

Supplementary Table 2: Oligonucleotides used in this study. All oligonucleotides ordered from Sigma/Merck (https://www.sigmaaldrich.com/DE/de/configurators/tube?product=standard).

**Primer**	**Sequence 5'->3'**
QC_VsHdaH_H144A_fo	gtcgtccgccgggtcatgcttgcctggcc
QC_VsHdaH_H144A_rev	ggccaggcaagcatgacccggcggacgac
QC_RsPrpH_H143A_fo	gccggccatgcctgtctgccggatac
QC_RsPrpH_H143A_rev	gtatccggcagacaggcatggccggc
QC_RwDmhA_H143A_fo	ggcaggtcatgcatgtctggcagatac
QC_RwDmhA_H143A_rev	gtatctgccagacatgcatgacctgcc
QC_VcHdaH_H126A_fo	gtggttatcatgccgcccatgcagac
QC_VcHdaH_H126A_rev	gtctgcatgggcggcatgataaccac
QC_ApaHD3_H159A_fo	ccgggccatgcagcagcaagtgatgttatg
QC_ApaHD3_H159A_rev	cataacatcacttgctgctgcatggcccgg
QC_LcApaH_E_H179A_fo	cgacccatgcagccttcgcagatgaag
QC_LcApaH_E_H179A_rev	cttcatctgcgaaggctgcatgggtcg
QC_LpApaH_H181A_fo	gccgagccatgccgcctataccc
QC_LpApaH_H181A_rev	gtataggcggcatggctcggc
Gi_pOPIN_fo	gttatgaaggtgcagcccgttagaccatttaaacaccac
Gi_pOPIN_rev	ctatacacactcagcataccgtaccaccgatctgttc
Gi_PA1409_fo	cagatcggtggtaccggtatgctgagtgtgtatagcg
Gi_PA1409_rev	ggtggtgtttaaatggtctaacgggctgcaccttc
QC_PsApaH_1-342_Stop_fo	gaaaatgtgtaacgtccgaataatagtaaaaaag
QC_PsApaH_1-342_Stop_rev	ctattattcggacgttacacattttcgaag
Insert_LpApaH_28-426_fo	tgtgcaattcagattccgag
Insert_LpApaH_28-426_fo	ggtacccacgtgatggtg
Gi_LpApaH_28-426_fo	catcaccacagccaggatccgtgtgcaattcagattccgag
Gi_LpApaH_28-426_rev	gcggtttctttaccagactcgagggtaccgctcgagttagtaactcatactatgac
Gi_pRSF-Duet_Lp_fo	gtatgagttactaactcgagcggtaccctcgagtctggtaaagaaac
Gi_pRSF-Duet_Lp_fo	gctcggaatctgaattgcacacggatcctggctgtggtgatg
QC_LpApaH_L414Stop_fo	gtgattgaaaaacagtaactgccgaaag
QC_LpApaH_L414Stop_rev	ctatctttcggcagttactgtttttcaatc
Insert_LcApaH_22-434_fo	tgtctgatccagattccgag
Insert_LcApaH_22-434_rev	ggtacccacgtgatggtg
Gi_LcApaH_22-434_fo	caccacagccaggatccgaattgtctgatccagattccgagtgcc
Gi_LcApaH_22-434_rev	cggtttctttaccagactcgagttacgggctgctggttttgcc
Gi_pRSF-Duet_Lc_fo	gcaaaaccagcagcccgtaactcgagtctggtaaagaaaccgctg
Gi_pRSF-Duet_Lc_rev	ctcggaatctggatcagacaattcggatcctggctgtggtg
QC_LcApaH_N409Stop_fo	gagcattgttctgtaagatctggttc
QC_LcApaH_N409Stop_rev	ctgatgaaccagatcttacagaacaatg

Supplementary Table 3: Peptide substrates with various acyl-chains used in this study. The peptide backbone was derived from histone H3 (APRK$_{acyl}$, H3$_{15-18}$ or TARK$_{acyl}$, H3$_{6-9}$), histone H4 (LGK$_{acyl}$, H4$_{10-12}$) tumor suppressor protein p53 (QPKK$_{acyl}$, p53$_{317-320}$) or DLAT **(**Dihydrolipoyllysine-residue acetyltransferase component of pyruvate dehydrogenase complex; ETDK$_{acyl}$; DLAT$_{256-259}$). The C-terminal lysine side chain carried various acyl-chain types, i.e. acetyl (C-2; K$_{ac}$), propionyl (C-3; K$_{pro}$), butyryl (C-4; K$_{but}$), hexanoly (C-6; K$_{hex}$), octanoyl (C-8; K$_{oct}$), decanoyl (C-10; K$_{dec}$), lauroyl (C-10; K$_{lau}$), myristoyl (C-12; K$_{myr}$), palmitoyl (C-14; K$_{pal}$), crotonyl (K$_{cro}$), L-lactyl (K$_{L-la}$), D-β-hydroxybutyryl K$_{D-bhb}$), succinyl (K$_{suc}$), glutaryl (K$_{glu}$) and biotinyl (K$_{bio}$). The references are given.

**Shorthand**	**Sequence**	**Source**
LGKac	Ac-Leu-Gly-Lys(Ac)-AMC	Bradner *et al.*, 2010[11]
QPKKac	Ac-Gln-Pro-Lys-Lys(Ac)-AMC	Madsen *et al.*, 2016[12]
APRKac	Ac-Ala-Pro-Arg-Lys(Ac)-AMC	Moreno-Yruela *et al.*, 2022[13]
ETDKac	Ac-Glu-Thr-Asp-Lys(Ac)-AMC	Galleano *et al.*, 2016
LGKpro	Ac-Leu-Gly-Lys(Pro)-AMC	Olesen *et al.*, 2018[14]
LGKbut	Ac-Leu-Gly-Lys(But)-AMC	Olesen *et al.*, 2018[14]
QPKKhex	Ac-Gln-Pro-Lys-Lys(Hex)-AMC	Madsen *et al.*, 2016[12]
QPKKoct	Ac-Gln-Pro-Lys-Lys(Oct)-AMC	Madsen *et al.*, 2016[12]
QPKKdec	Ac-Gln-Pro-Lys-Lys(Dec)-AMC	Madsen *et al.*, 2016[12]
TARKdec	Ac-Thr-Ala-Arg-Lys(Dec)-AMC	Madsen *et al.*, 2016[12]
ETDKdec	Ac-Glu-Thr-Asp-Lys(Dec)-AMC	Galleano *et al.*, 2016
QPKKlau	Ac-Gln-Pro-Lys-Lys(Lau)-AMC	Madsen *et al.*, 2016[12]
QPKKmyr	Ac-Gln-Pro-Lys-Lys(Myr)-AMC	Madsen *et al.*, 2016[12]
TARKmyr	Ac-Thr-Ala-Arg-Lys(Myr)-AMC	Madsen *et al.*, 2016[12]
ETDKmyr	Ac-Glu-Thr-Asp-Lys(Myr)-AMC	Galleano *et al.*, 2016
QPKKpal	Ac-Gln-Pro-Lys-Lys(Pal)-AMC	Madsen *et al.*, 2016[12]
LGKcr	Ac-Leu-Gly-Lys(Cr)-AMC	Madsen *et al.*, 2012a[15]
LGK(L-la)	Ac- Leu-Gly-Lys(L-la)-AMC	Moreno-Yruela *et al.*, 2022[13]
LGK(D-la)	Ac- Leu-Gly-Lys(D-la)-AMC	Moreno-Yruela *et al.*, 2022[13]
QPKK(L-la)	Ac-Gln-Pro-Lys-Lys(L-la)-AMC	Moreno-Yruela *et al.*, 2022[13]
QPKK(D-la)	Ac-Gln-Pro-Lys-Lys(D-la)-AMC	Moreno-Yruela *et al.*, 2022[13]
LGK(D-bhb)	Ac- Leu-Gly-Lys(D-bhb)-AMC	Moreno-Yruela *et al.*, 2022[13]
LGKsuc	Ac-Leu-Gly-Lys(Suc)-AMC	Madsen *et al.*, 2012b[16]
LGKglu	Ac-Leu-Gly-Lys(Glu)-AMC	Anderson *et al.*, 2017[17]
LGKbio	Ac-Leu-Gly-Lys(Bio)-AMC	Moreno-Yruela *et al.*, 2018[18]

Supplementary Table 4: Crystallization of the enzymes and complexes crystallized in this study. Shown are the enzymes of different sub-clusters, the crystallization conditions, the cryoprotectant used to freeze the crystals in liquid nitrogen, the time needed until crystals were observed (in days) and the time at which the crystals were harvested. The PDB code is shown under which the structures are deposited in the protein data bank (PDB; https://www.rcsb.org/).

**Protein**	**condition**	**Cryo-protection**	**Crystals observed**	**Harvested**	**PDB entry**
*Kp*HdaH (1b)	12% (w/v) PEG 8000, 10% (v/v) glycerol and 0.5M KCl	with additional 10% glycerol	overnight	overnight	9GLB
*Kp*HdaH H144A (1b)	12% (w/v) PEG 8000, 10% (v/v) glycerol and 0.5M KCl	with additional 10% glycerol	overnight	overnight	9GN1
*Kp*HdaH (1b)•SAHA	12% (w/v) PEG 8000, 10% (v/v) glycerol and 0.5M KCl	with additional 10% glycerol	overnight	overnight following soaking (1h)	9GN6
*Kp*HdaH (1b)•TSA	12% (w/v) PEG 8000, 10% (v/v) glycerol and 0.5M KCl	with additional 10% glycerol	overnight	overnight following soaking (1h)	9GN7
*Rs*PrpH (1b)	4% tacsimate pH 6 12% (w/v) PEG3350	with saturated glucose	5 days	16 days	9GKU
*Rw*DmhA (1b)	0.2 M KF pH 7.3 and 20% (w/v) PEG3350	with 15% (w/v) PEG400	4 days	7 days	9GKW
*Rw*DmhA (1b)•SAHA	0.2 M NaF pH 7.3 20% (w/v) PEG3350	with saturated glucose	3 days	5 days	9GKX
*Vc*HdaH (5b)	1 M Na/K tartrate, 0.1 M imidazole pH 8 0.2 M NaCl	with saturated glucose	9 days	14 days	9GKY
*Vc*HdaH (5b)•SAHA	1.4 M sodium acetate 0.1 M MES pH 6.5	with saturated glucose	12 days	20 days	9GKV
*Ps*ApaH$_{1\text{-}342}$ (4)	0.1 M sodium acetate pH 4.5 20% (w/v) PEG3000	with 15% (w/v) PEG400	10 days	29 days	9GKZ
*Lc*ApaH$_{22\text{-}409}$ (3)	1 M $(NH4)_2SO_4$ 5% (w/v) PEG400 0.1 M Na-MES pH 6.5	2 M $(NH4)_2SO_4$ 10% (w/v) PEG400 0.1 M Na-MES pH 6.5 12% (v/v) glycerol	26 days	30 days	9GL0
*Lp*ApaH (3)	0.2 M calcium acetate 0.1 M HEPES pH 7.5, 18% (w/v) PEG8000	with 12% (w/v) PEG400	6 days	11 days	9GL1

Supplementary Table 5: Data collection and refinement statistics (molecular replacement) for the *Kp*HdaH (1b) wildtype structure of cluster 1b enzymes and for the catalytically inactive mutant *Kp*HdaH (1b) H144A structure.

	*Kp*HdaH (1b) (PDB: 9GLB [https://doi.org/10.2210/pdb9GLB/pdb])	*Kp*HdaH (1b) H144A (PDB: 9GN1 [https://doi.org/10.2210/pdb9GLN1/pdb])
**Data collection**		
Space group	I 2 3	I 2 3
Cell dimensions		
a, *b*, *c* (Å)	147.163 147.163 147.163	146.117 146.117 146.117
α, β, γ (°)	90.0 90.0 90.0	90.0 90.0 90.0
Resolution (Å)	25.24 - 2.1 (2.175 - 2.1) *[1]	46.21 - 2.35 (2.434 - 2.35) *[2]
R_{merge} (%)[a]	14.14 (24.13)	2.628 (23.49)
$I / \sigma I$	15.77 (1.14)	13.44 (1.46)
Completeness (%)	99.84 (99.94)	99.88 (99.91)
Redundancy	19.9 (20.4)	22.6 (22.5)
**Refinement**		
Resolution (Å)	2.1	2.35
No. reflections	30973 (3080)	21754 (1152)
R_{work} / R_{free} [b]	0.1496 (0.2466) / 0.1784 (0.2710)	0.1533 (0.2633)/ 0.2042 (0.3170)
No. atoms	3071	2973
Protein	2818	2815
Ligand/ion	25	21
Water	239	137
No. protein residues	370	370
Ramachandran Plot (%)[c]		
Most favored	95.65	92.93
Additionallly allowed	3.53	4.89
Disfavored	0.82	2.17
Clashscore	4.12	6.09
B-factors	51.10	48.21
Protein	50.65	47.80
Ligand/ion	70.88	56.84
Water	55.27	41.93
R.m.s. deviations		
Bond lengths (Å)	0.004	0.013
Bond angles (°)	0.66	2.30

*[1] One crystal was used to solve the *Kp*HdaH (1b) WT structure by molecular replacement.
*[2] One crystal was used to solve the structure of *Kp*HdaH (1b) H144A by molecular replacement.
a: $R_{merge} = \Sigma_{hkl} \Sigma^{n}_{i=1} |I_i (hkl) - \bar{I} (hkl)| / \Sigma_{hkl} \Sigma^{n}_{i=1} I_i (hkl)$ where I_i (hkl) is the measured intensity of the reflection i at position hkl and $\bar{I}$ (hkl) is the average intensity of the reflection(s) at position hkl[19].
b: $R_{work} = \Sigma |F_o - F_c| / \Sigma F_o$ where F_o and F_c are the observed and calculated structure factor amplitudes. R_{free} is calculated similarly to R_{work} using random 5% of working set of reflections[20].
c: MolProbity[21].

Supplementary Table 6: Data collection and refinement statistics (molecular replacement) for the hydroxamate inhibitor complexes *Kp*HdaH (1b)•SAHA and *Kp*HdaH (1b)•TSA.

	*Kp*HdaH (1b)•SAHA (PDB: 9GN6 [https://doi.org/10.2210/pdb9GN6/pdb])	*Kp*HdaH (1b)•TSA (PDB: 9GN7 [https://doi.org/10.2210/pdb9GN7/pdb])
**Data collection**		
Space group	I 2 3	I 2 3
Cell dimensions		
a, *b*, *c* (Å)	146.206 146.206 146.206	146.57 146.57 146.57
α, β, γ (°)	90.0 90.0 90.0	90.0 90.0 90.0
Resolution (Å)	42.21 - 1.95 (2.02 - 1.95) *[1]	46.35 - 2.18 (2.258 - 2.18) *[2]
R_{merge} (%)[a]	11.63 (37.52)	2.781 (28.25)
$I / \sigma I$	22.62 (1.25)	12.16 (2.58)
Completeness (%)	99.97 (100.00)	99.97 (99.96)
Redundancy	39.5 (41.2)	2.0 (2.0)
**Refinement**		
Resolution (Å)	1.95	2.18
No. reflections	37871 (2629)	27454 (2367)
R_{work} / R_{free} [b]	0.1373 (0.2950)/ 0.1756 (0.3161)	0.1478 (0.2210)/ 0.1862 (0.2390)
No. atoms	3081	2985
Protein	2827	2822
Ligand/ion	22	44
Water	232	119
No. protein residues	369	369
Ramachandran Plot (%)[c]		
Most favored	95.11	94.29
Additionallly allowed	4.35	5.16
Disfavored	0.54	0.54
Clashscore	2.49	2.65
B-factors	48.60	43.95
Protein	48.38	43.23
Ligand/ion	53.06	59.37
Water	56.12	41.49
R.m.s. deviations		
Bond lengths (Å)	0.012	0.014
Bond angles (°)	1.94	2.24

*[1] One crystal was used to solve the *Kp*HdaH (1b)•SAHA structure by molecular replacement.
*[2] One crystal was used to solve the *Kp*HdaH (1b)•TSA structure by molecular replacement.
a: $R_{merge} = \Sigma_{hkl} \Sigma^{n}_{i=1} |I_i (hkl) - \bar{I} (hkl)| / \Sigma_{hkl} \Sigma^{n}_{i=1} I_i (hkl)$ where I_i (hkl) is the measured intensity of the reflection i at position hkl and $\bar{I}$ (hkl) is the average intensity of the reflection(s) at position hkl[19].
b: $R_{work} = \Sigma |F_o - F_c| / \Sigma F_o$ where F_o and F_c are the observed and calculated structure factor amplitudes. R_{free} is calculated similarly to R_{work} using random 5% of working set of reflections[20].
c: MolProbity[21].

Supplementary Table 7: Data collection and refinement statistics (molecular replacement) for the *Rw*DmhA (1b) octanoic acid structure and for the hydroxamate inhibitor complex *Rw*DmhA (1b)•SAHA.

	*Rw*DmhA (PDB: 9GKW [https://doi.org/10.2210/pdb9GKW/pdb])	*Rw*DmhA•SAHA (PDB: 9GKX [https://doi.org/10.2210/pdb9GKX/pdb])
**Data collection**		
Space group	C 1 2 1	C 1 2 1
Cell dimensions		
a, *b*, *c* (Å)	131.62 144.03 92.96	132.12 144.96 92.66
α, β, γ (°)	90.0 92.1 90.0	90.0 91.81 90.0
Resolution (Å)	54.64 - 2.1 (2.12 - 2.1) *[1]	46.31 - 1.75 (1.79 - 1.75) *[2]
R_{merge} (%)[a]	20.26 (97.36)	19.51 (146.3)
$I / \sigma I$	7.26 (1.57)	4.90 (0.64)
Completeness (%)	84.54 (85.80)	99.70 (99.05)
Redundancy	4.6 (4.3)	3.5 (3.3)
**Refinement**		
Resolution (Å)	2.1	1.75
No. reflections	393612 (18427)	174373 (11513)
R_{work} / R_{free} [b]	0.1719 (0.239)/ 0.2173 (0.273)	0.1626 (0.3732)/ 0.1903 (0.4108)
No. atoms	12389	13142
Protein	11187	11200
Ligand/ion	102	162
Water	1100	1780
No. protein residues	1469	1464
Ramachandran Plot (%)[c]		
Most favored	97.40	97.25
Additionallly allowed	2.40	2.75
Disfavored	0.21	0.00
Clashscore	7.45	3.43
B-factors	20.69	21.94
Protein	19.75	19.92
Ligand/ion	52.53	28.69
Water	27.34	34.03
R.m.s. deviations		
Bond lengths (Å)	0.027	0.007
Bond angles (°)	2.21	0.85

*[1] One crystal was used to solve the *Rw*DmhA structure by molecular replacement.
*[2] One crystal was used to solve the *Rw*DmhA•SAHA structure by molecular replacement.
a: $R_{merge} = \Sigma_{hkl} \ \Sigma^{n}{}_{i=1} |I_i\ (hkl) - \bar{I}\ (hkl)| / \Sigma_{hkl} \ \Sigma^{n}{}_{i=1} I_i\ (hkl)$ where I_i (hkl) is the measured intensity of the reflection i at position hkl and $\bar{I}$ (hkl) is the average intensity of the reflection(s) at position hkl[19].
b: $R_{work} = \Sigma |F_o - F_c| / \Sigma F_o$ where F_o and F_c are the observed and calculated structure factor amplitudes. R_{free} is calculated similarly to R_{work} using random 5% of working set of reflections[20].
c: MolProbity[21].

Supplementary Table 8: Data collection and refinement statistics (molecular replacement) for the apo-structure of *Vc*HdaH (5b) and for the hydroxamate inhibitor complex *Vc*HdaH (5b)•SAHA.

	*Vc*HdaH (5b) (PDB: 9GKY [https://doi.org/10.2210/pdb9GKY/pdb])	*Vc*HdaH (5b)•SAHA (PDB: 9GKV [https://doi.org/10.2210/pdb9GKV/pdb])
**Data collection**		
Space group	P 1 21 1	P 21 21 21
Cell dimensions		
a, *b*, *c* (Å)	51.11 51.15 108.82	50.92 81.53 133.17
α, β, γ (°)	90.0 102.22 90.0	90.0 90.0 90.0
Resolution (Å)	26.39 - 1.129 (1.14 - 1.13) *[1]	40.45 - 1.643 (1.68 - 1.64) *[2]
R_{merge} (%)[a]	5.977 (188.8)	10.44 (180.9)
$I / \sigma I$	5.56 (0.36)	7.31 (0.23)
Completeness (%)	94.57 (84.52)	77.17 (11.89)
Redundancy	1.9 (1.9)	5.4 (1.6)
**Refinement**		
Resolution (Å)	1.13	1.64
No. reflections	194387 (5740)	52756 (531)
R_{work} / R_{free} [b]	0.1576 (0.357)/ 0.1789 (0.364)	0.2081 (0.563)/ 0.2130 (0.558)
No. atoms	6006	5335
Protein	5148	4935
Ligand/ion	62	37
Water	796	363
No. protein residues	621	618
Ramachandran Plot (%)[c]		
Most favored	98.06	97.39
Additionallly allowed	1.94	2.61
Disfavored	0.00	0.00
Clashscore	5.86	4.57
B-factors	14.14	29.85
Protein	11.71	29.12
Ligand/ion	16.72	48.57
Water	29.67	37.96
R.m.s. deviations		
Bond lengths (Å)	0.013	0.014
Bond angles (°)	1.66	2.12

*[1] One crystal was used to solve the *Vc*HdaH (5b) structure by molecular replacement.
*[2] One crystal was used to solve the structure of *Vc*HdaH (5b)•SAHA by molecular replacement.
a: $R_{merge} = \Sigma_{hkl} \Sigma^{n}_{i=1} |I_i (hkl) - \bar{I} (hkl)| / \Sigma_{hkl} \Sigma^{n}_{i=1} I_i (hkl)$ where I_i (hkl) is the measured intensity of the reflection i at position hkl and $\bar{I}$ (hkl) is the average intensity of the reflection(s) at position hkl[19].
b: $R_{work} = \Sigma |F_o - F_c| / \Sigma F_o$ where F_o and F_c are the observed and calculated structure factor amplitudes. R_{free} is calculated similarly to R_{work} using random 5% of working set of reflections[20].
c: MolProbity[21].

Supplementary Table 9: Data collection and refinement statistics (molecular replacement) for the *Rs*PrpH (1b) structure and for the *Ps*ApaH (4) structure.

	*Rs*PrpH (1b) (PDB: 9GKU [https://doi.org/10.2210/pdb9GKU/pdb])	*Ps*ApaH$_{1-342}$ (4) (PDB: 9GKZ [https://doi.org/10.2210/pdb9GKZ/pdb])
**Data collection**		
Space group	P 1	P 21 21 2
Cell dimensions		
a, *b*, *c* (Å)	103.861 103.698 104.499	147.342 48.858 82.249
α, β, γ (°)	113.6 93 120.61	90.0 90.0 90.0
Resolution (Å)	44.56 - 1.481 (1.5 - 1.48) *[1]	73.67 - 2.716 (2.89 - 2.72) *[2]
R_{merge} (%)[a]	7.0 (137.3)	8.563 (49.85)
$I / \sigma I$	9.1 (1.1)	7.04 (1.22)
Completeness (%)	90.8 (64.6)	99.90 (100.00)
Redundancy	1.8 (1.5)	2.0 (2.0)
**Refinement**		
Resolution (Å)	1.48	2.72
No. reflections	493363 (12890)	16666 (2708)
R_{work} / R_{free} [b]	0.1528 (0.335)/ 0.1834 (0.334)	0.1556 (0.2166)/ 0.2324 (0.2976)
No. atoms	24997	5145
Protein	22739	4986
Ligand/ion	188	30
Water	2070	129
No. protein residues	2960	642
Ramachandran Plot (%)[c]		
Most favored	96.60	94.64
Additionallly allowed	3.12	5.36
Disfavored	0.27	0.00
Clashscore	5.17	7.92
B-factors	28.54	35.79
Protein	27.69	35.82
Ligand/ion	36.43	44.62
Water	37.26	32.87
R.m.s. deviations		
Bond lengths (Å)	0.013	0.008
Bond angles (°)	1.70	0.94

*[1] One crystal was used to solve the *Rs*PrpH (1b) structure by molecular replacement.
*[2] One crystal was used to solve the structure of *Ps*ApaH (4) by molecular replacement.
a: $R_{merge} = \Sigma_{hkl} \Sigma^{n}_{i=1} |I_i(hkl) - \bar{I}(hkl)| / \Sigma_{hkl} \Sigma^{n}_{i=1} I_i(hkl)$ where $I_i(hkl)$ is the measured intensity of the reflection i at position hkl and $\bar{I}$ (hkl) is the average intensity of the reflection(s) at position hkl[19].
b: $R_{work} = \Sigma |F_o - F_c| / \Sigma F_o$ where F_o and F_c are the observed and calculated structure factor amplitudes. R_{free} is calculated similarly to R_{work} using random 5% of working set of reflections[20].
c: MolProbity[21].

Supplementary Table 10: Data collection and refinement statistics (molecular replacement) for the structure *Lc*ApaH and for the *Lp*ApaH structure, both enzymes classified into cluster 3.

	*Lc*ApaH (PDB: 9GL0 [https://doi.org/10.2210/pdb9GL0/pdb])	*Lp*ApaH (PDB: 9GL1 [https://doi.org/10.2210/pdb9GL1/pdb])
**Data collection**		
Space group	P 32 2 1	P 41 2 2
Cell dimensions		
a, *b*, *c* (Å)	113.564 113.564 90.426	109.121 109.121 400.511
α, β, γ (°)	90.0 90.0 120.0	90.0 90.0 90.0
Resolution (Å)	43.2 - 2.4 (2.43 - 2.4) *[1]	45.96 - 2.701 (2.73 - 2.7)
R_{merge} (%)[a]	9.656 (137.7)	18.5 (365.9)
$I / \sigma I$	6.22 (0.45)	4.85 (0.27)
Completeness (%)	98.59 (97.53)	94.60 (27.26)
Redundancy	8.2 (1.4)	10.3 (1.1)
**Refinement**		
Resolution (Å)	2.4	2.7
No. reflections	26336 (867)	64002 (607)
R_{work} / R_{free} [b]	0.2373 (0.5791)/ 0.2633 (0.5272)	0.2403 (0.6215)/ 0.3160 (0.6736)
No. atoms	3141	12721
Protein	3130	12688
Ligand/ion	3	15
Water	8	18
No. protein residues	389	1568
Ramachandran Plot (%)[c]		
Most favored	87.86	87.88
Additionallly allowed	10.86	9.87
Disfavored	1.29	2.24
Clashscore	21.44	17.53
B-factors	123.57	113.81
Protein	123.60	113.83
Ligand/ion	112.79	106.77
Water	114.03	103.69
R.m.s. deviations		
Bond lengths (Å)	0.010	0.009
Bond angles (°)	1.14	1.08

*[1] One crystal was used to solve the *Lc*ApaH structure by molecular replacement.
*[2] One crystal was used to solve the *Lp*ApaH structure by molecular replacement.
a: $R_{merge} = \Sigma_{hkl} \Sigma^{n}_{i=1} |I_i (hkl) - \bar{I} (hkl)| / \Sigma_{hkl} \Sigma^{n}_{i=1} I_i (hkl)$ where I_i (hkl) is the measured intensity of the reflection i at position hkl and $\bar{I}$ (hkl) is the average intensity of the reflection(s) at position hkl[19].
b: $R_{work} = \Sigma |F_o - F_c| / \Sigma F_o$ where F_o and F_c are the observed and calculated structure factor amplitudes. R_{free} is calculated similarly to R_{work} using random 5% of working set of reflections[20].
c: MolProbity[21].

Supplementary References

1. Notredame, C., Higgins, D.G. & Heringa, J. T-Coffee: A novel method for fast and accurate multiple sequence alignment. *J Mol Biol* **302**, 205-17 (2000).
2. Robert, X. & Gouet, P. Deciphering key features in protein structures with the new ENDscript server. *Nucleic Acids Res* **42**, W320-4 (2014).
3. The PyMOL Molecular Graphics System, Version 3.0, Schrödinger, LLC.
4. Jurrus, E. et al. Improvements to the APBS biomolecular solvation software suite. *Protein Sci* **27**, 112-128 (2018).
5. Herring, C.D., Glasner, J.D. & Blattner, F.R. Gene replacement without selection: regulated suppression of amber mutations in Escherichia coli. *Gene* **311**, 153-63 (2003).
6. Wilksch, J.J. et al. MrkH, a novel c-di-GMP-dependent transcriptional activator, controls Klebsiella pneumoniae biofilm formation by regulating type 3 fimbriae expression. *PLoS Pathog* **7**, e1002204 (2011).
7. Brisse, S. et al. Virulent clones of Klebsiella pneumoniae: identification and evolutionary scenario based on genomic and phenotypic characterization. *PLoS One* **4**, e4982 (2009).
8. Nassif, X. & Sansonetti, P.J. Correlation of the virulence of Klebsiella pneumoniae K1 and K2 with the presence of a plasmid encoding aerobactin. *Infect Immun* **54**, 603-8 (1986).
9. Studier, F.W. & Moffatt, B.A. Use of bacteriophage T7 RNA polymerase to direct selective high-level expression of cloned genes. *J Mol Biol* **189**, 113-30 (1986).
10. Hanahan, D. DNA Cloning: A Practical Approach. *Glover, D. M. (ed.)* **1**, 109 (1985).
11. Bradner, J.E. et al. Chemical phylogenetics of histone deacetylases. *Nat Chem Biol* **6**, 238-243 (2010).
12. Madsen, A.S. et al. Investigating the Sensitivity of NAD+-dependent Sirtuin Deacylation Activities to NADH. *J Biol Chem* **291**, 7128-41 (2016).
13. Moreno-Yruela, C. et al. Class I histone deacetylases (HDAC1-3) are histone lysine delactylases. *Sci Adv* **8**, eabi6696 (2022).
14. Olesen, S.V., Rajabi, N., Svensson, B., Olsen, C.A. & Madsen, A.S. An NAD(+)-Dependent Sirtuin Depropionylase and Deacetylase (Sir2La) from the Probiotic Bacterium Lactobacillus acidophilus NCFM. *Biochemistry* **57**, 3903-3915 (2018).
15. Madsen, A.S. & Olsen, C.A. Profiling of substrates for zinc-dependent lysine deacylase enzymes: HDAC3 exhibits decrotonylase activity in vitro. *Angew Chem Int Ed Engl* **51**, 9083-7 (2012).
16. Madsen, A.S. & Olsen, C.A. Substrates for efficient fluorometric screening employing the NAD-dependent sirtuin 5 lysine deacylase (KDAC) enzyme. *J Med Chem* **55**, 5582-90 (2012).
17. Anderson, K.A. et al. SIRT4 Is a Lysine Deacylase that Controls Leucine Metabolism and Insulin Secretion. *Cell Metab* **25**, 838-855 e15 (2017).
18. Moreno-Yruela, C., Galleano, I., Madsen, A.S. & Olsen, C.A. Histone Deacetylase 11 Is an epsilon-N-Myristoyllysine Hydrolase. *Cell Chem Biol* **25**, 849-856 e8 (2018).
19. Diederichs, K. & Karplus, P.A. Improved R-factors for diffraction data analysis in macromolecular crystallography. *Nat Struct Biol* **4**, 269-75 (1997).
20. Brunger, A.T. Free R value: cross-validation in crystallography. *Methods Enzymol* **277**, 366-96 (1997).
21. Chen, V.B. et al. MolProbity: all-atom structure validation for macromolecular crystallography. *Acta Crystallogr D Biol Crystallogr* **66**, 12-21 (2010).
