# Supplementary material for: Distribution and diversity of classical deacylases in bacteria
Source: Nat Commun. 2024 Nov 3;15:9496. doi: 10.1038/s41467-024-53903-0 (PMC11532494; doi:10.1038/s41467-024-53903-0)
Supplement: Supplementary file 2 — Description of Additional Supplementary Files [file 41467_2024_53903_MOESM2_ESM.pdf]

Description of Additional Supplementary Files

Supplementary Data 1: All sequences identified by the screening of the UniProt database applying the Generalized Profile.

Supplementary Data 2: Sequences of the catalytic domains containing the catalytic general acid/base His (*Kp*HdaH (1b): His144) filtered from sequences listed in Supplementary Data 2.

Supplementary Data 3: Sequences of the catalytic domains of about 5973 representative members selected by the 'cd-hit' program by removing duplicates and highly-similar (>60% identity) and all containing the catalytic general acid/base His (*Kp*HdaH (1b): His144) filtered from sequences listed in Supplementary Data 3.

Supplementary Data 4: Sequences used for construction of the phylogenetic tree. The phylogenetic tree is based on a multiple sequence alignment generated with with MAFFT (L-Ins-I modus). Before using the alignment for tree construction, columns with more than 90% of gaps were removed. The alignment was used to generate a neighbor-joining tree with the programme belvu[1]. This was converted to newick format and the phylogenetic tree was visualized with iTOL[2,3].

Supplementary Data 5: Sequences used for construction of the phylogenetic tree in newick format. Sequences from which regions with >90% gaps were removed were used to generate a neighbor-joining tree with the program belvu[1]. This was converted to newick format and the phylogenetic tree was visualized with iTOL[2,3].

Supplementary Data 6: Structural alignments of selected representatives of the clusters.
To analyze the structural similarities of enzymes within the sub-clusters of a main cluster and of enzymes of the main clusters 1-5 structural alignments were performed. The alignment was done by superimposing the structures on a reference structure shown above. The reference structure was one of the experimentally solved structures of the bacterial deacylases presented here. If this was not possible, i.e. for cluster 2, we selected the structure of human HDAC2 (cluster 2; PDB: 4LY1 [https://doi.org/10.2210/pdb4LY1/pdb]) as reference structure. The r.m.s.d. values show the close structural similarities of enzymes of different sub-clusters within a main cluster. Moreover, the structural alignment of representative enzymes of the five clusters also shows close structural similarity between the main clusters. For almost all structural alignments we observed r.m.s.d. values close to 1Å or lower. We observed two exceptions, i.e. the eukaryotic enzyme HdaH of *Malassezia pachydermatis* (1f) of zoophilic yeast in comparison to the bacterial *Kp*HdaH (1b) with an r.m.s.d value of 7 Å in the comparison of cluster 1 enzymes and the similarity of the cluster 3 enzyme of *L. cherrii* HdaH (3) in the comparison of enzymes representing the main-clusters with r.m.s.d to *Kp*HdaH (1b) of 5.5 Å. *M. pachydermatis* (1f) shows an additional structural feature within the catalytic domain, i.e. an additional α-helical insert, a β-hairpin, and a long α-helical N-terminal domain preceding the catalytic domain, explaining the observed structural difference. The structural dissimilarity of the cluster 3 enzymes is due to the additional structural features, i.e. the extended β-sheet and the additional α-helices compared in the catalytic core domain. If not indicated by the PDB-code, AlphaFold2 structural models were used. The corresponding UniProt numbers of the enzymes are listed. The structural alignments were done using PyMOL[4].

Supplementary Data 7: ChemDraw file for chemical structures of peptides and acyl chain types used as substrate backbones for deacylation by selected bacterial deacylases.
The ChemDraw file shows chemical structures of peptides with varying sequences used to study substrate preference for various bacterial deacylases. The peptides used were derived from histone H3 (APRK$_{acyl}$, H3$_{15\text{-}18}$ or TARK$_{acyl}$, H3$_{6\text{-}9}$), histone H4 (LGK$_{acyl}$, H4$_{10\text{-}12}$) tumor suppressor protein p53 (QPKK$_{acyl}$, p53$_{317\text{-}320}$) and DLAT **(**Dihydrolipoyllysine-residue acetyltransferase component of pyruvate dehydrogenase complex; ETDK$_{acyl}$; DLAT$_{256\text{-}259}$). The peptides are modified with a C-terminally attached AMC (7-amino-4-methylcoumarin) fluorophore being released upon trypsin cleavage (Fluor-deLys-assay). The AMC fluorophore is quenched in the peptide but is fluorescent in its free form. Furthermore, the ChemDraw file encompasses drawings of chemical structures of the different acyl chain types used in this study i.e. acetyl (C-2; K$_{ac}$), propionyl (C-3; K$_{pro}$), butyryl (C-4; K$_{but}$), medium-sized aliphatic acylations, i.e. hexanoly (C-6; K$_{hex}$), octanoyl (C-8; K$_{oct}$), decanoyl (C-10; K$_{dec}$), longer-chain aliphatic acylations, i.e. lauroyl (C-10; K$_{lau}$), myristoyl (C-12; K$_{myr}$) and palmitoyl (C-14; K$_{pal}$), the unsaturated crotonyl (K$_{cro}$), the branched acylations L-lactyl (K$_{L\text{-}la}$), D-β-hydroxybutyryl K$_{D\text{-}bhb}$), charged-acylations,

i.e. succinyl (K_{suc}), glutaryl (K_{glu}) and biotinyl (K_{bio}) (Fig. 3a; Supplementary Fig. 5; Supplementary Fig. 6). Charges were shown as expected at physiological pH.

Supplementary Data 8: ChemDraw file for chemical structures of deacylase hydroxamate inhibitors SAHA, trichostatin A (TSA), benzamide entinostat (MS-275) and the cyclic peptides apicidin A and trapoxin A.

References:

1. Sonnhammer, E.L. & Hollich, V. Scoredist: a simple and robust protein sequence distance estimator. *BMC Bioinformatics* **6**, 108 (2005).
2. Letunic, I. & Bork, P. Interactive Tree Of Life (iTOL) v5: an online tool for phylogenetic tree display and annotation. *Nucleic Acids Res* **49**, W293-W296 (2021).
3. Letunic, I. & Bork, P. Interactive Tree Of Life (iTOL): an online tool for phylogenetic tree display and annotation. *Bioinformatics* **23**, 127-8 (2007).
4. The PyMOL Molecular Graphics System, Version 3.0, Schrödinger, LLC.
